# Development of xanthone derivatives as effective broad-spectrum antimicrobials: Disrupting cell wall and inhibiting DNA synthesis

**DOI:** 10.1126/sciadv.adt4723

**Published:** 2025-03-05

**Authors:** Haizhou Li, Wen-Tyng Kang, Yujiahui Zheng, Yonglv He, Rongcui Zhong, Shanfang Fang, Weijie Wen, Shouping Liu, Shuimu Lin

**Affiliations:** Guangdong Provincial Key Laboratory of Molecular Target and Clinical Pharmacology, School of Pharmaceutical Sciences and the Affiliated Qingyuan Hospital (Qingyuan People’s Hospital), Guangzhou Medical University, Guangzhou 511436, China.

## Abstract

Discovering potent antibiotics is of critical importance due to the substantial increases of microbial resistance. Xanthones are intriguing sources of antimicrobials, despite a scarcity of extensive investigations into their mechanisms of action. Here, we reported the development of a series of xanthone derivatives, among which compound **XT17** displayed strong broad-spectrum antibacterial activity, weak hemolytic activity, and low cytotoxicity against mammalian cell lines, low frequencies of drug resistance, and potent in vivo efficacy in *Staphylococcu aureus*– or *Pseudomonas aeruginosa*–induced murine corneal infection models. Compound **XT17** presented a multifaceted mode of actions, involving the disruption of cell wall by interacting with lipoteichoic acid or lipopolysaccharides and the suppression of DNA synthesis. A further docking study confirmed the capability of compound **XT17** to form a stable complex with the bacterial gyrase enzyme. This work could offer an innovative design strategy for developing broad-spectrum therapeutic agents against drug-resistant bacteria.

## Teaser

XT17, a broad-spectrum antimicrobial, effectively combats drug resistance with low toxicity and unique mechanisms.

## INTRODUCTION

The emergence of difficult-to-treat multidrug-resistant (MDR) bacterial strains is a global health concern (*1*). Since the MDR can be executed by multiple mechanisms, getting past this barrier is a challenging task (*2*). It is urgently necessary to discover and develop promising broad-spectrum antimicrobial agents with low bacterial resistance to increase the effectiveness of antibiotics against pathogenic microorganisms (*3*). One of the key tactics for resolving the MDR dilemma is to adapt structural features from natural products (*4*, *5*). In the field of anti-infectious agent research in particular, natural products have been indispensable in the search for small-molecule drugs (*6*–*8*). Natural products contain large fractions of sp^3^-hybridized bridgehead atoms, chiral centers, and diverse pharmacophores, which have essential fundamental molecular frameworks that serve as preliminary notions for drug discovery (*9*). In this state, the finding of potent antibacterial pharmacophores would be necessary for the development of antimicrobial agents (*10*, *11*).

Xanthones, also known as 9*H*-xanthen-9-one, are naturally occurring heterotricyclic compounds that have been isolated from a wide variety of plants, fungi, and lichens (*12*). Because of their diverse structural makeup, these secondary metabolites exhibit several important pharmacological properties, such as antimicrobial (*13*), antioxidant (*14*), anticarcinogenic (*15*), antidiabetic (*16*), and anti-inflammatory (*17*) effects. Xanthones are chemically made up of an oxygen-containing dibenzo-γ-pyrone heterocyclic scaffold (*18*). Driven by this “privileged structure,” it is thought to be a promising and intriguing structural scaffold for drug development as it could offer a broad range of diverse substitutions modulating different biological responses (*19*, *20*). The interesting structure and biological efficacy have prompted many researchers to synthesize xanthone derivatives as prospective drug candidates (*21*–*24*). Several studies have been published highlighting the antimicrobial activity of synthetic xanthone derivatives; however, only a small number of these studies have undergone comprehensive evaluation to determine their exact mechanism of action (*20*, *25*, *26*).

Of all the xanthones reported, α-mangostin (α-MN), originating from the pericarp, bark, and dried sap of mangosteen, is among the extensively researched and discovered xanthones (*27*). It has been shown to have a broad range of bioactivities, including antibacterial, antifungal, and antitumoral properties (*28*, *29*). More amphiphilic xanthone derivatives with structural modifications on C1, C3, and C6 hydroxyl groups of the parent compound α-MN are reported as a result of its antibacterial scaffold with the peptidomimetics biomimicking design of cationic antimicrobial peptides (*30*–*32*). For example, Zou *et al.* (*30*) reported the synthesis of xanthone derivatives as antimicrobials by cationic modification of the C3 and C6 hydroxyl groups of α-MN with high p*K*_a_ (where *K*_a_ is the acid dissociation constant) values of amine groups, which exhibited potent antimicrobial properties against Gram-positive bacteria. Another study by Lu *et al*. (*33*) reported that structural modifications at positions C1, C3, or C6 hydroxyl groups of α-MN resulted in xanthone derivatives with increased antimicrobial activity and higher selectivity against methicillin-resistant *Staphylococcus aureus* (MRSA). Previously, our group synthesized several α-MN–based peptidomimetics with effective activities against MDR Gram-positive bacterial infections. However, chemical modification of α-MN still has several drawbacks, including high hemolytic activity, high production costs, and low antibacterial activity against Gram-negative bacteria (*30*). Gram-negative bacterial infections pose a global threat to human health, and the scarcity of potent antimicrobial agents targeting Gram-negative pathogens in clinical development has led to the rapid depletion of the arsenal against Gram-negative bacterial infections (*34*, *35*). Since α-MN has been shown to have antibacterial activity, especially against Gram-positive bacteria (*30*), it is most plausible that synthetic modification of the xanthone compounds would be produced with enhanced antibacterial activity against Gram-negative pathogens.

**Fig. 1.**
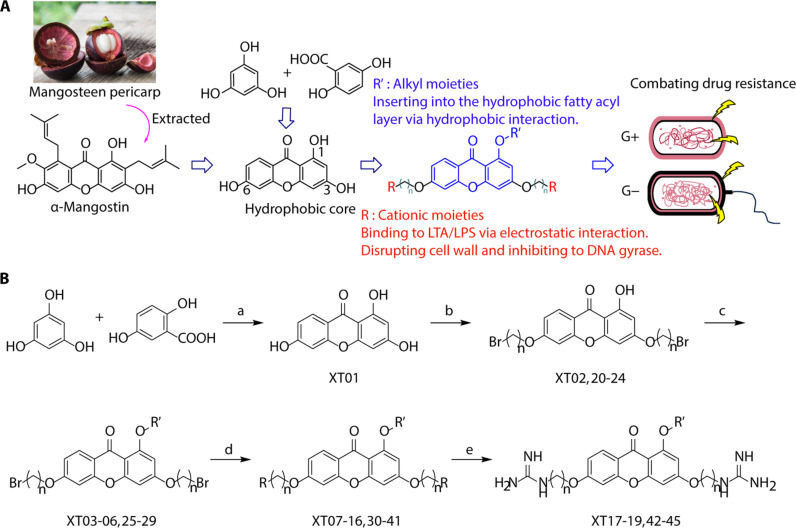
Design and synthesis of xanthone derivatives. (**A**) Design concept of cationic xanthone derivatives. (**B**) General chemical synthesis schemes of xanthone derivatives **XT01-45**. Reagents and conditions: [(B), a] Eaton’s reagent, 80°C, 1 hour; [(B), b] α,ω-dibromoalkanes, K_2_CO_3_, acetone, reflux, 6 hours; [(B), c] 1-iodoalkanes or 1-bromoolefin, Cs_2_CO_3_, acetone, reflux, overnight; [(B), d] corresponding amines, *N*,*N*′-dimethylformamide (DMF), room temperature (RT), 24 hours; [(B), e] 1*H*-pyrazole-1-carboxamidine hydrochloride, DIPEA, DMF, RT, overnight.

The negatively charged lipoteichoic acid (LTA) and lipopolysaccharide (LPS) are the main components of the cell wall of Gram-positive bacteria and the outer membrane (OM) of Gram-negative bacteria, respectively (*36*, *37*). Targeting LTA is also an attractive drug target given its critical importance for cellular integrity, cell division, and host inflammation (*38*, *39*). Bacterial endotoxin LPS are the main components of the OM of Gram-negative bacteria, composed of three key components, one of which is the highly conserved hydrophobic domain lipid A (*40*, *41*). LPS is crucial for bacterial survival by establishing an effective permeability barrier through the cross-linking of lipid A molecules with calcium or magnesium divalent cations (*42*, *43*). Thus, cationic peptides can disrupt the organization of the OM and increase its permeability by weakening the binding sites of divalent cations (*44*). On the basis of the ‌above idea, we first synthesized the 1,3,6-trihydroxyphenylxanthone and then attached hydrophobic chains at the C1 position to disrupt the intramolecular hydrogen bonds and enhance the hydrophobicity of the derivatives. Then, we attached cationic groups with high p*K*_a_ values (such as guanidine groups) to the C3 and C6 positions. We hypothesized that the cationic xanthone derivatives could electrostatically bind to the LPS of the Gram-negative bacterial OM, and the hydrophobic chains insert its hydrophobic terminal acyl fatty layer, weakening the binding sites for divalent cations on lipid A, increasing the permeability of the OM, and resulting in “self-promoted uptake” of this compound into the OM. Similarly, cationic xanthone derivatives containing hydrophobic tails could damage the integrity of the cell walls of Gram-positive bacteria composed of LTA through electrostatic interactions. Once inside the cell, the cationic compounds might bind to intracellular components such as nucleic acids and enzymes, affecting DNA synthesis and ultimately leading to bacterial cell deaths (Fig. 1A). Given the aforementioned constraints, a series of xanthone-derived antimicrobial agents were designed and synthesized. The xanthone scaffold serves as the core precursor for a further chemically modification at its phenolic hydroxyl positions to improve the pharmacological properties of xanthone derivatives against both Gram-positive and Gram-negative bacteria. The candidate compound **XT17**, identified through a structure-activity relationship study of these synthesized compounds, showed strong bactericidal properties and potent in vitro and in vivo efficacy. A comprehensive transcriptomic analysis of the candidate compound **XT17** was assessed using the high-throughput RNA sequencing (RNA-seq) technique to gain insight into the antibacterial mechanisms. A molecular docking study was adopted to discover the binding modes of this candidate compound **XT17**. This efficacy stems from a dual-action mechanism: cell wall destruction and DNA gyrase inhibition. This highlights their potential as powerful, low-toxicity xanthone-based agents against drug-resistant bacteria, providing unique insights for developing a distinct class of broad-spectrum therapeutic agents.

## RESULTS

### Synthesis of xanthone-based compounds

The chemical structures and the synthetic route of xanthone-based compounds are outlined in Fig. 1B. Initially, phloroglucinol and 2,4-dihydroxybenzoic acid were dissolved in Eaton’s reagent and reacted at 80°C for 1 hour to yield **XT01**. Subsequently, **XT01** was reacted with the corresponding α, ω-dibromoalkanes under alkaline conditions (K_2_CO_3_) to produce intermediates **XT02** and **XT20-24**. Then, **XT02** and **XT20-24** were alkylated with 1-iodoalkanes or 1-bromoalkenes under alkaline conditions (Cs_2_CO_3_) to yield compounds **XT03-06** and **XT25-29**. Compounds **XT07-16** and **XT30-41** were prepared by the treatment of **XT03-06** and **XT25-29** with the corresponding amines. Compounds **XT17-19** and **XT42-45** containing guanidine groups were obtained by treating primary amine-substituted compounds with 1*H*-pyrazole-1-carboxamide hydrochloride in the presence of *N,N*-diisopropylethylamine (DIPEA). All synthesized compounds were purified to more than 95% purity by high-performance liquid chromatography (HPLC) and characterized via nuclear magnetic resonance spectroscopy and high-resolution mass spectrometry.

### Antimicrobial susceptibility profiles and hemolytic activities

We first evaluated the in vitro antimicrobial potential of xanthone-based derivatives against three Gram-positive bacterial strains [*S. aureus* American Type Culture Collection (ATCC) 29213, MRSA N315, and MRSA National Collection of Type Cultures (NCTC) 10442] and one Gram-negative strain (*Escherichia coli* ATCC 25922) by determining their minimum inhibitory concentrations (MICs). Concurrently, hemolytic activity (HC_50_) against rabbit erythrocytes was assessed to gauge their selectivity for mammalian cell membranes (Table 1). Xanthone derivatives, which exhibited potent activity against *E. coli* ATCC 25922 with MICs ≤ 3.125 μg/ml, were further tested for their MICs against additional five Gram-negative strains including *Pseudomonas aeruginosa* ATCC 9027, *Acinetobacter baumannii* ATCC 17978, *A. baumannii* R2889, *Klebsiella pneumoniae* ATCC 10031, and *K. pneumoniae* ATCC 14581 (Table 2).

**Table 1.** MIC and hemolytic activities of a series of xanthone derivatives Cmpd, compound; VAN, vancomycin; AMO, amoxicillin; ND, not determined.

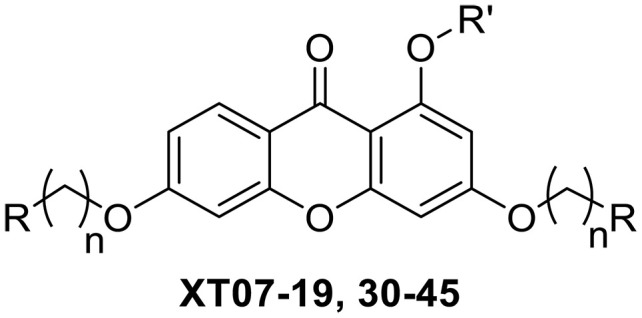								
Cmpd	R′	R	*n*	MIC_90_ (μg/ml)^*^				HC_50_^†^ (μg/ml)
				*S. aureus* ATCC 29213	MRSA N315	MRSA NCTC 10442	*E. coli* ATCC 25922	
**XT01**	H	H	–	50	50	50	>100	>200
**XT07**	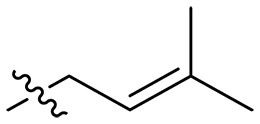	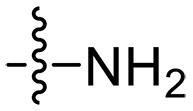	3	12.5	12.5	12.5	25	>200
**XT08**	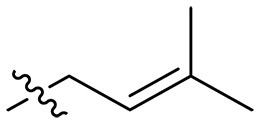	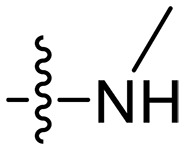	3	12.5	12.5	12.5	25	>200
**XT09**	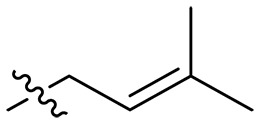	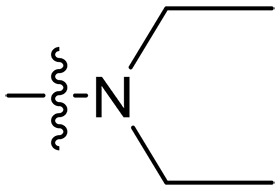	3	6.25	12.5	6.25	50	>200
**XT10**	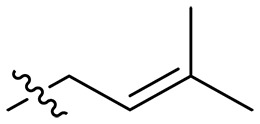	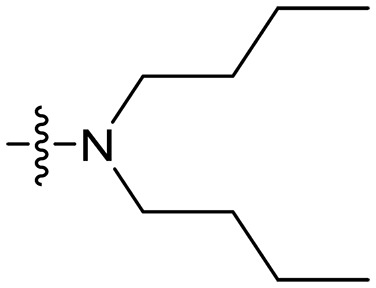	3	12.5	6.25	12.5	>50	>200
**XT11**	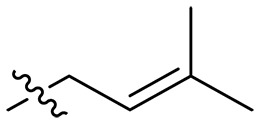	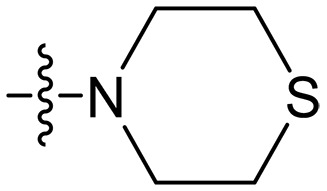	3	>50	>50	>50	>50	>200
**XT12**	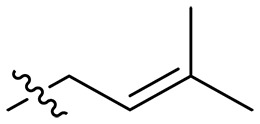	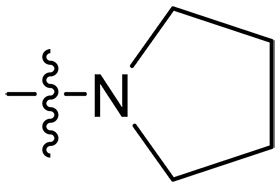	3	6.25	12.5	6.25	25	>200
**XT13**	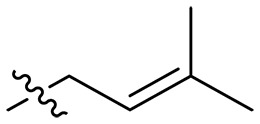	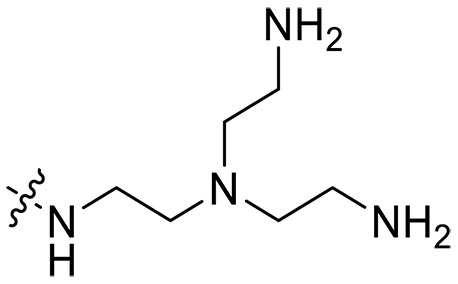	3	6.25	6.25	6.25	50	>200
**XT16**	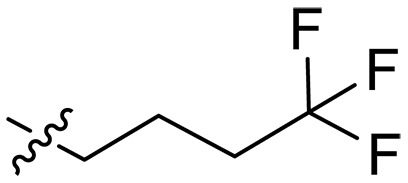	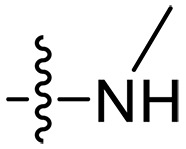	3	50	>50	>50	>100	>200
**XT17**	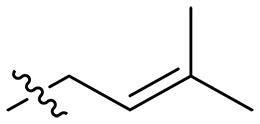	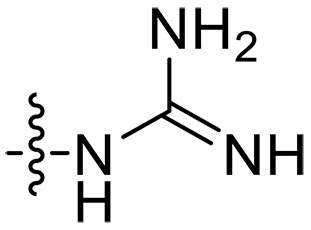	3	0.39	0.39	0.39	3.125	>200
**XT18**	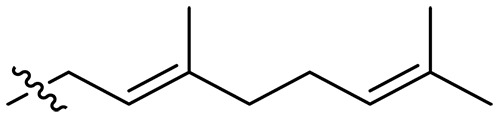	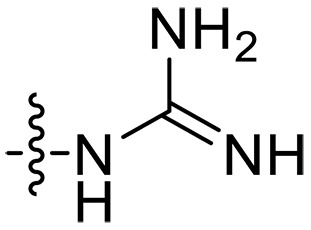	3	0.78	0.78	0.78	1.56	65.3 ± 2.75
**XT19**	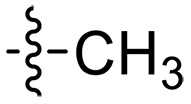	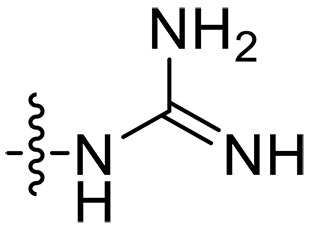	3	6.25	12.5	6.25	100	>200
**XT31**	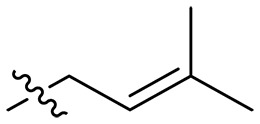	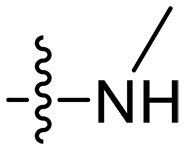	4	6.25	6.25	6.25	12.5	>200
**XT32**	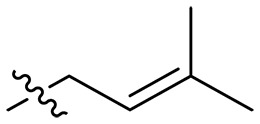	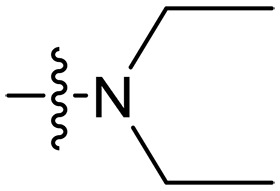	4	3.125	6.25	3.125	100	>200
**XT33**	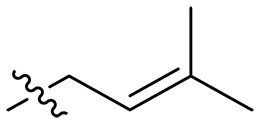	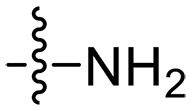	4	3.125	3.125	3.125	12.5	>200
**XT34**	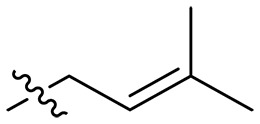	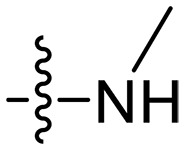	6	1.56	1.56	1.56	12.5	>200
**XT35**	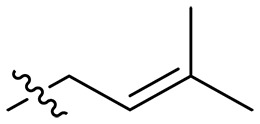	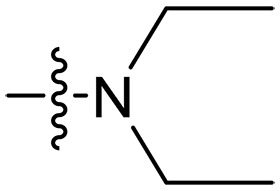	6	1.56	3.125	3.125	50	>200
**XT37**	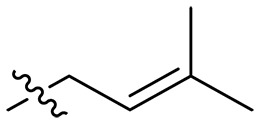	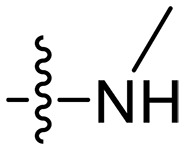	8	0.78	0.39	0.39	6.25	>200
**XT38**	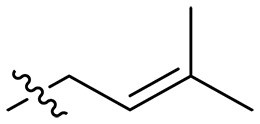	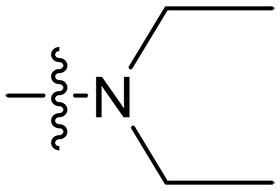	8	0.39	0.78	1.56	>50	>200
**XT40**	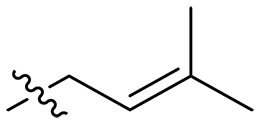	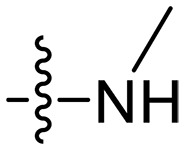	12	25	12.5	6.25	>100	>200
**XT41**	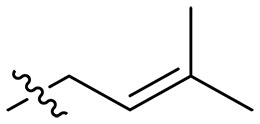	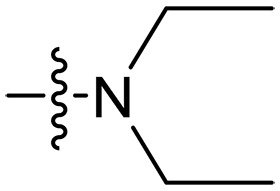	12	6.25	3.125	3.125	>100	>200
**XT42**	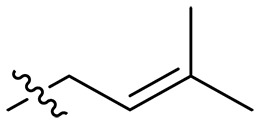	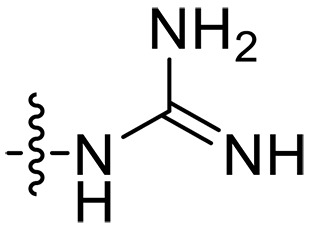	2	1.56	1.56	1.56	12.5	>200
**XT43**	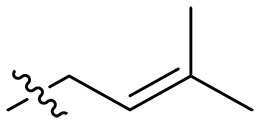	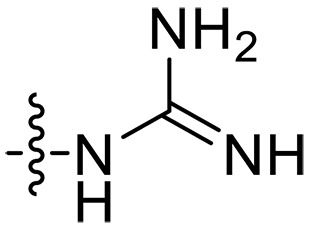	4	0.195	0.195	0.195	1.56	>200
**XT44**	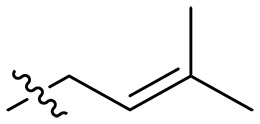	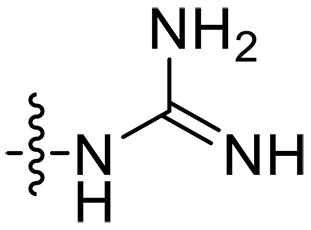	6	0.098	0.098	0.098	1.56	>200
**XT45**	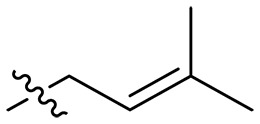	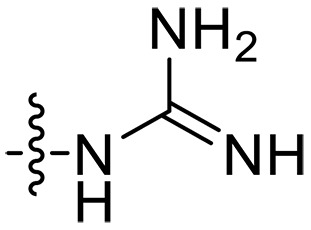	8	0.39	0.195	0.39	6.25	>200
**VAN**	–	–	–	0.78	1.56	1.56	>50	>200
**AMO**	–	–	–	ND	ND	ND	6.25	>200

*The MIC is the lowest concentration that completely inhibits microbial growth, > means no activity at the tested concentration.

†HC_50_ is the concentration causing 50% hemolysis of red blood cells, > means no activity at the concentration mentioned.

**Table 2.** MIC of xanthone derivatives against five additional Gram-negative bacterial strains. Cmpd, compound; CPF, ciprofloxacin.

Cmpd	MIC_90_ (μg/ml)^*^				
	*P. aeruginosa* ATCC 9027	*A. baumannii* ATCC 17978	*A. baumannii* R2889	*K. pneumonia* ATCC 10031	*K. pneumonia* ATCC 14581
**XT01**	>50	>50	>50	>50	>50
**XT17**	3.125	25	50	6.25	12.5
**XT18**	12.5	25	12.5	6.25	12.5
**XT42**	6.25	>50	>50	50	50
**XT43**	12.5	12.5	25	6.25	12.5
**XT44**	25	6.25	12.5	0.78	0.78
**XT45**	>50	50	50	12.5	25
**CPF**	0.195	0.39	0.78	0.098	0.098

*The MIC is the lowest concentration that completely inhibits microbial growth, > means no activity at the tested concentration.

### Effect of alkyl substitutions

To study the effects of varied alkyl moieties (R′ = isoprenyl group, geranyl group, methyl group, and trifluorobutyl group) on the biological activities of xanthone derivatives, compounds **XT17-19** containing identical cationic groups were synthesized. Initially, the spacer length between the xanthone scaffold and the cationic groups was fixed, and different alkyl moieties were attached. Compound **XT17**, which contained an isoprenyl group, demonstrated strong antibacterial activity against Gram-positive bacteria (MICs = 0.39 μg/ml) and good antibacterial activity against Gram-negative bacteria (MICs = 3.125 μg/ml), meanwhile exhibiting very weak hemolytic activity (HC_50_ > 200 μg/ml). Compound **XT18** (R′ = geranyl group) containing increased alkyl chains retained excellent antibacterial activity (MICs = 0.39 μg/ml), particularly against Gram-negative bacteria (MICs = 1.56 μg/ml), however exhibited moderate hemolytic activity (HC_50_ = 65.3±2.75 μg/ml), indicating that the increase in alkyl chains length contributed to both the increase of antibacterial and hemolytic activities of the xanthone derivatives. Compound **XT19** (R′ = methyl group) exhibited moderate antibacterial activity against Gram-positive bacteria (MICs = 6.25 to 12.5 μg/ml) and no activity against Gram-negative bacteria (MICs = 100 μg/ml), along with notably weak hemolytic activity (HC_50_ > 200 μg/ml), suggesting that a further reduction in alkyl chain length is detrimental to the enhancement of antimicrobial activities of xanthone derivatives. Compound **XT16** with a trifluorobutyl group exhibited almost no antibacterial activity (MICs = 50 to 100 μg/ml). The aforementioned results indicated that the isoprenyl group was the most suitable alkyl chain, and increasing or decreasing the length of the alkyl chain would lead to a reduction in antibacterial activity and membrane selectivity. Therefore, the isoprenyl group was chosen as the optimal hydrophobic substitution for subsequent research.

### Effect of spacer length

**Fig. 2.**
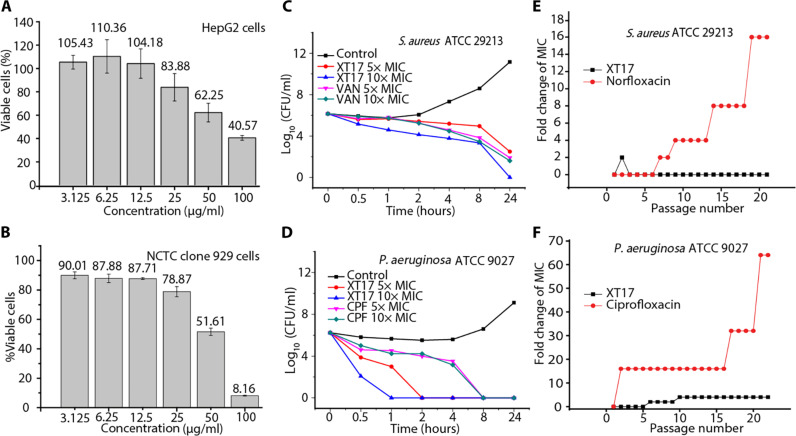
Biological profile assessments of compound XT17. (**A** and **B**) In vitro cytotoxicity of compound **XT17** toward two mammalian cells HepG2 (A) and mouse fibroblasts NCTC clone 929 cells (B) were determined by a CCK-8 assay. The data are mean ± SD from triplicate experiments. (**C**) Time-kill activities of compound **XT17** and vancomycin (VAN) against *S. aureus* ATCC 29213. (**D**) Time-kill curves of compound **XT17** and ciprofloxacin (CPF) against *P. aeruginosa* ATCC 9027. (**E**) Resistance ability studies of compound **XT17** and norfloxacin against *S. aureus* ATCC 29213. (**F**) Resistance ability studies of compound **XT17** and CPF against *P. aeruginosa* ATCC 9027. All experiments were performed in duplicates.

To investigate the influence of spacer lengths connecting cationic groups to the scaffold on the biological activity of xanthone derivatives, several compounds which had the same cationic groups including a guanidine, methylamine, or diethylamine group were prepared. Among these compounds coupled with guanidine moieties, compounds **XT42** (spacer length *n* = 2), **XT17** (*n* = 3), **XT43** (*n* = 4), **XT44** (*n* = 6), and **XT45** (*n* = 8) exhibited excellent antibacterial activity against three Gram-positive bacteria strains (MICs = 0.098 to 1.56 μg/ml) and good antibacterial activity against Gram-negative bacteria *E. coli* ATCC 25922 (MICs = 1.56 to 12.5 μg/ml) and displayed very weak hemolytic activity (HC_50_ > 200 μg/ml). No antibacterial activity against *E. coli* ATCC 25922 (MICs > 100 μg/ml) and notably reduced antibacterial activity against *S. aureus* ATCC 29213 (MICs = 25 μg/ml) were observed for compounds **XT40** and **XT41** with the longest spacer length (*n* = 12). An increasing trend in antibacterial activity was found as the spacer length (*n*) increased from 2 to 3, further to 4, and ultimately to 6. Conversely, a decreasing trend in antibacterial activity was noted as the spacer length increased from 6 to 8 and further to 12. Similar results were also acquired in compounds with different spacer lengths but the same methylamine group as the cationic group (including **XT08**, **XT31**, **XT34**, and **XT37**) or the diethylamine group as the cationic group (including **XT09**, **XT32**, **XT35**, and **XT38**). Therefore, an appropriate spacer length (*n* = 3, 4, or 6) was conducive to enhancing antibacterial activity of xanthone derivatives. Next, compounds **XT17** (*n* = 3), **XT43** (*n* = 4), and **XT44** (*n* = 6), which exhibited excellent antibacterial activity, were selected for in vitro cytotoxicity studies toward mouse fibroblasts using Cell Counting Kit-8 (CCK-8) assay. When treated with these three compounds at 25 μg/ml, the cell survival percentages of mouse fibroblasts NCTC clone 929 cells were maintained 78.87 ± 3.46%, 40.31 ± 1.33%, and 4.87 ± 0.40%, respectively (Fig. 2B and fig. S1). This result demonstrated that compounds with a spacer length (*n*) of 3 had very low toxicity to mammalian cells. Thus, a spacer length (*n*) of 3 was chosen as the optimal spacer length in the subsequent structural optimization study.

### Effect of varied amine substitutions

Compounds **XT08-13**, which had the same lipid chain of isoprenyl group and the same spacer length of *n* = 3, were synthesized to investigate the effects of the varied cationic substituents on the biological activities of xanthone derivatives. Both the secondary amine-substituted derivative **XT08** (methylamine-coupled) and the tertiary amine-substituted derivative **XT09** (*N*,*N*-diethylamine-coupled) exhibited moderate antibacterial activities against all tested strains (MICs = 6.25-50 μg/ml). Replacements using *N*,*N*-dibutylamine (**XT10**) instead of *N*,*N*-diethylamine (**XT09**) resulted in a roughly twofold decrease in antibacterial activity, indicating that excessive hydrophobicity reduced antibacterial activity. Compound **XT12** coupled with the cyclic amine pyrrolidine also displayed moderate antibacterial activity against the tested strains, with MIC values of 6.25 to 25 μg/ml. Compound **XT13** was incorporated with multiple nitrogen amines, but its antibacterial activity was not notably enhanced, with MICs of 6.25 to 50 μg/ml. The results suggested that cationic substituents including secondary amines, tertiary amines, long-chain tertiary amines, cyclic amines, and multiple nitrogen amines did not notably contribute to enhancing the antibacterial activity of xanthone derivatives.

### Effect of p*K*_a_ values of amine

To investigate the impact of the p*K*_a_ values of amine groups on the biological activities of xanthone derivatives, compounds **XT07**, **XT11**, and **XT17** were synthesized. Thiomorpholine-coupled **XT11** (p*K*_a_ = 9.0 for the free thiomorpholine group) (*45*) with a low p*K*_a_ value of the cationic group displayed almost no antibacterial activity (MICs > 50 μg/ml) and very weak hemolytic activity (HC_50_ > 200 μg/ml). **XT07** containing a primary amine (p*K*_a_ = 10.54 for the free primary amine group) (*46*) exhibited moderate antibacterial activity (MICs = 12.5 to 50 μg/ml) against both Gram-positive and Gram-negative bacteria, along with very poor hemolytic activity (HC_50_ > 200 μg/ml). Upon converting the primary amine to a guanidine group (p*K*_a_ = 12.48 for the free guanidine group) (*46*), a roughly 8- to 32-fold increase in antibacterial activity was observed for compound **XT17** with MICs of 0.39 to 3.125 μg/ml. The results suggested that cationic groups with higher p*K*_a_ values contributed to enhancing the antibacterial activity of xanthone derivatives.

Compounds **XT17-18** and **XT42-45** exhibited excellent antibacterial activity (MICs = 1.56 to 12.5 μg/ml) against Gram-negative bacteria *E. coli* ATCC 25922 (Table 1), consequently, in vitro antibacterial activities of these compounds were further measured against five additional Gram-negative bacteria including *P. aeruginosa* ATCC 9027, *A. baumannii* ATCC 17978, *A. baumannii* R2889, *K. pneumoniae* ATCC 10031, and *K. pneumoniae* ATCC 14581. As shown in Table 2, compound **XT44** exhibited excellent antibacterial activity against *K. pneumoniae* (MICs = 0.78 μg/ml) however moderate activity against *P. aeruginosa* (MICs = 25 μg/ml). Conversely, compound **XT17** demonstrated good antibacterial activity against *P. aeruginosa* (MICs = 3.125 μg/ml) and moderate activity against *K. pneumoniae* (MICs = 6.25 to 12.5 μg/ml). Both compounds displayed weak antibacterial activity against *A. baumannii* (MICs = 6.25 to 50 μg/ml). The specific reasons for this activity remain unclear. Among Gram-negative bacteria, infections caused by *P*. *aeruginosa* are particularly challenging as this organism is inherently resistant to many antibiotic categories (*47*, *48*). The infectious diseases society of America has included *P*. *aeruginosa* in the list of “ESKAPE” pathogens which represent the greatest threat to human health (*49*). Consequently, there is an urgent need to develop innovative molecules for pathogen infections caused by *P. aeruginosa*. Among the synthesized compounds, compound **XT17** exhibited great potential in treating infections caused by *P. aeruginosa*. In addition, compound **XT17** exhibited potent broad-spectrum antibacterial activity against both the three tested Gram-positive bacteria (MICs = 0.39 μg/ml) and Gram-negative bacteria (MIC = 3.125 to 12.5 μg/ml), very weak hemolytic activity (HC_50_ > 200 μg/ml), and low in vitro cytotoxicity [concentration of cytotoxicity 50% (CC_50_) > 50 µg/ml]. Therefore, compound **XT17** was selected as a candidate antimicrobial agent for further investigation.

### In vitro biological properties of compound XT17

Cytotoxicity toward mammalian cells is one of the main indicators for evaluating antibacterial agents. The cytotoxicity of compound **XT17** was determined against human hepatoma cells (HepG2) and mouse fibroblasts NCTC clone 929 cells by using CCK-8 assay (Fig. 2, A and B). When treated with compound **XT17** at 50 μg/ml, neither HepG2 nor mouse fibroblasts NCTC clone 929 cells showed obvious cytotoxicity, with 62.25 ± 8.01% and 51.61 ± 2.46% viability, respectively. The above results indicated that compound **XT17** had very low cytotoxicity toward mammalian cells.

The bactericidal growth properties of compound **XT17** against Gram-positive and Gram-negative bacteria were investigated using time-kill kinetic assays. Notably, as illustrated in Fig. 2C, for *S. aureus* ATCC 29213, compound **XT17** attained 5.16 log decrease in bacterial load in 24 hours at a concentration of 10× MIC, while the traditional vancomycin achieved a 4.56 log reduction of bacteria in 24 hours at 10× MIC, indicating that the bacterial killing kinetics of compound **XT17** is better than that of vancomycin. As shown in Fig. 2D, compound **XT17** posed 3.24 log bacterial load reduction in 1 hour at a concentration of 5× MIC, and no bacterial regrowth was observed at 24 hours, suggesting that compound **XT17** killed *P. aeruginosa* ATCC 9027 rapidly. On the contrary, ciprofloxacin (CPF) achieved a 1.71 log bacterial load reduction in 1 hour at a concentration of 5× MIC. These results indicated that compound **XT17** exhibited a rapid bactericidal property against Gram-negative bacteria *P. aeruginosa* ATCC 9027.

Overcoming bacterial resistance is one of the key factors in the development of antimicrobial agents. The drug resistance study of compound **XT17** against *S. aureus* ATCC 29213 and *P. aeruginosa* ATCC 9027 was performed. During the 20-day passages, no more than fourfold increase in MIC values of compound **XT17** was observed, indicating that a very low development tendency of bacterial resistance was found when bacteria was exposed to the continuous use of compound **XT17**. Contrary, when the same two strains of bacteria were treated with norfloxacin (for *S. aureus*) and CPF (for *P. aeruginosa*), the MIC values were enhanced by approximately 16 and 64 times folds, respectively (Fig. 2, E and F). These findings clearly implied that long-term use of the two commercial drugs led to bacterial resistance; however, compound **XT17** showed a very low frequency of developing bacterial resistance.

### Membrane binding and permeation ability of compound XT17

Cytoplasmic membrane permeabilization of compound **XT17** against *S. aureus* ATCC 29213 and *E. coli* ATCC 25922 was measured using the SYTOX Green uptake assay. SYTOX Green dye, a high-affinity nucleic acid probe, is able to penetrate damaged bacterial cell membranes and combine with intracellular nucleic acids, resulting in a notably enhancement of fluorescence (*50*). Following treatment of the bacterial cells with compound **XT17**, the fluorescence intensity of the mixture did not show a notable increase, even when tested with a high concentration of compound **XT17** at 8× MIC, as depicted in fig. S2 (A and B). These results demonstrated that compound **XT17** could not disrupt the cell membrane integrity of either Gram-positive or Gram-negative bacteria.

The fluorescence probe DiSC_3_(5) was used to investigate the membrane depolarization of compound **XT17** against *S. aureus* ATCC 29213 and *E. coli* ATCC 25922. DiSC3(5), a membrane potential-sensitive probe, exhibits a sharp increase in fluorescence intensity when the membrane potential is changed (*51*). There were no changes in fluorescence intensity in compound **XT17**–treated groups, implying that this compound might not be able to depolarize the bacterial cell membranes (fig. S2, C and D).

**Fig. 3.**
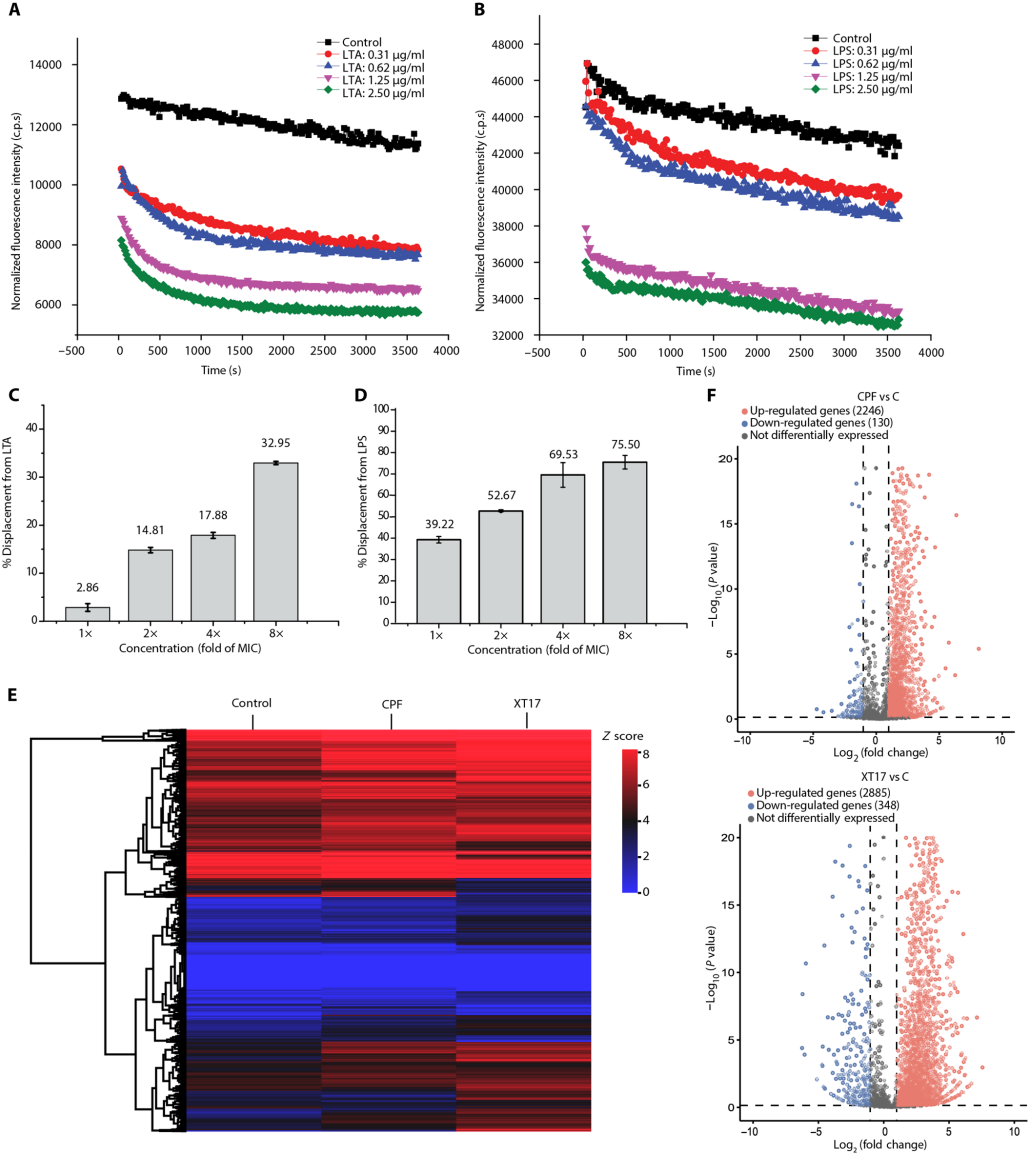
Antibacterial mechanism study of compound XT17. (**A** and **B**) Interaction between compound **XT17** (1× MIC) with different concentrations (final concentrations of 0, 0.31, 0.62, 1.25, and 2.50 μg/ml) of LTA (A) or LPS (B) was evaluated using the change in the fluorescence intensity of compound **XT17**; DMSO served as a negative control. c.p.s, counts per second. (**C** and **D**) The percentage of BODIPY-TR-cadaverine displacement from LTA (C) or LPS (D) by compound **XT17**. The data are mean ± SD from triplicate experiments. (**E**) Differential expression of *E. coli* ATCC 25922 exposed to antimicrobial agents. Heatmap depicted the *z* scores for each gene corresponding to CPF- and **XT17**-treated and untreated samples. Scale is shown on the left. (**F**) Volcano plots showed the differentially expressed genes (DEGs) for *E. coli* treated with CPF and **XT17**, each in comparison to the untreated control. The genes are colored if they pass the thresholds for −log_10_ *P* value (*P* < 0.05) and log_2_fold change greater than 1, red if they are up-regulated and blue if they are down-regulated.

We evaluated the binding ability of compound **XT17** to negatively charged LTA or LPS by measuring the change in fluorescence intensity of compound **XT17**. LTA is a major component of the cell wall of Gram-positive bacteria, whereas LPS is a major component of the OM of Gram-negative bacteria (*36*). When LTA or LPS was bound to compound **XT17**, the intensity of the fluorescence decreased notably. After LTA or LPS (final concentrations of 0, 0.31, 0.62, 1.25, and 2.5 μg/ml, respectively) was treated with compound **XT17** (1× MIC), the changes of fluorescence intensity were monitored. As shown in Fig. 3 (A and B), fluorescence emission of compound **XT17** was decreased in a concentration-dependent manner upon binding to either LTA or LPS. These findings revealed that compound **XT17** had strong binding effects with both LPS and LTA.

The interaction between compound **XT17** and LTA or LPS on the bacterial surface was further investigated through BODIPY-TR-cadaverine displacement assay. When the probe was bonded to LTA or LPS, quenching of fluorescence intensity was observed, whereas a marked fluorescence enhancement was observed when the probe was displaced by other molecules to interact with LTA or LPS and redissolved in solution (*52*, *53*). As depicted in Fig. 3 (C and D), compound **XT17** at a concentration of 2× MIC was able to displace 14.81% of BODIPY-TR-cadaverine from LTA-BODIPY or 52.67% from LPS-BODIPY. When the concentration of compound **XT17** reached 8× MIC, the percentages of displacement increased to 32.95% (LTA) and 75.50% (LPS), respectively. These results indicated a strong binding ability between compound **XT17** and LTA or LPS, exhibiting a concentration-dependent manner.

### Transcriptional response of compound XT17 against *E. coli* ATCC 25922

Treatment with compound **XT17** at 4× MIC for 2 hours was chosen to ensure a sufficient yield and quality of RNA from viable cells. To better understand the molecular mechanisms underlying **XT17**-mediated antibacterial activities against Gram-negative bacteria, we explored the transcriptome changes on compound **XT17**–treated *E. coli* ATCC 25922 via RNA-seq technology. Approximately 17 M reads were obtained from each sample. After filtering by quality, all the reads from three different groups were mapped to *E. coli* genome. The summary of transcriptome sequence data is shown in table S1.

The heatmap was constructed showing *z* score values for differentially expressed genes (DEGs) of untreated, compound **XT17**–, and CPF-treated groups. According to the RNA-seq results, a total of 4617 genes were expressed in the compound **XT17**–treated group, 4682 genes in the CPF-treated group, and 4552 genes in the untreated group. We used two criteria to assess the significance of differences in gene expression: *P* value less than 0.05 and absolute log_2_ (fold change) greater than 1 (Fig. 3E). The volcano plots of the DEGs demonstrated that 2376 and 3233 genes in *E. coli* were notably differentially expressed after CPF and compound **XT17** treatments, respectively (Fig. 3F). In response to CPF treatment, 2246 genes were up-regulated and 130 were down-regulated. In response to compound **XT17** treatment, we discovered that 2885 genes were up-regulated and 348 genes were down-regulated. CPF and **XT17** treatments shared a total of 1859 DEGs, indicating that both agents may exert comparable mechanisms (fig. S3). The shared most notable alterations of genes in *E. coli* treated with CPF and **XT17** are listed in table S2.

### GO, KEGG enrichment, and COG analyses

**Fig. 4.**
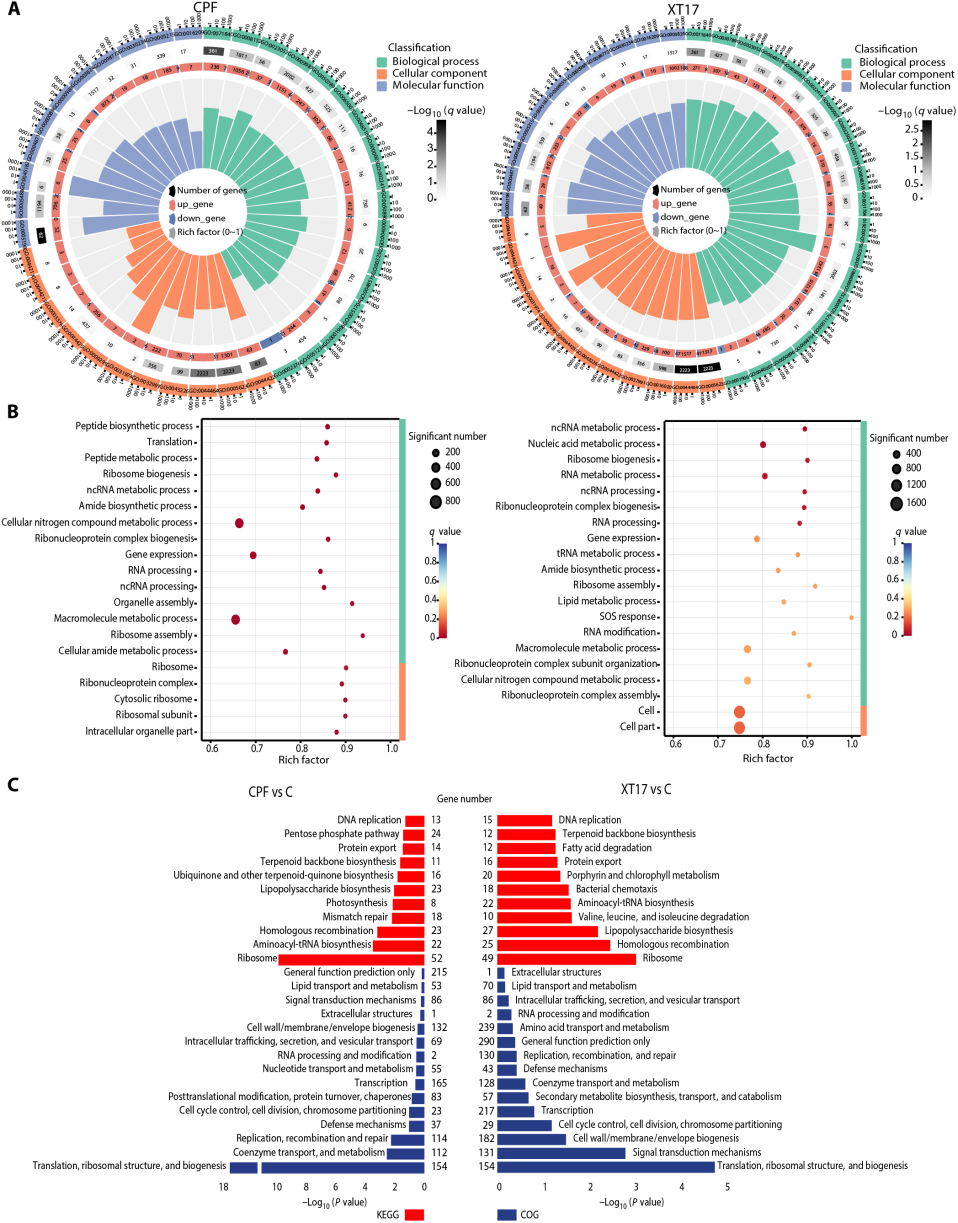
Functional analysis of treated *E. coli* ATCC 25922 with CPF and compound XT17 based on GO, KEGG, and COG. (**A**) Functional classification of biological processes, molecular functions, and cellular components identified for the notable DEGs between the treated and untreated samples. (**B**) Bubble map presented the top 20 terms in the GO enrichment analysis. Circle size indicates the notable number of genes enriched in each term. Color saturation represents the significance level. The *q* value of each term is depicted by the color scale. (**C**) KEGG pathways and COG categories enriched in the target genes of the CPF- and compound **XT17**–enriched mRNAs. ncRNA, noncoding RNA.

On the basis of the comprehensive high-throughput transcriptome analysis, compound **XT17** was found to be complex that targets multiple metabolic pathways. According to Gene Ontology (GO) annotation, the primary targets of compound **XT17** and CPF were most scattered among biological processes, including metabolic and cellular processes. Meanwhile, the predominant molecular functions were catalytic activity, binding, and transporter activity (Fig. 4, A and B). In addition, in the Kyoto Encyclopedia of Genes and Genomes (KEGG) enrichment analysis, we observed that the treatment by compound **XT17** and CPF led to up-regulation of numerous genes encoding large ribosomal subunits, which were enriched in the ribosome pathway (Fig. 4C). The mRNA levels of *rpsQ* (30*S* ribosomal protein S17) under the influence of antibacterial compounds were increased up to sixfold after 2 hours of exposure (fig. S4, left). Both compound **XT17** and CPF also up-regulated the genes involved in homologous recombination, mismatch repair, and LPS biosynthesis, which are believed to be the targets of these agents (table S3 and fig. S4, right). Conversely, the CPF treatment led to down-regulation of genes differed from compound **XT17** treatment. Furthermore, the Clusters of Orthologous Genes (COG) annotation analysis of DEGs was also analyzed, and the results revealed that the genes mainly involved in translation, ribosomal structure, and biogenesis (Fig. 4C). From the interaction network constructed based on COG, we discovered a particularly strong relationship between the majority of the genes and compound **XT17**. These genes were probably connected to signal transduction mechanisms, transcription, and replication, recombination, and repair (fig. S5).

### Validation of qPCR

**Fig. 5.**
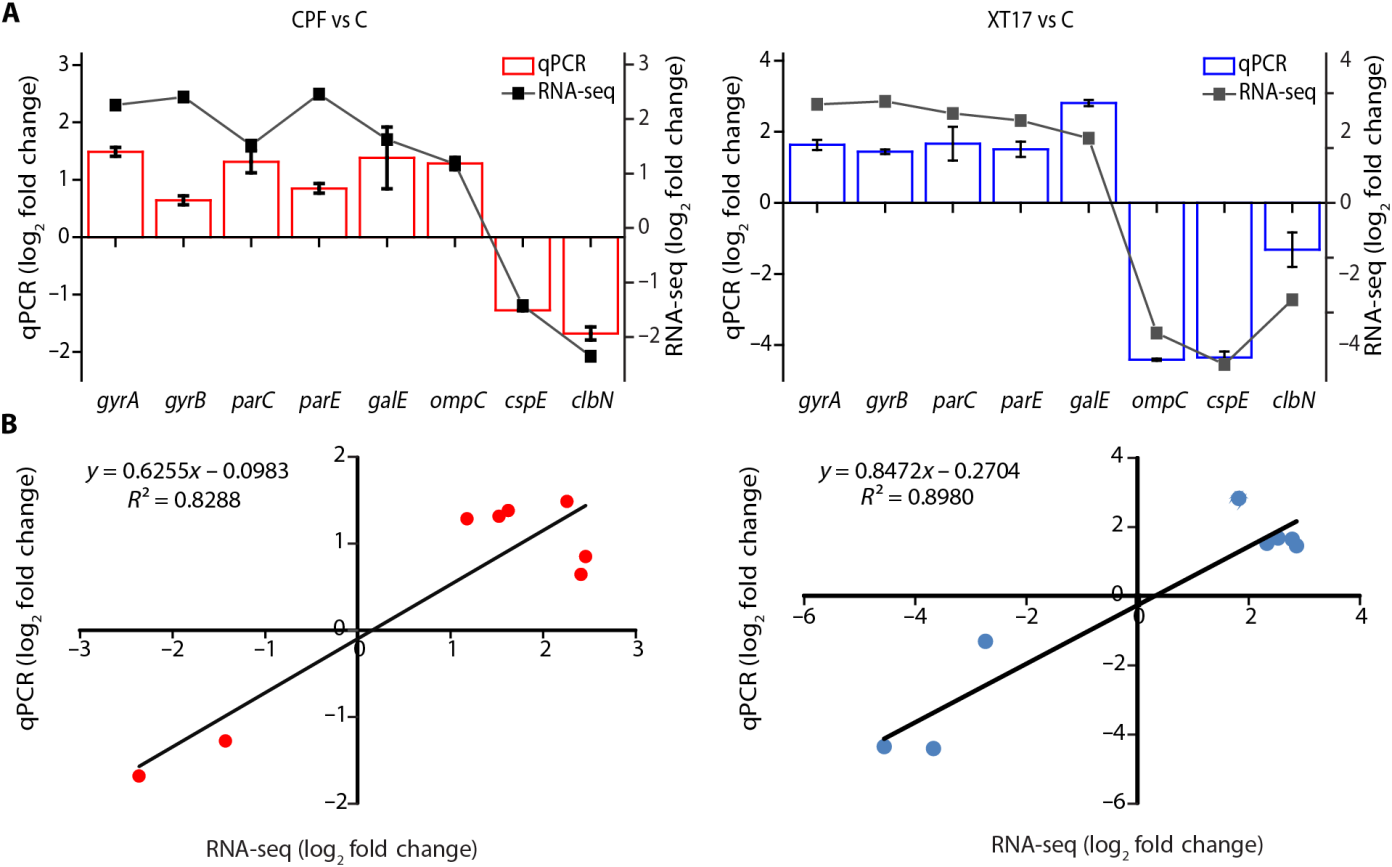
Validation for the representative DEGs of RNA-seq datasets via qPCR assay. (**A**) The relative transcript levels were normalized with *GAPDH* as a housekeeping gene. The error bars indicate the SD of the mean of three independent replicates. (**B**) Correlation between RNA-seq and qPCR results for verification of selected DEGs. The gene expression fold change of both RNA-seq and qPCR for the DEGs were log transformed into base 2, and the RNA-seq log_2_ (fold change) was plotted against the qPCR log_2_ (fold change).

A set of eight DEGs, including up- and down-regulation types, were selected randomly for the quantitative real-time quantitative polymerase chain reaction (qPCR) assay to verify the expression patterns of RNA-seq. We compared the transcript profiles obtained from qPCR with those generated from RNA-seq analysis of the CPF- and compound **XT17**–treated cells. The obtained data confirmed that the expression patterns of all DEGs produced by qPCR were consistent with the RNA-seq data, and both methods yielded the same expression trends. A high degree of correlation in the expressional profile between qPCR and RNA-seq was attained with *R*^2^ values of 0.8288 and 0.8980, for both CPF- and compound **XT17**–treated cells, respectively (Fig. 5). These findings proved the validity of the RNA-seq analysis and may serve as a foundation for future research on the roles played by the genes.

### Compound XT17 targets DNA gyrase and topoisomerase IV

**Fig. 6.**
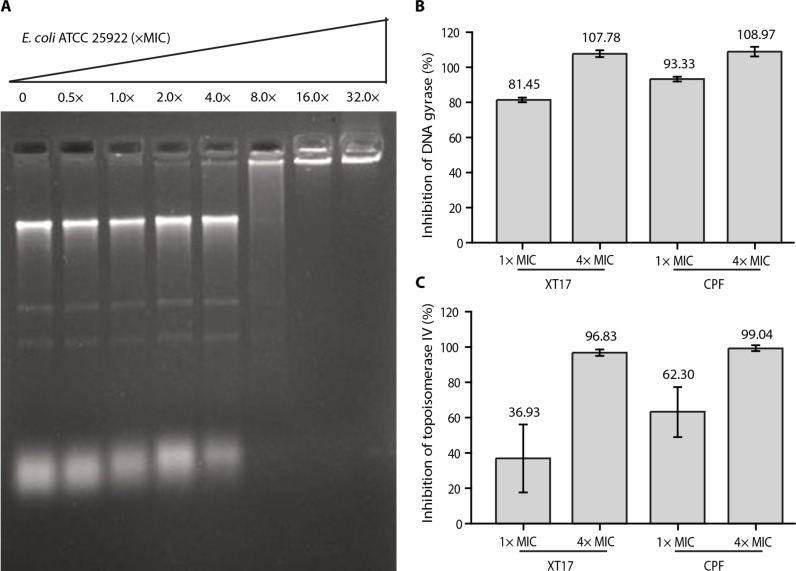
Inhibition of *E. coli* DNA gyrase and topoisomerase IV. (**A**) Interaction of compound **XT17** and DNA at the indicated concentrations (0 to 32× MIC). DNA product was separated by agarose gel electrophoresis. (**B**) In vitro inhibition percentage of DNA gyrase from *E. coli* after incubation with compound **XT17** and CPF. (**C**) Inhibition percentage of topoisomerase IV at different concentrations of compound **XT17** and CPF. The data are mean ± SD from triplicate experiments.

For the electrophoretic mobility shift assay, compound **XT17** at 8× MIC was able to directly interact with the *E. coli* DNA (Fig. 6A). Besides, several studies have reported that CPF affect DNA replication by inhibiting DNA gyrase (topoisomerases II and IV) (*54*–*56*). The compound **XT17** was tested for in vitro inhibition against two enzymes, DNA gyrase, and topoisomerase IV from *E. coli*, to explore its mechanism of action. For the purpose of comparison, CPF was selected as the standard reference for both enzymes and tested concurrently. Compounds **XT17** exhibited high inhibition against *E. coli* DNA gyrase. The degree of inhibition by compound **XT17** (81.45 ± 1.33%) was remarkably similar to that of CPF (93.33 ± 1.36%) at 1× MIC. More than 100% inhibition of DNA gyrase was induced by compound **XT17** (Fig. 6B), which is comparable to the inhibitory effect of CPF at high concentration (4× MIC). In contrast, compound **XT17** only showed a notable inhibition of topoisomerase IV activity at high concentration (4× MIC), devoting that compound **XT17** was mainly selective for *E. coli* gyrase (Fig. 6C).

### Molecular docking study

**Fig. 7.**
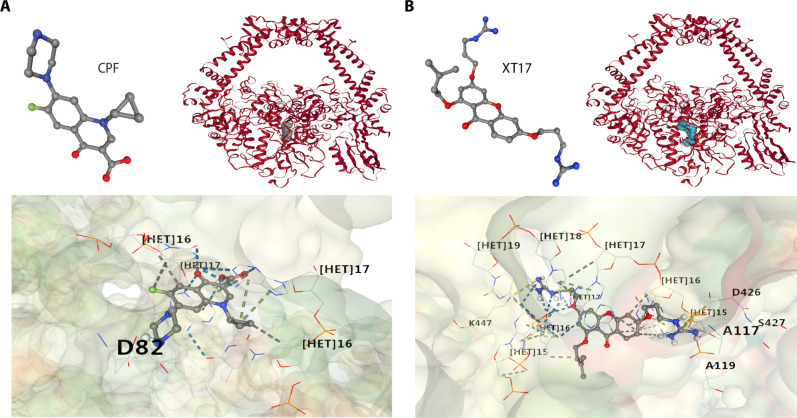
Comparing binding of XT17 with the known DNA gyrase inhibitor ciprofloxacin (CPF) in silico. The best molecular docking model of CPF (**A**) and compound XT17 (**B**) with cryo–electron microscopy structure of DNA gyrase from *E. coli* (PDB code: 6RKV). Hydrogen bonds between compounds and amino acids are represented by blue dashed lines, while hydrophobic interactions are represented by gray dashed lines, and ionic interactions are represented by yellow dashed lines. Ribbon model shows the binding pocket structure of DNA gyrase in the presence of CPF or compound **XT17**. Data are collected from the top ranked interactive conformations predicted by CB-DOCK2.

The binding interactions of CPF and compound **XT17** with the pocket of DNA gyrase from *E. coli* [Protein Data Bank (PDB) code: 6RKV] (*57*) were examined through a molecular docking study. The synthesized compound **XT17** formed strong bonds with one or more amino acids in the active pocket of the enzyme (Fig. 7). The compounds **XT17**_1 to **XT17**_4 displayed minimum binding energies ranging from −7.0 to −8.2 kcal/mol, which showed comparable binding scores and amino acid interactions compared to CPF_1 to CPF_4 (table S4). In addition, a molecular docking study for CPF and compound **XT17** against DNA gyrase from *S. aureus* (PDB code: 2XCT) (*58*) was performed. Molecular modeling of the cocrystallized ligand (CPF) revealed different types of interactions with the active site of gyrase enzyme including a hydrogen bonding and hydrophobic interactions with DNA nucleotide bases (fig. S6A). Also, docking studies of **XT17** revealed that they have the ability to interact with crystal structure of gyrase enzyme via hydrophobic interactions, hydrogen bonding interactions, and ionic interactions with amino acid residues and DNA nucleotide bases (fig. S6B). The docked compound **XT17** displayed minimum binding energy to gyrase enzyme with the scores value of −8.3 kcal/mol, which had comparatively binding energy to the cocrystallized ligand CPF (−8.5 kcal/mol) (table S5). In silico molecular docking studies revealed that compound **XT17** had close binding energy to the standard drug and might be considered as a good DNA gyrase inhibitor. The binding affinity and bonding interactions of the docked complexes are summarized in tables S4 and S5 and figs. S6 and S7.

### In vivo acute toxicity and pharmacokinetic studies

**Fig. 8.**
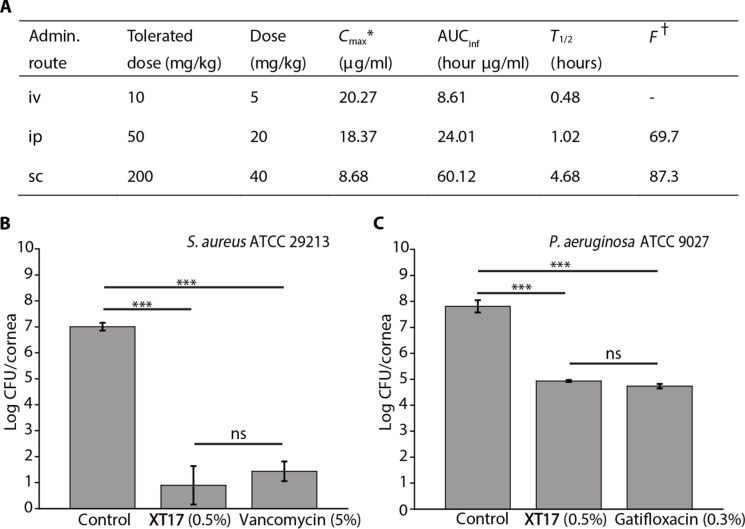
PK study of XT17 in mice and efficacy of XT17 (5 mg/ml) in a murine corneal infection model. (**A**) PK parameters of **XT17** administered via intravenous (iv), intraperitoneal (ip), and subcutaneous (sc) routes in mice (*n* = 3). ^*^For bolus iv, *C*_max_ = *C*_0_ (concentration extrapolated to zero time, *T*_max_ ≈ 0); ^†^Bioavailability, *F* = AUC_ip (or sc)_/AUC_iv_ at the same dose. AUC, area under the curve. (**B**) A mouse corneal infection model caused by *S. aureus* ATCC 29213. Vancomycin (50 mg/ml) and 5% glucose solution were served as positive and negative controls, respectively. (**C**) A mouse corneal infection model caused by *P. aeruginosa* ATCC 9027. Gatifloxacin (3 mg/ml) and 5% glucose solution were served as positive and negative controls, respectively. *P* values are determined using one-way analysis of variance (ANOVA; ***P < 0.001 compared to the model control; ns, not significant).

Initial tolerability studies in mice revealed that compound **XT17** was well tolerated at doses up to 200 mg/kg administered subcutaneously (sc), 50 mg/kg administered intraperitoneally (ip), and 10 mg/kg administered intravenously (iv) (Fig. 8A). The pharmacokinetic (PK) parameters of **XT17** were evaluated in CD-1 mice, administered via intravenous, intraperitoneal, and subcutaneous at the doses of 5, 20, and 40 mg/kg, respectively. The results were summarized in Fig. 8A, and complete details of various routes of administration were displayed in tables S6 to S8. PK analysis indicated that **XT17** had the clearance half-life (*T*_1/2_) of 0.48, 1.02, and 4.68 hours when administered at the doses of 5 mg/kg iv, 20 mg/kg ip, and 40 mg/kg sc, respectively. The maximum plasma concentrations (*C*_max_) of **XT17** were 18.37 and 8.68 μg/ml for the 20 mg/kg ip dose and 40 mg/kg sc dose, respectively, exceeding its MIC values, suggesting potentially notable in vivo efficacy. Compound **XT17** exhibited high bioavailability (*F*) with subcutaneous injection (87.3%) and intraperitoneal injection (69.7%), indicating that **XT17** can be rapidly and largely absorbed in vivo. Blood concentrations of **XT17** were maintained above MICs for more than 8 hours when administered subcutaneously at a single dose of 40 mg/kg and more than 4 hours when administered intravenously at a single dose of 5 mg/kg, indicating its good exposure in mouse plasma (fig. S8).

### In vivo antibacterial efficacy

To assess the in vivo therapeutic performance, compound **XT17** was tested in a murine corneal infection model against *S. aureus* ATCC 29213 or *P. aeruginosa* ATCC 9027. In the *S. aureus* ATCC 29213–induced corneal infection model, the mice were immunosuppressed by intraperitoneal injections of cyclophosphamide (100 mg/kg) three times 5 days before infection. One day after infection, each group of mice (*n* = 5) was topically treated with 0.5% compound **XT17**, 5% vancomycin (positive control), or 5% glucose solution (negative control) four times daily for 3 days. A notable decrease in infection was observed in the compound **XT17**–treated group (5 mg/ml) against *S. aureus* ATCC 29213 (*P* < 0.001), with reduction in colony-forming units (CFU) equivalent to that caused by 10-fold higher dosage of vancomycin (50 mg/ml). As shown in Fig. 8B, compared with the control, compound **XT17** and vancomycin produced 6.10 log (*P* < 0.001) and 5.57 log (*P* < 0.001) reductions in the number of viable bacterial colonies in the infected corneas, respectively. This result showed that the in vivo efficacy of compound **XT17** (5 mg/ml) against *S. aureus* ATCC 29213 was comparable to that of vancomycin (50 mg/ml), although its concentration was much lower than that of vancomycin. In the *P. aeruginosa* ATCC 9027–induced corneal infection model, the mice were not immunosuppressed, and 0.3% gatifloxacin was used as a positive control. In the same model, the compound **XT17**–treated group (5 mg/ml) against *P. aeruginosa* ATCC 9027 showed a similar reduction in CFU levels compared to the gatifloxacin-treated group (3 mg/ml). As shown in Fig. 8C, as compared with the control, compound **XT17** (5 mg/ml) and gatifloxacin (3 mg/ml) produced 2.88 log (*P* < 0.001) and 3.08 log (*P* < 0.001) reductions in the number of viable bacterial colonies in the infected corneas, suggesting that both of them exhibited potent efficacy.

In addition, the in vivo toxicity assessment of compound **XT17** toward murine cornea was conducted through local application using 1% fluorescein sodium solution to stain the cornea. The slit-lamp photographs indicated that no notable signs of corneal damage were observed on the **XT17**-treated murine cornea (fig. S9). In vivo efficacy studies demonstrated that compound **XT17** exhibited notable potential as an effective broad-spectrum antibacterial agent for treating infections caused by both Gram-positive and Gram-negative bacteria.

## DISCUSSION

Natural xanthone derivatives are a promising class of antibacterial compounds that could be used to combat pathogens (*13*, *25*, *59*–*61*). Emerging from previous studies performed for xanthone analogs as potential antimicrobials, we have unambiguously improved the development of these analogs by following a rational design approach, which accounts for lipophilicity, charge, and amphipathicity (*30*, *62*). Along these lines, we first synthesized the 1,3,6-triphenol hydroxyl xanthone parent nucleus and then structurally modified the three phenolic hydroxyl groups. A series of xanthone derivative compounds with strong antibacterial activity, weak hemolytic activity, and low toxicity was designed and synthesized. Total synthesis design has many advantages, such as low cost, simple synthesis, and a large scale for transformation (*63*). We successfully synthesized 45 xanthone analogs and investigated the structure-activity relationship. On the basis of the biological properties, it is noteworthy that the promising lead compound **XT17** was very active against the high priority pathogens including MRSA, *P. aeruginosa*, and *K. pneumonia.* This compound, in particular, displayed promising in vivo efficacy in both *S. aureus* ATCC 29213 and *P. aeruginosa* ATCC 9027–induced corneal infections and low mammalian toxicity.

Since compound **XT17** had the potential to be a broad-spectrum antibacterial agent, it was predominantly selected for in-depth biological assessments. The findings revealed that this compound had bactericidal properties and a low likelihood of resistance development. Good biocompatibility with mammalian cells is considered one of the critical indicators for the clinical application of antimicrobial agent (*64*). Compound **XT17** had no hemolytic activity (HC_50_ > 200 μg/ml) and low cytotoxicity against mammalian cells (CC_50_ > 50 μg/ml). According to mode of action studies, compound **XT17** showed good LTA- and LPS-binding abilities, which were most probably due to the presence of the two cationic guanidyl moieties, hydrophobic xanthone scaffold, and lipid chain of isoprenyl group. Small-molecule–based peptidomimetics of cationic antimicrobial peptides electrostatically bind to the negatively charged of LTA or the bacterial OM of LPS (*65*). These interactions cause membrane-stabilizing cations like Ca^2+^ and Mg^2+^ to be displaced, resulting in self-promoted uptake of this compound into the OM (*66*). It can be concluded that compound **XT17** would be most likely to target bacterial cell wall membranes.

We further used RNA-seq to scrutinize the transcriptomic changes induced by antimicrobial compound treatment. On the basis of the transcriptomic analysis, we deduced that compound **XT17** might pose microbial death by inhibiting bacterial DNA synthesis as well as cell wall/membrane disruption. It was especially noteworthy that this compound might inhibit the activity of DNA gyrase as CPF to introduce negative supercoils in relaxed DNA. Several studies have demonstrated that the bactericidal action of CPF was induced by a direct inhibition of bacterial DNA synthesis (*67*, *68*). This antimicrobial agent specifically targets DNA gyrase, a bacterial enzyme that regulates chromosomal supercoiling, which is required for nucleic acid synthesis (fig. S10) (*69*). In line with the transcriptomic study, we found that compound **XT17** had a high inhibitory effect (~90% inhibition) on bacterial DNA gyrase in vitro, implying that it might be capable of preventing DNA replication. In addition, molecular modeling was also used to explore the interaction between compound **XT17** and *E. coli* DNA gyrase. This compound provides an intriguing possibility, with the best docking score (−8.2), within the binding pocket to DNA gyrase. The residual interactions and the docking score of compound **XT17** were comparable to those of CPF (fig. S7). In silico molecular docking analysis coupled with in vitro inhibitory studies suggested that compound **XT17** acted as a DNA gyrase inhibitor.

From a mechanistic perspective, as the compound **XT17** might have at least two modes of action, further in-depth analysis via in vivo study was conducted to examine the therapeutic effect. In vivo therapeutic efficacy of compound **XT17** was better or comparable to those of two tested commercial antibiotics vancomycin and gatifloxacin. This compound maintained excellent antibacterial efficacy in a mouse corneal infection model. This paved a way for compound **XT17** as a lead compound to be further developed as an antimicrobial candidate to treat bacterial infections. The two mechanisms of **XT17**, cell wall disruption and nucleic acid synthesis inhibition, could work together to effectively kill both Gram-positive and Gram-negative bacteria. It was believed that this compound acted by simultaneously inhibiting multiple targets, which might increase potency against drug-resistant bacterial strains, broaden the spectrum of activity, and minimize the possibility of bacterial resistance. In comparison with commercially available drugs, compound **XT17** had a substantially lower likelihood of developing resistance, demonstrating that it was a potential antibacterial agent to kill bacteria effectively and combat multidrug bacterial resistance.

## MATERIALS AND METHODS

### Chemically synthetical study

All chemicals and solvents were purchased from commercial vendors and used as received without purification. All compounds were purified by semipreparative HPLC on Agilent Technologies 1260 Infinity system using a C_18_ column (5 μm, YMC Pack) with ultraviolet radiation detected at 254 nm and a flow rate of 15 ml/min. A mixture of phase A (H_2_O + 0.1% formic acid) and phase B (methanol + 0.1% formic acid) was used as the gradient elution. The purity of all final products was more than 95% by HPLC analysis. Nuclear magnetic resonance spectroscopy spectra (^1^H and ^13^C) were recorded by a JEOL 400-MHz spectrometer. All resonance bands were compared to tetramethylsilane, which served as an internal chemical shift reference standard, with reported chemical shifts (δ) indicated in parts per million and coupling constants (*J*) in hertz. Mass spectra were acquired using a Thermal Scientific DFS high-resolution mass spectrometer. Details on the synthesis and characterization of xanthone derivatives used in this study are provided in the Supplementary Materials.

### Antibacterial susceptibility testing

MIC was determined using the broth microdilution method in accordance with Clinical and Laboratory Standards Institute guidelines. All compounds were dissolved in dimethyl sulfoxide (DMSO; final DMSO concentration of less than 0.5%) and sterile double-distilled water and then diluted with Mueller-Hinton broth (MHB). Each compound’s stock solution was serially twofold diluted, resulting in concentrations ranging from 0.39 to 100 μg/ml, and thereafter added to sterile 96-well plates. All bacterial cell suspensions were adjusted in MHB to attain a starting inoculum of 1 × 10^6^ cells/ml. A total of 100 μl volume of the bacterial suspension was then added to each well. Following a 24-hour incubation at 37°C, the optical density at 600 nm (OD_600_) was confirmed by measuring absorbance at 600 nm with a BioTek microplate reader. The MIC was characterized by the lowest concentration of the antibiotics/compounds that completely inhibited microbial growth. All measurements were repeated three times with duplicates each time.

### Hemolysis assays

The hemolytic activity of antimicrobial agents was investigated as previously described (*70*). Briefly, the rabbit erythrocytes were centrifuged at 2500 rpm for 3 min before being washed twice with sterile phosphate-buffered saline (PBS) (pH 7.4). Subsequently, the erythrocytes were diluted with PBS to a final concentration of 8% (v/v). The DMSO-dissolved compounds were twofold serially diluted in PBS to achieve a final concentration with DMSO less than 0.5%. A total of 100-μl erythrocyte suspension was then incubated with different concentrations of compound **XT17**. Sterile PBS and 0.2% Triton X-100 were used as negative and positive controls, respectively. After 1 hour of incubation at 37°C, the tested samples were centrifuged at 2500 rpm for 5 min, and aliquots of the supernatant were added to 96-well microplates. The hemoglobin release was determined by measuring the absorbance at 576 nm using a BioTek multimode reader. The percentage of hemolytic activity was calculated using the following formula: Hemolysis rate (%) = [OD_576_ (compound) − OD_576_ (PBS)]/[OD_576_ (Triton X-100) − OD_576_ (PBS)] × 100. All measurements were repeated three times with duplicates each time.

### Cytotoxicity assays

Cytotoxicity of the compound tested was assessed by CCK-8 assays as previously described (*71*). Human hepatoma cells (HepG2) and mouse fibroblasts NCTC clone 929 cells were seeded in 96-well microplates at a density of 2 × 10^4^ cells per well and placed in a 5% CO_2_ incubator at 37°C for 24 hours. The cell medium was then replaced with fresh medium containing various concentrations of compounds (3.125 to 100 μg/ml). Following a 24-hour incubation, 10 μl of CCK-8 reagent was added to each well and incubated for 1 hour. The absorbance at 450 nm was measured by a microplate reader. All measurements were repeated three times with duplicates each time.

### Time-kill kinetic study

Two doubling concentrations of compound **XT17** (5× and 10× MIC) were tested against *S. aureus* ATCC 29213 and *P. aeruginosa* ATCC 9027. The bacterial cultures were standardized to obtain an initial density that ranged from 10^5^ to 10^6^ cells/ml. The cultures were incubated in a shaking bath at 37°C. Viable counts were determined at 0-, 0.5-, 1.0-, 2.0-, 4.0-, 8.0-, and 24-hour intervals by spotting 10-μl aliquots of serially 10-fold diluted cultures onto Mueller-Hinton agar (MHA) plates. After incubation at 37°C for 24 hours, the bactericidal activity, defined as the ability to reduce the initial inoculum by 3 log_10_ CFU/ml, was examined for each tested concentration over time. All measurements were repeated three times with duplicates each time.

### Serial passage resistance induction studies

Compound **XT17** and norfloxacin resistance selection against *S. aureus* ATCC 29213, as well as compound **XT17** and CPF resistance selection against *P. aeruginosa* ATCC 9027, was done on the basis of the progressive increase in the MIC of the strains over several passages (*72*). The overnight bacteria culture was diluted and grown to exponential phase (OD_600_ = 0.4) and then incubated with the tested compound to determine the initial MIC. The bacteria were grown in a medium containing 0.5× MIC of each compound to prepare the bacterial suspension for the next MIC determination. The MICs were then recorded after incubation at 37°C for 24 hours. Daily passages were continued until the initial MIC increased by more than fourfold. The stability of acquired resistance was investigated by MIC determination and repeated for 20 days.

### SYTOX Green uptake assays

The SYTOX Green uptake was measured to explore the inner membrane permeability. The overnight bacteria culture was centrifuged and resuspended in sterile PBS to obtain an OD_600_ of 0.2. SYTOX Green dye was added to a final concentration of 0.3 μM and incubated for 20 min without being exposed to the light. Fluorescence was measured at excitation and emission wavelengths of 504 and 523 nm, respectively. After the fluorescence signal of SYTOX Green–treated suspension was stabilized, different concentrations of compounds (1×, 2×, 4×, and 8× MIC) were immediately added to each well of an opaque black 96-well microplate. The changes in fluorescence intensity were continuously monitored for 1 hour. All measurements were repeated three times with duplicates each time.

### 3,3′-Dipropylthiadicarbocyanine iodide [DiSC_3_(5)] assays

The DiSC_3_(5) assay was carried out to investigate the cytoplasmic membrane depolarization. Mid-log phase bacteria culture was centrifuged and resuspended in sterile Hepes buffer [5 mM (pH 7.4)] to adjust the concentration of bacterial cells to obtain OD_600_ of 0.1. The bacterial suspension was then incubated with 2 μM DiSC_3_(5) for 1 hour without being exposed to the light. The change in fluorescence intensity was monitored by a BioTek multidetector microplate reader with an excitation wavelength of 622 nm and an emission wavelength of 670 nm. After the fluorescence signal was stabilized, 4 μl of compounds (1×, 2×, 4×, and 8× MIC) were immediately added, and the fluorescence intensity was continuously monitored for about 1 hour. All measurements were repeated three times with duplicates each time.

### Fluorescence-based binding assays

Changes in the fluorescence emission spectrum or intensity were assessed for compound **XT17** with and without the presence of targeted proteins. The fluorescent properties of compound **XT17** (excitation wavelength of 350 nm and emission wavelength of 440 nm) were measured using a fluorescent plate reader upon binding to the targets. LTA-targeted protein concentrations ranged from 0.2 to 6.25 μg/ml, while LPS concentrations ranged from 0.78 to 25 μg/ml. The changes in the emission from a constant concentration of fluorescent compound were monitored after adding increasing concentrations of LTA or LPS. All measurements were repeated three times with duplicates each time.

### Displacement from LTA-BODIPY/LPS-BODIPY

The percentage of displacement from LTA-BODIPY/LPS-BODIPY by test compounds was determined according to BODIPY cadaverine displacement assay. BODIPY cadaverine dye was mixed with LTA/LPS at the desired concentrations in a 24-well plate, followed by incubating in the dark for 15 min and adding the dilutions of sample (final concentrations of 64×, 32×, 16×, 8×, 4×, 2×, 1×, and 0.5× MIC) dissolved in DMSO. The mixture was incubated in the dark for 30 min, and then the fluorescence was monitored at an excitation wavelength of 580 nm and an emission wavelength of 620 nm using a BioTek multidetector microplate reader. The percentage of displacement from LTA-BODIPY/LPS-BODIPY was calculated by the following formula: % Displacement = (*F*_S_ − *F*_min_)/(*F*_max_ − *F*_min_) × 100. Where *F*_S_ is the fluorescence intensity observed at a given test compound concentration, *F*_min_ is the fluorescence intensity of BODIPY with LTA/LPS and DMSO, and *F*_max_ is the fluorescence intensity of BODIPY with only DMSO. All tests were carried out at least twice and with biological replicates.

### Growth condition and total RNA extraction

For the challenge experiments, 5 ml of *E. coli* ATCC 25922 at an OD_600_ of 0.5, representing the mid-log phase, was exposed to compound **XT17** (4× MIC) for 2 hours in a biological duplicate. Cultures treated with 4× MIC CPF and without antibiotics served as positive and negative controls, respectively. After exposure, 1-ml aliquots of cells were immediately pelleted at 4°C by centrifugation for 2 min at 2000 rpm. The supernatants were removed and immediately frozen in liquid nitrogen. The samples were stored at −80°C for later total RNA isolation. Total RNA was extracted using the bacterial RNA kit (Omega Bio-Tek, USA) according to the manufacturer’s instructions. The RNA integrity number values were obtained to assess RNA quality using an Agilent Genomics 2200 Tape Station instrument.

### Transcriptome sequencing

A total amount of 2 to 5 ng of the RNA per sample was executed as the input material to construct each cDNA library for RNA-seq using the NEBNext Ultra Directional RNA Library prep kit from Illumina. The quality of the resulting libraries was checked using Agilent High Sensitivity DNA chips to ensure proper library size distribution and the absence of small adapters. Libraries were quantified and normalized by qPCR before being sequenced at 150 cycles with the NextSeq 500 High Output Kit, yielding approximately 9 million, 75 bp, and paired-end reads for each library (*73*).

### Bioinformatic analysis

Read mapping was aligned using Bowtie2 (*74*). HTSeq v0.6.1 was used for quantification of expression levels (*75*). DESeq2 on the R package (v1.12.4) was used to calculate DEGs. Differential expression was computed using edgeR: exactTest for each treatment versus the untreated control (*76*). The *E. coli* database used for mapping was GCF_017357505.1_ASM1735750v1_genomic.fna. Both the GO and KEGG (KEGG Ortholog database) enrichment analyses were done with the Fisher’s exact test. All *P* values were adjusted with the Benjamini-Hochberg procedure to generate false discovery rates (adjusted *P* values). GO enrichment analysis was carried out using the clusterProfiler package on the R platform (*77*). KEGG pathway mapping and figure generation were performed with the PathView website (*78*). COG of protein databases were searched with an *E* value of 1e^-5^. For hierarchal clustering analysis of gene expression, the gene expression levels were normalized to *z* scores of log_10_ (FPKM + 1), where FPKM is fragments per kilobase per million. For hierarchal clustering analysis of the regulation of transcription factors, the unweighted pair group method with arithmetic mean (UPGMA) method was used as the clustering method. Transcription of genes that were not notably regulated was considered unchanged. Volcano plots were generated by plot_volcano from soothsayer (https://github.com/jolespin/soothsayer) in Python v.3.6.6. Directed networks were constructed and plotted using NetworkX and Matplotlib Python packages, respectively. Hierarchical clustering analysis and heatmap calculation were performed with the pheatmap package on the R platform. The umap package on the R platform was used for uniform manifold approximation and projection (UMAP) analysis of FPKM vectors of each transcriptome. The figures were created via the ggplot2 package (*79*).

### Real-time qPCR validation

The real-time qPCR was performed to validate the RNA-seq data of DEGs. All gene-specific primers used in the study are shown in table S9. The qPCR reaction mixture (20 μl) consisted of 2× SYBR Green PCR Master Mix (10 μl, Vazyme, China), template cDNA (100 ng), and forward (0.4 μl, 10 μM) and reverse (0.4 μl, 10 μM) primers. The validation was accomplished on a real-time PCR system (LightCycler 96 instrument, Roche). The thermal cycling conditions involved a two-step cycling profile, which was composed of 95°C (initial melting) for 5 min, followed by 40 cycles of 95°C (melting) for 10 s and 60°C (annealing and amplification) for 30 s. The amplification reaction was performed in triplicate. All data were analyzed using the LightCycler software provided by the manufacturer. The specificity of each primer pair was determined by the presence of a single melting temperature peak. The constitutively expressed and highly conserved *GAPDH* genes produced uniform expression levels varying by less than 0.5 cell type-specific (CTs) between sample conditions and thus served as the reference gene to normalize the gene expression level. The results were lastly analyzed using the 2^-ΔΔCt^ relative expression method (*80*).

### Electrophoretic mobility shift assay

Isolation of DNA from pure culture of *E. coli* ATCC 25922 was carried out by the TIANamp bacterial DNA kit (TIANGEN, China) according to the manufacturer’s instructions. For the gel electrophoresis experiments, DNA product in tris-EDTA (TE) buffer solution [10 mM tris-HCl (pH 8.0) and 1 mM EDTA] was treated with various concentrations of compound **XT17** (from 0 to 32× MIC). A loading buffer containing 10 mM TE (pH 7.5), 0.03% bromophenol blue, 0.03% xylene cyanol FF, 60% glycerol, and 60 mM EDTA was added in the sample following incubation for 30 min at 37°C. The electrophoresis was then performed at 100 V for 2 hours in 1× tris-acetate-EDTA buffer solution [0.04 M tris-acetate (pH 8.0) and 0.001 M EDTA] using a 1% (w/v) agarose gel. The bands were visualized using the ChemiDoc imaging system with ultraviolet light. All of the experiments were repeated at least twice with duplicates each time.

### DNA topoisomerase II (gyrase) supercoiling and topoisomerase IV decatenation inhibition assays

*E. coli* topoisomerase enzymes (II and IV) were supplied by ProFoldin (Worcester, MA), and assays were performed according to the manufacturer’s instructions. Briefly, the DNA supercoiling assay was conducted in a volume of 40 μl containing 24 μl of H_2_O, 4 μl of 10× buffer, 4 μl of 10× relaxed DNA, 4 μl of 10× enzyme, and 4 μl of 10 mM adenosine triphosphate (ATP). Following a 60-min incubation at room temperature, 200 μl of H_2_O was added to each reaction mixture. Topoisomerase IV decatenation inhibition assay was conducted in a volume of 50 μl containing 38.5 μl of H_2_O, 5 μl of 10× buffer, 5 μl of 10× concatenated DNA, 0.5 μl of 100× enzyme, and 1 μl of 10 mM ATP. After 60 min of incubation at 37°C, 5 μl of 0.4 M EDTA was added to stop the reaction. The final concentrations for both assays contained 20 mM tris-HCl (pH 8), 35 mM NH_4_OAc, 4.6% glycerol, 1 mM dithiothreitol, 0.005% Brij35, and 8 mM MgCl_2_. Typically, the supercoiling assay enclosed relaxed plasmid DNA (25 μg/ml), 1 mM ATP, and 20 nM topoisomerase II. Whereas the decatenation assay enclosed concatenated DNA (2 μg/ml), 0.2 mM ATP, and 5 nM topoisomerase IV. The reaction mixture for both assays without ATP served as a negative control.

For high-throughput screening of gyrase and topoisomerase IV inhibitors, reactions in the presence of either CPF or compound **XT17** were carried out with the addition of different concentrations (1× and 4× MIC). In the supercoiling inhibition assay, the fluorescence intensity was measured using freshly prepared 1× H19 dye, which was prepared by diluting 100× H19 dye with 10 mM tris-HCl and 10 mM NaCl (pH 7.0). A total of 250 μl of the 1× H19 dye was mixed with each reaction solution before incubating the mixture at room temperature for 15 min. In the decatenation inhibition assay, the change in fluorescence intensity was monitored using the 1× fluorescence dye after diluting the 20× dye with water. The reaction solution eluted from the column was mixed with 150 μl of 1× fluorescence dye. The fluorescence intensity for both assays was lastly detected at 535 nm using an excitation wavelength of 485 nm. All of the experiments were repeated three times with duplicates each time.

### Molecular docking

The structure of DNA gyrase was retrieved from PDB (PDB code: 6RKV or 2XCT) (*57*, *58*). The protein was prepared by removing the cocrystallized ligand, selected water molecules, and cofactors. The three-dimensional structure of CPF was obtained from DrugBank (ID: DB00537), while the chemical structure of compound **XT17** was obtained from ChemDraw 20 software. The interactive models of gyrase with CPF or compound **XT17** were generated using a CB-Dock2 protein-ligand docking server (https://cadd.labshare.cn/cb-dock2) (*81*, *82*). The hierarchical multifeature alignment approach was used in the FitDock method to fit the initial conformation, which ultimately produced a possible docking conformation. The conformations with the most favorable (least) free binding energy (kilocalories per mole) were designated for analyzing the interactions between the target receptor and ligands by Accelrys Discovery Studio visualizer and PyMOL.

### Acute toxicity studies

All animal experiments were approved by the Experimental Animal Center of Guangzhou Medical University and performed in accordance with the policies of the Ministry of Health of China. Acute toxicity studies of **XT17** were performed in CD-1 mice by intravenous, intraperitoneal, and subcutaneous injection, respectively. The CD-1 mice (*n* = 3) were treated with different doses of **XT17** using the up-and-down procedure. The dose for subsequent animals was determined on the basis of the response of the preceding animal, with gradual adjustments to increase or decrease the dose until the maximum tolerated dose was established. All surviving mice were monitored regularly for any indications of toxicity for 7 days and subsequently euthanized.

### PK studies in mice

Mouse plasma PK studies were performed at Shanghai Ruizhi Chemical Research Co. Ltd. All experiments related to animals were conducted in compliance with the ethical guidelines approved by the Institutional Animal Care and Use Committee (IACUC) of the Shanghai Ruizhi Chemical Research Co. Ltd. Male CD-1 mice (6 to 8 weeks) were treated with the corresponding dose of drugs formulated in 5% glucose solution (D5W) delivered via intravenous injection via tail vein, intraperitoneal injection, or subcutaneous injection. Following administration, approximately 110 μl of blood was taken via facial vein injection at 0.083, 0.25, 0.5, 1, 2, 4, 8, and 24 hours into K_2_EDTA tubes. Blood samples were placed on ice and centrifuged to obtain plasma samples (2000*g*, 5 min under 4°C). Plasma samples were stored at approximately −70°C until analysis. The **XT17** concentration in plasma PK studies was measured by liquid chromatography–tandem mass spectrometry (LC-MS/MS) assay using an LC-MS/MS-22 (Triple Quad 6500) instrument. Plasma concentrations versus time data and PK parameters were estimated from noncompartmental analysis using WinNonlin 8.2.

### In vivo efficacy

Female C57BL/6 mice (average weight, 20 g) were used for the in vivo efficacy experiments. Mice were housed in a specific pathogen–free facility on a 12-hour light-dark cycle, under the condition with ad libitum access to irradiation sterilized rodent diet and aseptic water. All animal tests were performed on experiment-naïve female mice ranging in age from 6 to 8 weeks. These in vivo experiments were approved by the Experimental Animal Center of South China Agricultural University and conducted in accordance with the policies of the Ministry of Health of China.

The in vivo efficacy of compound **XT17** was investigated in a murine corneal infection model. Before infection, the mice were immunosuppressed with three intraperitoneal injections of cyclophosphamide (100 mg/kg) over 5 days. After anesthesia with 2.5% avertin (500 mg/kg, ip), the left corneas of mice were scratched with 15 μl of *S. aureus* ATCC 29213 or *P. aeruginosa* ATCC 9027 (approximately 5 × 10^7^ CFU/ml). Following 1 day of infection, the infected mice were randomly assigned into three groups (*n* = 5) and subsequently administered with 0.5% compound **XT17**, 5% glucose solution, 5% vancomycin, or 0.3% gatifloxacin topically. *S. aureus*–caused murine corneal infections were treated four times a day for three consecutive days. Only 2 days of treatment were given for *P. aeruginosa*–caused murine corneal infections. On the first day, the drug was administered every 20 min for 1 hour and then three times at 2-hour intervals on the second day. The mice were given a 5% glucose solution as a negative control in the absence of any antimicrobial agent treatment. The infected corneas were collected after the mice were euthanized, and the number of viable bacteria was counted using MHA plates.

### Statistical analysis

All statistical analyses were performed using GraphPad Prism software (GraphPad Software Inc., La Jolla, CA, USA) with all data represented as the mean ± SD from at least three independent experiments. Statistical analysis of differences between the experimental groups was performed using the SPSS version 25.0 software. Data analyses were performed using the *t* test or one-way analysis of variance (ANOVA), and a *P* value less than 0.05 was considered statistically significant.
