# Supplementary material for: Development of xanthone derivatives as effective broad-spectrum antimicrobials: Disrupting cell wall and inhibiting DNA synthesis
Source: Sci Adv. 2025 Mar 5;11(10):eadt4723. doi: 10.1126/sciadv.adt4723 (PMC11881906; doi:10.1126/sciadv.adt4723)
Supplement: Supplementary file 1 — Supplementary Text Figs. S1 to S35 Tables S1 to S9 [file sciadv.adt4723_sm.pdf]

Science Advances
AAAS

Supplementary Materials for

Development of xanthone derivatives as effective broad-spectrum antimicrobials: Disrupting cell wall and inhibiting DNA synthesis

Haizhou Li *et al.*

Corresponding author: Shuimu Lin, linshuimu020@163.com; Shouping Liu, liushouping2018@163.com; Wen-Tyng Kang, wntyng@gmail.com

Sci. Adv. **11**, eadt4723 (2025)
DOI: 10.1126/sciadv.adt4723

This PDF file includes:

Supplementary Text
Figs. S1 to S35
Tables S1 to S9

Supplementary Text

1. Chemically synthesis

The purity of each compound was verified by high-pressure liquid chromatography (HPLC). The results showed that every compound was successfully synthesized with high purities (> 95%) (figs. S11 to S35).

1.1 1,3,6-Trihydroxy-9*H*-xanthen-9-one (**XT01**).

Phloroglucinol (1.00 g, 7.93 mmol) and 2,4-dihydroxybenzoic acid (1.22 g, 7.93 mmol) were dissolved in Eaton's reagent (10 mL). The mixture was stirred at 80 °C for 1 h. After the reaction was completed. After the reaction was completed, the reaction mixture was poured into ice water, and a large amount of red-brown solid was precipitated and filtered. The crude product was purified by silica gel column chromatography (petroleum ether/ethyl acetate = 1/1) to yield product **XT01** (1.065 g, 54.9%) as a yellow solid. ^{1}H NMR (400 MHz, CD_3OD) δ 7.95 (d, J = 8.8 Hz, 1H), 6.83 – 6.79 (m, 1H), 6.71 (d, J = 2.2 Hz, 1H), 6.25 (d, J = 2.1 Hz, 1H), 6.13 (d, J = 2.1 Hz, 1H). ^{13}C NMR (100 MHz, CD_3OD) δ 181.19, 166.65, 165.68, 164.61, 159.39, 159.33, 128.24, 114.72, 114.20, 103.35, 103.08, 98.99, 94.97. HRMS (ESI+): calculated for $C_{13}H_8O_5$ $[M + H]^+$ 245.0444, found 245.0438.

1.2 3,6-Bis(3-bromopropoxy)-1-hydroxy-9*H*-xanthen-9-one (**XT02**).

To a solution of **XT01** (200.0 mg, 818.99 μmol) in acetone (10 mL), K_2CO_3 (565.9 mg, 4.09 mmol) and 1,3-dibromopropane (5.99 mL, 53.70 mmol) were added, and then the reaction was stirred at 65 °C for 6 h. After the reaction was completed, the reaction mixture was extracted with dichloromethane and water. The organic phase was concentrated under reduced pressure. Then the crude product was purified by silica gel column chromatography (petroleum ether/dichloromethane = 1/2) to provide the product **XT02** (948.6 mg, 54.5%) as a yellow solid. ^{1}H NMR (400 MHz, $CDCl_3$) δ 12.95 (s, 1H), 8.12 (d, J = 8.9 Hz, 1H), 6.94 – 6.89 (m, 1H), 6.83 (d, J = 2.3 Hz, 1H), 6.38 (d, J = 2.3 Hz, 1H), 6.32 (d, J = 2.3 Hz, 1H), 4.25 – 4.15 (m, 4H), 3.66 – 3.57 (m, 4H), 2.42 – 2.32 (m, 4H). ^{13}C NMR (100 MHz, $CDCl_3$) δ 180.14, 165.36, 164.39, 163.57, 157.99, 157.78, 127.49, 114.53, 113.48, 103.78, 100.83, 97.49, 93.26, 66.09, 65.98, 32.04, 32.02, 29.66, 29.62. HRMS (ESI+): calculated for $C_{19}H_{18}Br_2O_5$ $[M + H]^+$

486.9573, found 486.9558.

1.3 3,6-Bis(3-bromopropoxy)-1-((3-methylbut-2-en-1-yl)oxy)-9*H*-xanthen-9-one (**XT03**).

To a solution of **XT02** (200.0 mg, 411.39 μmol) in acetone (10 mL), Cs_2CO_3 (335.1 mg, 1.03 mmol) and 1-bromo-3-methylbut-2-ene (118.8 μL, 1.03mmol) were added, and then the reaction was stirred at 65 °C for 12 h. After the reaction was completed, the reaction mixture was extracted with ethyl acetate and water. The organic phase was concentrated under reduced pressure. Then the crude product was purified by silica gel column chromatography (petroleum ether/ethyl acetate = 3/1) to yield the pure product **XT03** (189.6 mg, 83.2%) as a yellow gel. ^{1}H NMR (400 MHz, $CDCl_3$) δ 8.16 (d, J = 8.8 Hz, 1H), 6.87 – 6.82 (m, 1H), 6.74 (d, J = 2.4 Hz, 1H), 6.42 (d, J = 2.3 Hz, 1H), 6.31 (d, J = 2.3 Hz, 1H), 5.64 – 5.58 (m, 1H), 4.65 (d, J = 6.4 Hz, 2H), 4.17 (t, J = 5.8 Hz, 4H), 3.63 – 3.57 (m, 4H), 2.39 – 2.30 (m, 4H), 1.79 (s, 3H), 1.75 (s, 3H). ^{13}C NMR (100 MHz, $CDCl_3$) δ 174.75, 163.48, 163.21, 161.34, 159.85, 156.63, 137.75, 128.38, 119.44, 117.22, 112.87, 107.61, 100.29, 96.45, 93.44, 66.50, 65.88, 65.85, 32.11, 32.09, 29.77, 29.72, 25.93, 18.50. HRMS (ESI+): calculated for $C_{19}H_{18}Br_2O_5$ $[M + H]^+$ 486.9573, found 486.9558.

1.4 (*Z*)-3,6-Bis(3-bromopropoxy)-1-((3,7-dimethylocta-2,6-dien-1-yl)oxy)-9*H*-xanthen-9-one (**XT04**).

The target compound was prepared from **XT02** (200.0 mg, 411.39 μmol), Cs_2CO_3 (335.1 mg, 1.03 mmol) and geranyl bromide (204.9 μL, 1.03 mmol) according to the similar approach used to synthesize **XT03**. The crude product was purified by silica gel column chromatography (petroleum ether/ethyl acetate = 3/1) to yield the pure product **XT04** (196.1 mg, 76.6%) as a white solid. ^{1}H NMR (400 MHz, $CDCl_3$) δ 8.22 – 8.16 (m, 1H), 6.92 – 6.83 (m, 1H), 6.78 (d, J = 1.6 Hz, 1H), 6.51 – 6.42 (m, 1H), 6.33 (d, J = 1.5 Hz, 1H), 5.62 (t, J = 6.2 Hz, 1H), 5.09 (t, J = 6.6 Hz, 1H), 4.71 (d, J = 6.2 Hz, 2H), 4.20 (t, J = 5.8 Hz, 4H), 3.66 – 3.59 (m, 4H), 2.43 – 2.32 (m, 4H), 2.17 – 2.04 (m, 4H), 1.76 (s, 3H), 1.67 (s, 3H), 1.60 (s, 3H). ^{13}C NMR (100 MHz, $CDCl_3$) δ 174.84, 163.48, 163.24, 161.36, 159.88, 156.66, 140.79, 131.85, 128.44, 123.93, 119.32, 117.24, 112.89, 107.69, 100.31, 96.58, 93.48, 66.63, 65.87, 65.85, 39.64, 32.12, 32.09, 29.75,

29.67, 26.41, 25.76, 17.80, 16.94. HRMS (ESI+): calculated for $C_{29}H_{34}Br_2O_5$ $[M + H]^+$ 623.0825, found 623.0818.

1.5 3,6-Bis(3-bromopropoxy)-1-methoxy-9*H*-xanthen-9-one (**XT05**).

The target compound was prepared from **XT02** (100.0 mg, 205.70 μmol), Cs_2CO_3 (167.6 mg, 514.24 μmol) and iodomethane (128.1 μL, 2.06 mmol) according to the similar approach used to synthesize **XT03**. The crude product was purified by silica gel column chromatography (petroleum ether/ethyl acetate = 2/1) to yield the pure product **XT05** (80.3 mg, 78.0%) as a yellow gel. ^{1}H NMR (400 MHz, $CDCl_3$) δ 8.19 (d, *J* = 8.9 Hz, 1H), 6.91 – 6.85 (m, 1H), 6.79 (d, *J* = 2.3 Hz, 1H), 6.48 (d, *J* = 2.3 Hz, 1H), 6.34 (d, *J* = 2.3 Hz, 1H), 4.23 – 4.12 (m, 4H), 3.97 (s, 3H), 3.45 – 3.33 (m, 4H), 2.39 – 2.27 (m, 4H). ^{13}C NMR (100 MHz, $CDCl_3$) δ 174.95, 163.65, 163.32, 162.09, 159.90, 156.66, 128.43, 117.18, 112.99, 107.39, 100.30, 95.41, 93.44, 67.89, 67.87, 56.48, 32.65, 32.63, 2.18, 2.05. HRMS (ESI+): calculated for $C_{20}H_{20}Br_2O_5$ $[M + H]^+$ 500.9730, found 500.9724.

1.6 3,6-Bis(3-bromopropoxy)-1-(4,4,4-trifluorobutoxy)-9*H*-xanthen-9-one (**XT06**).

The target compound was prepared from **XT02** (100.0 mg, 205.70 μmol), Cs_2CO_3 (167.6 mg, 514.24 μmol) and 1,1,1-trifluoro-4-iodobutane (66.2 μL, 514.24 μmol) according to the similar approach used to synthesize **XT03**. The crude product was purified by silica gel column chromatography (petroleum ether/ethyl acetate = 3/1) to yield the pure product **XT06** (41.2 mg, 33.6%) as a white solid. ^{1}H NMR (400 MHz, $CDCl_3$) δ 8.18 (d, *J* = 8.9 Hz, 1H), 6.92 – 6.85 (m, 1H), 6.78 (d, *J* = 2.1 Hz, 1H), 6.51 – 6.47 (m, 1H), 6.30 (d, *J* = 2.2 Hz, 1H), 4.23 – 4.06 (m, 6H), 3.66 – 3.35 (m, 4H), 2.65 – 2.48 (m, 2H), 2.43 – 2.27 (m, 4H), 2.25 – 2.14 (m, 2H). ^{13}C NMR (100 MHz, $CDCl_3$) δ 174.79, 163.54, 163.33, 160.99, 159.86, 156.68, 128.31, 126.06, 117.17, 112.99, 107.55, 100.41, 96.36, 93.80, 67.91, 67.42, 65.91, 32.60, 32.04, 30.78, 30.50, 29.73, 22.17. HRMS (ESI+): calculated for $C_{23}H_{23}Br_2F_3O_5$ $[M + H]^+$ 596.9917, found 596.9907.

1.7 3,6-Bis(3-aminopropoxy)-1-((3-methylbut-2-en-1-yl)oxy)-9*H*-xanthen-9-one (**XT07**).

To a solution of **XT03** (56.0 mg, 101.03 μmol) in DMF (5 mL), ammonia (3 mL)

was added, and then the reaction was stirred at 50 °C for 24 h. After the reaction was completed, the reaction mixture was directly concentrated under reduced pressure. Then the crude product was purified by HPLC to yield the pure product **XT07** (14.6 mg, 33.9%) as a brown solid. ^{1}H NMR (400 MHz, CD_3OD) δ 8.10 (d, *J* = 8.8 Hz, 1H), 7.03 – 6.95 (m, 2H), 6.63 (s, 1H), 6.49 (d, *J* = 2.0 Hz, 1H), 5.60 – 5.52 (m, 1H), 4.73 (d, *J* = 6.4 Hz, 2H), 4.29 – 4.20 (m, 4H), 3.19 (t, *J* = 7.3 Hz, 4H), 2.26 – 2.17 (m, 4H), 1.83 – 1.77 (m, 6H). ^{13}C NMR (100 MHz, DMSO-d6) δ 173.27, 163.84, 163.46, 161.06, 159.50, 156.43, 137.45 (2×CH), 127.87, 120.05 (2×CH), 116.71, 113.61, 106.83, 100.87, 66.29, 66.10, 66.08, 49.07, 45.41, 36.45 (2×CH_2), 25.98, 18.60. HRMS (ESI+): calculated for $C_{24}H_{30}N_2O_5$ $[M + H]^+$ 427.2227, found 427.2218.

1.8 3,6-Bis(3-(methylamino)propoxy)-1-((3-methylbut-2-en-1-yl)oxy)-9*H*-xanthen-9-one (**XT08**).

To a solution of **XT03** (50.0 mg, 90.21 μmol) in DMF (5 mL), methanamine (1 mL) were added, and then the reaction was stirred at room temperature for 24 h. After the reaction was completed, the reaction mixture was extracted with 1-butanol and water, and the organic phase was concentrated under reduced pressure. Then the crude product was purified by HPLC to provide the pure product **XT08** (31.7 mg, 77.3%) as a yellow gel. ^{1}H NMR (400 MHz, CD_3OD) δ 8.07 – 8.03 (m, 1H), 6.97 – 6.82 (m, 2H), 6.45 (d, *J* = 35.4 Hz, 2H), 5.55 (t, *J* = 5.9 Hz, 1H), 4.68 (d, *J* = 6.3 Hz, 2H), 4.28 – 4.11 (m, 4H), 3.24 (t, *J* = 7.2 Hz, 4H), 2.81 – 2.72 (m, 6H), 2.29 – 2.17 (m, 4H), 1.82 – 1.77 (m, 6H). ^{13}C NMR (100 MHz, CD_3OD) δ 176.62, 165.34, 164.97, 162.34, 161.16, 158.11, 138.98, 128.81, 120.64, 117.75, 114.35, 108.05, 101.51, 97.80, 94.79, 67.35, 66.92, 66.86, 47.94, 47.91, 33.81 (2×CH_3), 26.98, 26.95, 25.92, 18.40. HRMS (ESI+): calculated for $C_{26}H_{34}N_2O_5$ $[M + H]^+$ 455.2540, found 455.2527.

1.9 3,6-Bis(3-(diethylamino)propoxy)-1-((3-methylbut-2-en-1-yl)oxy)-9*H*-xanthen-9-one (**XT09**).

The target compound was prepared from **XT03** (65.6 mg, 118.35 μmol) and dimethylamine (1 mL) according to the similar approach used to synthesize **XT08**. The product **XT09** (38.3 mg) was obtained in 60.1% yield as a brown gel. ^{1}H NMR (400 MHz, CD_3OD) δ 8.09 (d, *J* = 8.7 Hz, 1H), 7.00 – 6.94 (m, 2H), 6.60 (s, 1H), 6.46 (s,

1H), 5.55 (t, J = 6.4 Hz, 1H), 4.72 (d, J = 6.3 Hz, 2H), 4.27 – 4.20 (m, 4H), 3.37 – 3.32 (m, 4H), 3.29 – 3.23 (m, 8H), 2.29 – 2.22 (m, 4H), 1.83 – 1.76 (m, 6H), 1.35 (t, J = 7.3 Hz, 12H). ^{13}C NMR (100 MHz, CD_3OD) δ 176.50, 165.31, 164.95, 162.32, 161.14, 158.10, 138.82, 128.80, 120.70, 117.75, 114.32, 108.03, 101.49, 97.75, 94.75, 67.35, 66.76, 66.71, 50.03 (2×CH_2), 48.30 (4×CH_2), 25.93, 24.85, 24.81, 18.42, 9.21 (2×CH_3), 9.20 (2×CH_3). HRMS (ESI+): calculated for $C_{32}H_{46}N_2O_5$ $[M + H]^+$ 539.3479, found 539.3461.

1.10 3,6-Bis(3-(dibutylamino)propoxy)-1-((3-methylbut-2-en-1-yl)oxy)-9*H*-xanthen-9-one (**XT10**).

The target compound was prepared from **XT03** (42.8 mg, 77.22 μmol) and dibutylamine (1 mL) according to the similar approach used to synthesize **XT08**. The product **XT10** (32.5 mg) was obtained in 64.7% yield as a yellow gel. ^{1}H NMR (400 MHz, CD_3OD) δ 8.08 (d, J = 8.8 Hz, 1H), 6.99 – 6.88 (m, 2H), 6.56 (s, 1H), 6.42 (s, 1H), 5.55 (t, J = 6.3 Hz, 1H), 4.71 (d, J = 6.3 Hz, 2H), 4.26 – 4.12 (m, 4H), 3.07 (t, J = 13.9 Hz, 4H), 2.94 – 2.79 (m, 8H), 2.20 – 2.05 (m, 4H), 1.83 – 1.74 (m, 6H), 1.68 – 1.55 (m, 8H), 1.45 – 1.33 (m, 8H), 0.97 (t, J = 7.3 Hz, 12H). ^{13}C NMR (100 MHz, CD_3OD) δ 176.61, 165.57, 165.19, 162.35, 161.23, 158.18, 138.78, 128.83, 120.74, 117.66, 114.35, 107.97, 101.42, 97.78, 94.75, 67.32, 67.22, 67.21, 54.46 (2×CH_2), 54.42 (2×CH_2), 51.21, 51.18, 28.23 (2×CH_2), 28.11, 25.94 (2×CH_2), 25.82, 25.76, 21.35 (2×CH_2), 21.30 (2×CH_2), 18.43, 14.19 (2×CH_3), 14.15 (2×CH_3). HRMS (ESI+): calculated for $C_{40}H_{62}N_2O_5$ $[M + H]^+$ 651.4731, found 651.4724.

1.11 1-((3-Methylbut-2-en-1-yl)oxy)-3,6-bis(3-thiomorpholinopropoxy)-9*H*-xanthen-9-one (**XT11**).

The target compound was prepared from **XT03** (42.8 mg, 77.22 μmol) and thiomorpholine (1 mL) according to the similar approach used to synthesize **XT08**. The product **XT11** (40.0 mg) was obtained in 86.5% yield as a brown gel. ^{1}H NMR (400 MHz, CD_3OD) δ 8.08 (d, J = 8.8 Hz, 1H), 6.99 – 6.87 (m, 2H), 6.56 (s, 1H), 6.42 (s, 1H), 5.56 (t, J = 6.3 Hz, 1H), 4.72 (d, J = 6.2 Hz, 2H), 4.23 – 4.09 (m, 4H), 3.08 (t, J = 9.1 Hz, 8H), 2.93 – 2.77 (m, 12H), 2.20 – 2.07 (m, 4H), 1.87 – 1.74 (m, 6H). ^{13}C NMR (100 MHz, CD_3OD) δ 176.61, 165.66, 165.30, 162.29, 161.17, 158.15, 138.73, 128.75,

120.79, 117.55, 114.35, 107.87, 101.32, 97.74, 94.69, 67.60, 67.59, 67.32, 56.60 (2×CH_2), 55.96 (2×CH_2), 55.94 (2×CH_2), 27.65 (2×CH_2), 27.58 (2×CH_2), 26.28, 26.25, 25.94, 18.44. HRMS (ESI+): calculated for $C_{32}H_{42}N_2O_5S_2$ $[M + H]^+$ 599.2608, found 599.2600.

1.12 1-((3-Methylbut-2-en-1-yl)oxy)-3,6-bis(3-(pyrrolidin-1-yl)propoxy)-9*H*-xanthen-9-one (**XT12**).

The target compound was prepared from **XT03** (42.8 mg, 77.22 μmol) and pyrrolidine (1 mL) according to the similar approach used to synthesize **XT08**. The product **XT12** (28.0 mg) was obtained in 67.8% yield as a yellow gel. ^1H NMR (400 MHz, CD_3OD) δ 8.11 (d, *J* = 8.8 Hz, 1H), 7.03 – 6.94 (m, 2H), 6.62 (s, 1H), 6.47 (s, 1H), 5.57 (t, *J* = 6.3 Hz, 1H), 4.74 (d, *J* = 6.3 Hz, 2H), 4.29 – 4.18 (m, 4H), 3.29 (t, *J* = 6.6 Hz, 12H), 2.31 – 2.22 (m, 4H), 2.12 – 2.04 (m, 8H), 1.87 – 1.75 (m, 6H). ^{13}C NMR (100 MHz, CD_3OD) δ 176.68, 165.51, 165.11, 162.40, 161.25, 158.20, 138.95, 128.85, 120.64, 117.73, 114.35, 108.04, 101.34, 97.78, 94.78, 67.34, 66.98, 66.95, 55.18 (2×CH_2), 55.16 (2×CH_2), 53.60, 53.57, 27.37, 27.35, 25.91, 24.08 (2×CH_2), 24.06 (2×CH_2), 18.39. HRMS (ESI+): calculated for $C_{32}H_{42}N_2O_5$ $[M + H]^+$ 535.3166, found 535.3161.

1.13 3,6-Bis(3-((2-(bis(2-aminoethyl)amino)ethyl)amino)propoxy)-1-((3-methylbut-2-en-1-yl)oxy)-9*H*-xanthen-9-one **(XT13).**

The target compound was prepared from **XT03** (50.0 mg, 90.21 μmol)and tris(aminoethyl)amine (1 mL) according to the similar approach used to synthesize **XT08**. The product **XT13** (28.0 mg) was obtained in 45.5% yield as a yellow gel. ^1H NMR (400 MHz, CD_3OD) δ 8.02 (d, *J* = 8.8 Hz, 1H), 7.00 – 6.79 (m, 2H), 6.58 – 6.23 (m, 2H), 5.54 (t, 1H), 4.69 (d, *J* = 5.2 Hz, 2H), 4.28 – 4.09 (m, 4H), 3.27 – 3.04 (m, 14H), 2.92 – 2.68 (m, 14H), 2.36 – 2.21 (m, 4H), 1.80 (d, *J* = 6.2 Hz, 6H). ^{13}C NMR (100 MHz, CD_3OD) δ 170.23, 165.15, 164.78, 162.04, 160.86, 157.84, 138.63, 128.55, 120.51, 117.41, 114.16, 107.73, 101.11, 97.55, 94.57, 67.17, 66.64, 66.53, 52.20 (4×CH_2), 51.24 (2×CH_2), 46.17 (2×CH_2), 46.14 (2×CH_2), 37.78 (4×CH_2), 26.78, 26.76, 25.76, 18.28. HRMS (ESI+): calculated for $C_{36}H_{60}N_8O_5$ $[M + H]^+$ 685.4759, found 685.4748.

1.14 3,6-Bis(3-(methylamino)propoxy)-1-(4,4,4-trifluorobutoxy)-9*H*-xanthen-9-one

(**XT16**).

The target compound was prepared from **XT06** (39.3 mg, 65.91 μmol) and methanamine (1 mL) according to the similar approach used to synthesize **XT08**. The product **XT16** (15.5 mg) was obtained in 47.4% yield as a yellow gel. ^{1}H NMR (400 MHz, CD_3OD) δ 8.08 (d, *J* = 8.8 Hz, 1H), 7.03 – 6.92 (m, 2H), 6.63 (s, 1H), 6.49 (s, 1H), 4.28 – 4.11 (m, 6H), 3.25 – 3.14 (m, 4H), 2.73 (s, 6H), 2.59 – 2.47 (m, 2H), 2.24 – 2.08 (m, 6H). ^{13}C NMR (100 MHz, CD_3OD) δ 176.68, 165.47, 165.01, 162.31, 161.17, 158.20, 130.40, 128.82, 117.83, 114.42, 107.98, 101.59, 97.42, 95.04, 68.67, 66.93 (2×CH_2), 47.93, 47.89, 33.80, 31.55, 31.26, 26.99, 26.95, 23.01. HRMS (ESI+): calculated for $C_{25}H_{31}F_3N_2O_5$ $[M + H]^+$ 497.2258, found 497.2249.

1.15 1,1'-(((1-((3-Methylbut-2-en-1-yl)oxy)-9-oxo-9*H*-xanthene-3,6-diyl)bis(oxy))bis(propane-3,1-diyl))diguanidine (**XT17**).

To a solution of **XT03** (50.0 mg, 90.21 μmol) in DMF (5 mL), ammonia (3 mL) was added, and then the reaction was stirred at 50 °C for 24 h. After the reaction was completed, the reaction mixture was directly concentrated under reduced pressure. To a solution of the residue in DMF (5 mL), 1*H*-pyrazole-1-carboximidamide hydrochloride (33.1 mg, 225.49 μmol) and *N,N*-diisopropylethylamine (37.3 μL, 225.49 μmol) were added, and then the reaction mixture was stirred at room temperature for 24 h. After the reaction was completed, the reaction mixture was directly concentrated under reduced pressure. Then the crude product was purified by HPLC to yield the pure product **XT17** (23.7 mg, 51.5%) as a brown solid. ^{1}H NMR (400 MHz, DMSO-d6) δ 9.06 (d, *J* = 36.1 Hz, 2H), 7.98 – 7.74 (m, 7H), 7.04 – 6.90 (m, 2H), 6.61 (s, 1H), 6.51 (s, 1H), 5.48 (t, *J* = 5.7 Hz, 1H), 4.62 (d, *J* = 5.9 Hz, 2H), 4.21 – 4.12 (m, 4H), 3.29 – 3.20 (m, 4H), 2.01 – 1.91 (m, 4H), 1.77 (s, 3H), 1.73 (s, 3H). ^{13}C NMR (100 MHz, DMSO-d6) δ 172.86, 167.19, 163.49, 163.10, 160.61, 159.10, 157.57, 156.03, 137.00, 127.44, 119.63, 116.24, 113.19, 106.36, 100.39, 96.60, 93.54, 65.82, 65.74 (2×CH_2), 37.56 (2×CH_2), 27.98, 25.56, 18.18 (2×CH_3). HRMS (ESI+): calculated for $C_{26}H_{34}N_6O_5$ $[M + H]^+$ 511.2663, found 511.2648.

1.16 (*Z*)-1,1'-(((1-((3,7-Dimethylocta-2,6-dien-1-yl)oxy)-9-oxo-9*H*-xanthene-3,6-diyl)bis(oxy))bis(propane-3,1-diyl))diguanidine (**XT18**).

The target compound was prepared from **XT04** (93.4 mg, 150.07 μmol) and ammonia (3 mL) according to the similar approach used to synthesize **XT17**. The product **XT18** (17.0 mg) was obtained in 19.6% yield as a brown gel. ^{1}H NMR (400 MHz, DMSO-d6) δ 9.04 (d, J = 39.7 Hz, 2H), 7.97 – 7.80 (m, 7H), 6.98 (d, J = 7.5 Hz, 2H), 6.62 (s, 1H), 6.51 (s, 1H), 5.48 (t, J = 6.0 Hz, 1H), 5.15 – 5.02 (m, 1H), 4.71 – 4.58 (m, 2H), 4.17 (s, 4H), 3.27 – 3.23 (m, 4H), 2.14 – 2.04 (m, 4H), 1.99 – 1.94 (m, 4H), 1.73 (s, 3H), 1.63 (s, 3H), 1.57 (s, 3H). ^{13}C NMR (100 MHz, DMSO-d6) δ 173.37, 167.73, 163.98, 163.61, 161.08, 159.59, 158.06, 158.02, 156.53, 140.54 (2×C), 131.58 (2×CH), 124.29 (2×CH), 119.93 (2×C), 116.74, 106.91, 66.25, 66.23, 49.12, 38.05 (2×CH_2), 38.04, 28.48, 28.45, 26.33, 25.98, 18.10, 16.96. HRMS (ESI+): calculated for $C_{31}H_{42}N_6O_5$ $[M + H]^+$ 579.3289, found 579.3275.

1.17 1,1'-(((1-Methoxy-9-oxo-9*H*-xanthene-3,6-diyl)bis(oxy))bis(propane-3,1-diyl))diguanidine (**XT19**).

The target compound was prepared from **XT05** (96.8 mg, 193.53 μmol) and ammonia (3 mL) according to the similar approach used to synthesize **XT17**. The product **XT19** (48.8 mg) was obtained in 55.2% yield as a white solid. ^{1}H NMR (400 MHz, DMSO-d6) δ 8.94 (d, J = 27.2 Hz, 2H), 7.98 – 7.71 (m, 7H), 7.03 – 6.91 (m, 2H), 6.61 (d, J = 2.0 Hz, 1H), 6.48 (d, J = 2.1 Hz, 1H), 4.25 – 4.09 (m, 4H), 3.79 – 3.70 (m, 7H), 2.05 – 1.86 (m, 4H). ^{13}C NMR (100 MHz, DMSO-d6) δ 173.41, 167.51, 164.12, 163.64, 161.97, 159.61, 158.03, 156.54, 127.95, 116.70, 113.69, 106.68, 100.93, 96.16, 94.07, 66.26, 56.70, 49.12, 38.07 (2×CH_2), 28.46 (2×CH_2). HRMS (ESI+): calculated for $C_{22}H_{28}N_6O_5$ $[M + H]^+$ 457.2194, found 457.2185.

1.18 3,6-Bis(2-bromoethoxy)-1-hydroxy-9*H*-xanthen-9-one (**XT20**).

The target compound was prepared from **XT01** (200.0 mg, 818.99 μmol), K_2CO_3 (565.9 mg, 4.09 mmol) and 1,2-dibromoethane (1.06 mL, 12.28 mmol) according to the similar approach used to synthesize **XT02**. The crude product was purified by silica gel column chromatography (petroleum ether/dichloromethane = 1/9) to yield the pure product **XT20** (160.0 mg, 42.6%) as a white solid. ^{1}H NMR (400 MHz, DMF-d7) δ 13.02 (s, 1H), 8.11 (d, J = 9.1 Hz, 1H), 7.19 – 7.08 (m, 2H), 6.63 (d, J = 2.4 Hz, 1H), 6.44 – 6.40 (m, 1H), 4.77 – 4.50 (m, 4H), 4.11 – 3.87 (m, 4H). ^{13}C NMR (100 MHz, DMF-d7) δ

179.97, 165.34, 164.48, 163.43, 158.05, 157.88, 127.20, 114.27, 114.13, 103.45, 101.40, 97.65, 93.44, 69.13, 69.01, 30.58, 30.54. HRMS (ESI+): calculated for $C_{17}H_{14}Br_2O_5$ $[M + H]^+$ 458.9260, found 458.9250.

1.19 3,6-Bis(4-bromobutoxy)-1-hydroxy-9*H*-xanthen-9-one (**XT21**).

The target compound was prepared from **XT01** (200.0 mg, 818.99 μmol), K_2CO_3 (565.9 mg, 4.09 mmol) and 1,4-dibromobutane (1.47 mL, 12.28 mmol) according to the similar approach used to synthesize **XT03**. The crude product was purified using by silica gel column chromatography (petroleum ether/dichloromethane = 1/2) to yield the pure product **XT21** (215.9 mg, 52.4%) as a yellow solid. ^1H NMR (400 MHz, $CDCl_3$) δ 12.97 – 12.89 (m, 1H), 8.11 – 8.03 (m, 1H), 6.89 – 6.82 (m, 1H), 6.77 – 6.71 (m, 1H), 6.35 – 6.29 (m, 1H), 6.28 – 6.22 (m, 1H), 4.12 – 3.99 (m, 4H), 3.55 – 3.44 (m, 4H), 2.15 – 1.94 (m, 8H). ^{13}C NMR (100 MHz, $CDCl_3$) δ 180.04, 165.51, 164.54, 163.48, 157.93, 157.69, 127.31, 114.29, 113.43, 103.57, 100.63, 97.35, 93.17, 67.71, 67.58, 33.35, 33.31, 29.38, 29.36, 27.71 (2×CH_2). HRMS (ESI+): calculated for $C_{21}H_{22}Br_2O_5$ $[M + H]^+$ 514.9886, found 514.9871.

1.20 3,6-Bis((6-bromohexyl)oxy)-1-hydroxy-9*H*-xanthen-9-one (**XT22**).

The target compound was prepared from **XT01** (200.0 mg, 818.99 μmol), K_2CO_3 (565.9 mg, 4.09 mmol) and 1,6-dibromohexane (1.02 mL, 12.28 mmol) according to the similar approach used to synthesize **XT03**. The crude product was purified by silica gel column chromatography (petroleum ether/dichloromethane = 8/1) to yield the pure product **XT22** (299.5 mg, 64.1%) as a yellow solid. ^1H NMR (400 MHz, $CDCl_3$) δ 12.98 (s, 1H), 8.12 (d, J = 8.9 Hz, 1H), 6.94 – 6.87 (m, 1H), 6.80 (d, J = 2.3 Hz, 1H), 6.37 (d, J = 2.3 Hz, 1H), 6.31 (d, J = 2.3 Hz, 1H), 4.09 – 4.01 (m, 4H), 3.46 – 3.41 (m, 4H), 1.96 – 1.80 (m, 8H), 1.56 – 1.47 (m, 8H). ^{13}C NMR (100 MHz, $CDCl_3$) δ 180.07, 165.74, 164.77, 163.47, 157.98, 157.73, 127.25, 114.18, 113.49, 103.49, 100.59, 97.36, 93.19, 68.56, 68.44, 33.85, 33.84, 32.70, 32.69, 29.78, 28.88, 27.95 (2×CH_2), 25.30, 25.29. HRMS (ESI+): calculated for $C_{25}H_{30}Br_2O_5$ $[M + H]^+$ 571.0512, found 571.0500.

1.21 3,6-Bis((8-bromooctyl)oxy)-1-hydroxy-9*H*-xanthen-9-one (**XT23**).

The target compound was prepared from **XT01** (200.0 mg, 818.99 μmol), K_2CO_3 (565.9 mg, 4.09 mmol) and 1,8-dibromooctane (2.26 mL, 12.28 mmol) according to the similar

approach used to synthesize **XT03**. The crude product was purified by silica gel column chromatography (petroleum ether/dichloromethane = 9/1) to obtain the pure product **XT23** (262.6 mg, 51.2%) as a yellow solid. ^{1}H NMR (400 MHz, $CDCl_3$) δ 12.98 (s, 1H), 8.10 (d, *J* = 8.9 Hz, 1H), 6.92 – 6.86 (m, 1H), 6.78 (d, *J* = 2.3 Hz, 1H), 6.35 (d, *J* = 2.3 Hz, 1H), 6.30 (d, *J* = 2.3 Hz, 1H), 4.08 – 3.98 (m, 4H), 3.41 (t, *J* = 6.8 Hz, 4H), 1.92 – 1.78 (m, 8H), 1.49 – 1.34 (m, 16H). ^{13}C NMR (100 MHz, $CDCl_3$) δ 180.18, 165.86, 164.89, 163.55, 158.08, 157.81, 127.35, 114.23, 113.54, 103.53, 100.66, 97.37, 93.29, 68.77, 68.64, 34.06, 34.05, 32.84, 32.82, 29.20 (2×CH_2), 28.99 (2×CH_2), 28.74 (2×CH_2), 28.16, 28.14, 25.95, 25.93. HRMS (ESI+): calculated for $C_{29}H_{38}Br_2O_5$ $[M + H]^+$ 627.1138, found 627.1125.

1.22 3,6-bis((12-bromododecyl)oxy)-1-hydroxy-9*H*-xanthen-9-one (**XT24**).

The target compound was prepared from **XT01** (100.0 mg, 409.50 μmol), K_2CO_3 (283.0 mg, 2.05 mmol) and 1,12-dibromododecane (2.02 g, 6.14 mmol) according to the similar approach used to synthesize **XT03**. The crude product was purified by silica gel column chromatography (petroleum ether/dichloromethane = 10/1) to yield the pure product **XT24** (173.5 mg, 57.4%) as a white solid. ^{1}H NMR (400 MHz, $CDCl_3$) δ 12.98 (s, 1H), 8.12 (d, *J* = 8.9 Hz, 1H), 6.95 – 6.87 (m, 1H), 6.79 (d, *J* = 2.3 Hz, 1H), 6.37 (d, *J* = 2.2 Hz, 1H), 6.31 (d, *J* = 2.2 Hz, 1H), 4.10 – 3.98 (m, 4H), 3.40 (t, *J* = 6.9 Hz, 4H), 1.89 – 1.77 (m, 8H), 1.66 – 1.56 (m, 4H), 1.37 – 1.24 (m, 28H). ^{13}C NMR (100 MHz, $CDCl_3$) δ 180.17, 165.90, 164.92, 163.54, 158.07, 127.32, 114.20, 113.54, 103.50, 100.64, 100.03, 97.36, 93.29, 68.85, 68.72, 34.16 (2×CH_2), 32.90 (2×CH_2), 29.59 (6×CH_2), 29.50 (2×CH_2), 29.39, 29.38, 29.03 (2×CH_2), 28.84 (2×CH_2), 28.24 (2×CH_2), 26.03, 26.01. HRMS (ESI+): calculated for $C_{37}H_{54}Br_2O_5$ $[M + H]^+$ 739.2390, found 739.2364.

1.23 3,6-Bis(2-bromoethoxy)-1-((3-methylbut-2-en-1-yl)oxy)-9*H*-xanthen-9-one (**XT25**).

The target compound was prepared from **XT20** (88.0 mg, 192.10 μmol), Cs_2CO_3 (156.5 mg, 480.24 μmol) and 1-bromo-3-methylbut-2-ene (55.5 μL, 480.24 μmol) according to the similar approach used to synthesize **XT03**. The crude product was purified by silica gel column chromatography (petroleum ether/ethyl acetate = 3/1) to provide the

pure product **XT25** (44.6 mg, 44.2%) as a yellow solid. ^{1}H NMR (400 MHz, $CDCl_3$) δ 8.19 (d, *J* = 8.9 Hz, 1H), 6.91 – 6.85 (m, 1H), 6.76 – 6.73 (m, 1H), 6.44 – 6.38 (m, 1H), 6.35 (d, *J* = 2.3 Hz, 1H), 5.67 – 5.57 (m, 1H), 4.66 (d, *J* = 6.4 Hz, 2H), 4.36 (t, *J* = 6.2 Hz, 4H), 3.71 – 3.64 (m, 4H), 1.80 (s, 3H), 1.76 (s, 3H). ^{13}C NMR (100 MHz, $CDCl_3$) δ 174.66, 162.86, 162.57, 161.49, 159.81, 156.54, 137.92, 128.62, 119.31, 117.57, 112.78, 107.90, 100.66, 96.60, 93.41, 68.18, 68.08, 66.57, 28.51, 28.48, 25.93, 18.51. HRMS (ESI+): calculated for $C_{22}H_{22}Br_2O_5$ $[M + H]^+$ 526.9886, found 526.9879.

1.24 3,6-Bis(4-bromobutoxy)-1-((3-methylbut-2-en-1-yl)oxy)-9*H*-xanthen-9-one (**XT26**).

The target compound was prepared from **XT21** (30.0 mg, 58.34 μmol), Cs_2CO_3 (47.5 mg, 145.85 μmol) and 1-bromo-3-methylbut-2-ene (16.85 μL, 145.85 μmol) according to the similar approach used to synthesize **XT03**. The crude product was purified by silica gel column chromatography (petroleum ether/ethyl acetate = 3/1) to provide the pure product **XT26** (30.0 mg, 88.3%) as a white solid. ^{1}H NMR (400 MHz, $CDCl_3$) δ 8.17 (d, *J* = 8.8 Hz, 1H), 6.88 – 6.82 (m, 1H), 6.74 (d, *J* = 2.4 Hz, 1H), 6.42 (d, *J* = 2.3 Hz, 1H), 6.32 (d, *J* = 2.3 Hz, 1H), 5.66 – 5.59 (m, 1H), 4.66 (d, *J* = 6.4 Hz, 2H), 4.08 (t, *J* = 5.9 Hz, 4H), 3.52 – 3.48 (m, 4H), 2.14 – 2.04 (m, 4H), 2.03 – 1.96 (m, 4H), 1.80 (s, 3H), 1.76 (s, 3H). ^{13}C NMR (100 MHz, $CDCl_3$) δ 174.82, 163.69, 163.42, 161.35, 159.90, 156.67, 137.74, 128.38, 119.45, 117.12, 112.86, 107.55, 100.20, 96.49, 93.30, 67.47, 67.42, 66.48, 33.35, 33.31, 29.40, 29.39, 27.75, 27.74, 25.93, 18.50. HRMS (ESI+): calculated for $C_{26}H_{30}Br_2O_5$ $[M + H]^+$ 583.0512, found 583.0496.

1.25 3,6-Bis((6-bromohexyl)oxy)-1-((3-methylbut-2-en-1-yl)oxy)-9*H*-xanthen-9-one (**XT27**).

The target compound was prepared from **XT22** (200.0 mg, 350.68 μmol), Cs_2CO_3 (285.7 mg, 876.70 μmol) and 1-bromo-3-methylbut-2-ene (101.3 μL, 876.70 μmol) according to the similar approach used to synthesize **XT03**. The crude product was purified by silica gel column chromatography (petroleum ether/ethyl acetate = 4/1) to yield the pure product **XT27** (173.4 mg, 77.4%) as a yellow solid. ^{1}H NMR (400 MHz, $CDCl_3$) δ 8.17 (d, *J* = 8.9 Hz, 1H), 6.88 – 6.82 (m, 1H), 6.74 (d, *J* = 2.3 Hz, 1H), 6.42 (d, *J* = 2.3 Hz, 1H), 6.32 (d, *J* = 2.3 Hz, 1H), 5.67 – 5.56 (m, 1H), 4.66 (d, *J* = 6.4 Hz,

2H), 4.04 (t, J = 6.4 Hz, 4H), 3.47 – 3.41 (m, 4H), 1.95 – 1.82 (m, 8H), 1.81 – 1.79 (m, 3H), 1.76 (s, 3H), 1.56 – 1.49 (m, 8H). ^{13}C NMR (100 MHz, $CDCl_3$) δ 174.87, 163.89, 163.64, 161.32, 159.92, 156.70, 137.66, 128.33, 119.51, 117.00, 112.90, 107.45, 100.15, 96.54, 93.29, 68.35, 68.30, 66.46, 33.84 (2×CH_2), 32.70 (2×CH_2), 28.94 (2×CH_2), 27.95 (2×CH_2), 25.93, 25.34 (2×CH_2), 18.49. HRMS (ESI+): calculated for $C_{30}H_{38}Br_2O_5$ [M + H]$^+$ 639.1138, found 639.1134.

1.26 3,6-Bis((8-bromooctyl)oxy)-1-((3-methylbut-2-en-1-yl)oxy)-9*H*-xanthen-9-one (**XT28**).

The target compound was prepared from **XT23** (200.0 mg, 319.27 μmol), Cs_2CO_3 (260.1 mg, 798.18 μmol) and 1-bromo-3-methylbut-2-ene (92.2 μL, 798.18 μmol) according to the similar approach used to synthesize **XT03**. The crude product was purified by silica gel column chromatography (petroleum ether/ethyl acetate = 4/1) to yield the pure product **XT28** (159.8 mg, 72.1%) as a yellow solid. ^{1}H NMR (400 MHz, $CDCl_3$) δ 8.17 (d, J = 8.9 Hz, 1H), 6.91 – 6.81 (m, 1H), 6.74 (d, J = 2.3 Hz, 1H), 6.42 (d, J = 2.3 Hz, 1H), 6.32 (d, J = 2.3 Hz, 1H), 5.68 – 5.58 (m, 1H), 4.66 (d, J = 6.4 Hz, 2H), 4.03 (t, J = 6.5 Hz, 4H), 3.44 – 3.38 (m, 4H), 1.90 – 1.79 (m, 11H), 1.75 (s, 3H), 1.51 – 1.42 (m, 8H), 1.40 – 1.33 (m, 8H). ^{13}C NMR (100 MHz, $CDCl_3$) δ 174.89, 163.96, 163.71, 161.31, 159.93, 156.71, 137.63, 128.29, 119.53, 116.95, 112.92, 107.41, 100.14, 96.54, 93.30, 68.54, 68.49, 66.45, 34.07, 32.84, 32.82, 29.23, 29.21, 29.06, 28.74 (2×CH_2), 28.17, 28.15, 28.14, 25.98, 25.92, 18.49. HRMS (ESI+): calculated for $C_{34}H_{46}Br_2O_5$ [M + H]$^+$ 695.1764, found 695.1762.

1.27 3,6-Bis((12-bromododecyl)oxy)-1-((3-methylbut-2-en-1-yl)oxy)-9*H*-xanthen-9-one (**XT29**).

The target compound was prepared from **XT24** (150.0 mg, 203.08 μmol), Cs_2CO_3 (165.4 mg, 507.69 μmol) and 1-bromo-3-methylbut-2-ene (70.4 μL, 609.23 μmol) according to the similar approach used to synthesize **XT03**. The crude product was purified by silica gel column chromatography (petroleum ether/ethyl acetate = 7/1) to yield the pure product **XT29** (139.0 mg, 84.8%) as a yellow gel. ^{1}H NMR (400 MHz, $CDCl_3$) δ 8.17 (d, J = 9.3 Hz, 1H), 7.01 – 6.69 (m, 2H), 6.37 (d, J = 42.4 Hz, 2H), 5.77 – 5.53 (m, 1H), 4.65 (d, J = 6.7 Hz, 2H), 4.10 – 3.95 (m, 4H), 3.39 (t, J = 9.3 Hz, 4H),

2.04 – 1.70 (m, 14H), 1.51 – 1.11 (m, 32H). ^{13}C NMR (100 MHz, $CDCl_3$) δ 174.85, 163.96, 163.71, 161.28, 159.90, 156.69, 137.56, 128.25, 119.54, 116.91, 112.89, 107.37, 100.11, 96.52, 93.28, 68.60, 68.56, 66.42, 34.14 (2×CH_2), 32.89, 32.88, 29.59 (2×CH_2), 29.57 (2×CH_2), 29.56 (2×CH_2), 29.48 (2×CH_2), 29.40, 29.38, 29.08 (2×CH_2), 28.82 (2×CH_2), 28.22 (2×CH_2), 26.05, 26.03, 25.90, 18.46. HRMS (ESI+): calculated for $C_{42}H_{62}Br_2O_5$ $[M + H]^+$ 807.3016, found 807.3008.

1.28 3,6-Bis(4-(methylamino)butoxy)-1-((3-methylbut-2-en-1-yl)oxy)-9*H*-xanthen-9-one (**XT31**).

The target compound was prepared from **XT26** (26.8 mg, 46.02 μmol) and methanamine (1 mL) according to the similar approach used to synthesize **XT08**. The product **XT31** (14.0 mg) was obtained in 63.0% yield as a yellow gel. ^{1}H NMR (400 MHz, CD_3OD) δ 8.07 (d, *J* = 8.8 Hz, 1H), 7.00 – 6.88 (m, 2H), 6.63 – 6.28 (m, 2H), 5.59 – 5.51 (m, 1H), 4.71 (d, *J* = 6.3 Hz, 2H), 4.16 (t, *J* = 5.8 Hz, 4H), 3.14 – 3.04 (m, 4H), 2.72 (d, *J* = 1.3 Hz, 6H), 1.92 (s, 8H), 1.81 (s, 3H), 1.80 (s, 3H). ^{13}C NMR (100 MHz, CD_3OD) δ 176.68, 165.72, 165.37, 162.34, 161.24, 158.21, 138.83, 128.78, 120.75, 117.55, 114.37, 107.90, 101.35, 97.76, 94.74, 68.98, 68.92, 67.33, 50.04, 50.02, 33.55 (2×CH_3), 27.09, 27.08, 25.93, 24.11, 24.08, 18.42. HRMS (ESI+): calculated for $C_{28}H_{38}N_2O_5$ $[M + H]^+$ 483.2853, found 483.2839.

1.29 3,6-Bis(4-(diethylamino)butoxy)-1-((3-methylbut-2-en-1-yl)oxy)-9*H*-xanthen-9-one (**XT32**).

The target compound was prepared from **XT26** (60.0 mg, 103.03 μmol) and diethylamine (1 mL) according to the similar approach used to synthesize **XT08**. The product **XT32** (41.4 mg) was obtained in 70.9% yield as a yellow gel. ^{1}H NMR (400 MHz, CD_3OD) δ 8.09 (d, *J* = 8.7 Hz, 1H), 7.04 – 6.92 (m, 2H), 6.66 – 6.32 (m, 2H), 5.56 (t, *J* = 6.3 Hz, 1H), 4.72 (d, *J* = 6.3 Hz, 2H), 4.23 – 4.15 (m, 4H), 3.28 – 3.21 (m, 12H), 1.97 – 1.90 (m, 8H), 1.89 – 1.74 (m, 6H), 1.33 (t, *J* = 7.2 Hz, 12H). ^{13}C NMR (100 MHz, CD_3OD) δ 170.24, 165.75, 165.38, 162.38, 161.27, 158.24, 138.81, 128.80, 120.73, 117.58, 114.37, 107.92, 101.37, 97.78, 94.75, 69.00, 68.95, 67.32, 52.63, 52.62, 48.20 (4×CH_2), 27.25, 25.92 (2×CH_2), 22.05, 22.03, 18.40, 9.30 (2×CH_3), 9.27 (2×CH_3). HRMS (ESI+): calculated for $C_{34}H_{50}N_2O_5$ $[M + H]^+$ 567.3792, found 567.3776.

1.30 3,6-Bis(4-aminobutoxy)-1-((3-methylbut-2-en-1-yl)oxy)-9*H*-xanthen-9-one (**XT33**).

The target compound was prepared from **XT26** (60.0 mg, 103.03 μmol) and ammonia (2 mL) according to the similar approach used to synthesize **XT07**. The product **XT33** (19.3 mg) was obtained in 41.2% yield as a white solid. ^{1}H NMR (400 MHz, CD_3OD) δ 7.95 (d, *J* = 9.4 Hz, 1H), 7.01 – 6.90 (m, 2H), 6.65 – 6.44 (m, 2H), 5.48 (t, *J* = 6.5 Hz, 1H), 4.63 (d, *J* = 6.5 Hz, 2H), 4.13 (t, *J* = 5.6 Hz, 4H), 2.83 (t, *J* = 7.1 Hz, 4H), 1.86 – 1.66 (m, 14H). ^{13}C NMR (100 MHz, CD_3OD) δ 182.10, 175.19, 172.46, 169.91, 168.37, 165.31, 146.21, 136.70, 128.95, 125.46, 122.43, 115.60, 109.62, 105.80, 102.86, 77.16, 77.11, 75.11, 54.14, 54.12, 34.86, 34.81 (2×CH_2), 33.55, 27.45 (2×CH_3). HRMS (ESI+): calculated for $C_{26}H_{34}N_2O_5$ $[M + H]^+$ 455.2540, found 455.2532.

1.31 3,6-Bis((6-(methylamino)hexyl)oxy)-1-((3-methylbut-2-en-1-yl)oxy)-9*H*-xanthen-9-one (**XT34**).

The target compound was prepared from **XT27** (43.4 mg, 67.98 μmol) and methanamine (1 mL) according to the similar approach used to synthesize **XT08** obtaining product **XT34** (29.0 mg) in 79.2% yield as a brown gel. ^{1}H NMR (400 MHz, CD_3OD) δ 8.07 – 7.95 (m, 1H), 6.97 – 6.78 (m, 2H), 6.50 (s, 1H), 6.36 (s, 1H), 5.52 (t, *J* = 6.3 Hz, 1H), 4.68 (d, *J* = 6.3 Hz, 2H), 4.14 – 3.99 (m, 4H), 3.04 – 2.92 (m, 4H), 2.84 – 2.60 (m, 6H), 1.89 – 1.60 (m, 14H), 1.59 – 1.41 (m, 8H). ^{13}C NMR (100 MHz, CD_3OD) δ 169.88, 165.93, 165.59, 162.24, 161.20, 158.19, 138.64, 128.66, 120.84, 117.35, 114.36, 107.72, 101.17, 97.74, 94.61, 69.58, 69.52, 67.28, 50.25 (2×CH_2), 33.49 (2×CH_3), 30.04, 29.87, 27.52, 27.25, 27.23, 27.11 (2×CH_2), 26.63, 25.93, 18.42. HRMS (ESI+): calculated for $C_{32}H_{46}N_2O_5$ $[M + H]^+$ 539.3479, found 539.3475.

1.32 3,6-Bis((6-(diethylamino)hexyl)oxy)-1-((3-methylbut-2-en-1-yl)oxy)-9*H*-xanthen-9-one (**XT35**).

The target compound was prepared from **XT27** (43.4 mg, 67.98 μmol) and diethylamine (1 mL) according to the similar approach used to synthesize **XT08** obtaining product **XT35** (32.9 mg) in 77.7% yield as a brown gel. ^{1}H NMR (400 MHz, CD_3OD) δ 8.06 (d, *J* = 8.9 Hz, 1H), 7.01 – 6.81 (m, 2H), 6.54 (d, *J* = 2.1 Hz, 1H), 6.40 (d, *J* = 2.2 Hz, 1H), 5.59 – 5.51 (m, 1H), 4.71 (d, *J* = 6.4 Hz, 2H), 4.19 – 4.03 (m, 4H),

3.20 – 3.13 (m, 8H), 3.10 – 3.03 (m, 4H), 1.91 – 1.82 (m, 4H), 1.82 – 1.78 (m, 6H), 1.77 – 1.69 (m, 4H), 1.63 – 1.54 (m, 4H), 1.53 – 1.44 (m, 4H), 1.29 (t, *J* = 7.3 Hz, 12H). ^{13}C NMR (100 MHz, CD_3OD) δ 176.73, 166.02, 165.67, 162.33, 161.29, 158.28, 138.72, 128.72, 120.83, 117.40, 114.41, 107.78, 101.24, 97.80, 94.68, 69.61, 69.56, 67.30, 52.99 (2×CH_2), 48.18 (4×CH_2), 29.92 (2×CH_2), 27.52, 27.49, 26.73, 26.72, 25.94, 25.10, 25.08, 18.42, 9.30 (2×CH_3), 9.28 (2×CH_3). HRMS (ESI+): calculated for $C_{38}H_{58}N_2O_5$ $[M + H]^+$ 623.4418, found 623.4403.

1.33 3,6-Bis((8-(methylamino)octyl)oxy)-1-((3-methylbut-2-en-1-yl)oxy)-9*H*-xanthen-9-one (**XT37**).

The target compound was prepared from **XT28** (41.7 mg, 60.04 μmol) and methanamine (1 mL) according to the similar approach used to synthesize **XT08**. The product **XT37** (30.1 mg) was obtained in 84.3% yield as a brown gel. ^{1}H NMR (400 MHz, CD_3OD) δ 8.08 – 8.02 (m, 1H), 6.96 – 6.84 (m, 2H), 6.53 (d, *J* = 2.2 Hz, 1H), 6.39 (d, *J* = 2.2 Hz, 1H), 5.55 (t, *J* = 6.4 Hz, 1H), 4.71 (d, *J* = 6.3 Hz, 2H), 4.13 – 4.02 (m, 4H), 3.04 – 2.92 (m, 4H), 2.85 – 2.63 (m, 6H), 1.85 – 1.78 (m, 10H), 1.72 – 1.49 (m, 8H), 1.42 (s, 12H). ^{13}C NMR (100 MHz, CD_3OD) δ 176.77, 166.05, 165.72, 162.30, 161.28, 158.27, 138.69, 128.69, 120.84, 117.34, 114.39, 107.73, 101.18, 97.79, 94.65, 69.80, 69.74, 67.28, 50.36 (2×CH_2), 33.49 (2×CH_3), 30.24, 30.21, 30.17, 30.15, 30.11, 27.46, 27.43, 27.17, 27.16, 27.00 (2×CH_2), 25.92 (2×CH_2), 18.41. HRMS (ESI+): calculated for $C_{36}H_{54}N_2O_5$ $[M + H]^+$ 595.4105, found 595.4099.

1.34 3,6-Bis((8-(diethylamino)octyl)oxy)-1-((3-methylbut-2-en-1-yl)oxy)-9*H*-xanthen-9-one (**XT38**).

The target compound was prepared from **XT28** (41.7 mg, 60.04 μmol) and diethylamine (1 mL) according to the similar approach used to synthesize **XT08**. The product **XT38** (33.9 mg) was obtained in 83.1% yield as a brown gel. ^{1}H NMR (400 MHz, CD_3OD) δ 8.05 (d, *J* = 8.9 Hz, 1H), 6.97 – 6.81 (m, 2H), 6.53 (d, *J* = 2.0 Hz, 1H), 6.39 (d, *J* = 2.1 Hz, 1H), 5.58 – 5.51 (m, 1H), 4.71 (d, *J* = 6.3 Hz, 2H), 4.15 – 4.00 (m, 4H), 3.19 – 3.11 (m, 8H), 3.07 – 3.00 (m, 4H), 1.86 – 1.76 (m, 10H), 1.73 – 1.47 (m, 8H), 1.46 – 1.38 (m, 12H), 1.28 (t, *J* = 7.3 Hz, 12H). ^{13}C NMR (100 MHz, CD_3OD) δ 176.72, 166.04, 165.70, 162.30, 161.27, 158.26, 138.65, 128.68, 120.86, 117.35, 114.40,

107.73, 101.19, 97.78, 94.65, 69.79, 69.74, 67.28, 53.07 (2×CH_2), 48.16 (4×CH_2), 30.28, 30.24, 30.23, 30.20, 30.09 (2×CH_2), 27.73, 27.70, 26.98 (2×CH_2), 25.93, 25.14, 25.11, 18.42, 9.29 (2×CH_3), 9.27 (2×CH_3). HRMS (ESI+): calculated for $C_{42}H_{66}N_2O_5$ $[M + H]^+$ 679.5044, found 679.5032.

1.35 3,6-Bis((12-(methylamino)dodecyl)oxy)-1-((3-methylbut-2-en-1-yl)oxy)-9*H*-xanthen-9-one (**XT40**).

The target compound was prepared from **XT29** (50.0 mg, 61.98 μmol) and methanamine (1 mL) according to the similar approach used to synthesize **XT08**. The product **XT40** (41.7 mg) was obtained in 95.2% yield as a brown gel. ^1H NMR (400 MHz, CD_3OD) δ 7.96 (d, J = 8.9 Hz, 1H), 6.85 – 6.77 (m, 1H), 6.76 – 6.69 (m, 1H), 6.41 – 6.32 (m, 1H), 6.30 – 6.24 (m, 1H), 5.53 – 5.45 (m, 1H), 4.64 (d, J = 6.2 Hz, 2H), 4.03 – 3.87 (m, 4H), 2.92 (t, J = 7.7 Hz, 4H), 2.80 – 2.59 (m, 6H), 1.80 – 1.70 (m, 10H), 1.66 – 1.55 (m, 4H), 1.48 – 1.39 (m, 4H), 1.35 – 1.24 (m, 28H). ^{13}C NMR (100 MHz, CD_3OD) δ 169.97, 165.90, 165.58, 162.18, 161.14, 158.14, 138.44, 128.61, 120.98, 117.30, 114.34, 107.68, 101.12, 97.72, 94.60, 69.81, 69.73, 67.29, 50.34 (2×CH_2), 33.47 (2×CH_3), 30.73, 30.71 (2×CH_2), 30.69, 30.67 (2×CH_2), 30.65, 30.54, 30.52 (2×CH_2), 30.27, 30.25, 30.18, 30.14, 27.52, 27.51, 27.17 (2×CH_2), 27.11, 27.09, 25.97, 18.48. HRMS (ESI+): calculated for $C_{44}H_{70}N_2O_5$ $[M + H]^+$ 707.5357, found 707.5348.

1.36 3,6-Bis((12-(diethylamino)dodecyl)oxy)-1-((3-methylbut-2-en-1-yl)oxy)-9*H*-xanthen-9-one (**XT41**).

The target compound was prepared from **XT29** (50.0 mg, 61.98 μmol) and diethylamine (1 mL) according to the similar approach used to synthesize **XT08**. The product **XT41** (40.8 mg) was obtained in 83.2% yield as a brown gel. ^1H NMR (400 MHz, CD_3OD) δ 8.03 (d, J = 8.9 Hz, 1H), 6.97 – 6.74 (m, 2H), 6.47 (s, 1H), 6.36 (s, 1H), 5.54 (t, J = 6.1 Hz, 1H), 4.69 (d, J = 6.2 Hz, 2H), 4.15 – 3.93 (m, 4H), 2.92 – 2.79 (m, 8H), 2.78 – 2.67 (m, 4H), 1.88 – 1.71 (m, 10H), 1.62 – 1.44 (m, 8H), 1.41 – 1.26 (m, 28H), 1.16 (t, J = 7.2 Hz, 12H). ^{13}C NMR (100 MHz, CD_3OD) δ 176.48, 165.84, 165.52, 162.19, 161.10, 158.09, 138.32, 128.63, 121.03, 117.33, 114.31, 107.69, 101.11, 97.66, 94.57, 69.80, 69.73, 67.27, 53.39 (2×CH_2), 47.92 (4×CH_2), 30.72 (4×CH_2), 30.69 (2×CH_2), 30.67 (2×CH_2), 30.52 (2×CH_2), 30.50 (2×CH_2), 30.19, 30.15, 28.28,

28.26, 27.11, 27.09, 26.04, 26.02, 26.00, 18.51, 10.18 (2×CH_3), 10.16 (2×CH_3). HRMS (ESI+): calculated for $C_{50}H_{82}N_2O_5$ $[M + H]^+$ 791.6297, found 791.6279.

1.37 1,1'-(((1-((3-Methylbut-2-en-1-yl)oxy)-9-oxo-9*H*-xanthene-3,6-diyl)bis(oxy))bis(ethane-2,1-diyl))diguanidine (**XT42**).

The target compound was prepared from **XT25** (50.0 mg, 95.02 μmol) and ammonia (3 mL) according to the similar approach used to synthesize **XT17**. The product **XT42** (13.8 mg) was obtained in 30.1% yield as a yellow solid. ^{1}H NMR (400 MHz, DMSO-d6) δ 8.79 (d, J = 35.1 Hz, 2H), 8.01 – 7.78 (m, 7H), 7.02 – 6.92 (m, 2H), 6.59 (d, J = 2.1 Hz, 1H), 6.48 (d, J = 2.1 Hz, 1H), 5.49 – 5.41 (m, 1H), 4.60 (d, J = 6.6 Hz, 2H), 4.21 – 4.13 (m, 4H), 3.53 – 3.48 (m, 4H), 1.74 (s, 3H), 1.70 (s, 3H). ^{13}C NMR (100 MHz, DMSO-d6) δ 173.34, 167.68, 163.69, 163.30, 161.17, 159.55, 158.30, 158.26, 156.48, 137.56, 128.03, 120.07, 116.95, 107.05, 101.11, 97.10, 94.30, 67.28, 67.25, 66.37, 40.70 (2×CH_2), 26.03, 18.67. HRMS (ESI+): calculated for $C_{24}H_{30}N_6O_5$ $[M + H]^+$ 483.2350, found 483.2342.

1.38 1,1'-(((1-((3-Methylbut-2-en-1-yl)oxy)-9-oxo-9*H*-xanthene-3,6-diyl)bis(oxy))bis(butane-4,1-diyl))diguanidine (**XT43**).

The target compound was prepared from **XT26** (94.8 mg, 162.79 μmol) and ammonia (3 mL) according to the similar approach used to synthesize **XT17**. The product **XT43** (31.0 mg) was obtained in 35.5% yield as a white solid. ^{1}H NMR (400 MHz, DMSO-d6) δ 8.95 (d, J = 34.2 Hz, 2H), 7.97 – 7.75 (m, 7H), 7.00 – 6.91 (m, 2H), 6.59 (s, 1H), 6.49 (d, J = 1.8 Hz, 1H), 5.53 – 5.42 (m, 1H), 4.62 (d, J = 6.5 Hz, 2H), 4.12 (t, J = 6.0 Hz, 4H), 3.17 – 3.11 (m, 4H), 1.80 – 1.72 (m, 10H), 1.67 – 1.59 (m, 4H). ^{13}C NMR (100 MHz, DMSO-d6) δ 173.35, 167.89, 164.12, 163.74, 161.12, 159.62, 157.94, 156.55, 137.43, 127.91, 120.16, 116.66, 106.80, 100.84, 100.00, 97.09, 94.02, 68.46, 68.41, 66.33, 40.70 (2×CH_2), 26.22, 26.17, 26.03 (2×CH_2), 25.68, 18.66. HRMS (ESI+): calculated for $C_{28}H_{38}N_6O_5$ $[M + H]^+$ 539.2976, found 539.2966.

1.39 1,1'-(((1-((3-Methylbut-2-en-1-yl)oxy)-9-oxo-9*H*-xanthene-3,6-diyl)bis(oxy))bis(hexane-6,1-diyl))diguanidine (**XT44**).

The target compound was prepared from **XT27** (97.7 mg, 153.03 μmol) and ammonia (3 mL) according to the similar approach used to synthesize **XT17**. The product **XT44**

(25.7 mg) was obtained in 28.2% yield as a brown gel. ^{1}H NMR (400 MHz, DMSO-d6) δ 8.77 (d, *J* = 25.0 Hz, 2H), 7.94 (d, *J* = 9.1 Hz, 1H), 7.86 – 7.65 (m, 6H), 6.95 (d, *J* = 7.9 Hz, 2H), 6.58 (s, 1H), 6.49 (s, 1H), 5.49 (t, *J* = 6.0 Hz, 1H), 4.63 (d, *J* = 6.3 Hz, 2H), 4.10 (t, *J* = 6.0 Hz, 4H), 3.12 – 2.99 (m, 4H), 1.85 – 1.64 (m, 10H), 1.54 – 1.30 (m, 12H). ^{13}C NMR (100 MHz,) δ 173.30, 167.70, 164.14, 163.77, 161.06, 159.56, 157.90, 156.50, 137.30, 127.84, 120.16, 116.57, 113.58, 106.73, 100.72, 97.01, 93.92, 68.75, 66.29, 49.07, 41.00 (2×CH_2), 28.87, 28.83, 28.80, 26.33, 26.30, 25.97 (2×CH_2), 25.56, 25.49, 18.61. HRMS (ESI+): calculated for $C_{32}H_{46}N_6O_5$ $[M + H]^+$ 595.3602, found 595.3585.

1.40 1,1'-(((1-((3-Methylbut-2-en-1-yl)oxy)-9-oxo-9*H*-xanthene-3,6-diyl)bis(oxy))bis(octane-8,1-diyl))diguanidine (**XT45**).

The target compound was prepared from **XT28** (89.0 mg, 128.14 μmol) and ammonia (3 mL) according to the similar approach used to synthesize **XT17**. The product **XT45** (22.7 mg) was obtained in 27.2% yield as a brown gel. ^{1}H NMR (400 MHz, DMSO-d6) δ 8.73 (d, *J* = 18.2 Hz, 2H), 7.94 (d, *J* = 9.3 Hz, 1H), 7.76 (br, 6H), 6.95 (d, *J* = 7.5 Hz, 2H), 6.60 – 6.42 (m, 2H), 5.48 (t, *J* = 6.3 Hz, 1H), 4.63 (d, *J* = 6.3 Hz, 2H), 4.09 (t, *J* = 6.2 Hz, 4H), 3.10 – 2.96 (m, 4H), 1.83 – 1.66 (m, 10H), 1.50 – 1.24 (m, 20H). ^{13}C NMR (100 MHz, DMSO-d6) δ 173.30, 167.66, 164.16, 163.78, 161.06, 159.57, 157.89, 157.87, 156.51, 137.29, 127.84, 120.17, 116.56, 106.72, 100.71, 100.00, 93.92, 68.84, 66.28, 49.07, 41.02 (2×CH_2), 29.19, 29.15, 29.09, 29.06, 28.93, 28.89, 28.85, 26.58, 26.55, 25.97, 25.90, 25.88, 25.85, 18.61. HRMS (ESI+): calculated for $C_{36}H_{54}N_6O_5$ $[M + H]^+$ 651.4228, found 651.4217.

2. ^{1}H and ^{13}C NMR Spectra of xanthone derivatives

^{1}H-NMR spectrum of **XT01**

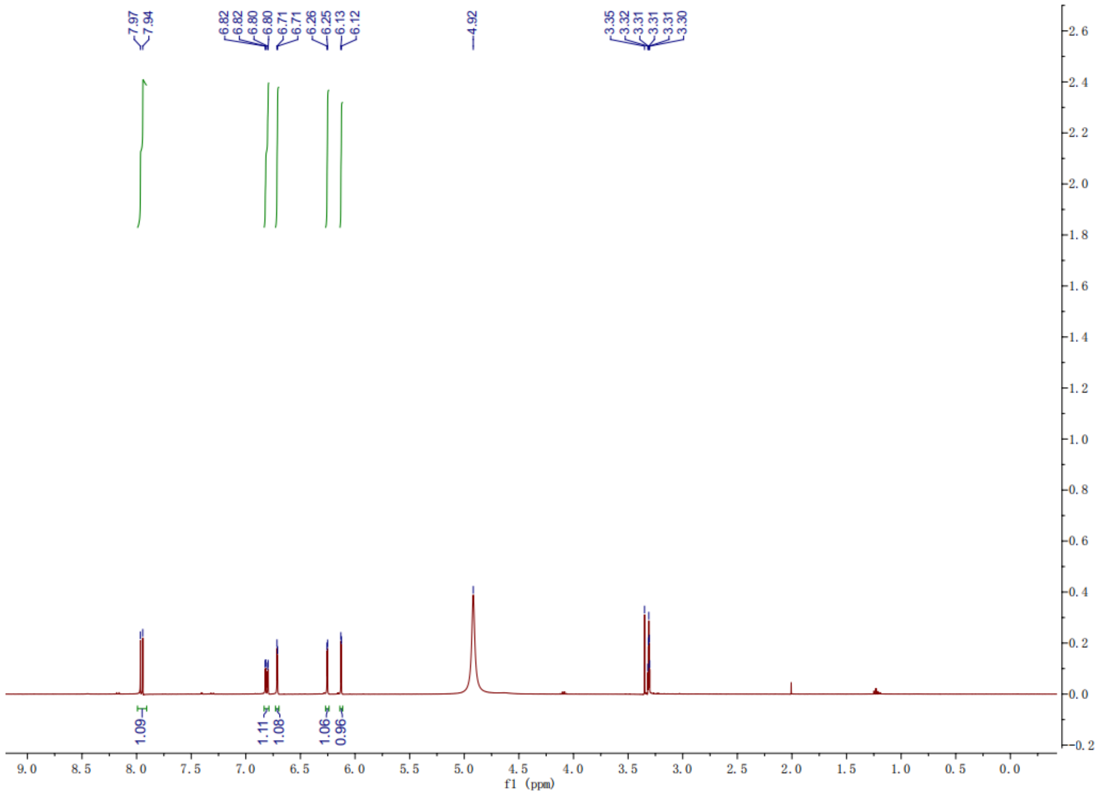

^{13}C-NMR spectrum of **XT01**

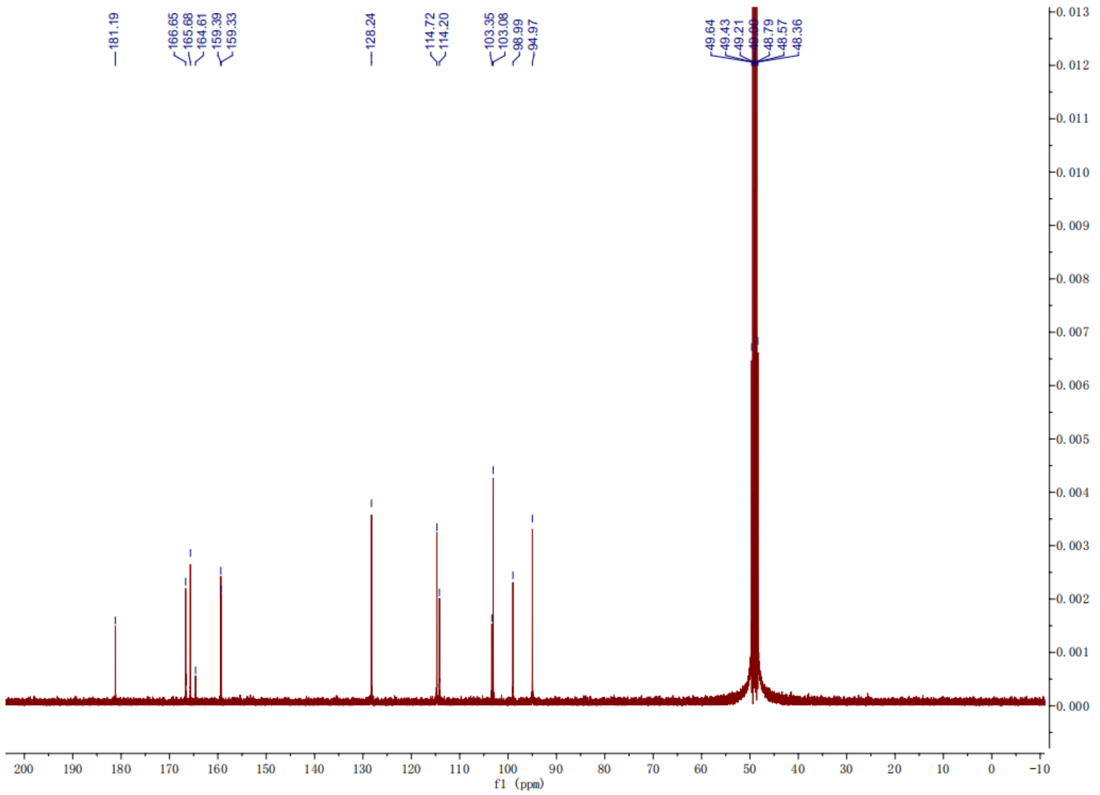

^{1}H-NMR spectrum of **XT02**

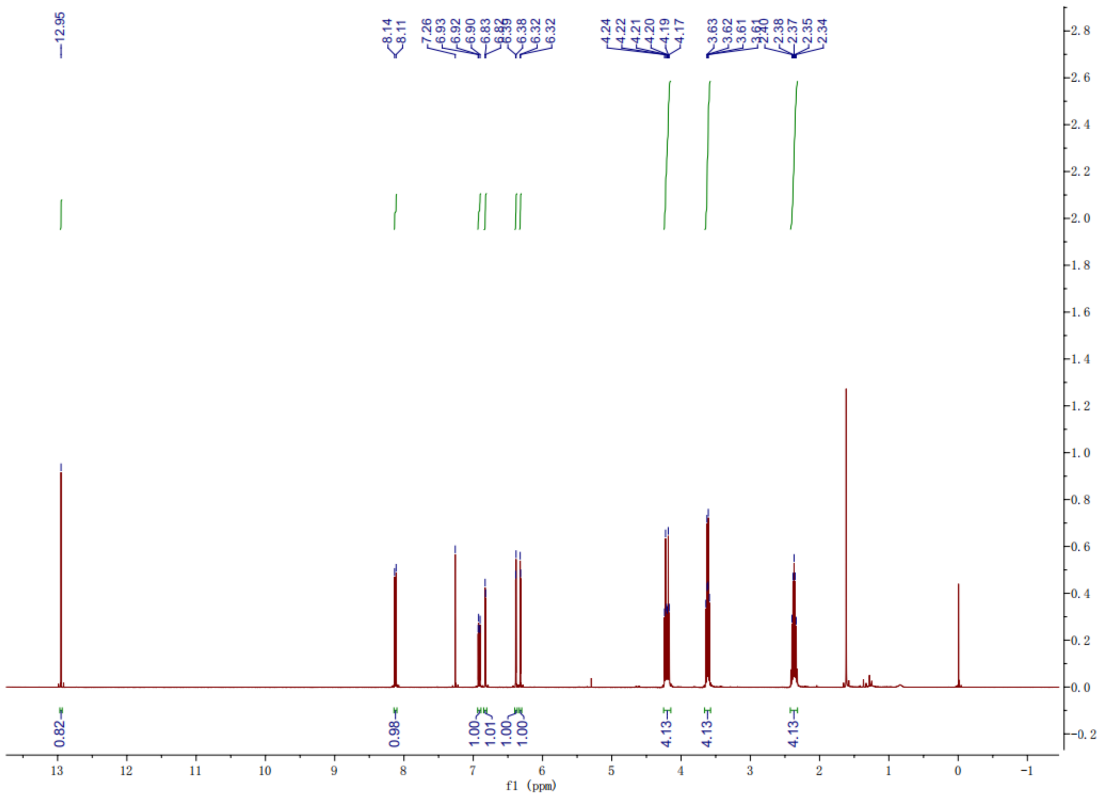

^{13}C-NMR spectrum of **XT02**

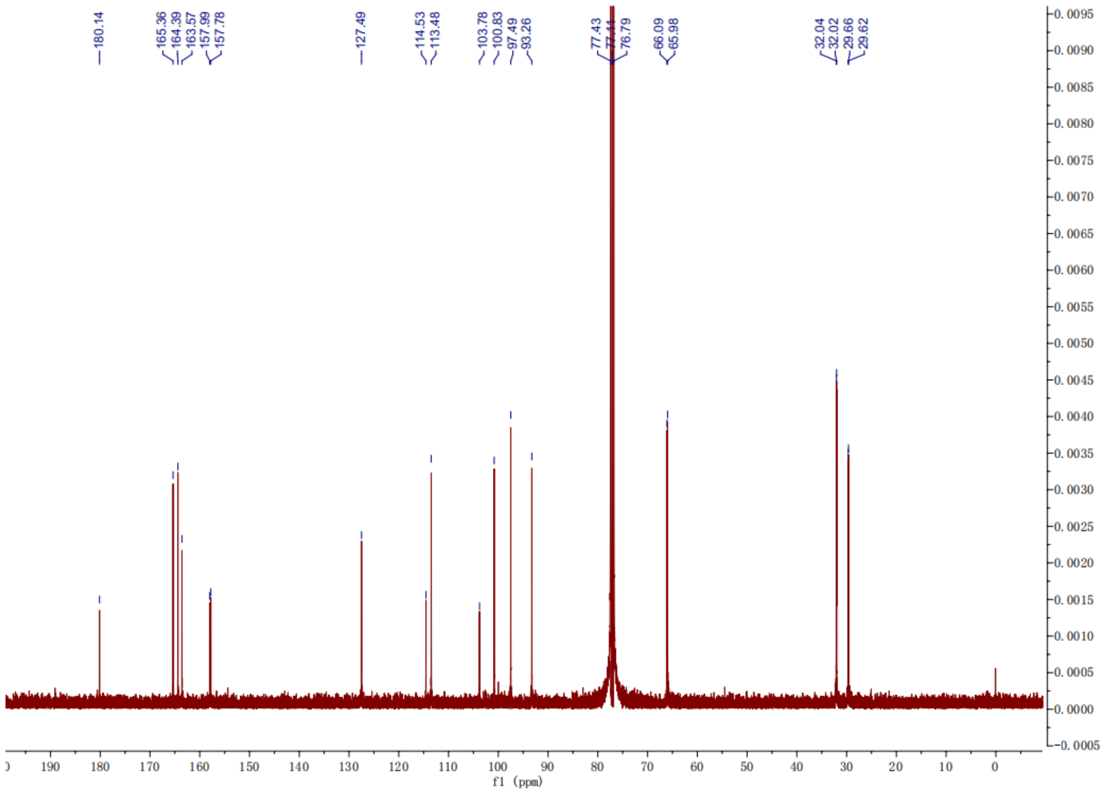

^{1}H-NMR spectrum of **XT03**

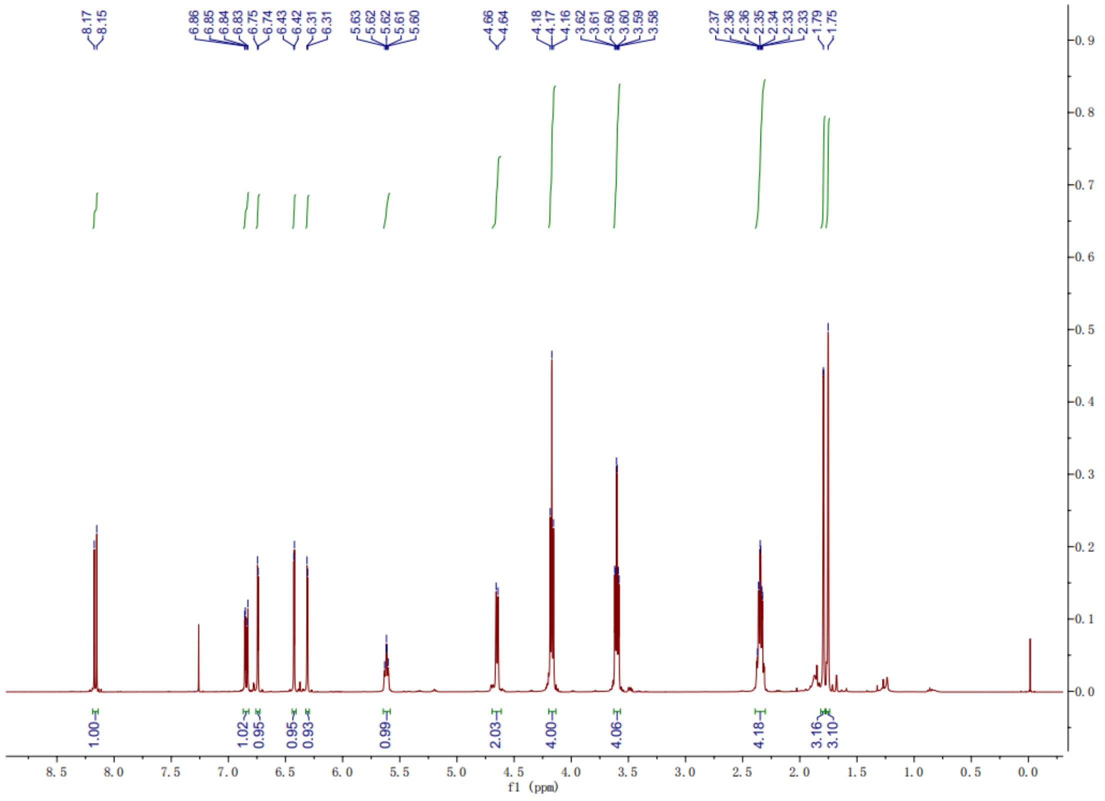

^{13}C-NMR spectrum of **XT03**

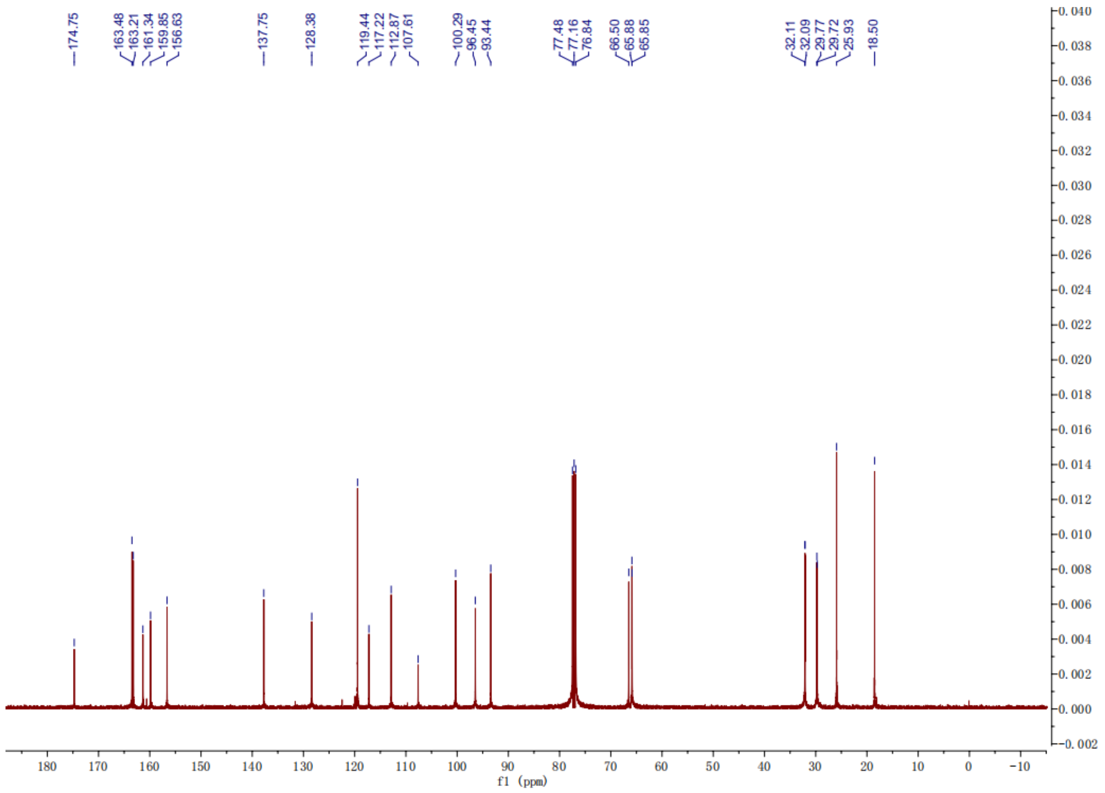

^{1}H-NMR spectrum of **XT04**

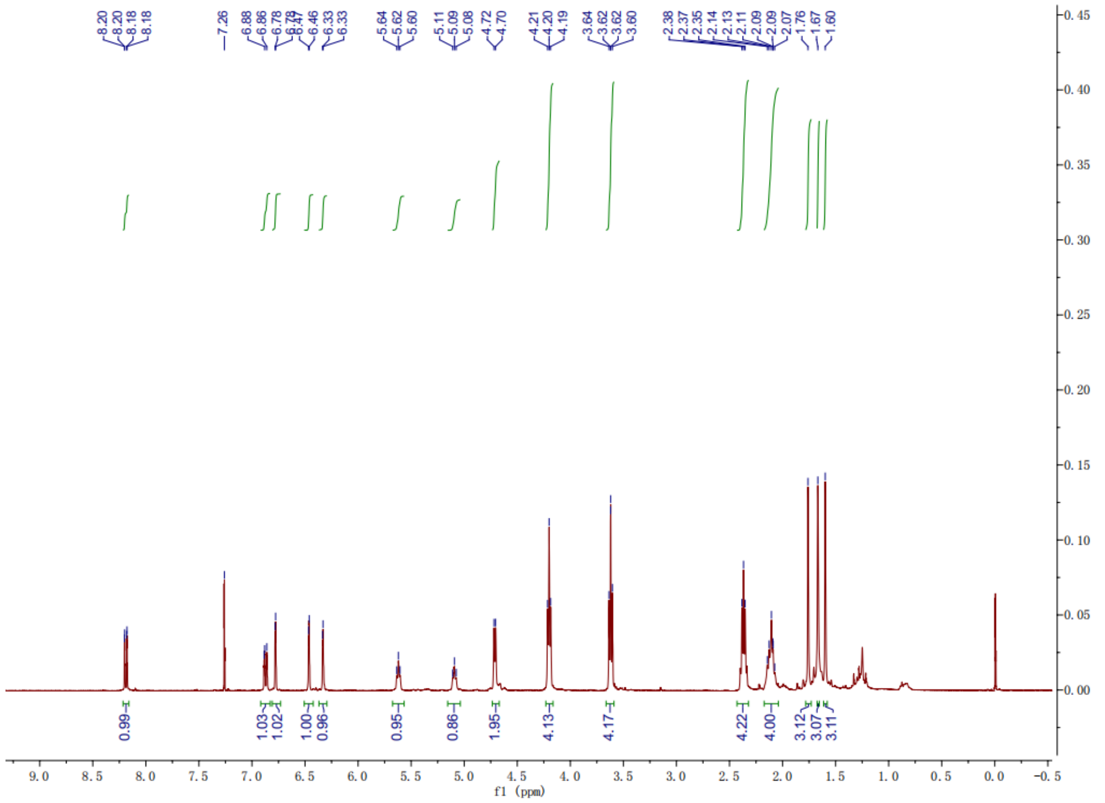

^{13}C-NMR spectrum of **XT04**

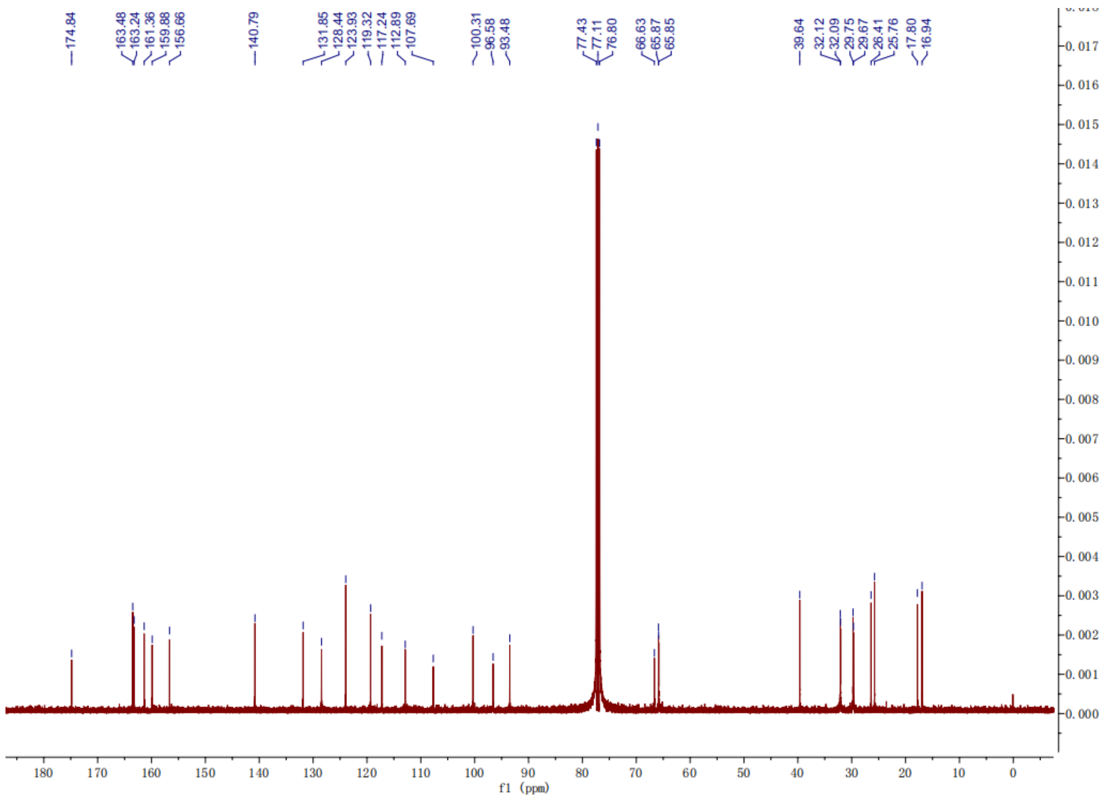

^{1}H-NMR spectrum of **XT05**

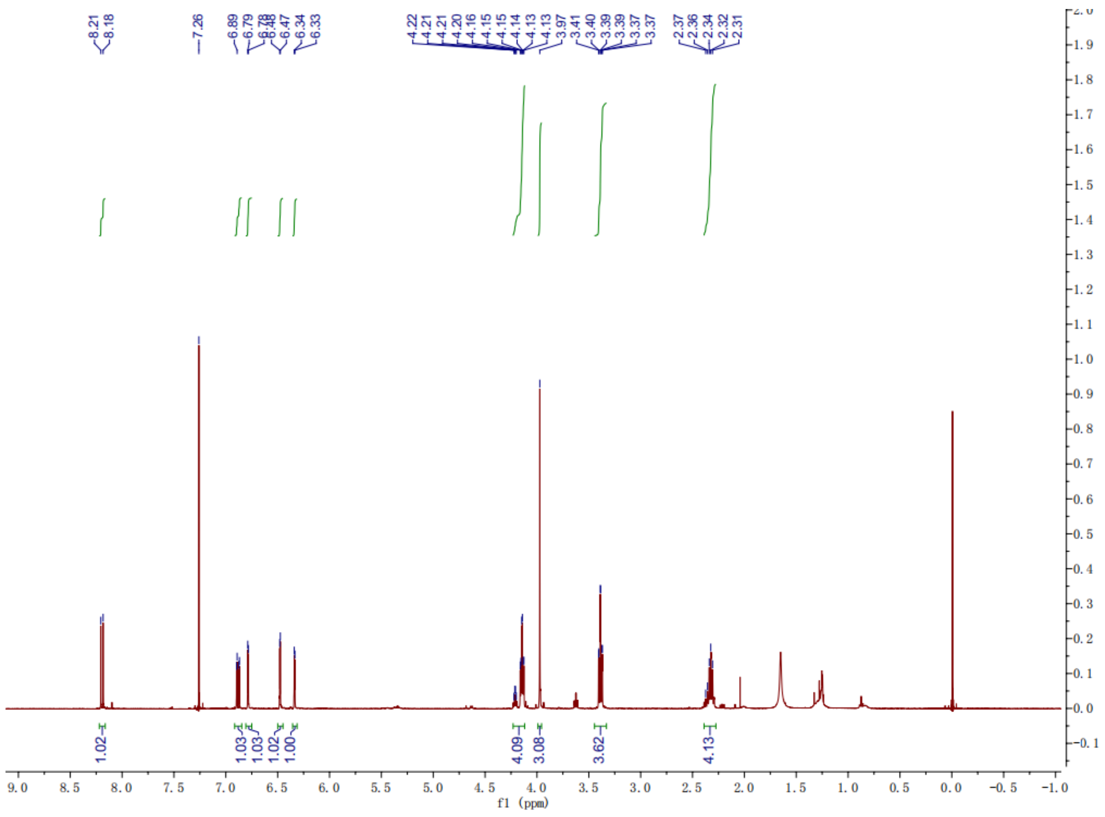

^{13}C-NMR spectrum of **XT05**

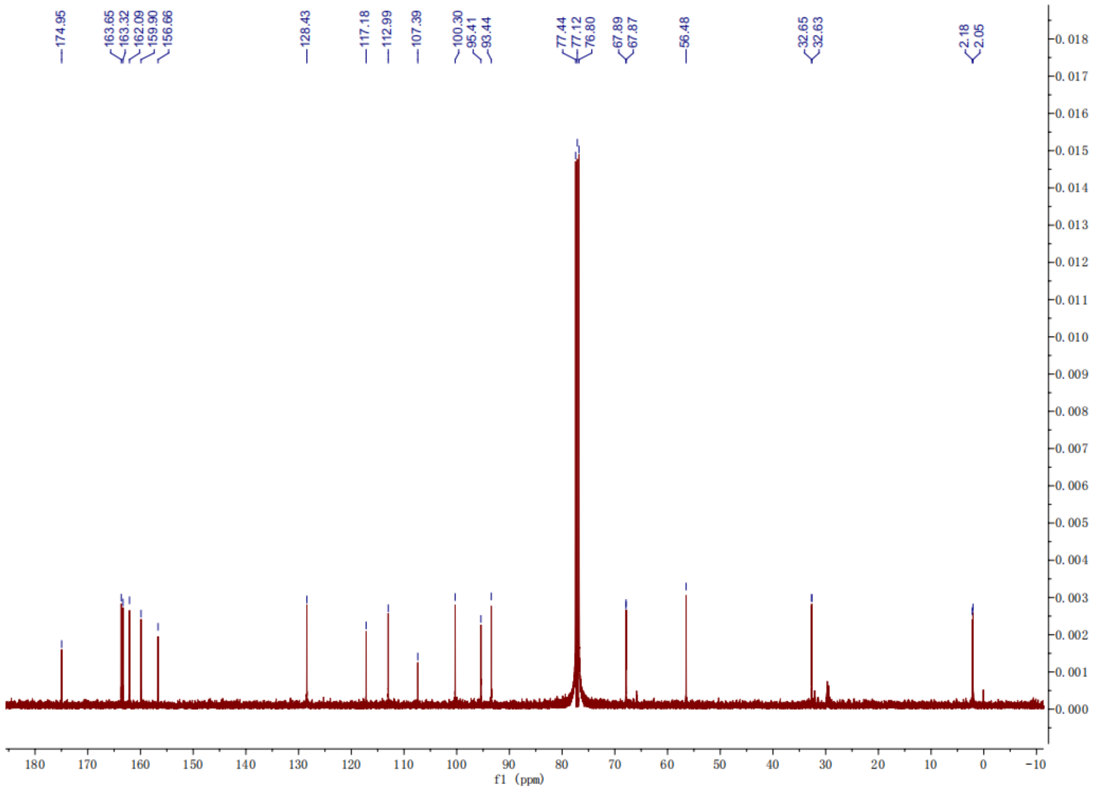

^{1}H-NMR spectrum of **XT06**

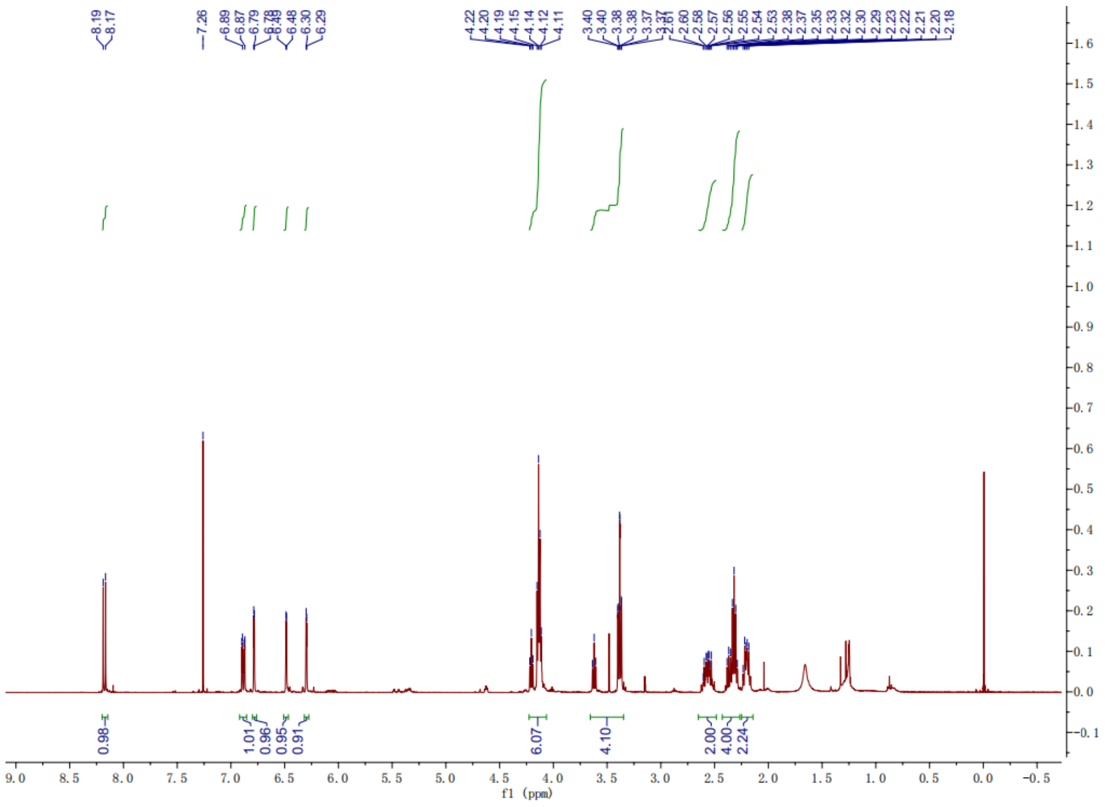

^{13}C-NMR spectrum of **XT06**

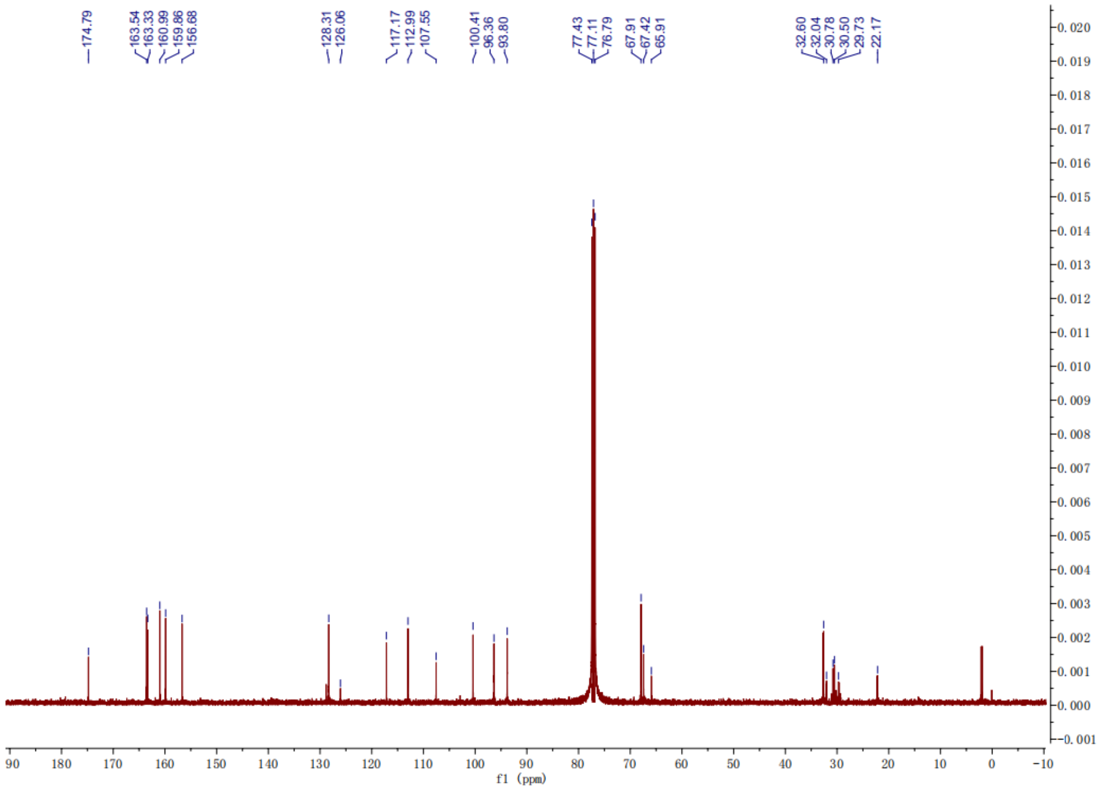

^{1}H-NMR spectrum of **XT07**

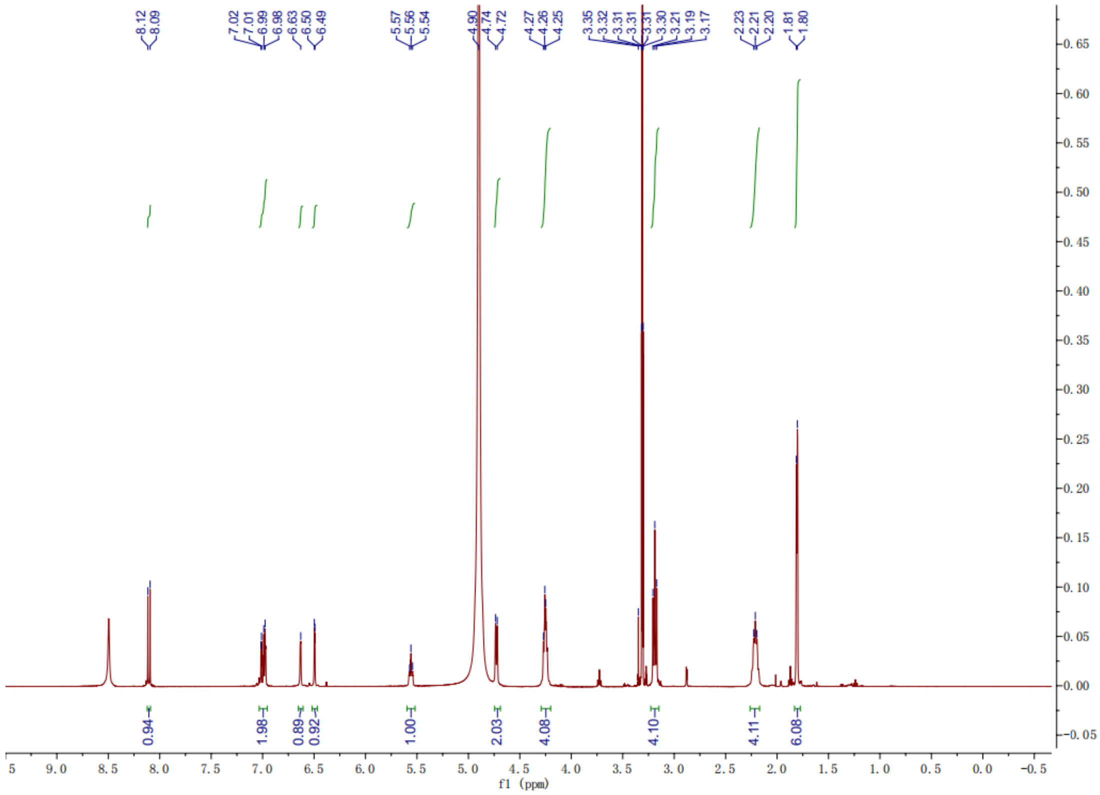

^{13}C-NMR spectrum of **XT07**

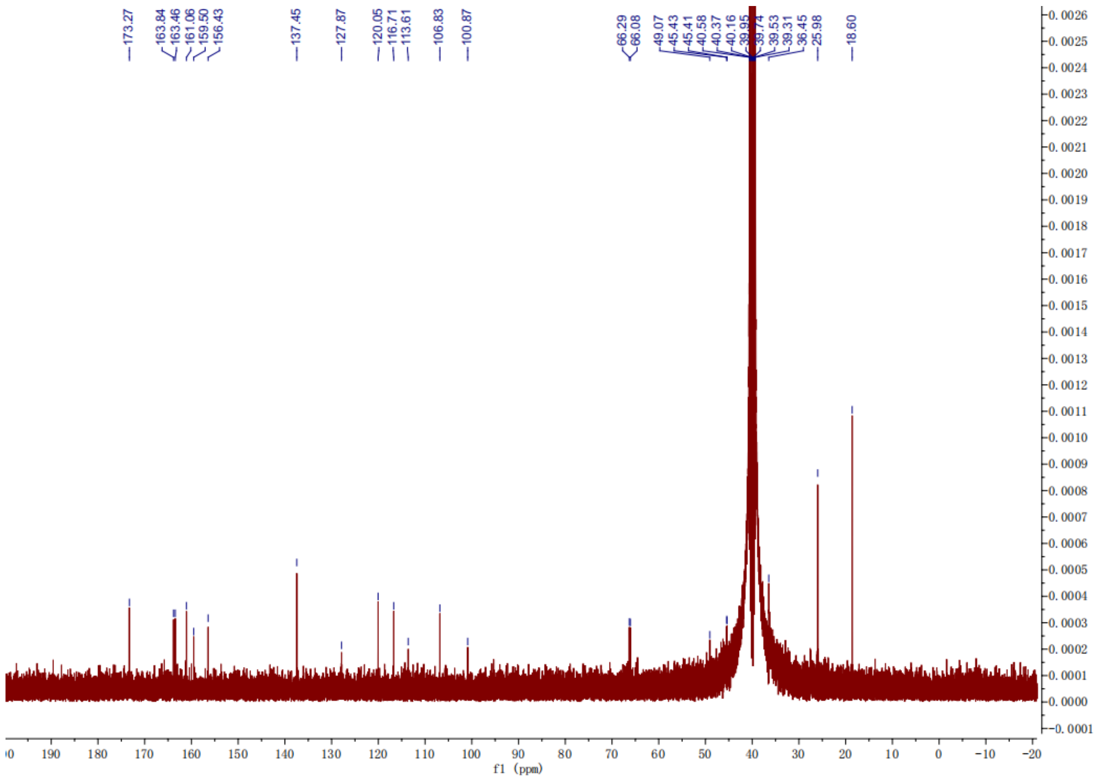

^{1}H-NMR spectrum of **XT08**

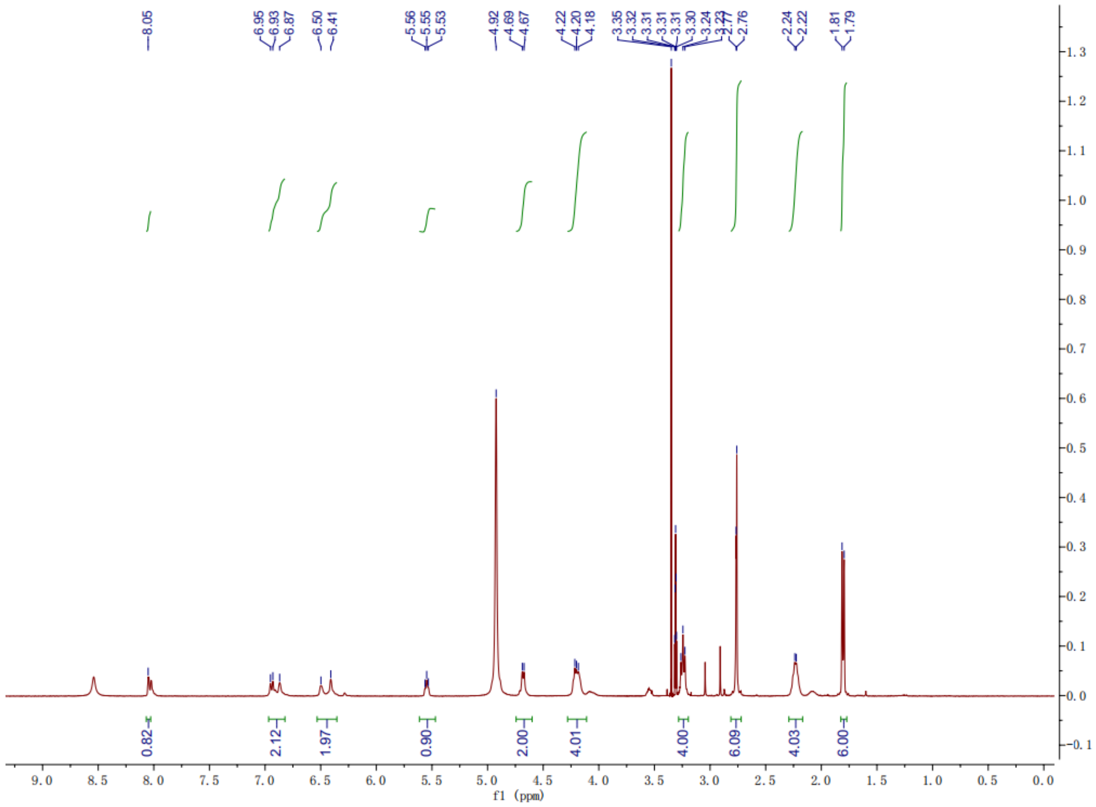

^{13}C-NMR spectrum of **XT08**

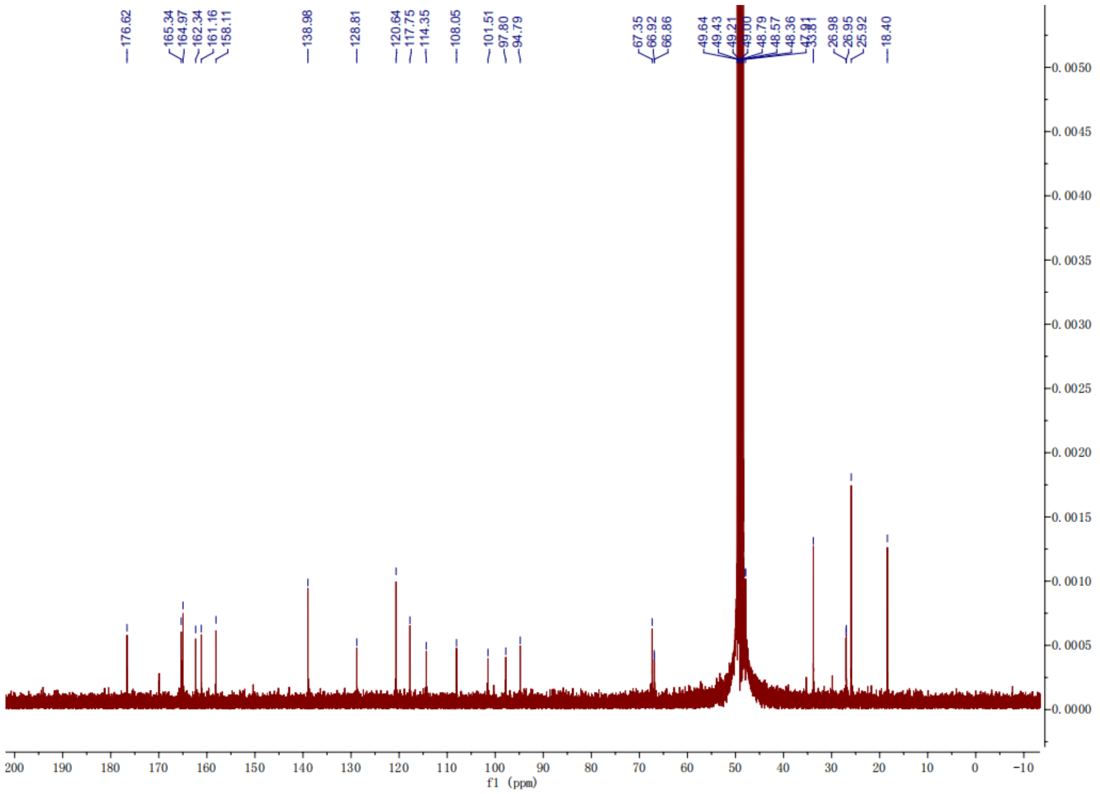

^{1}H-NMR spectrum of **XT09**

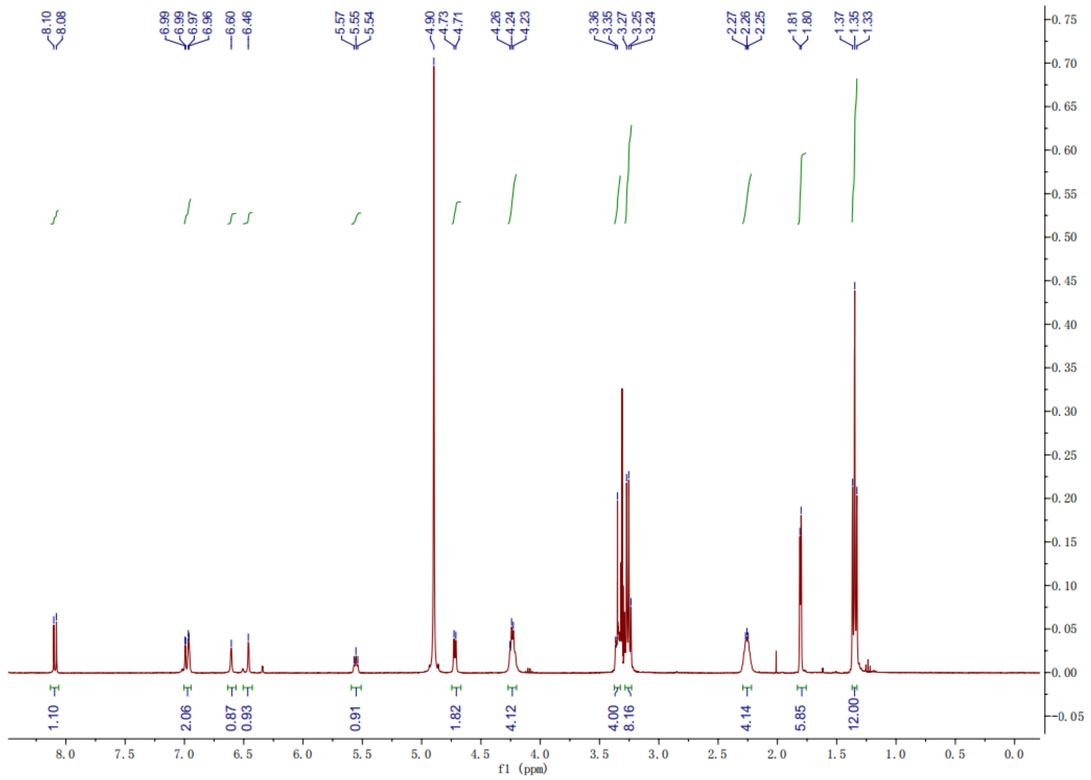

^{13}C-NMR spectrum of **XT09**

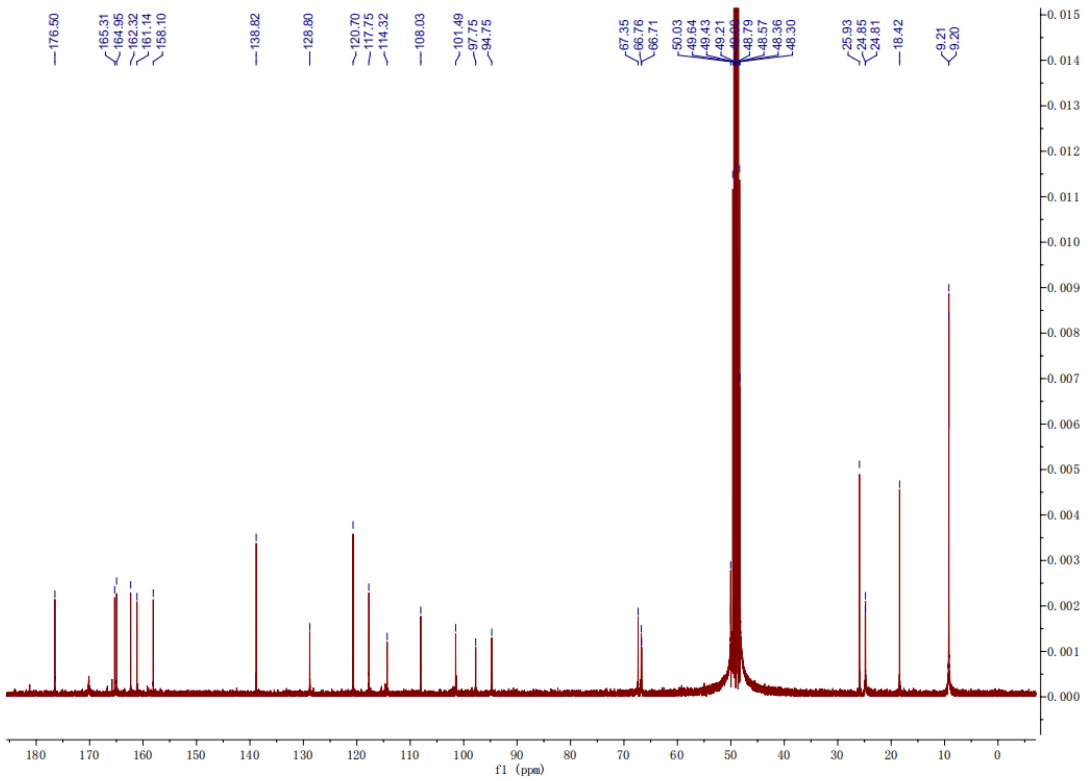

^{1}H-NMR spectrum of **XT10**

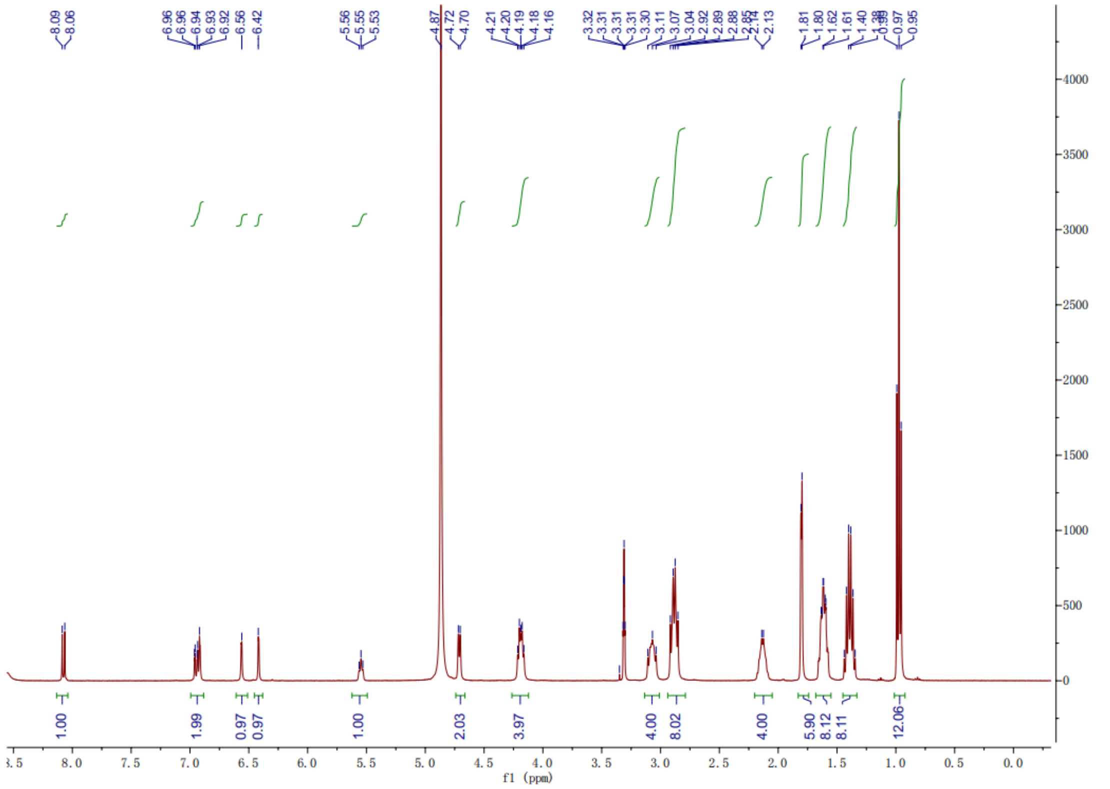

^{13}C-NMR spectrum of **XT10**

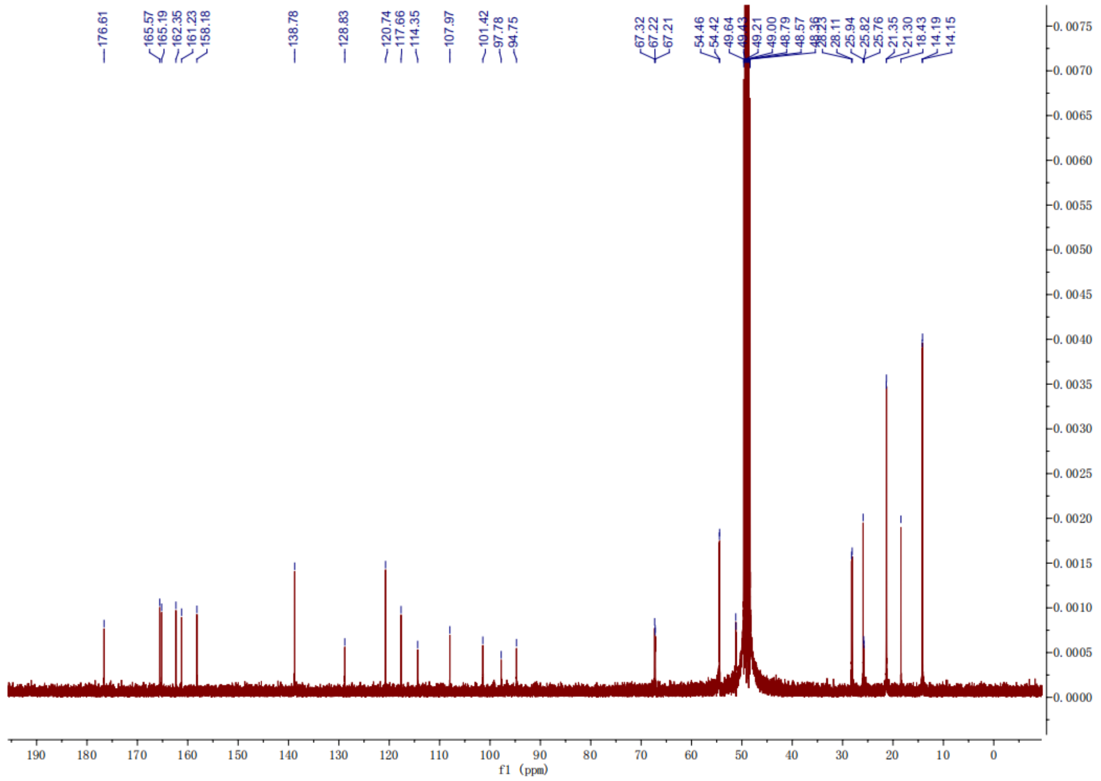

^{1}H-NMR spectrum of **XT11**

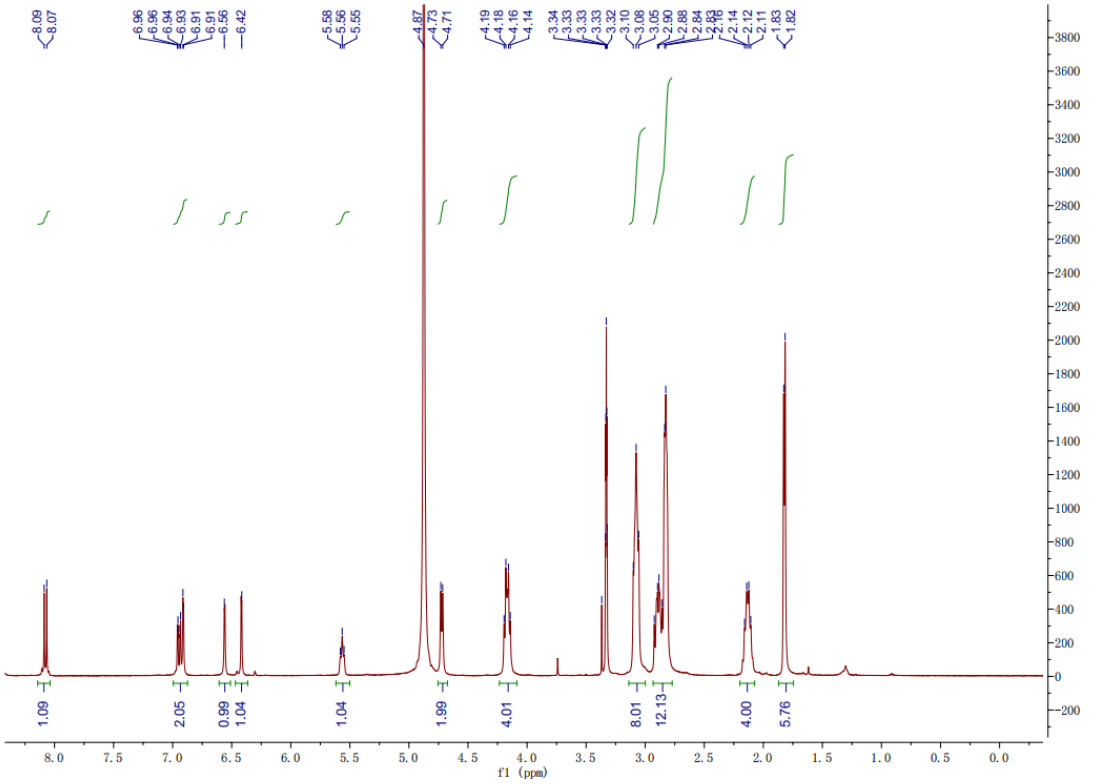

^{13}C-NMR spectrum of **XT11**

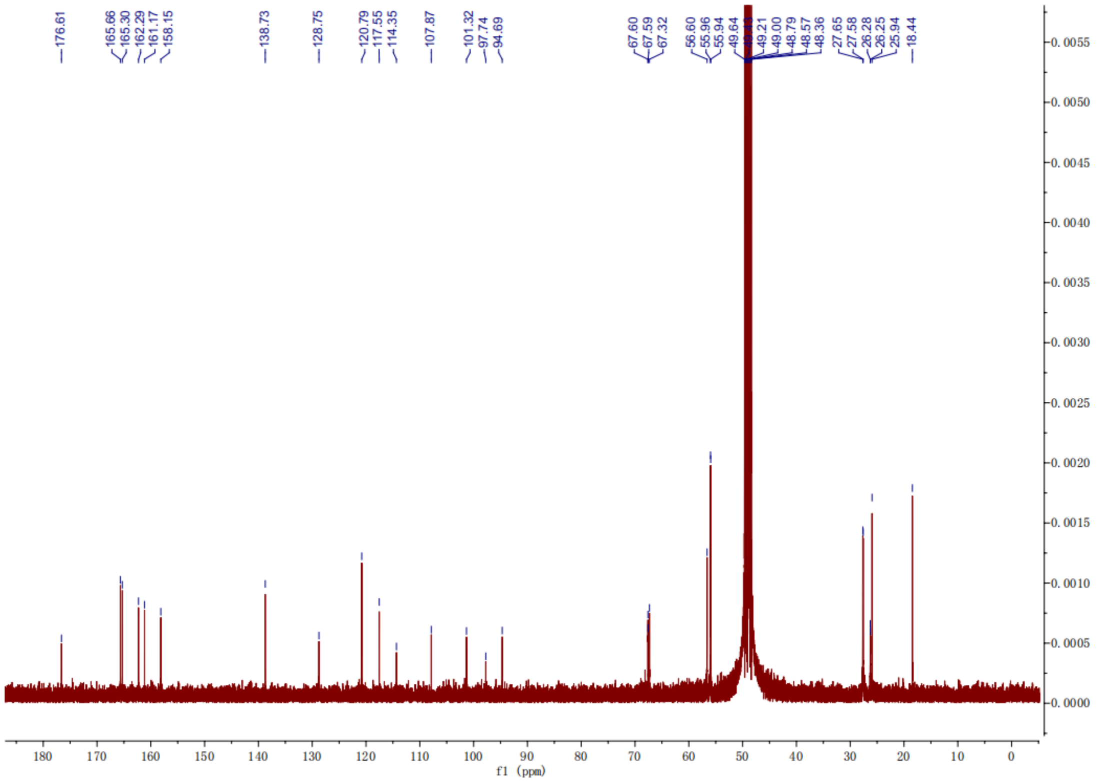

^{1}H-NMR spectrum of **XT12**

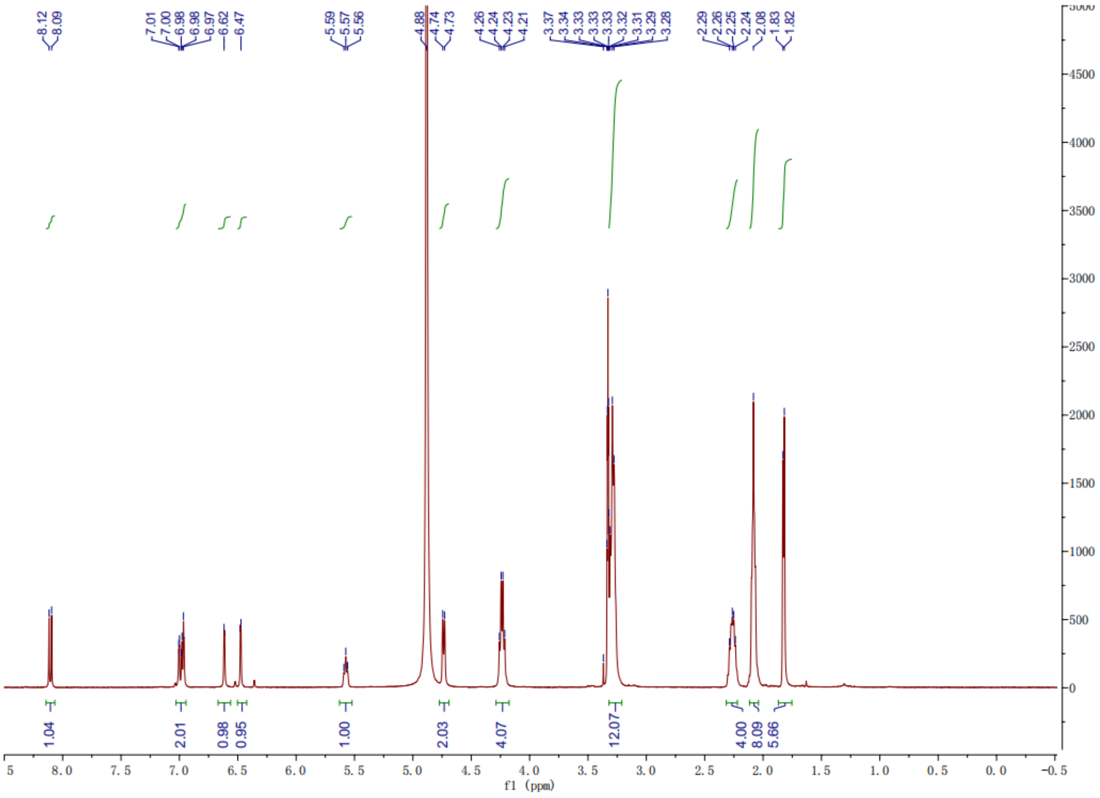

^{13}C-NMR spectrum of **XT12**

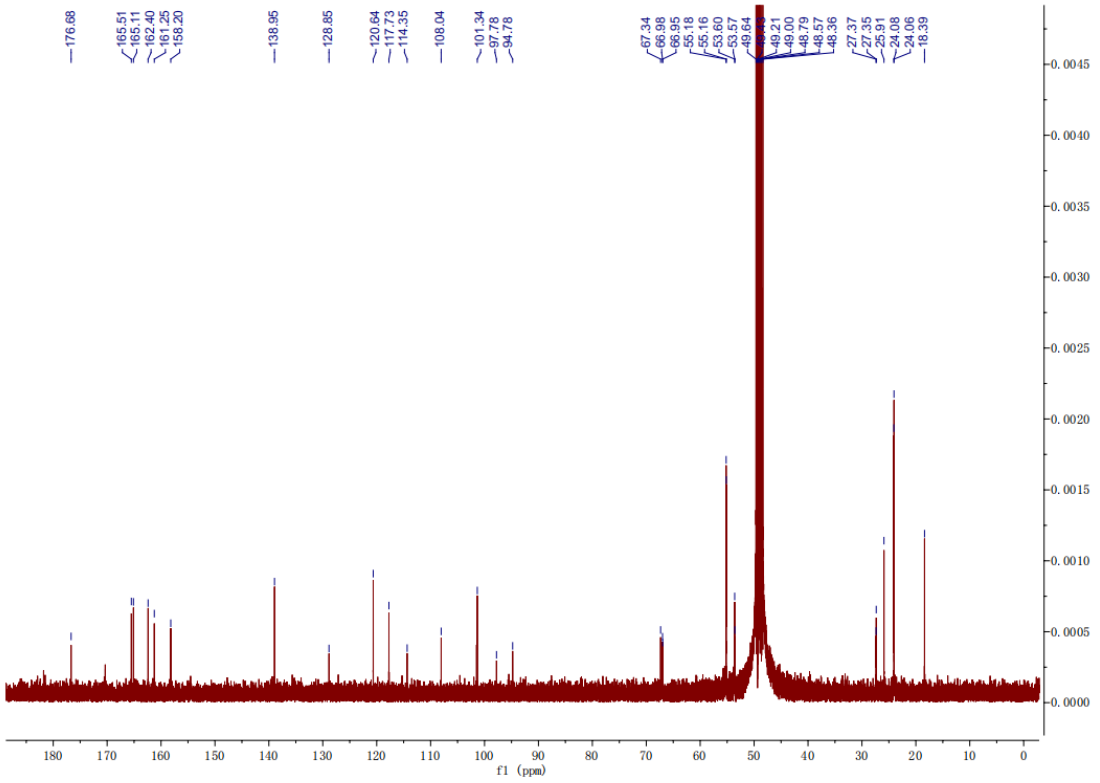

^{1}H-NMR spectrum of **XT13**

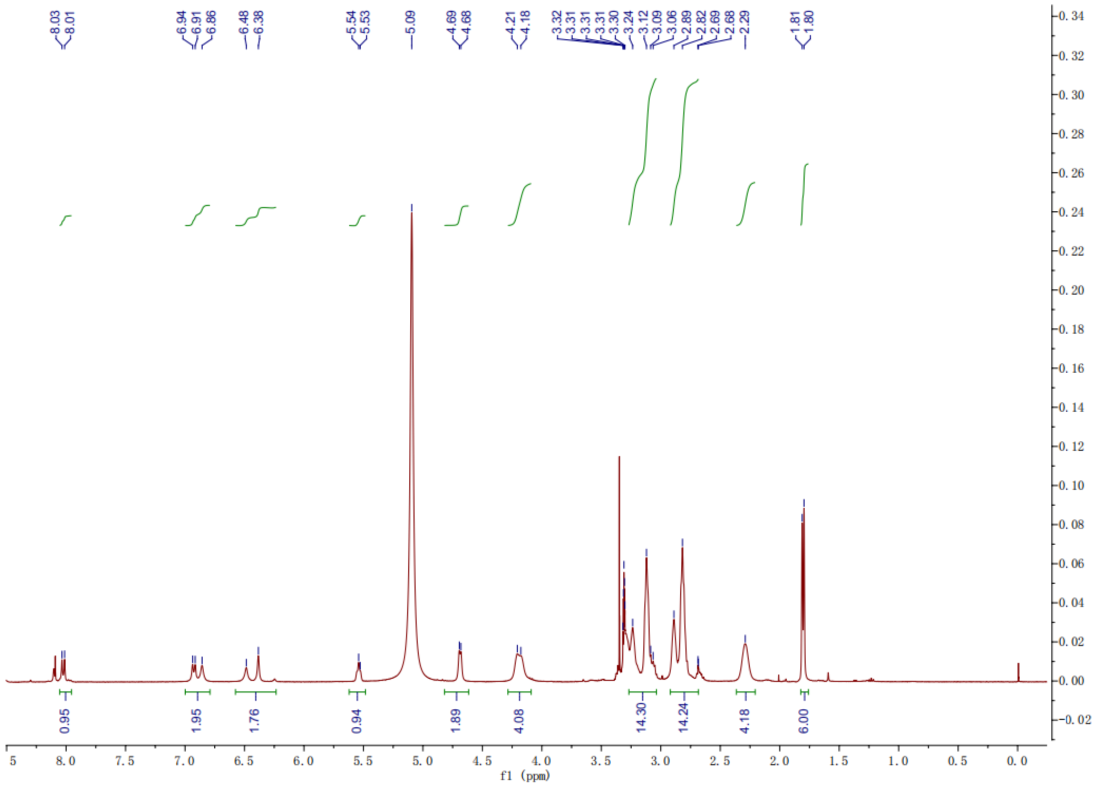

^{13}C-NMR spectrum of **XT13**

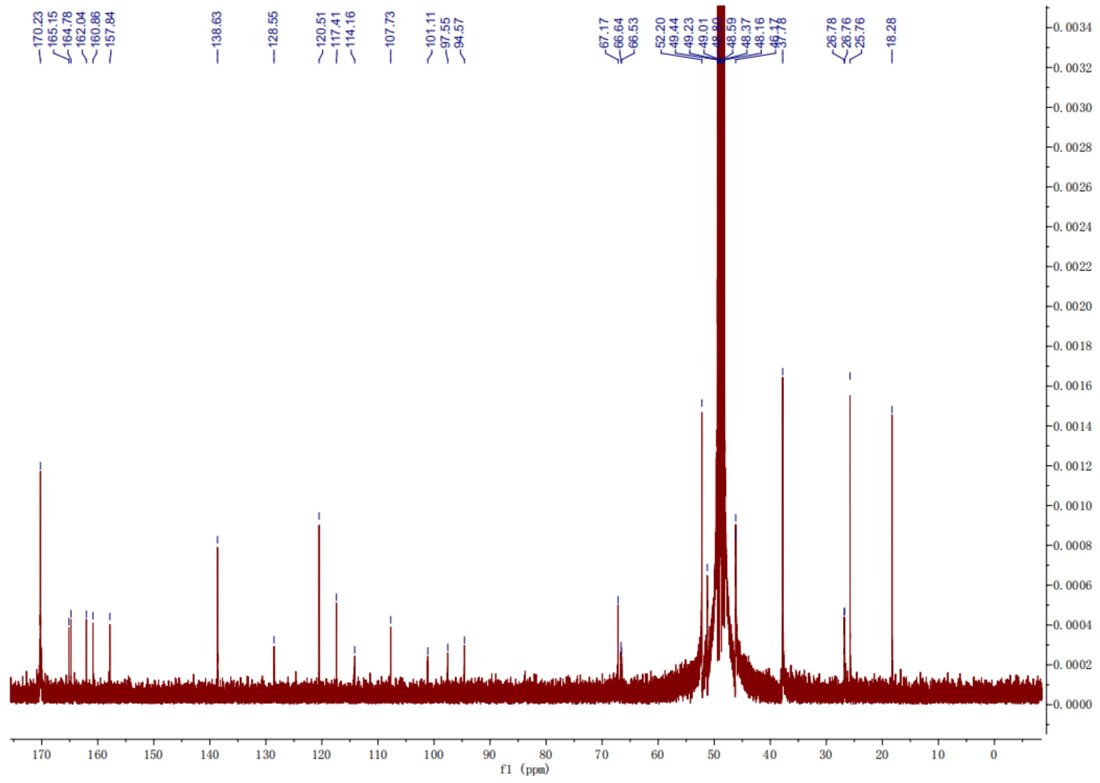

^{1}H-NMR spectrum of **XT16**

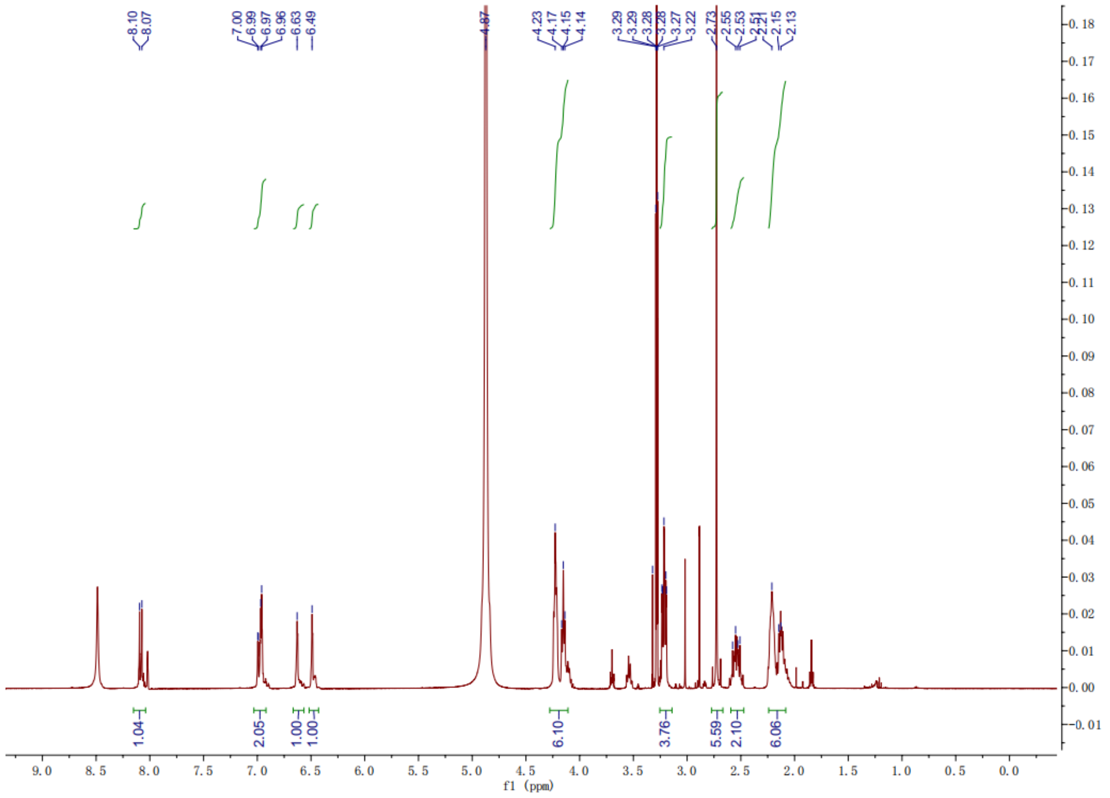

^{13}C-NMR spectrum of **XT16**

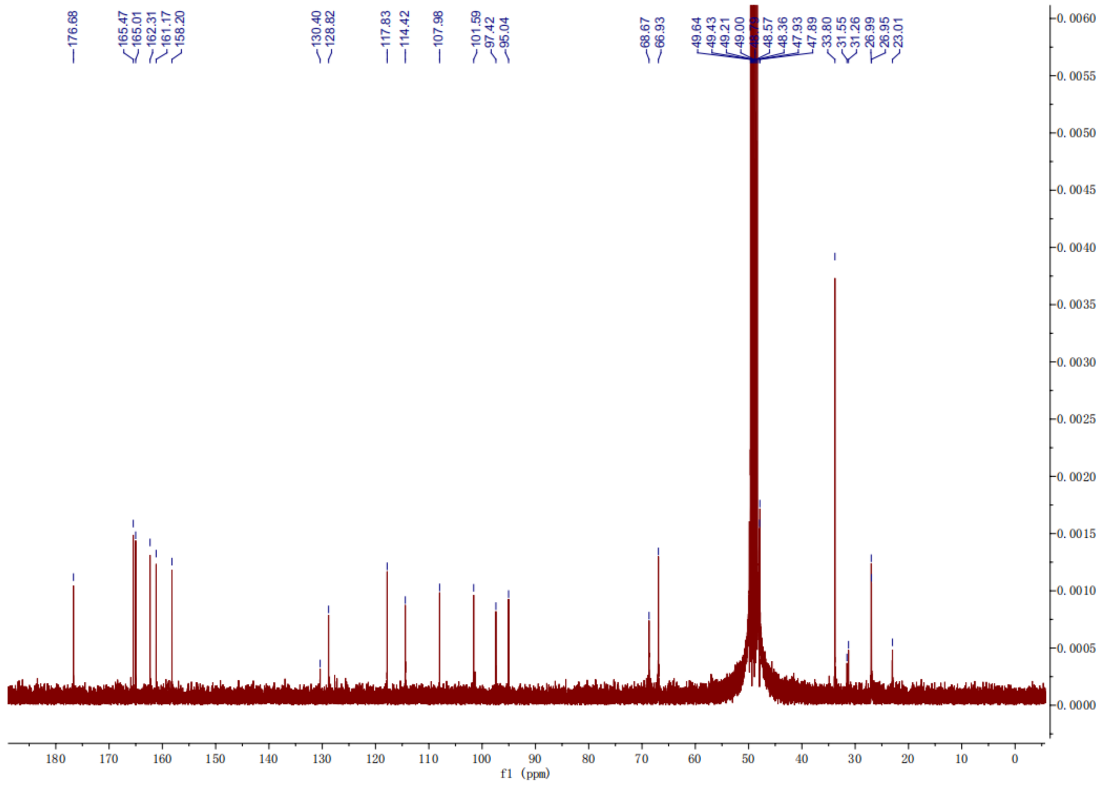

^{1}H-NMR spectrum of **XT17**

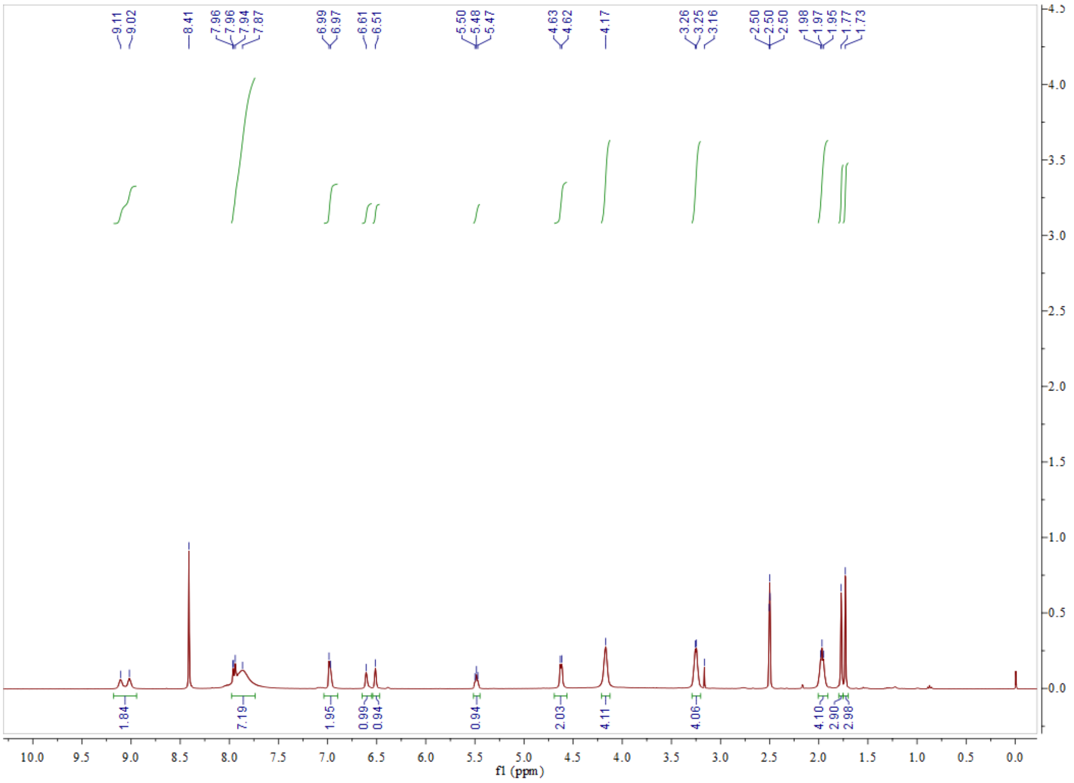

^{13}C-NMR spectrum of **XT17**

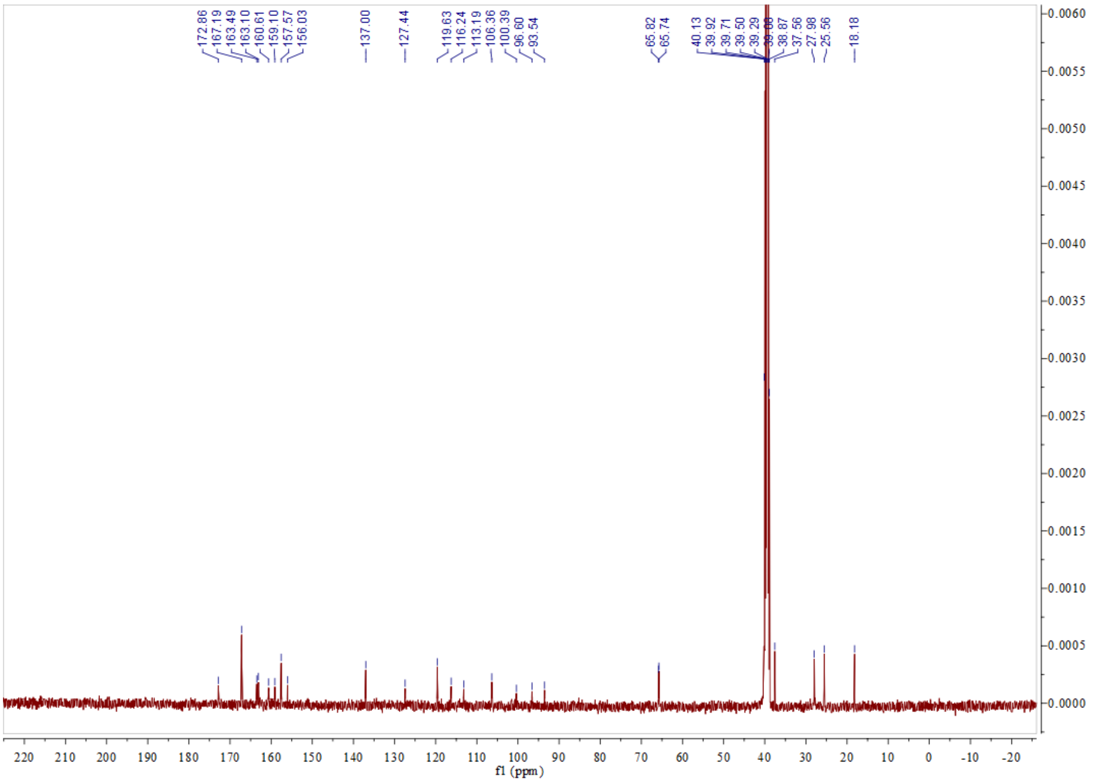

^{1}H-NMR spectrum of **XT18**

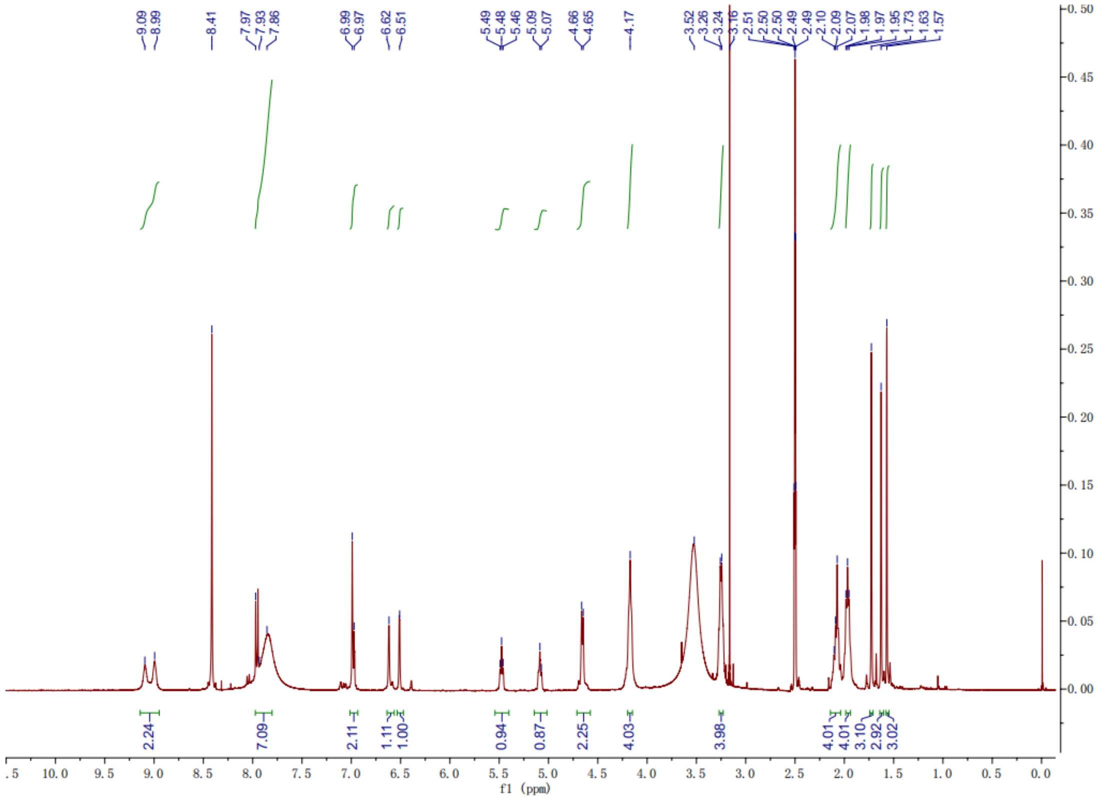

^{13}C-NMR spectrum of **XT18**

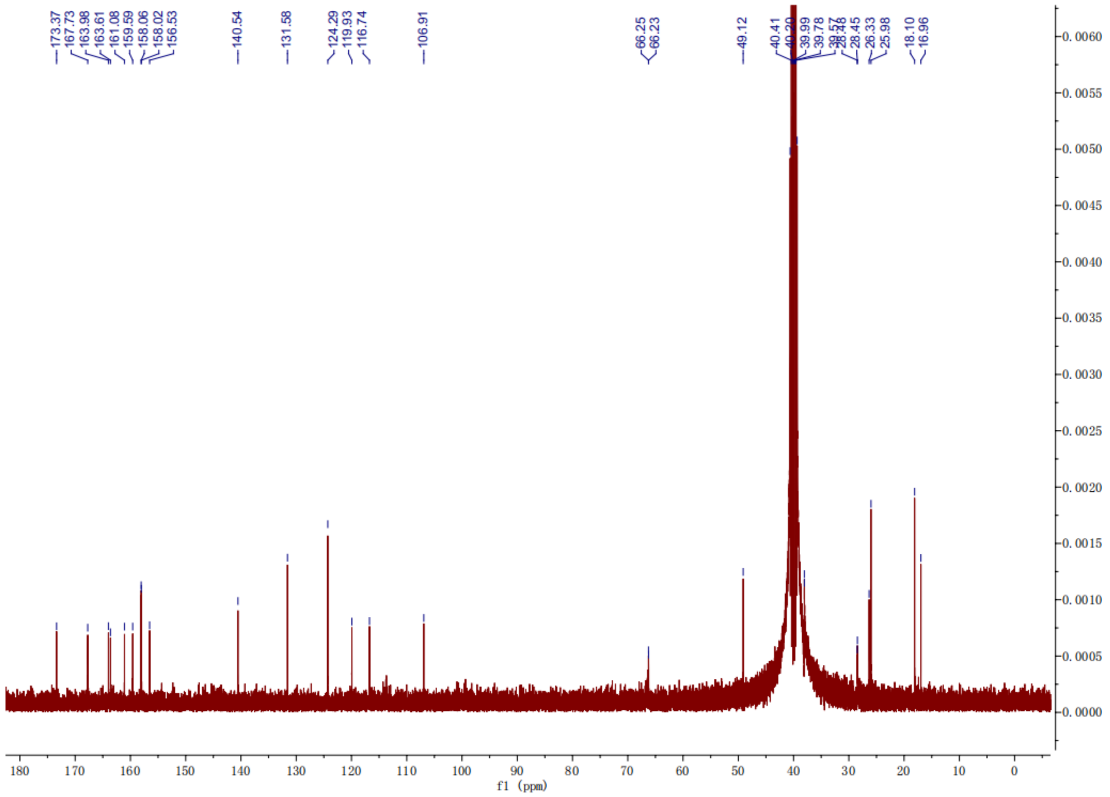

^{1}H-NMR spectrum of **XT19**

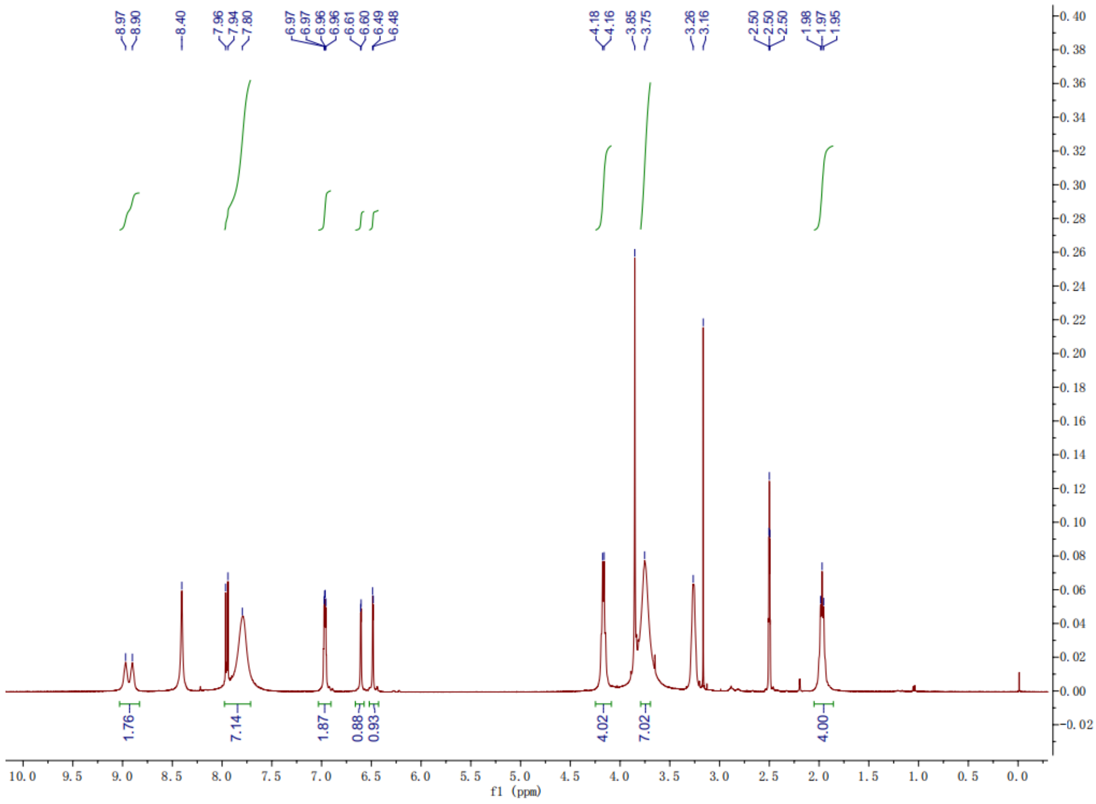

^{13}C-NMR spectrum of **XT19**

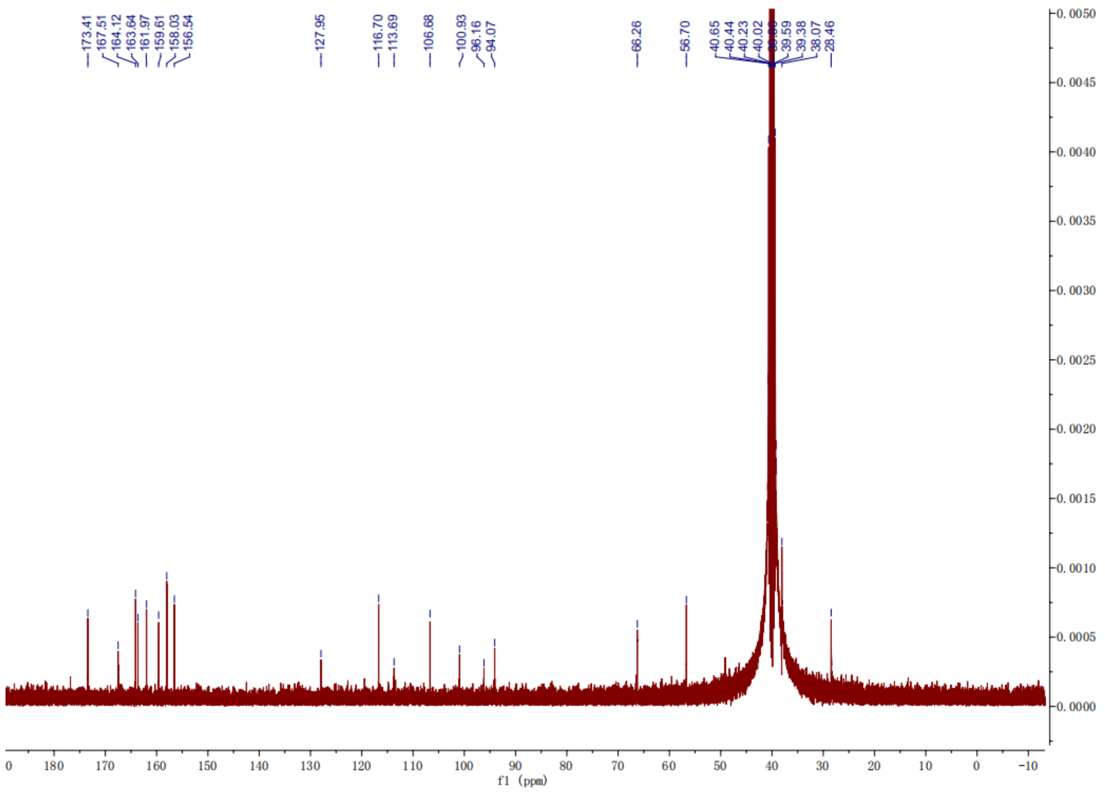

^{1}H-NMR spectrum of **XT20**

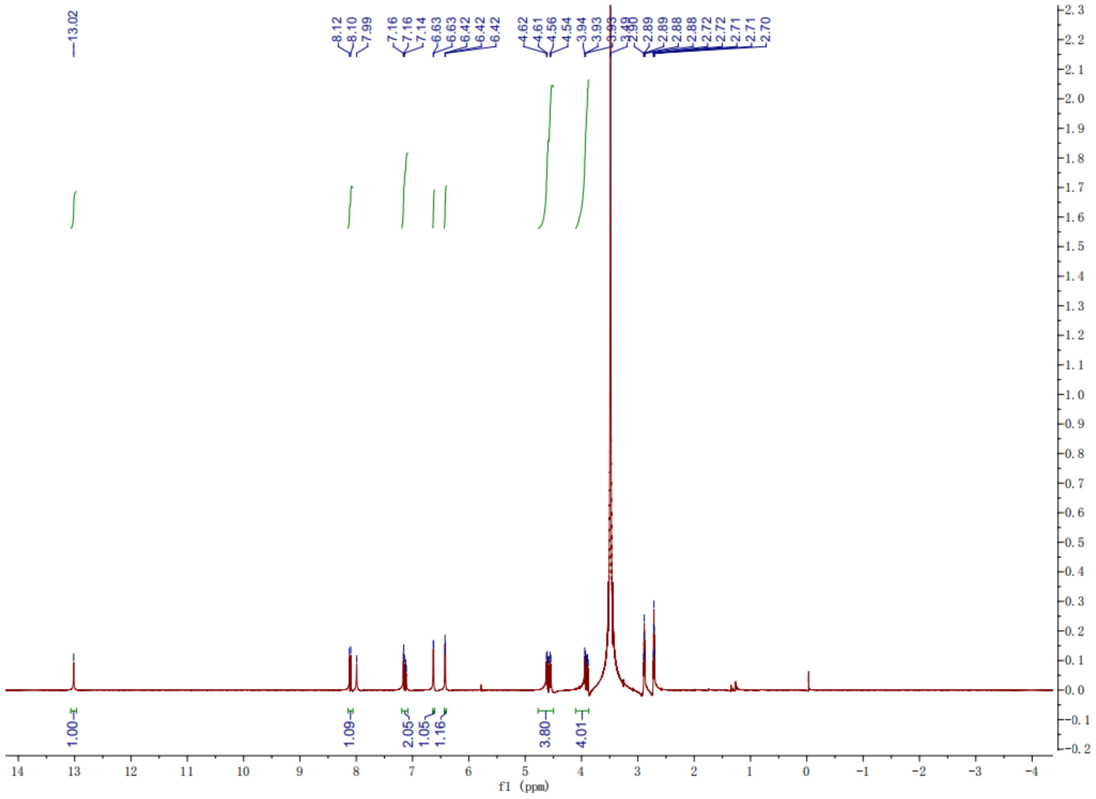

^{13}C-NMR spectrum of **XT20**

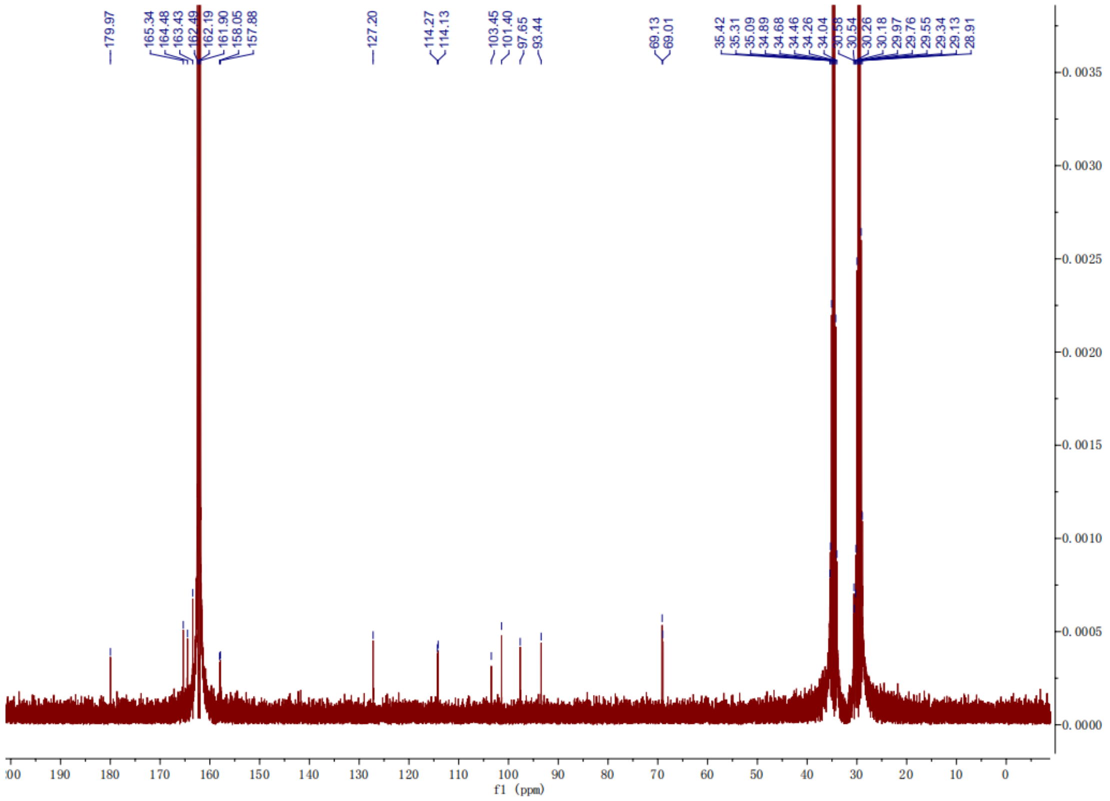

^{1}H-NMR spectrum of **XT21**

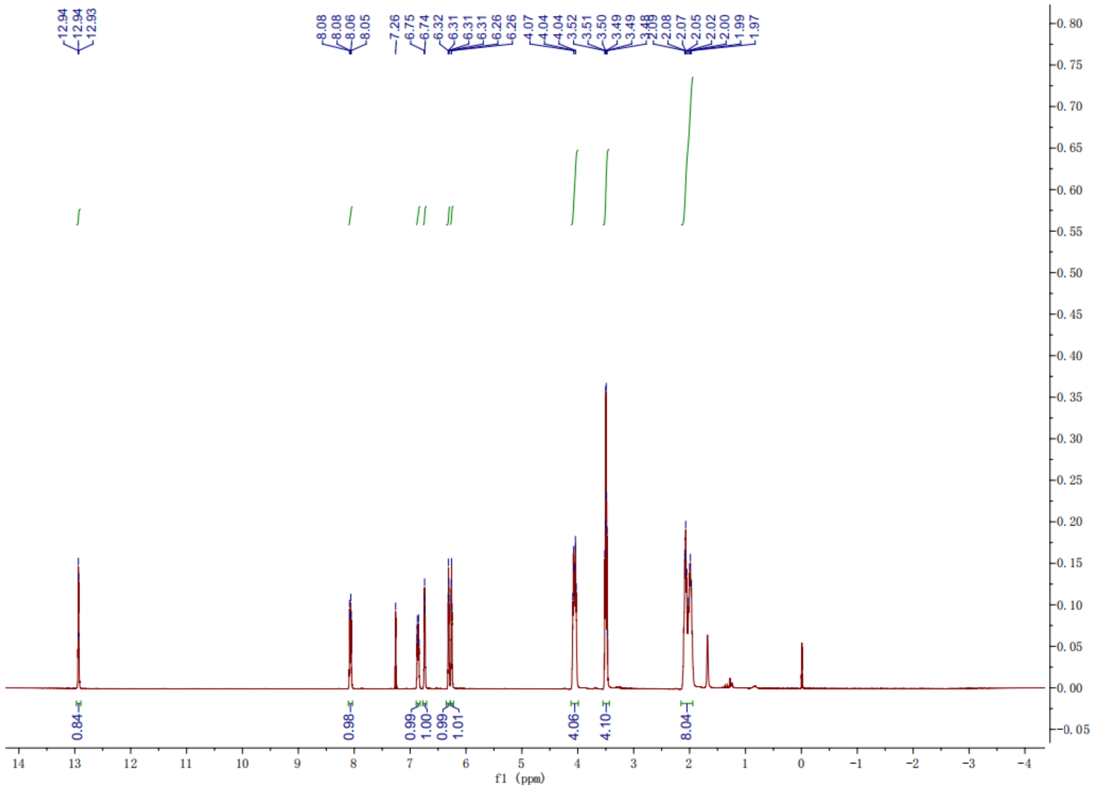

^{13}C-NMR spectrum of **XT21**

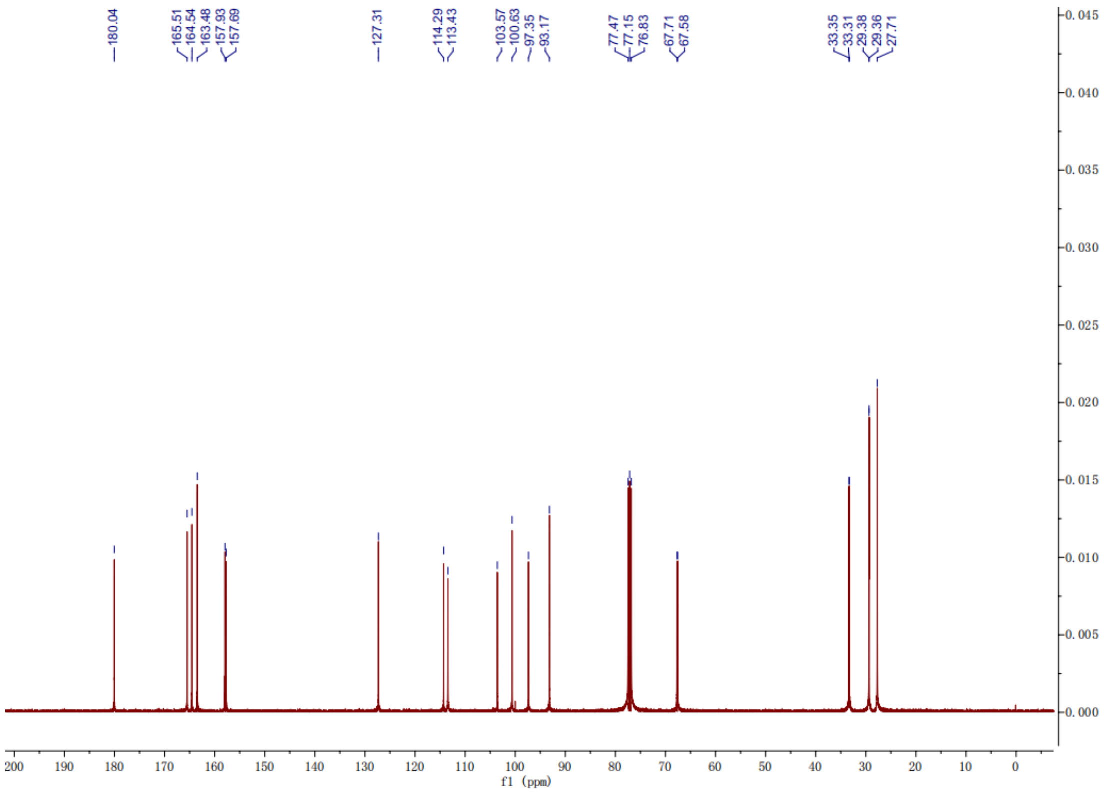

^{1}H-NMR spectrum of **XT22**

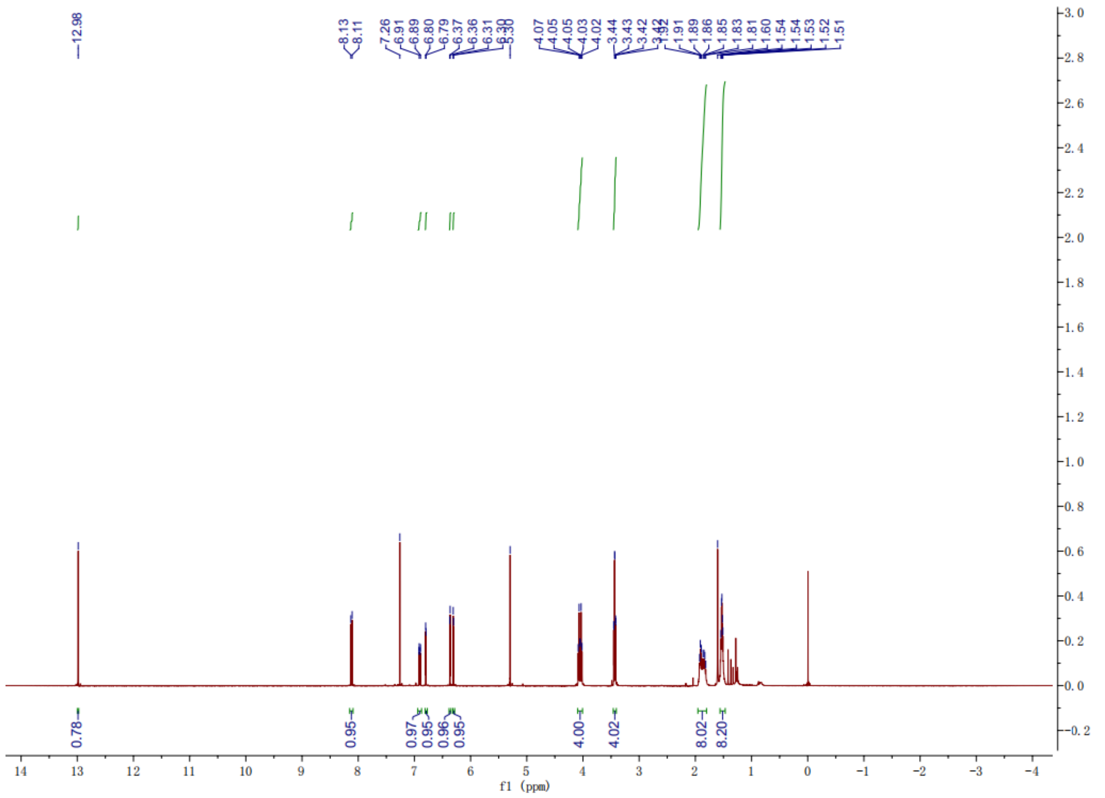

^{13}C-NMR spectrum of **XT22**

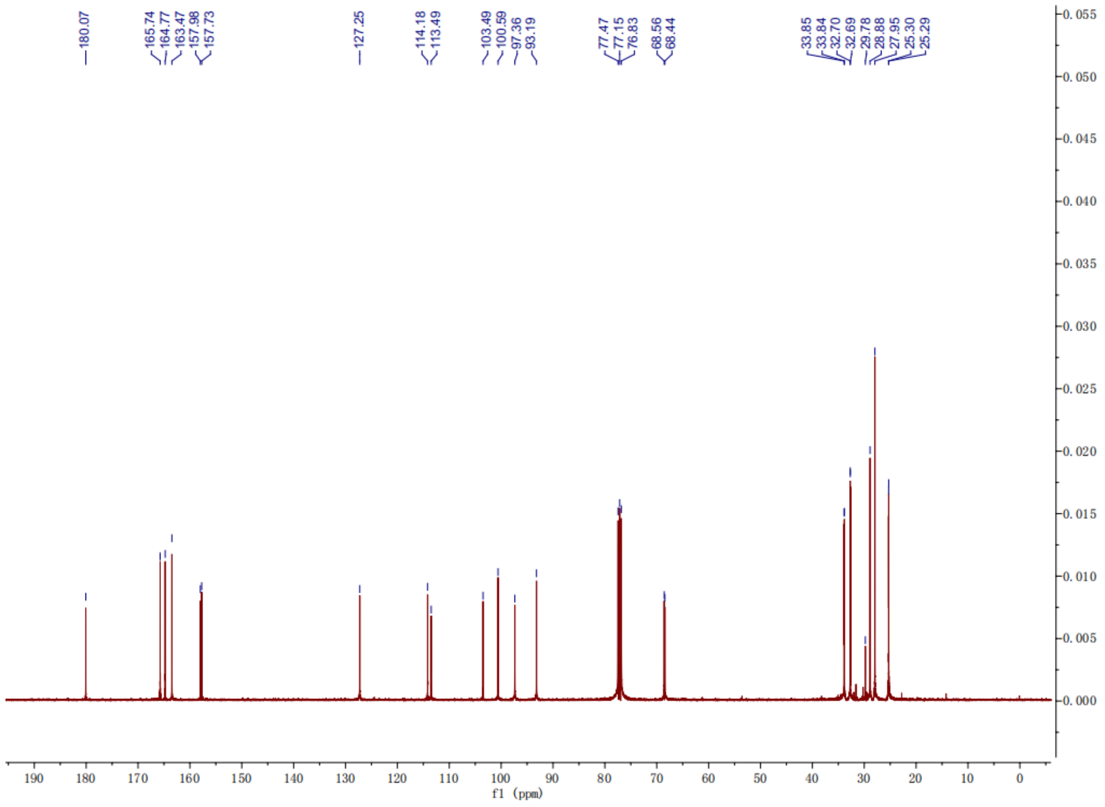

^{1}H-NMR spectrum of **XT23**

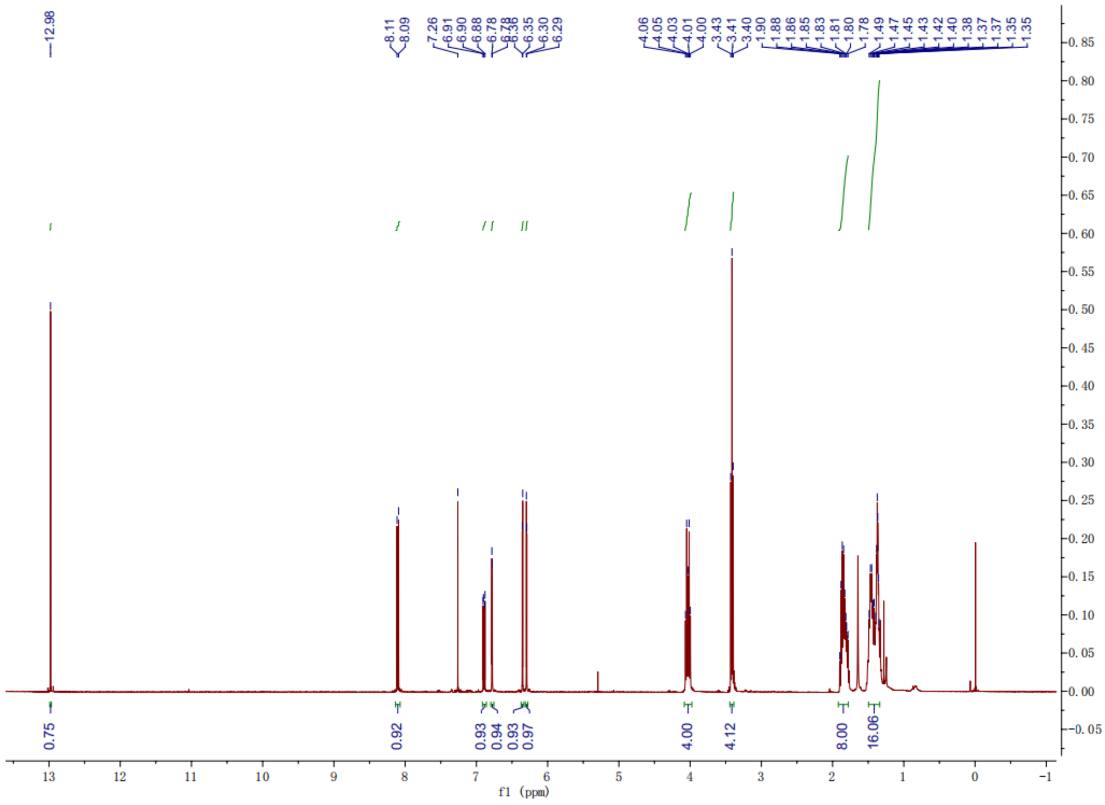

^{13}C-NMR spectrum of **XT23**

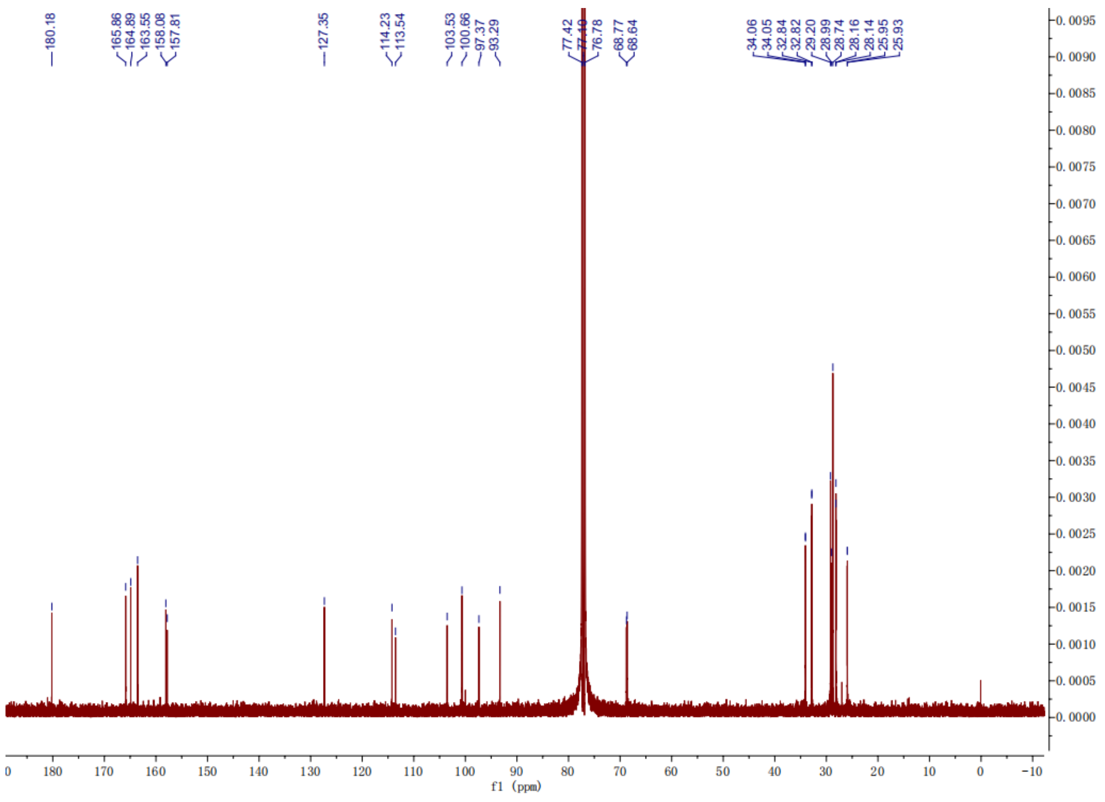

^{1}H-NMR spectrum of **XT24**

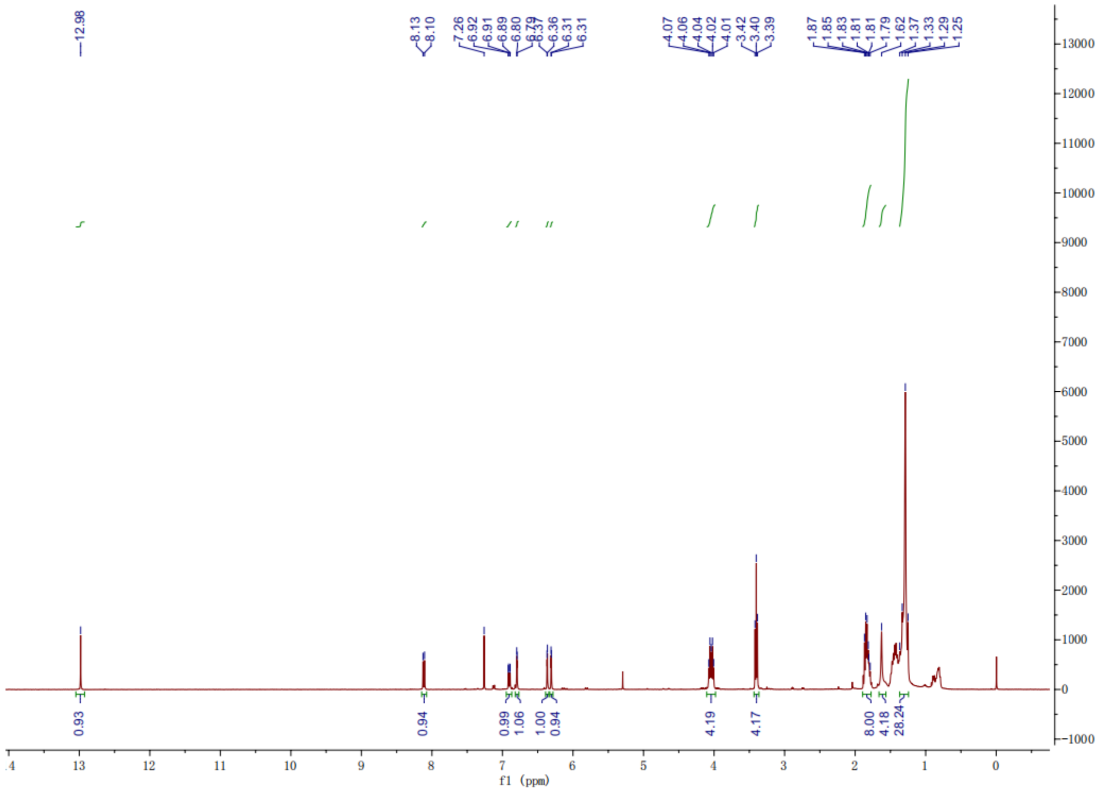

^{13}C-NMR spectrum of **XT24**

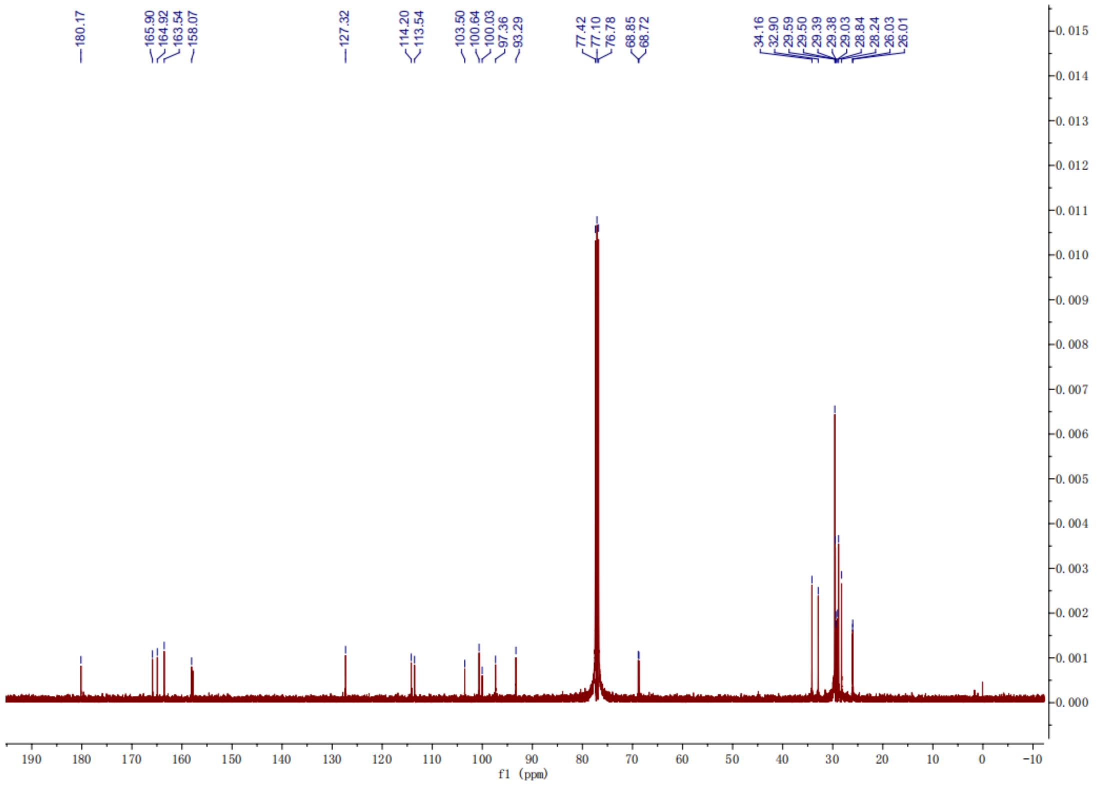

^{1}H-NMR spectrum of **XT25**

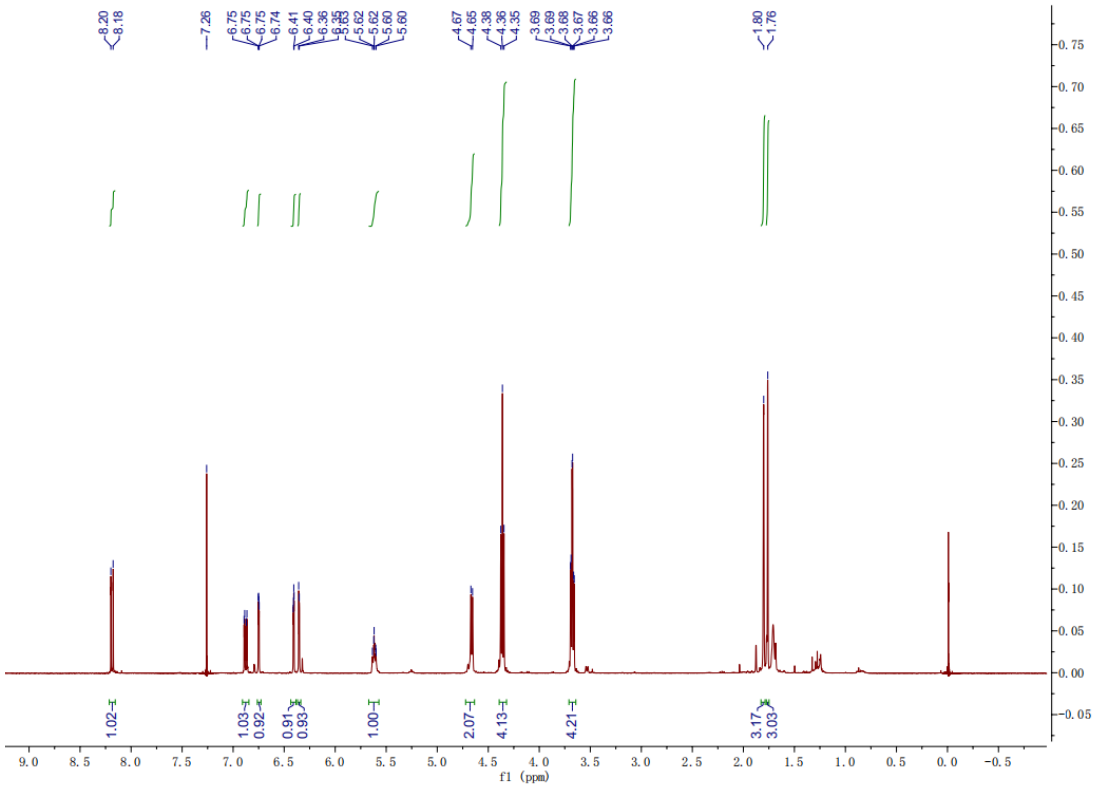

^{13}C-NMR spectrum of **XT25**

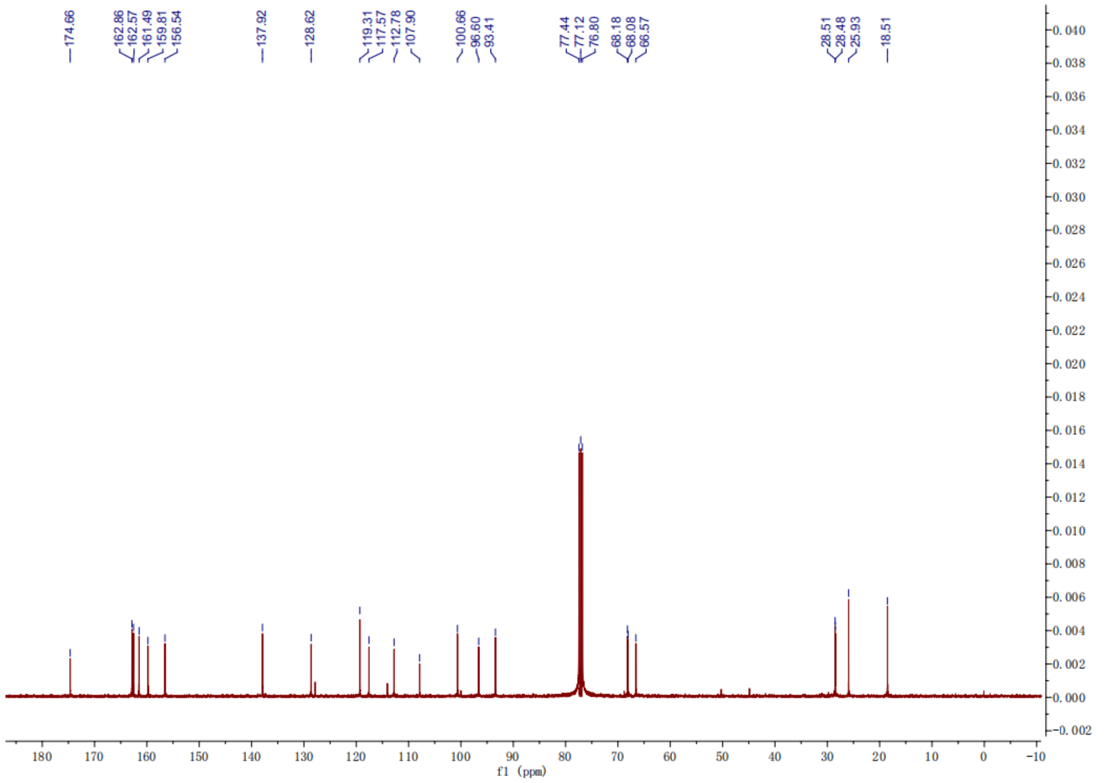

^{1}H-NMR spectrum of **XT26**

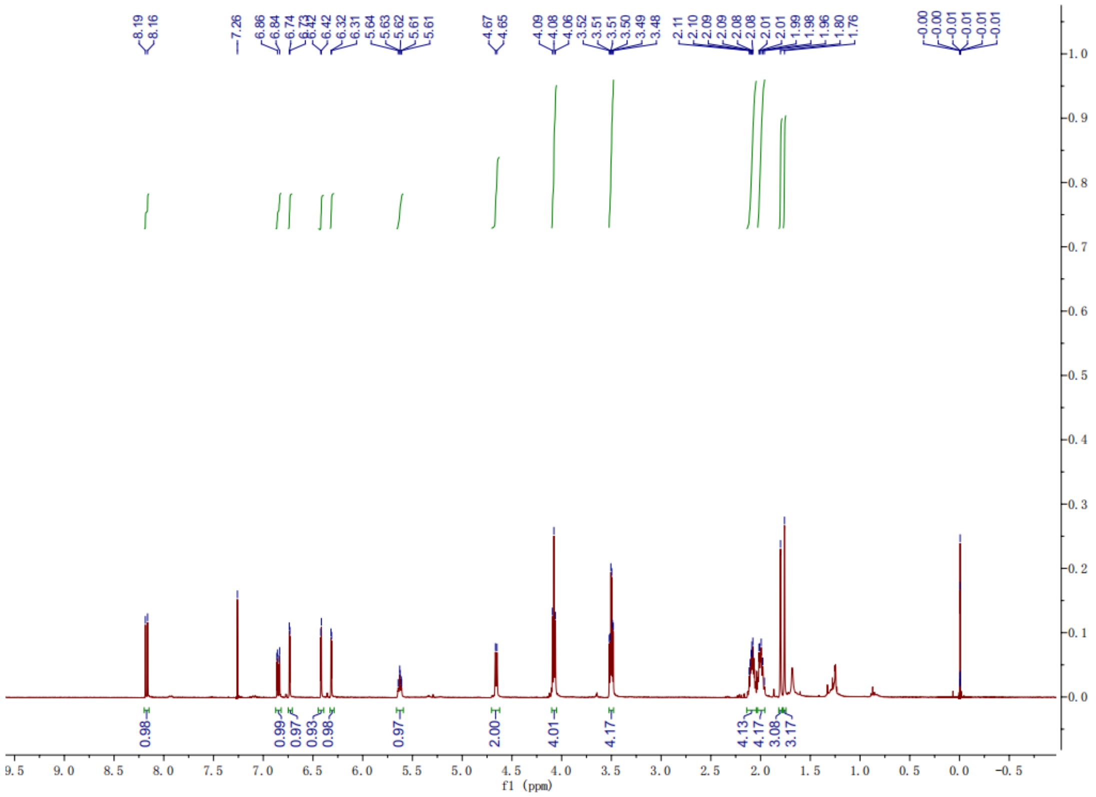

^{13}C-NMR spectrum of **XT26**

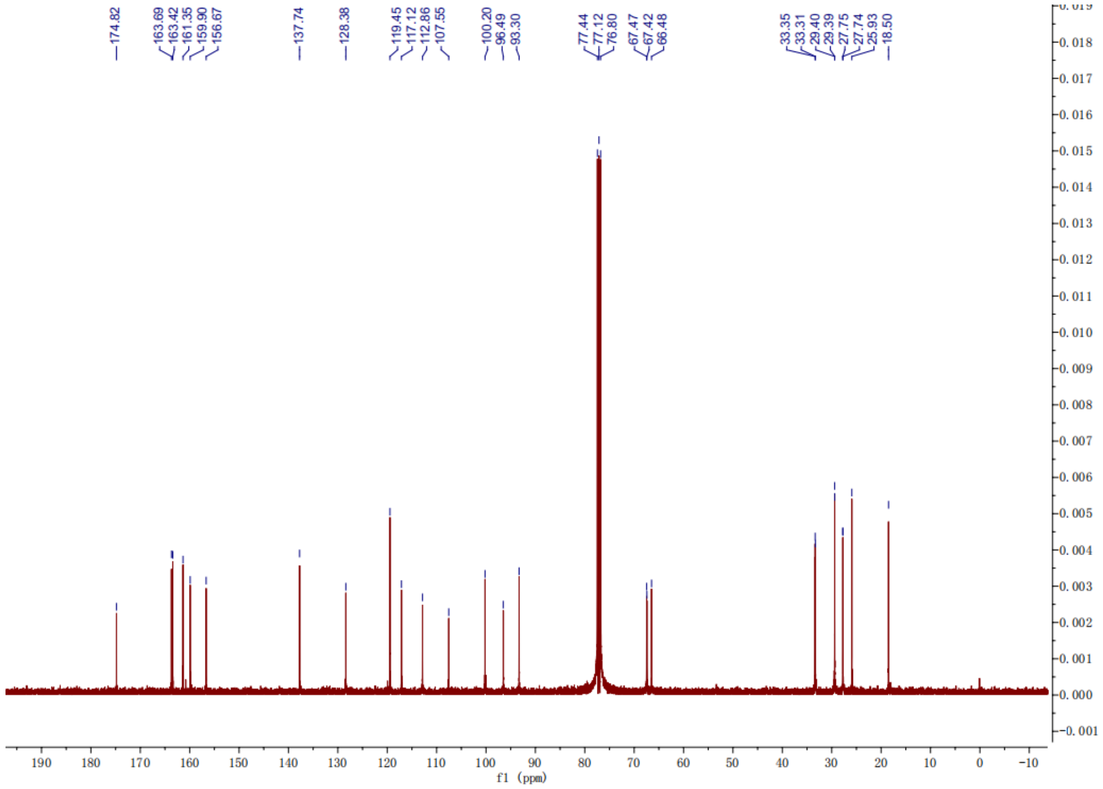

^{1}H-NMR spectrum of **XT27**

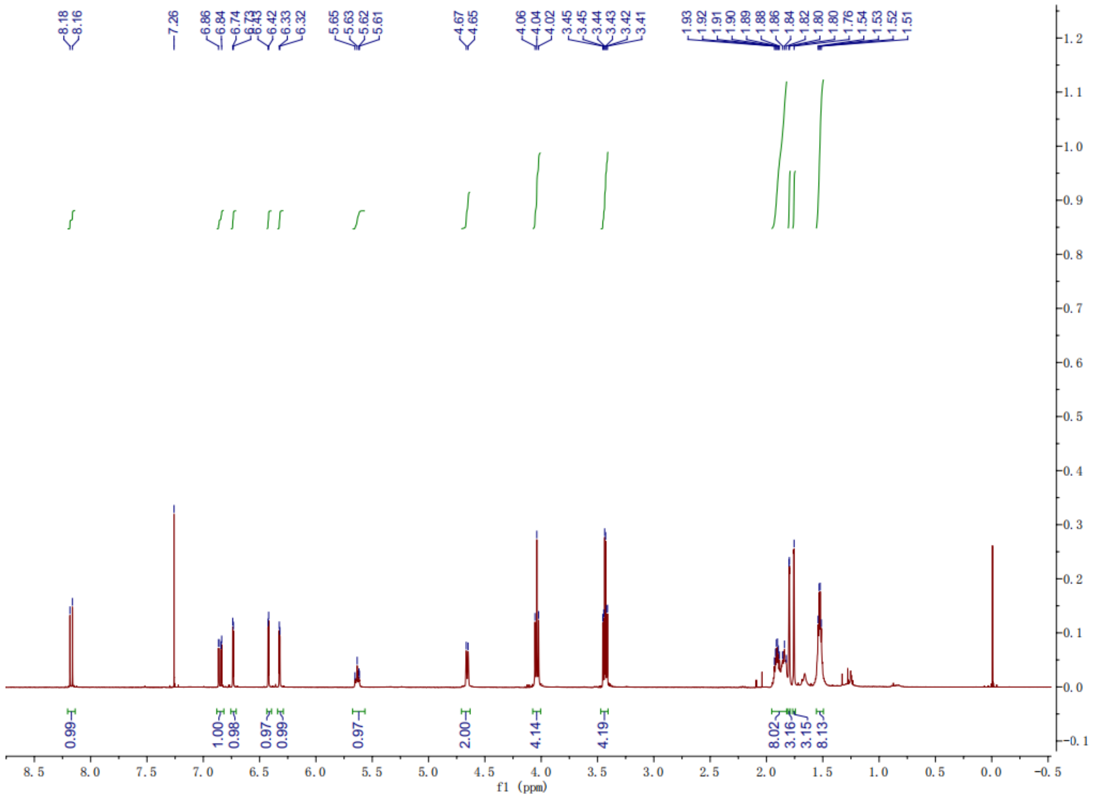

^{13}C-NMR spectrum of **XT27**

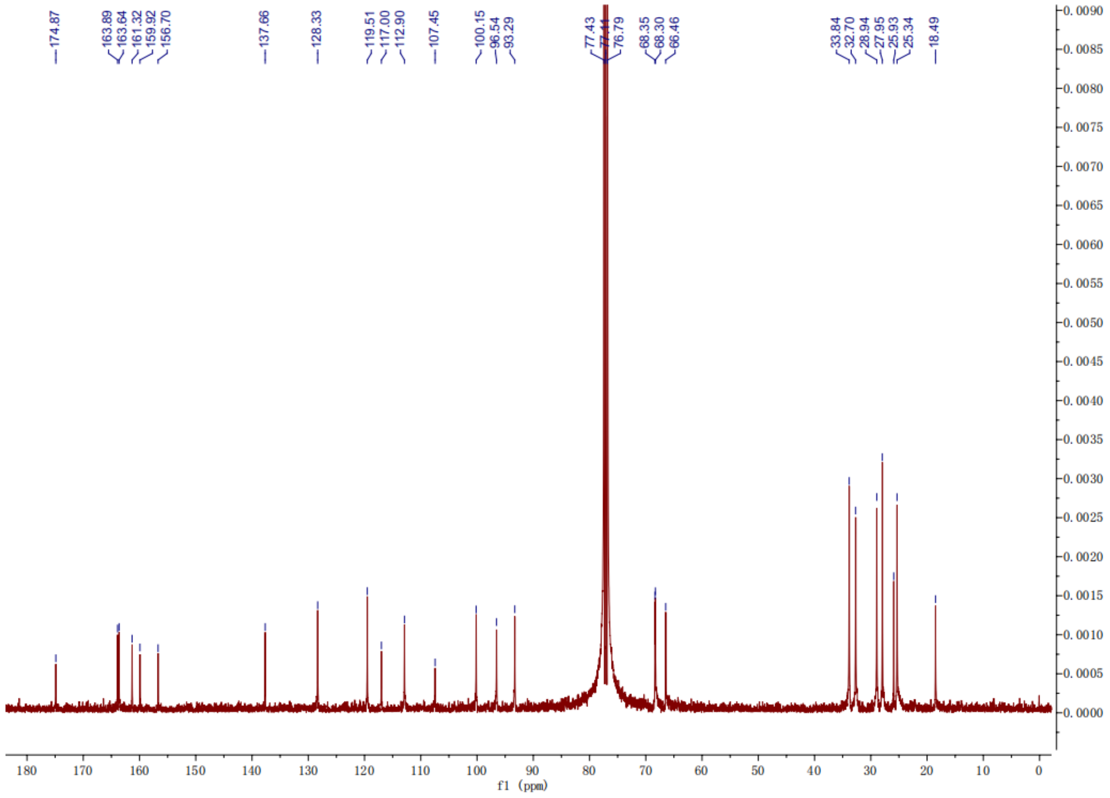

^{1}H-NMR spectrum of **XT28**

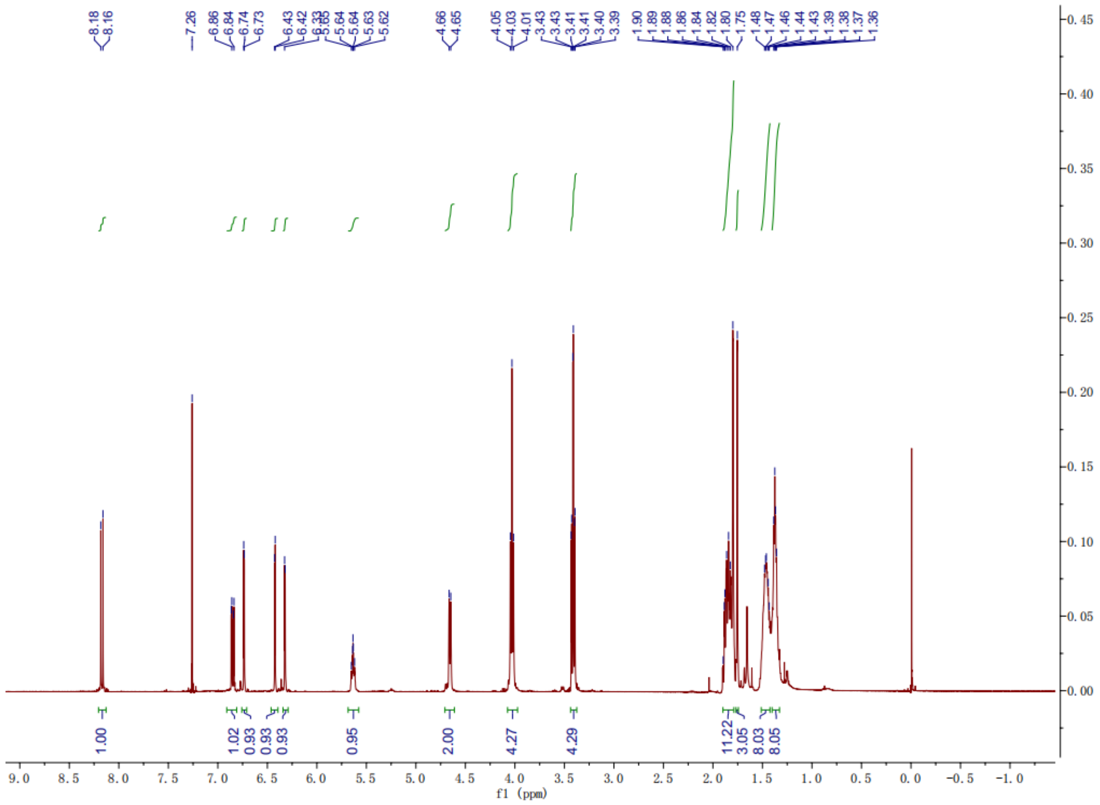

^{13}C-NMR spectrum of **XT28**

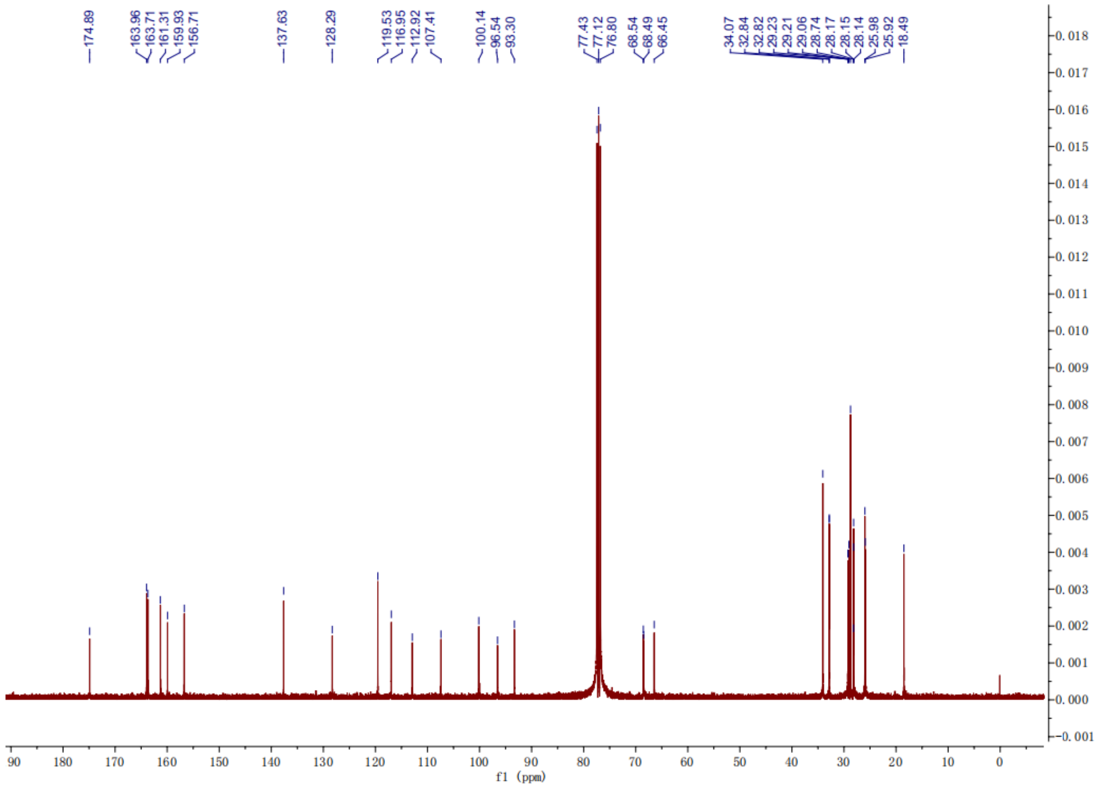

^{1}H-NMR spectrum of **XT29**

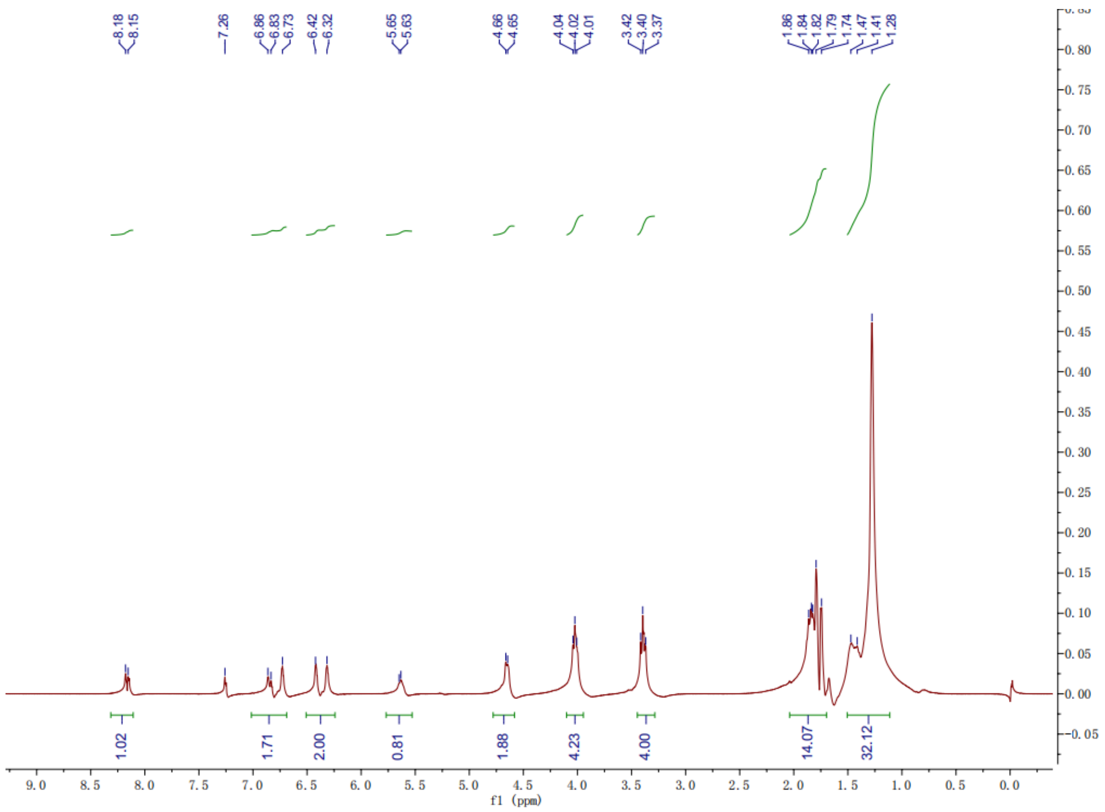

^{13}C-NMR spectrum of **XT29**

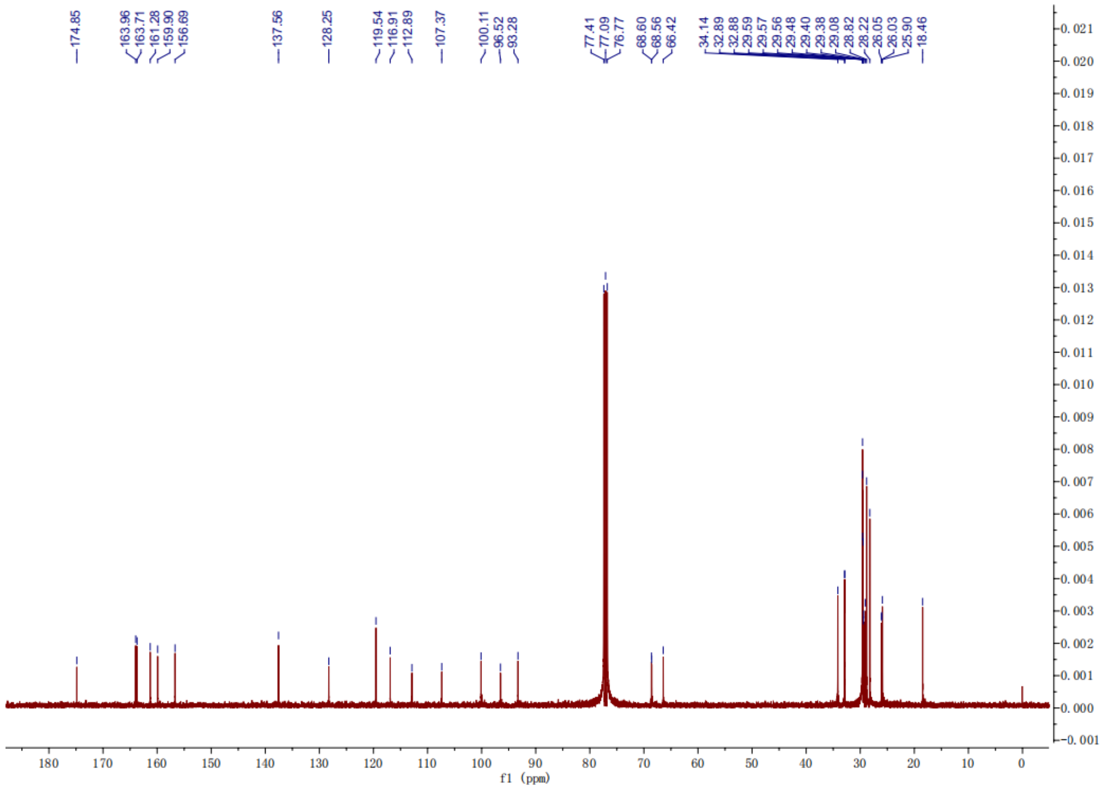

^{1}H-NMR spectrum of **XT31**

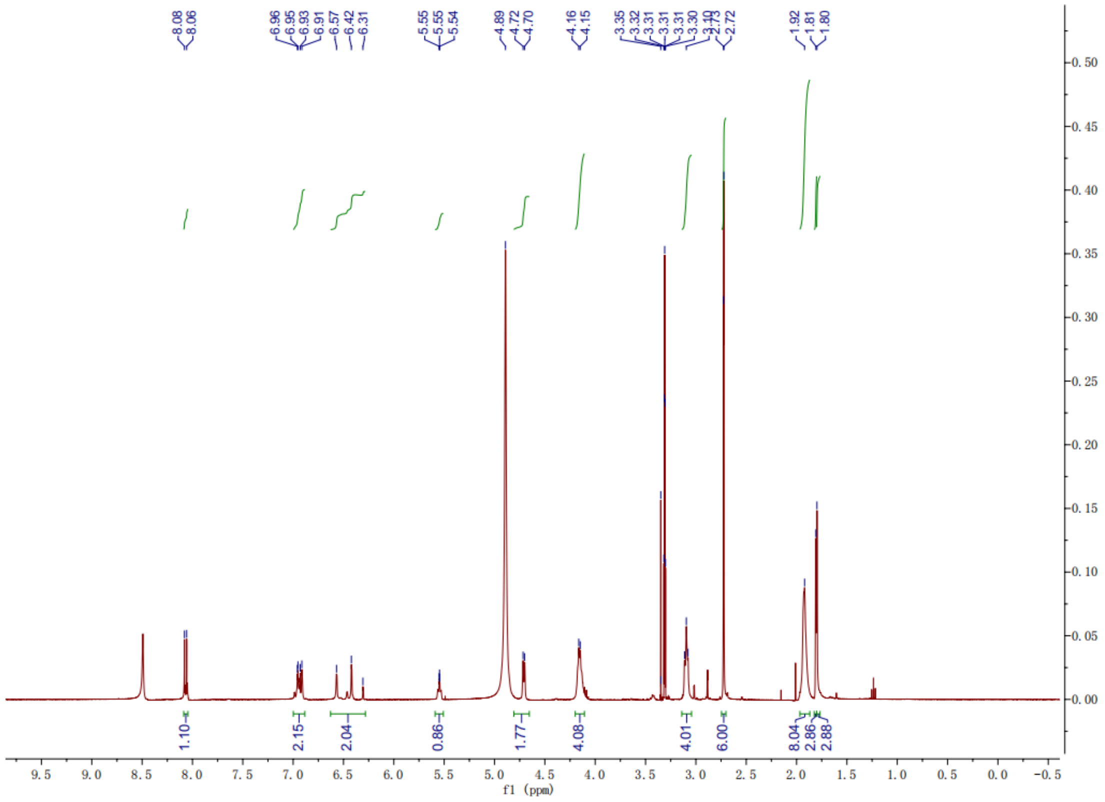

^{13}C-NMR spectrum of **XT31**

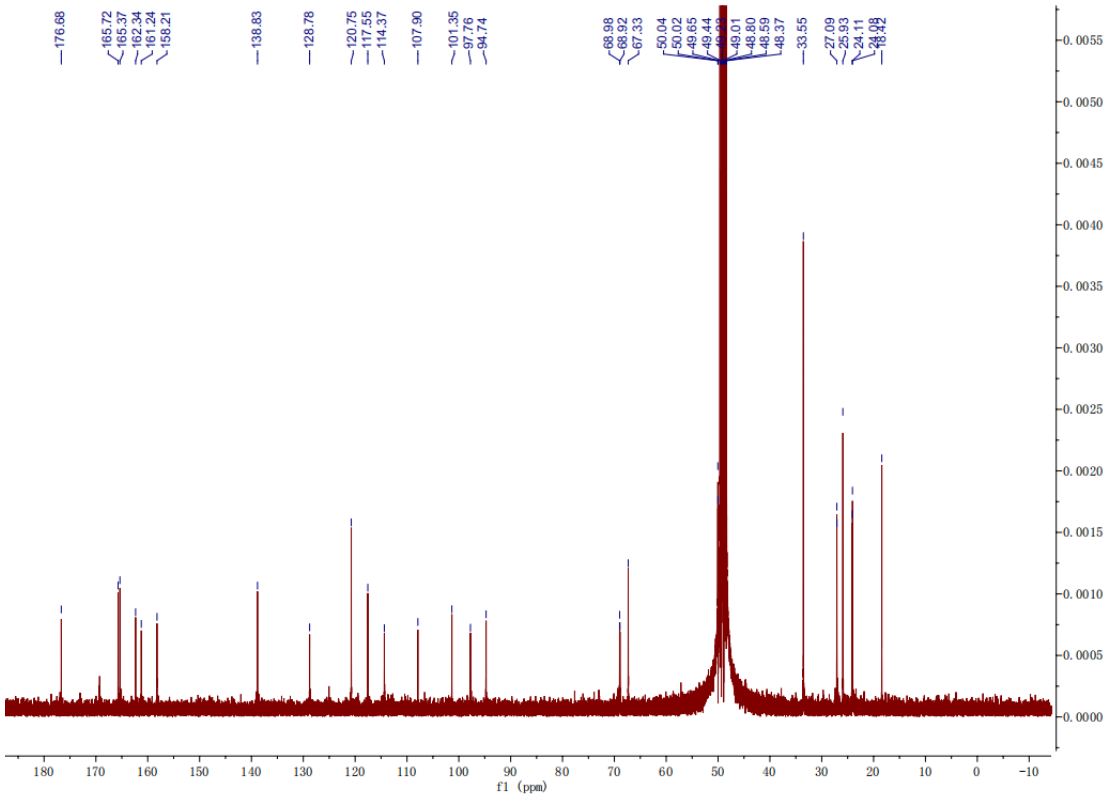

^{1}H-NMR spectrum of **XT32**

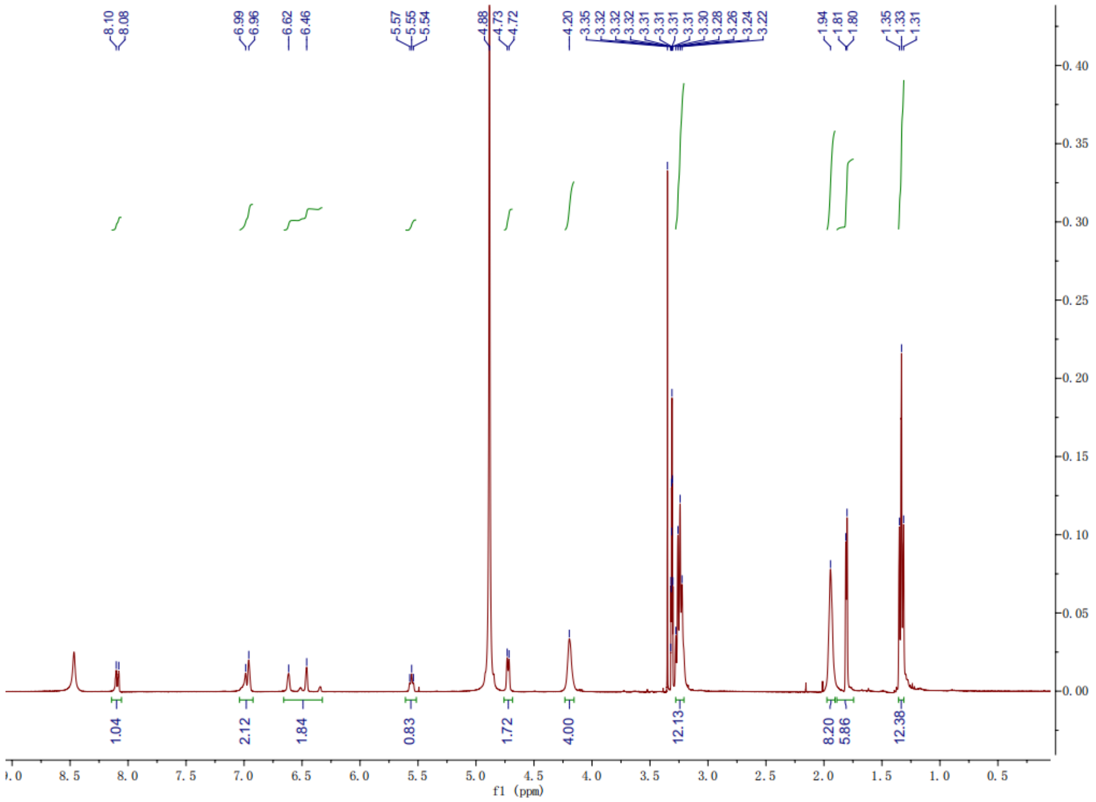

^{13}C-NMR spectrum of **XT32**

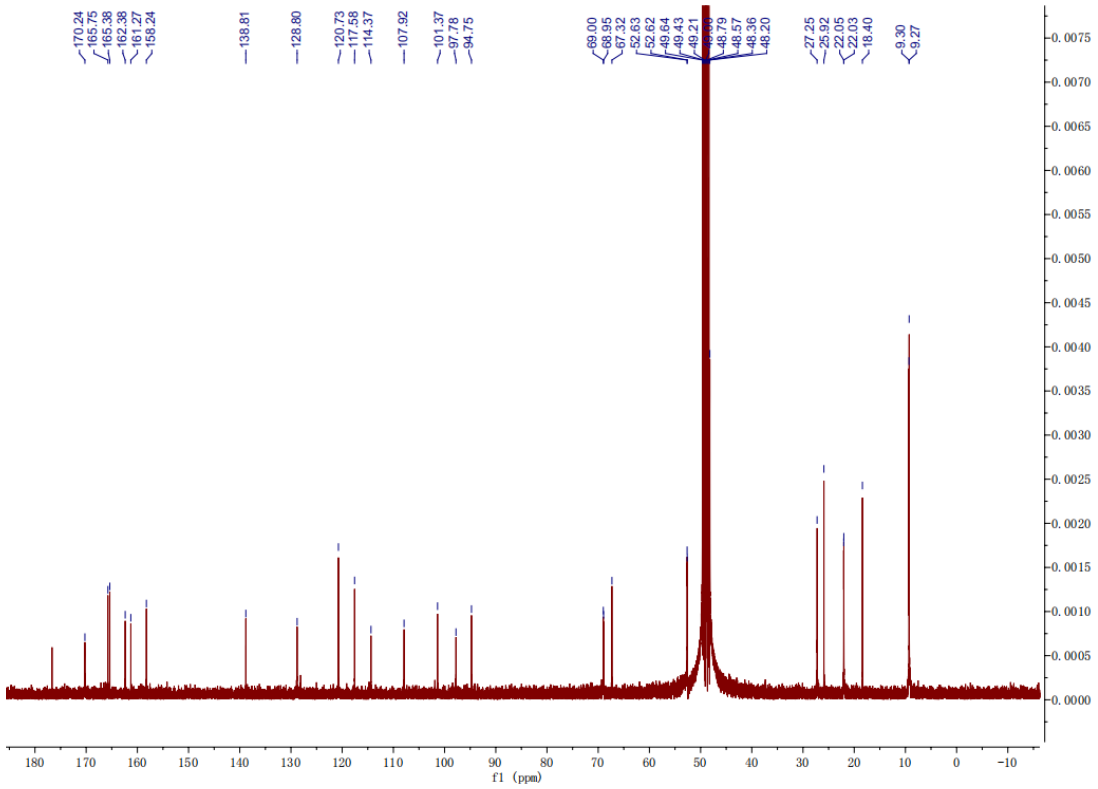

^{1}H-NMR spectrum of **XT33**

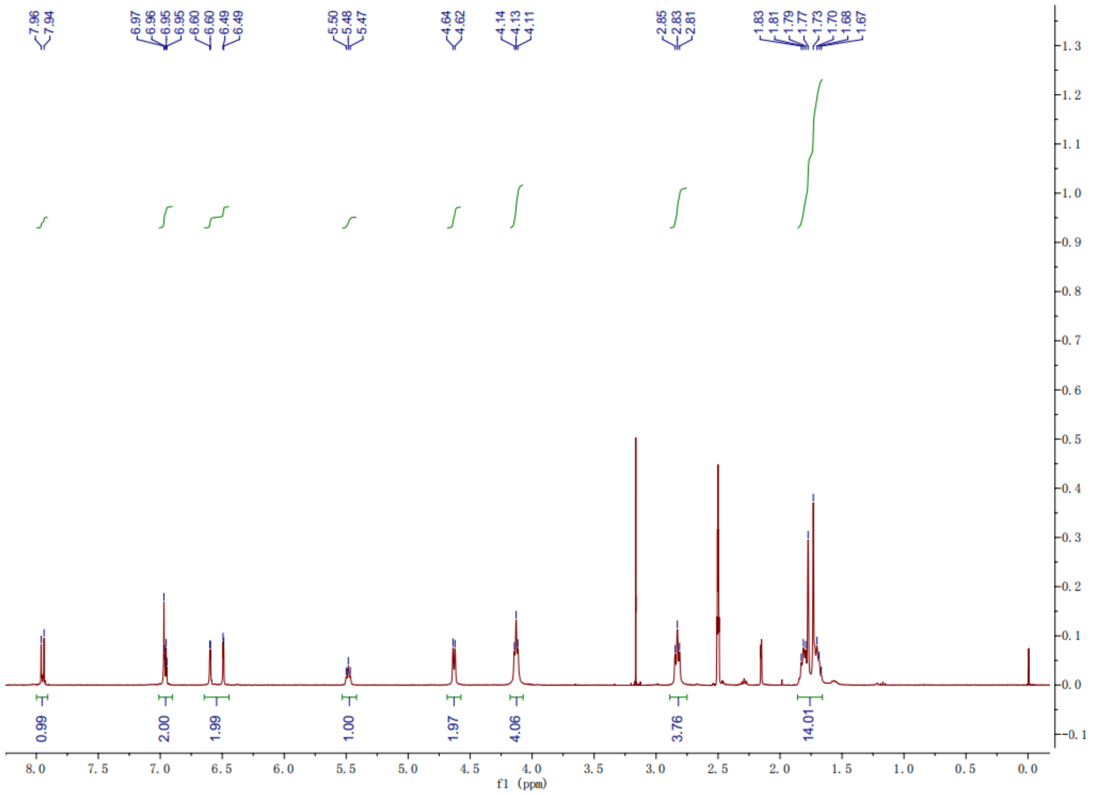

^{13}C-NMR spectrum of **XT33**

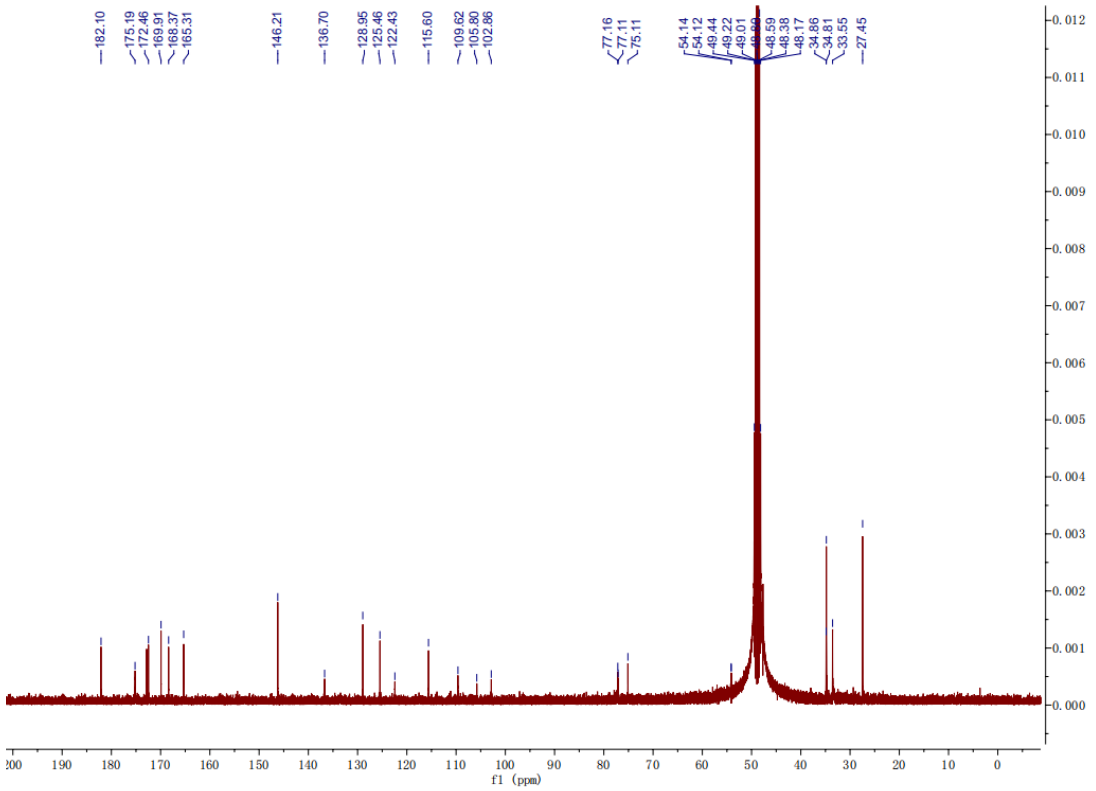

^{1}H-NMR spectrum of **XT34**

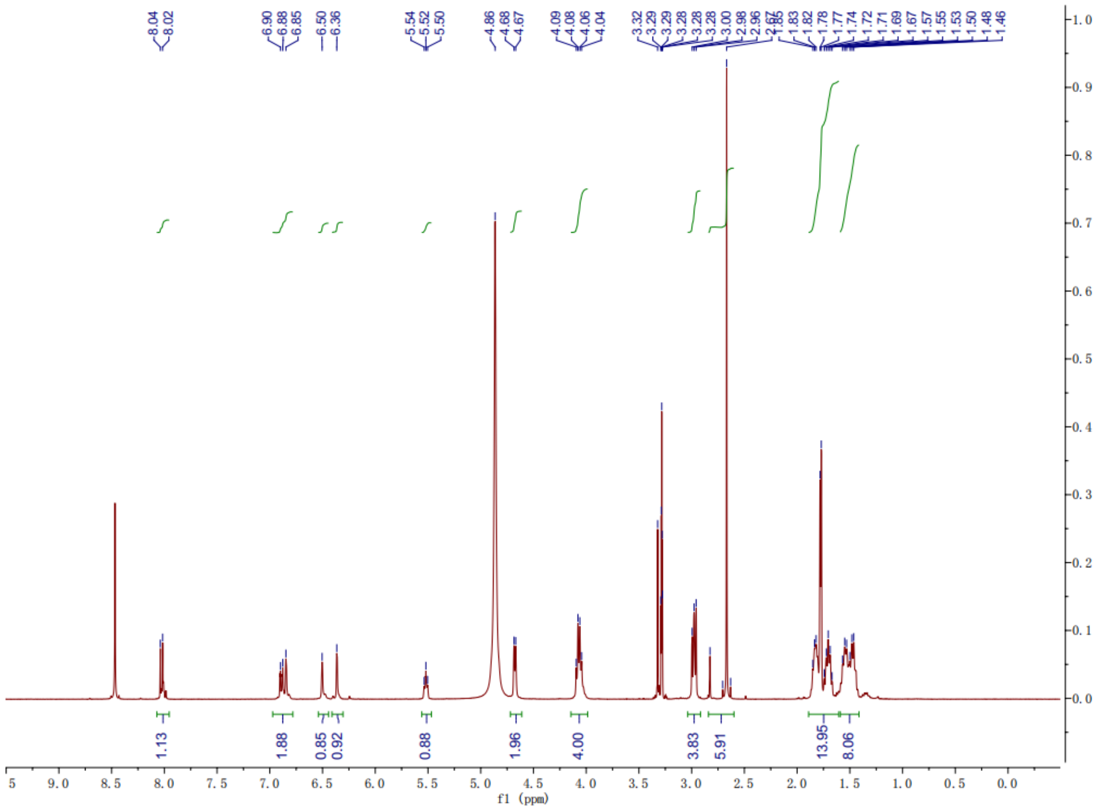

^{13}C-NMR spectrum of **XT34**

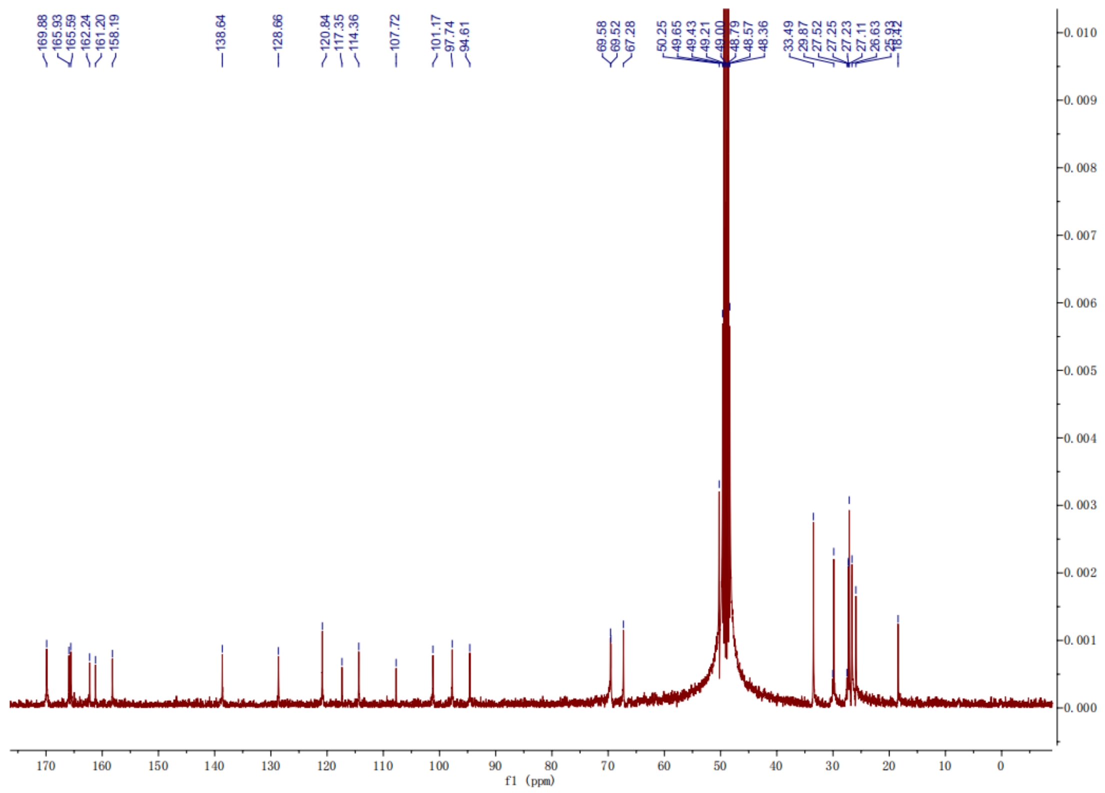

^{1}H-NMR spectrum of **XT35**

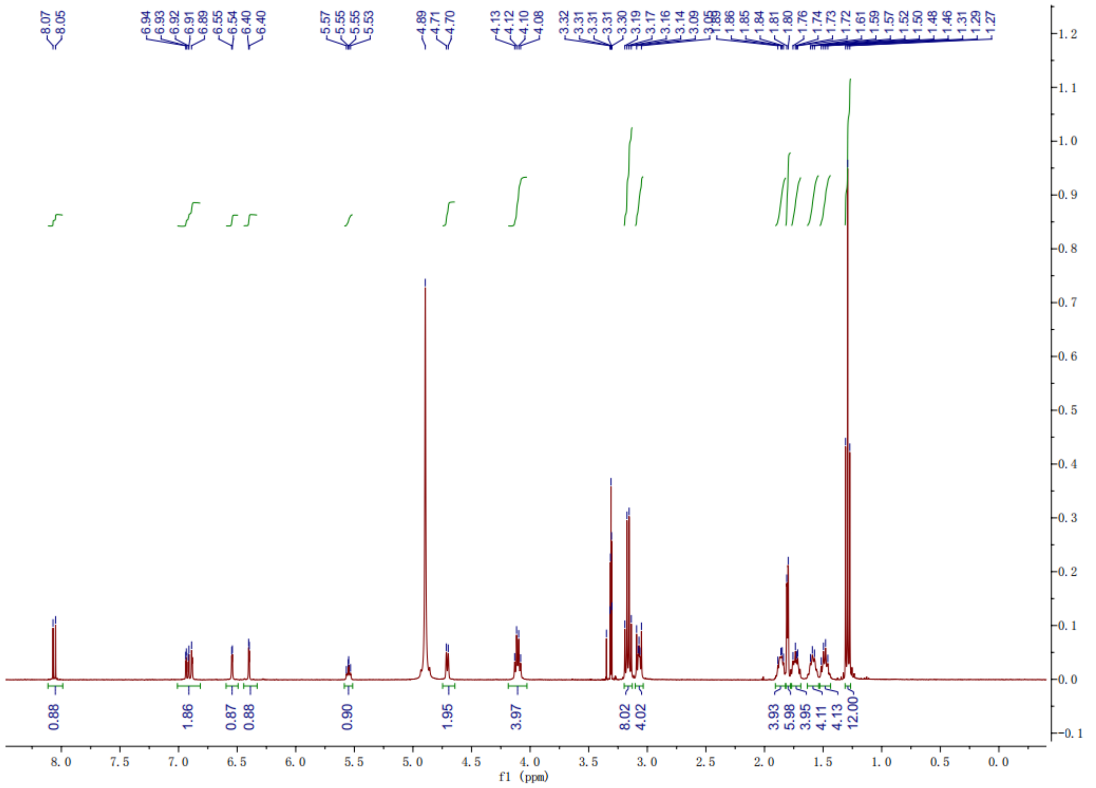

^{13}C-NMR spectrum of **XT35**

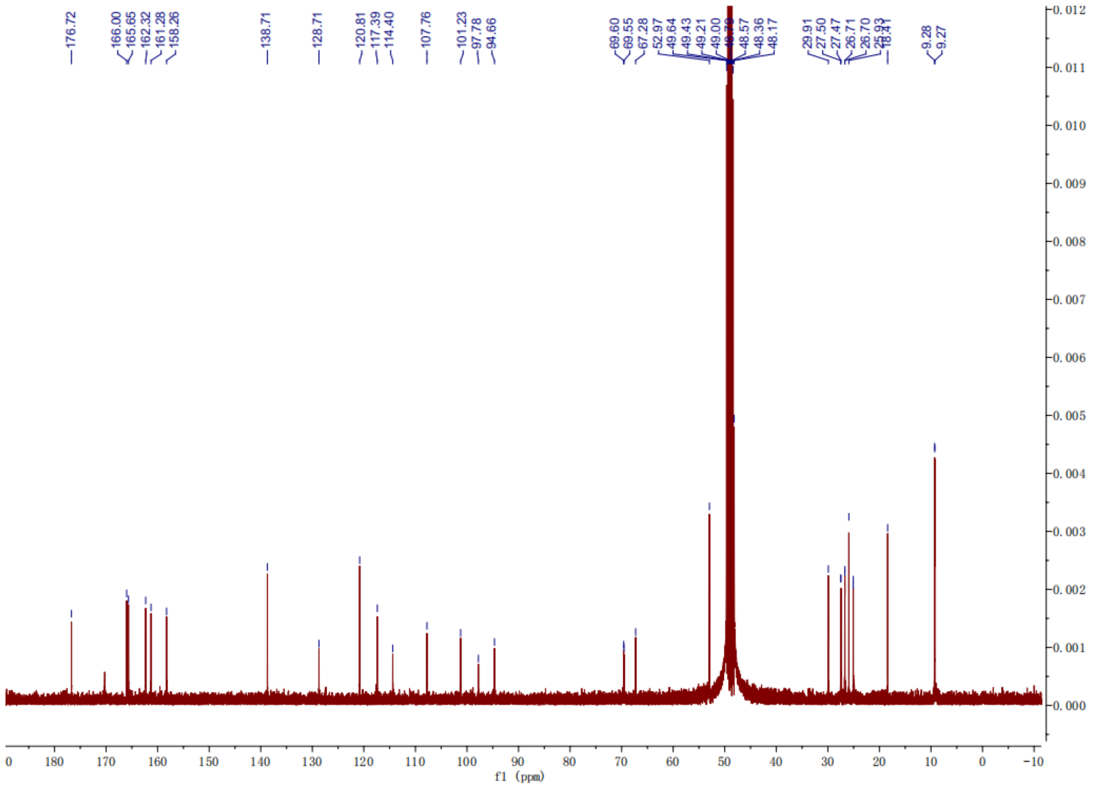

^{1}H-NMR spectrum of **XT37**

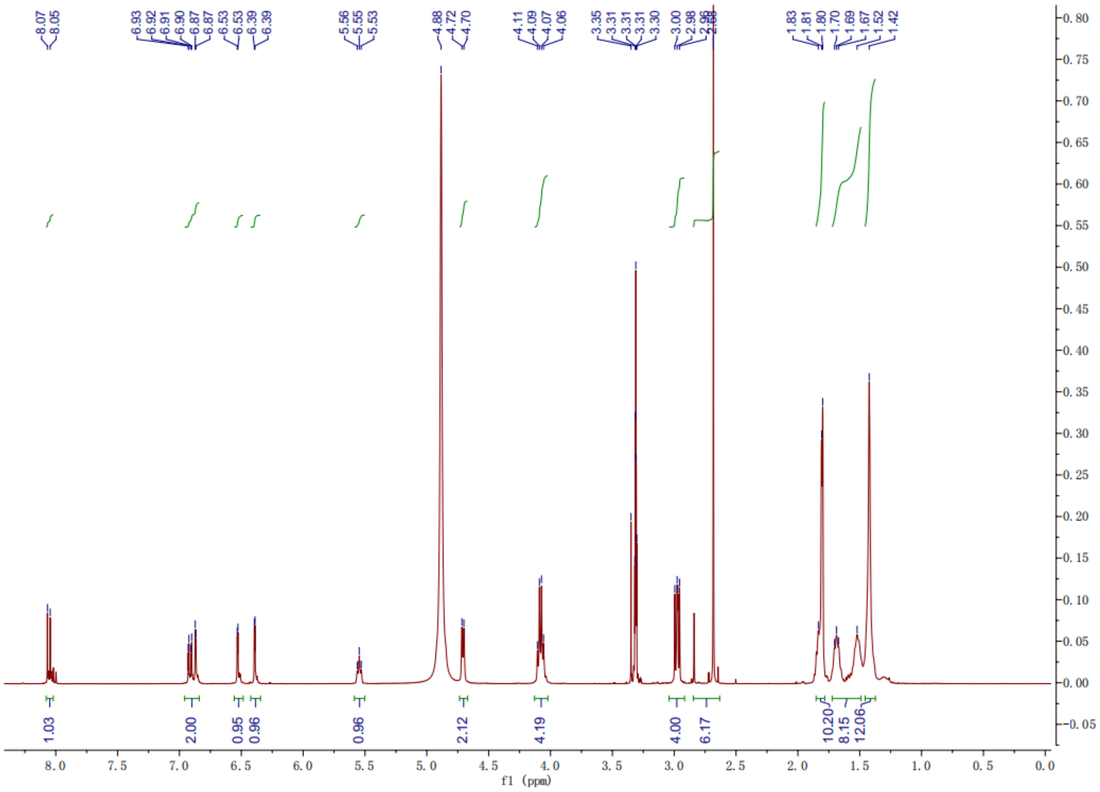

^{13}C-NMR spectrum of **XT37**

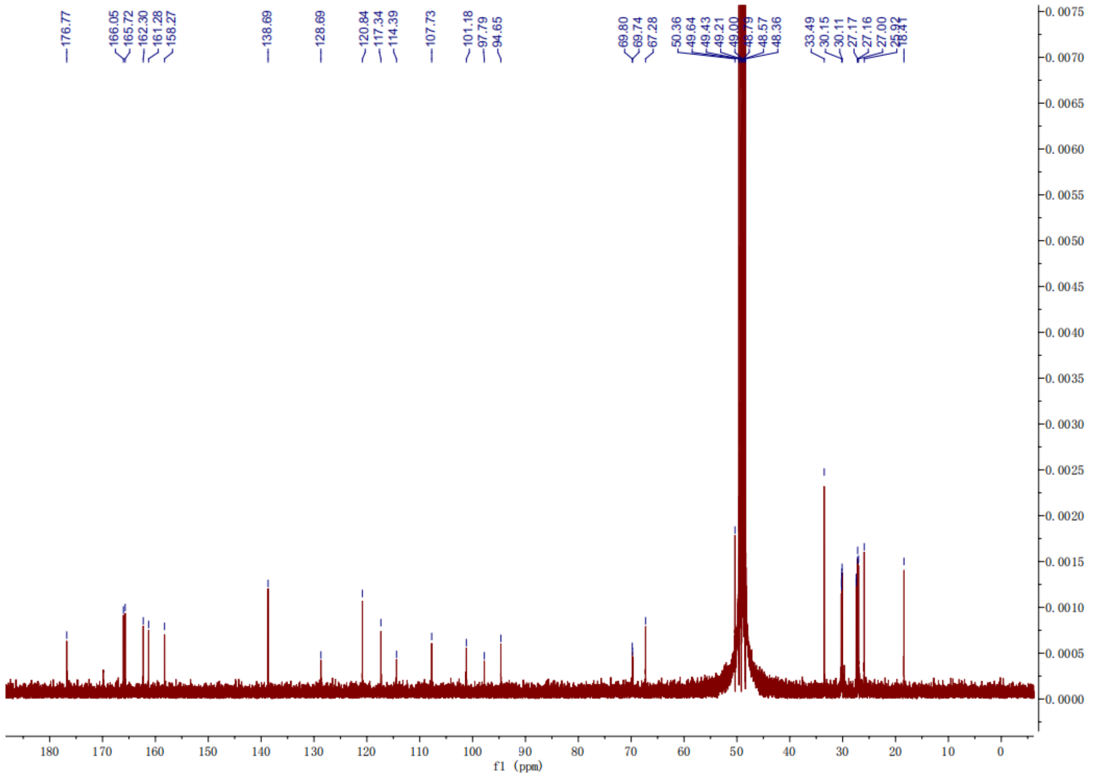

^{1}H-NMR spectrum of **XT38**

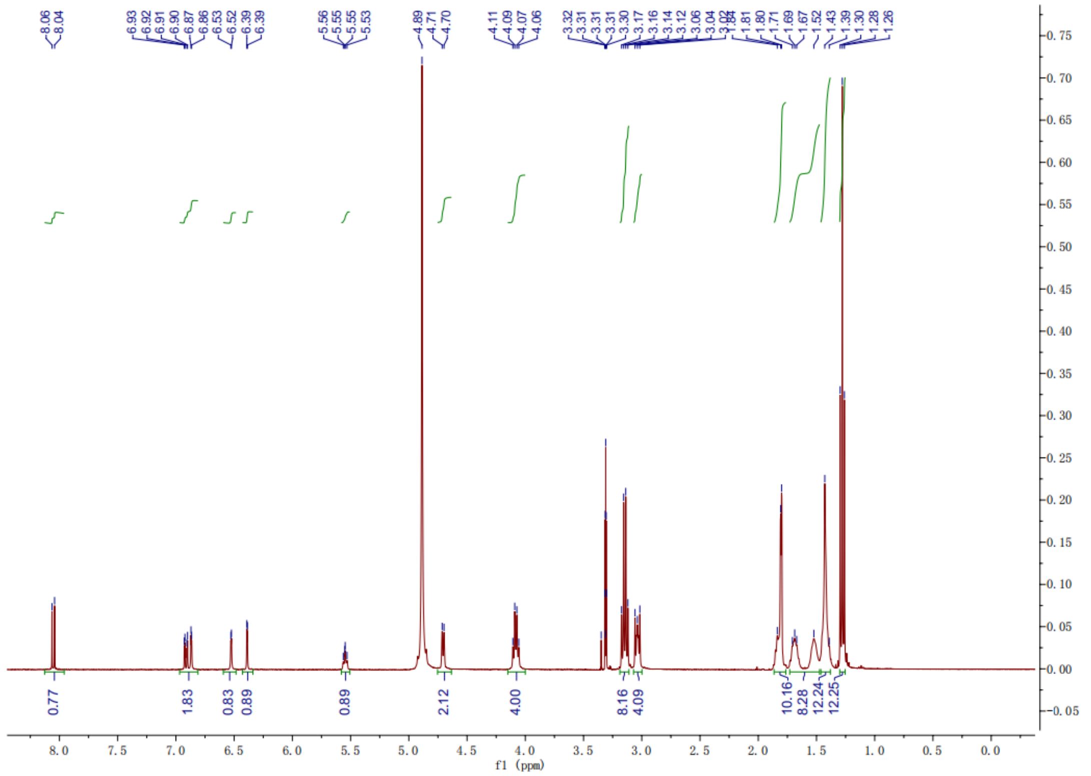

^{13}C-NMR spectrum of **XT38**

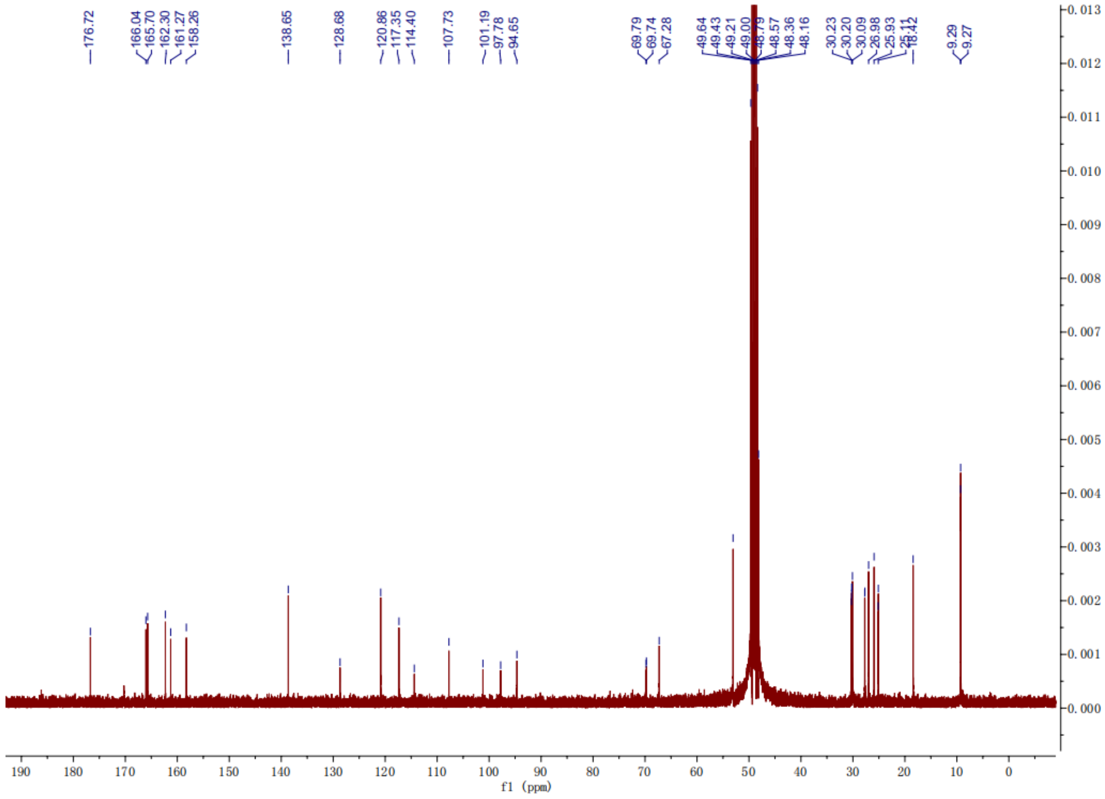

^{1}H-NMR spectrum of **XT40**

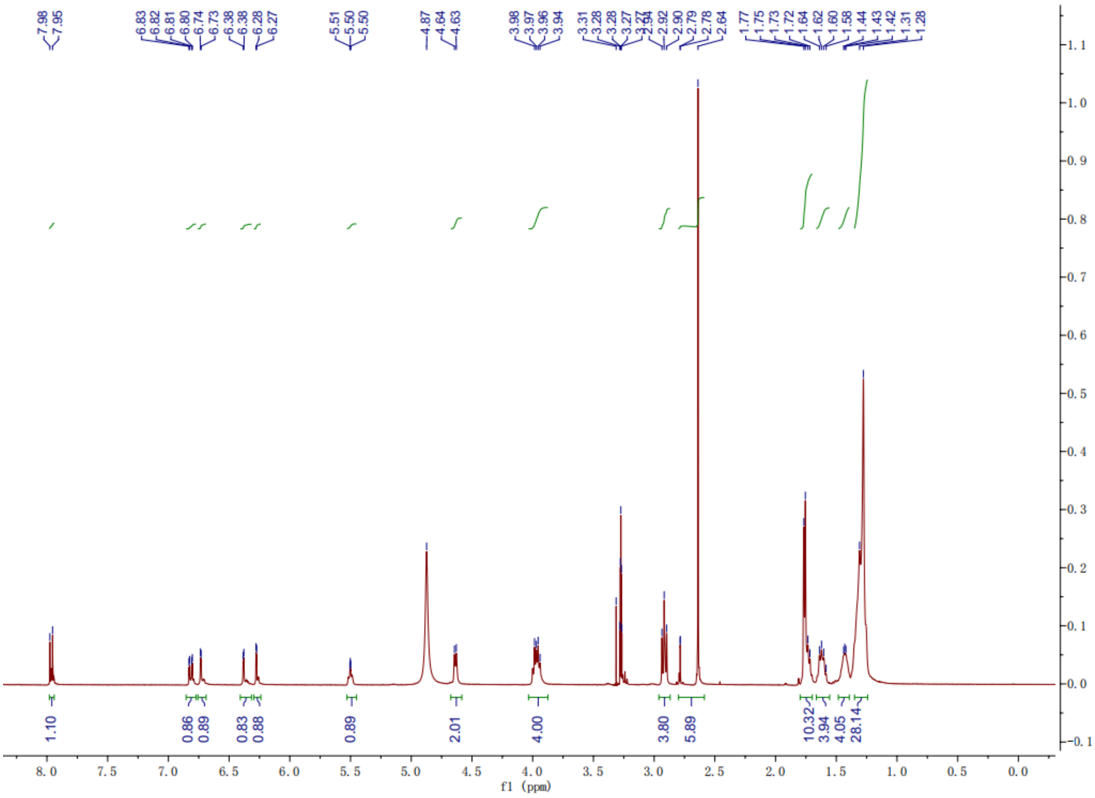

^{13}C-NMR spectrum of **XT40**

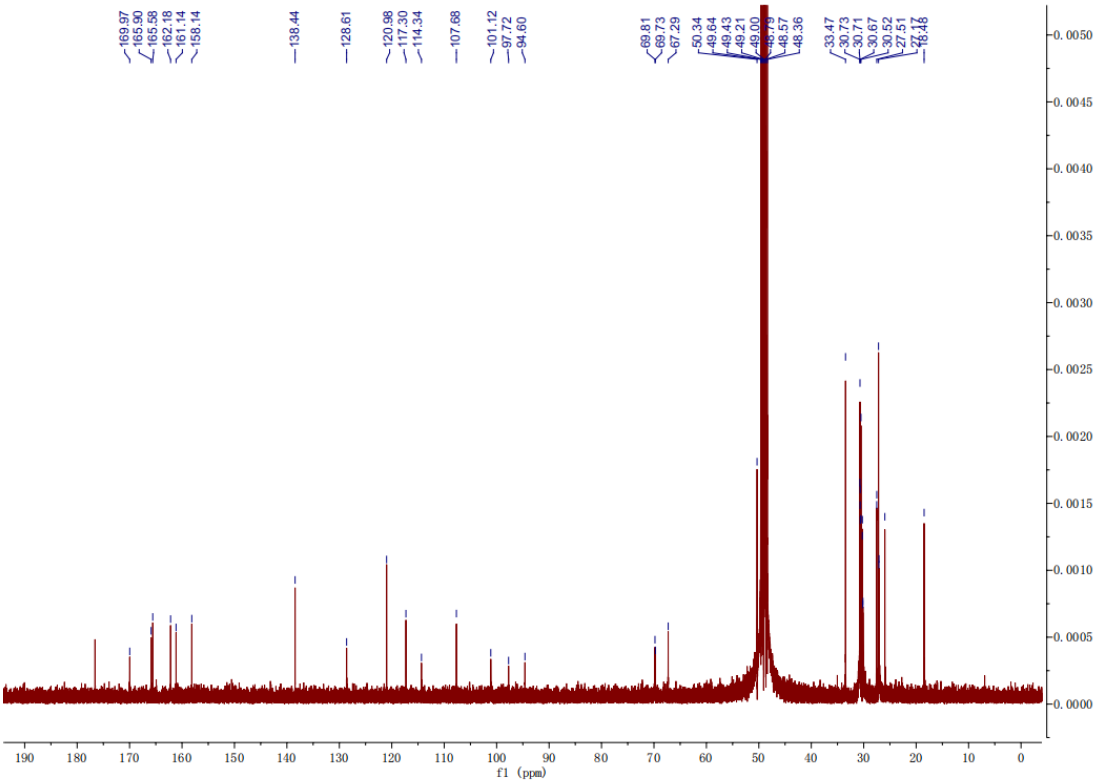

^{1}H-NMR spectrum of **XT41**

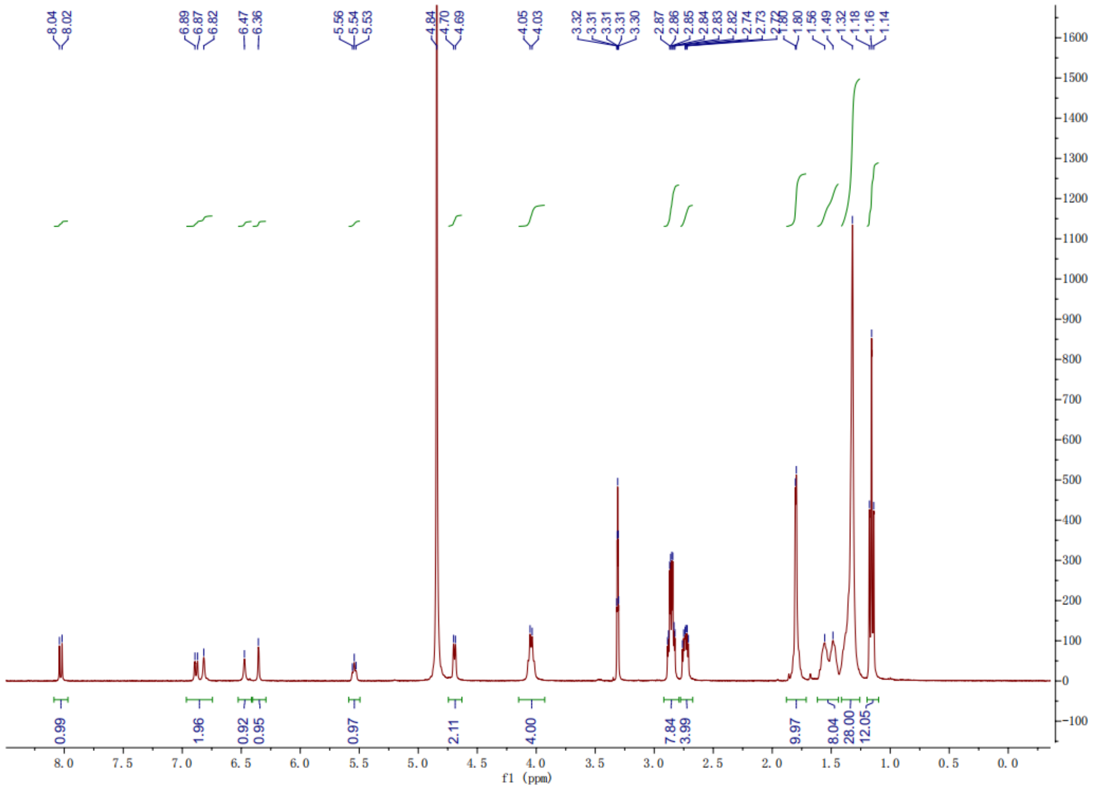

^{13}C-NMR spectrum of **XT41**

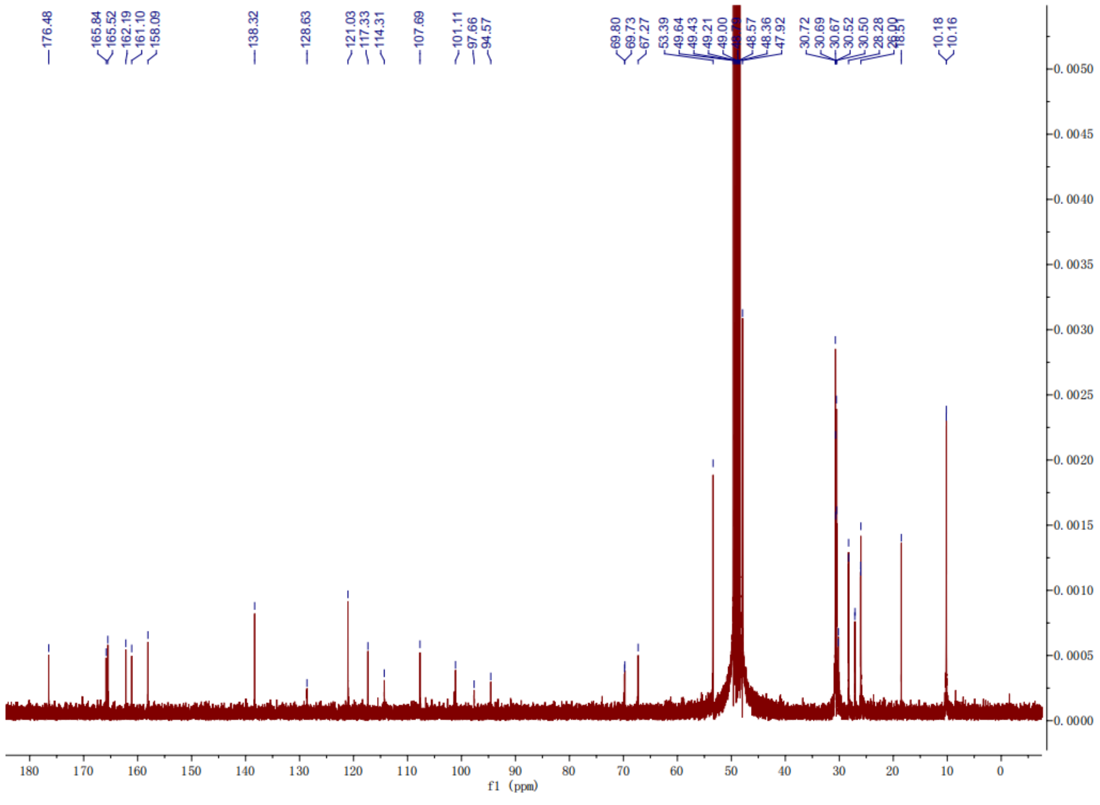

^{1}H-NMR spectrum of **XT42**

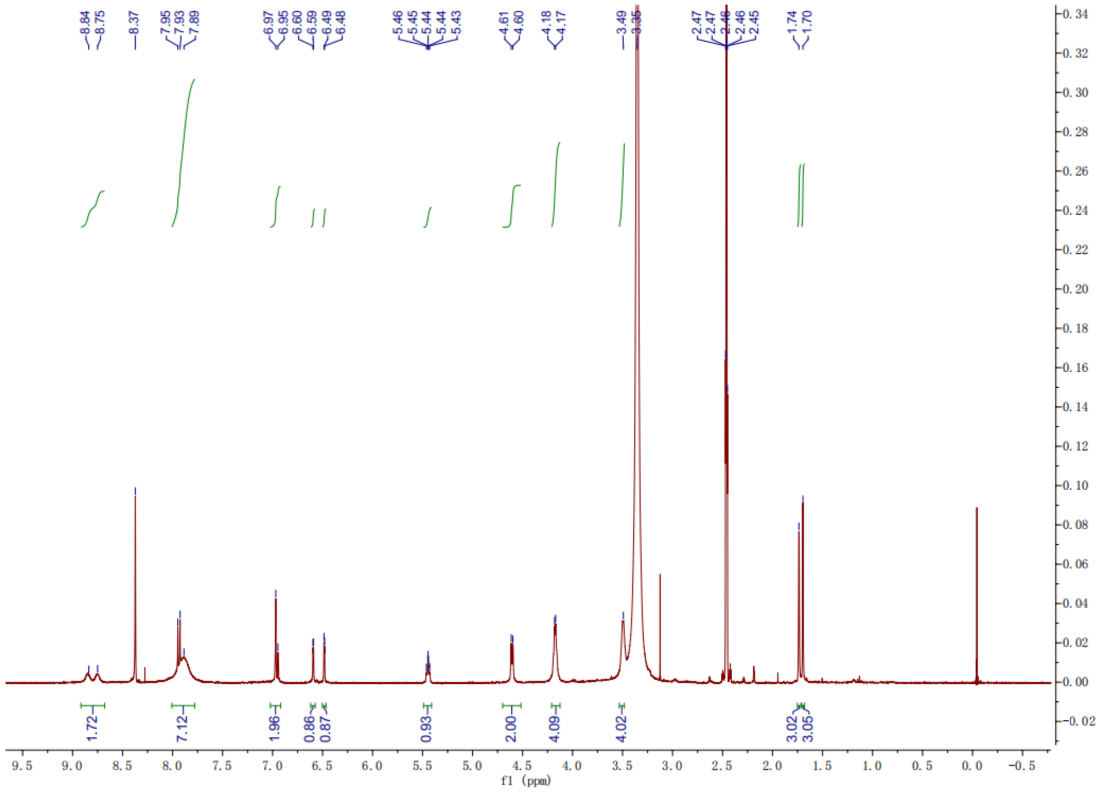

^{13}C-NMR spectrum of **XT42**

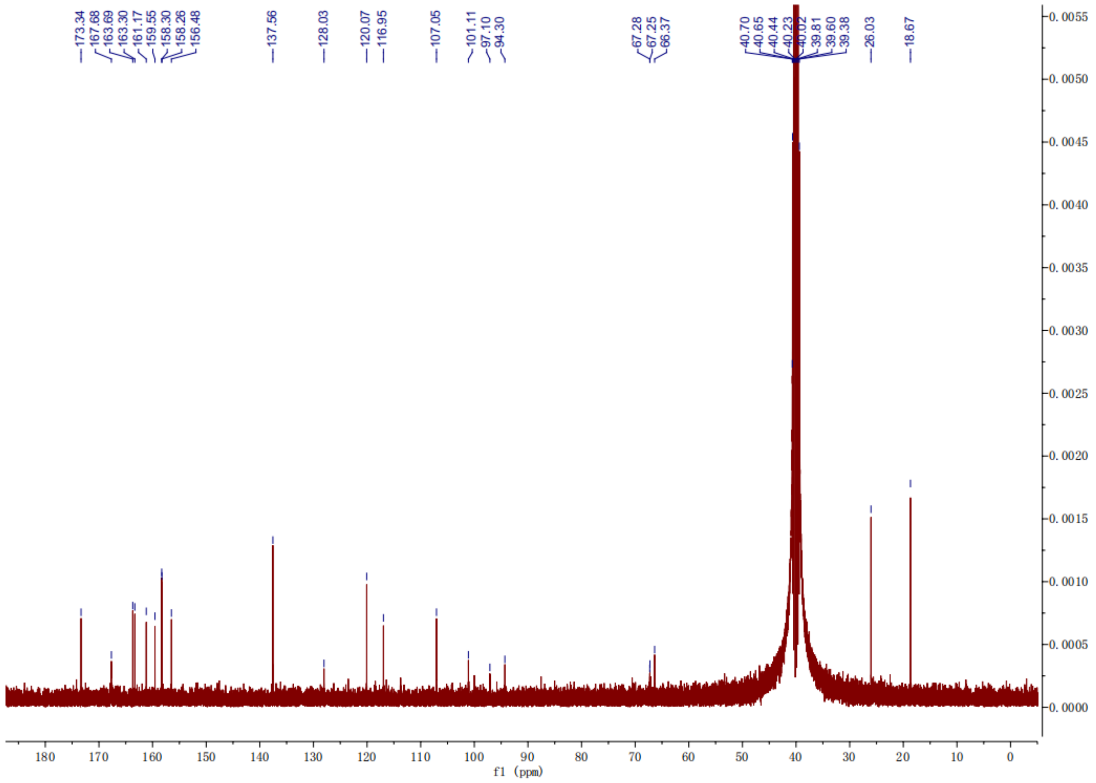

^{1}H-NMR spectrum of **XT43**

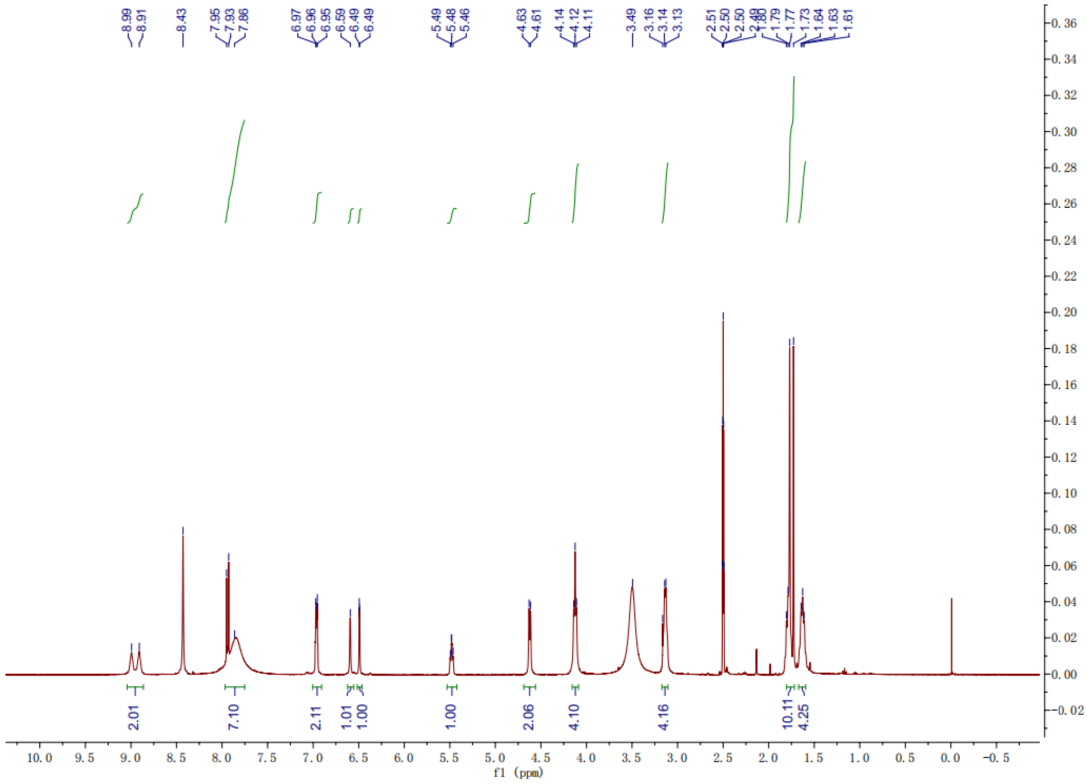

^{13}C-NMR spectrum of **XT43**

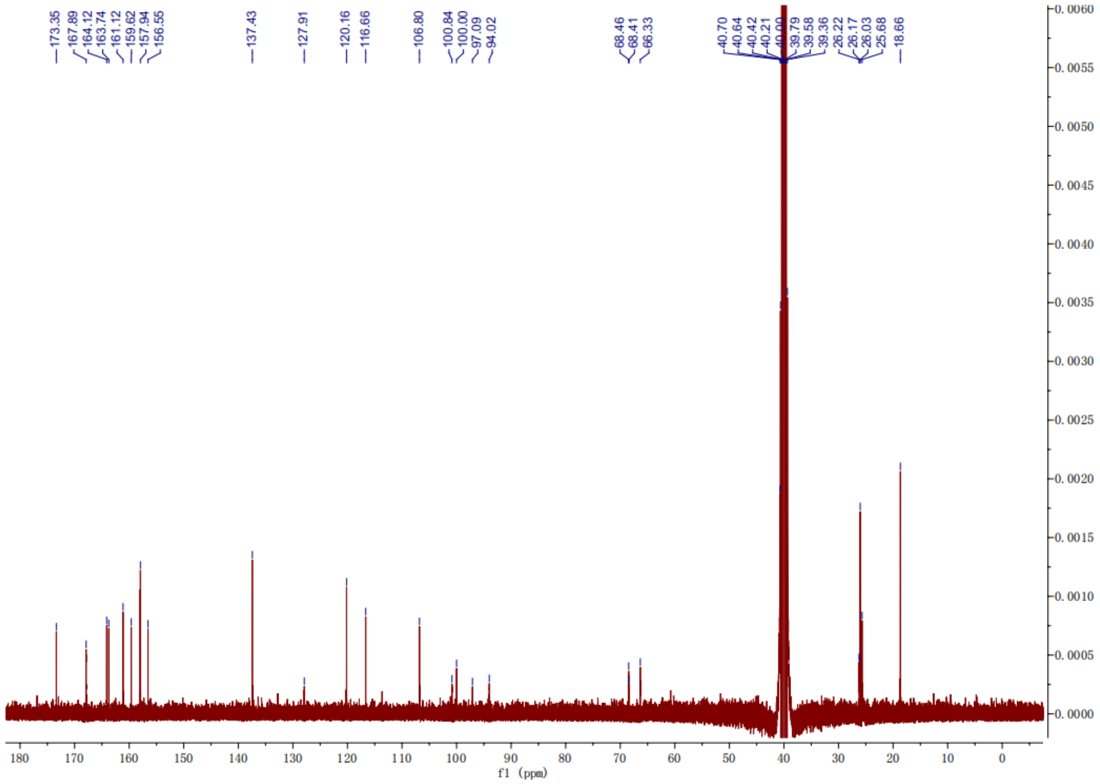

^{1}H-NMR spectrum of **XT44**

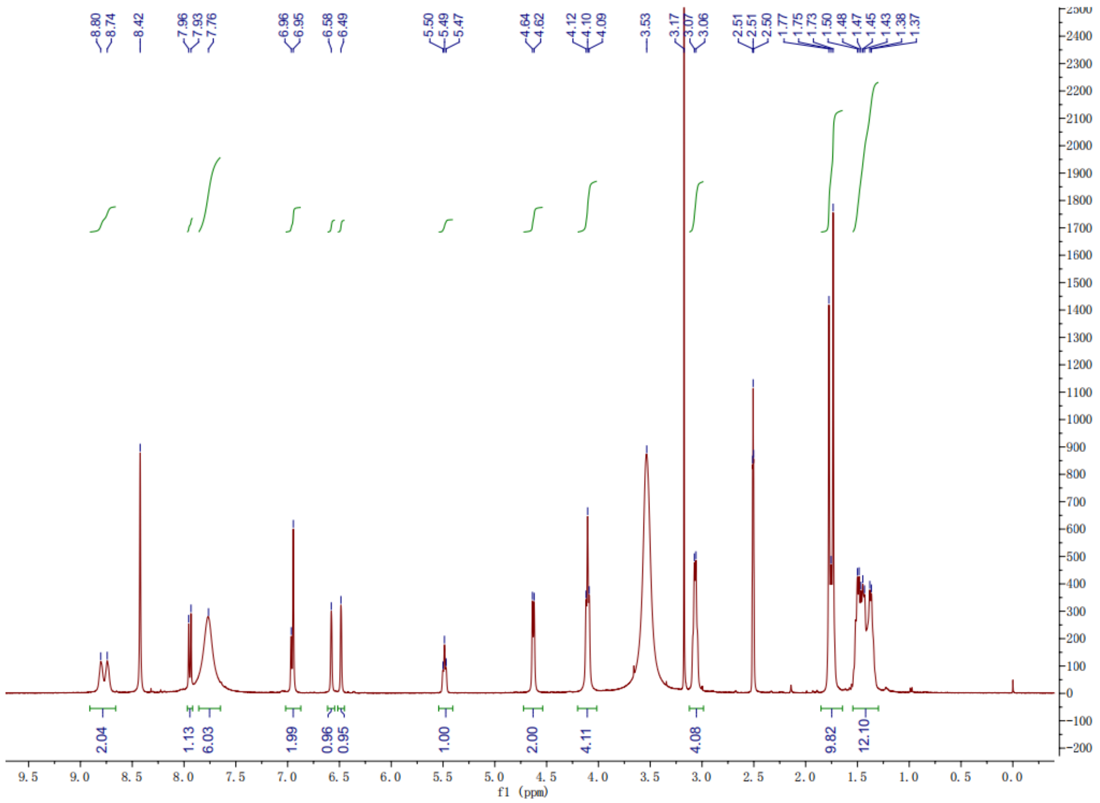

^{13}C-NMR spectrum of **XT44**

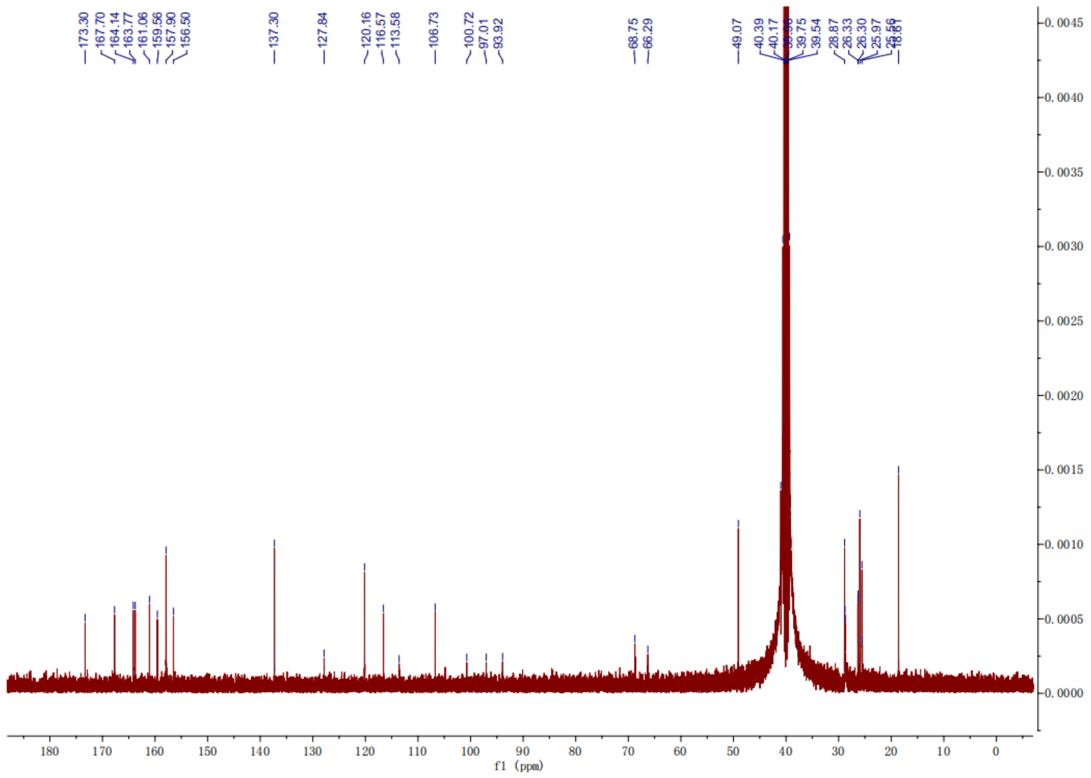

^{1}H-NMR spectrum of **XT45**

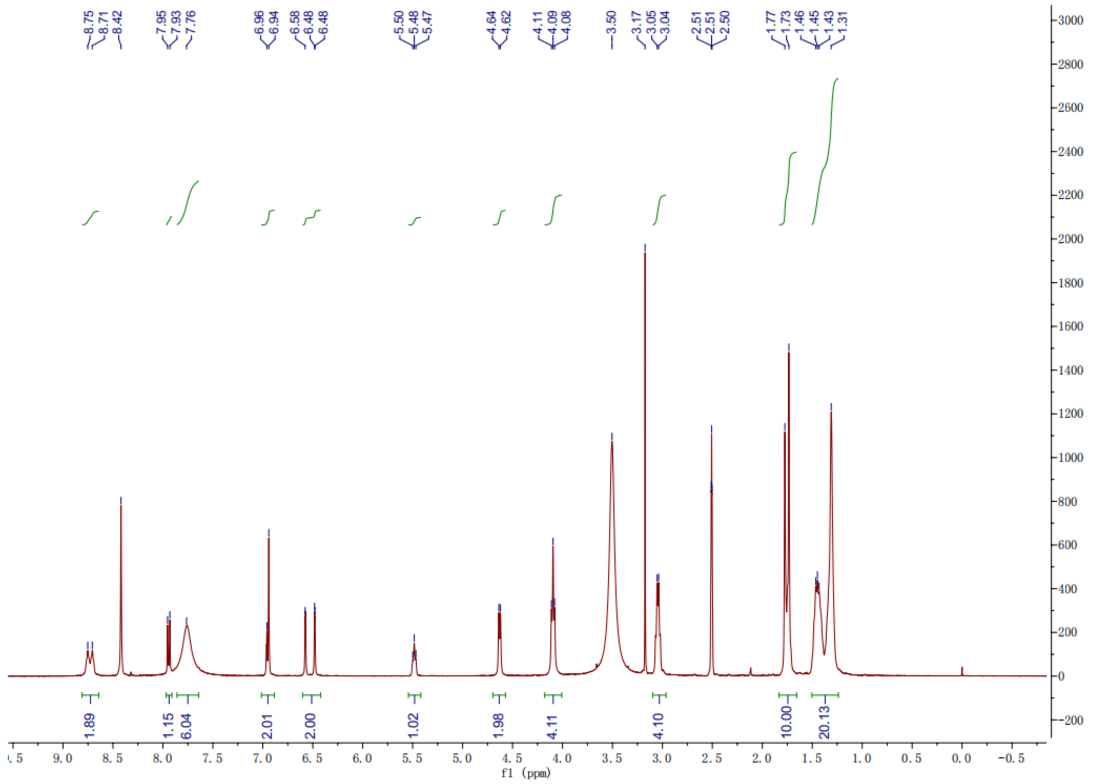

^{13}C-NMR spectrum of **XT45**

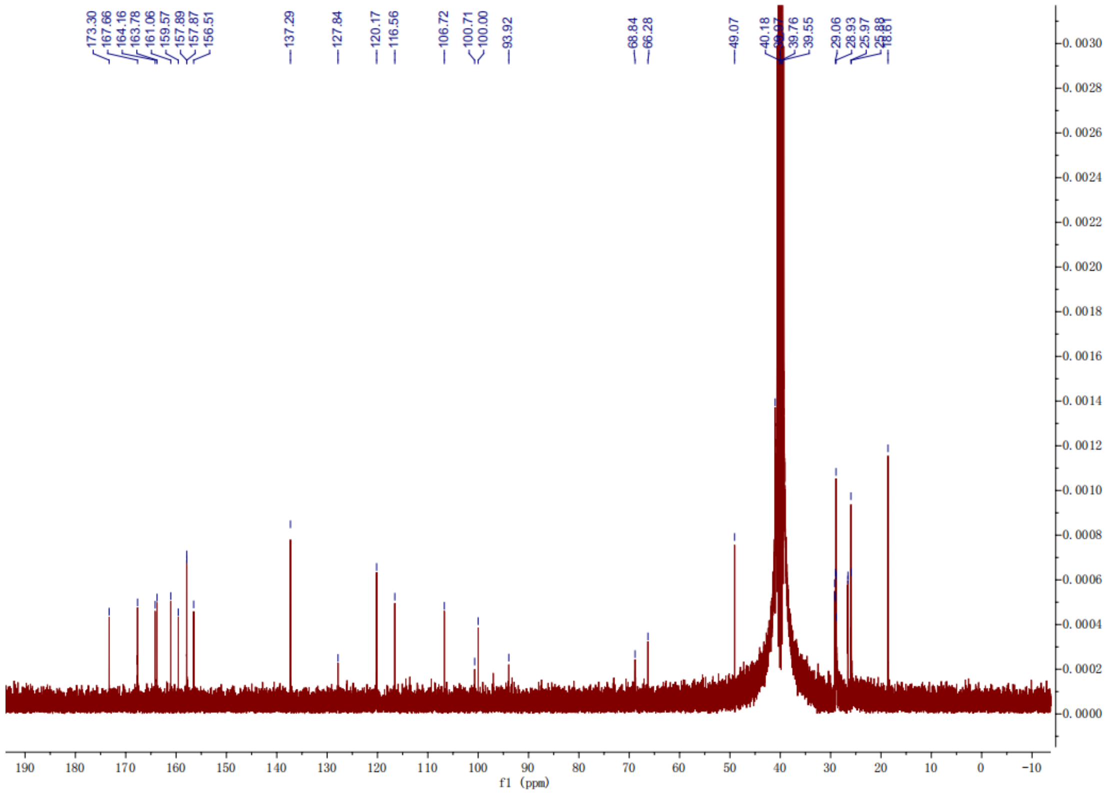

Supplementary figures

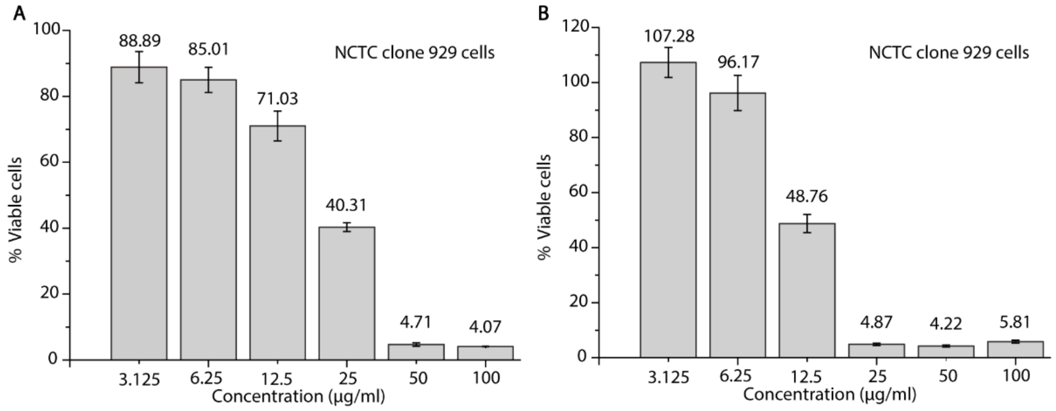

Fig. S1. In vitro cytotoxicity of compounds XT43 (A) and XT44 (B) toward mouse fibroblasts NCTC clone 929 cells using CCK-8 assay. The data are mean ± SD from triplicate experiments.

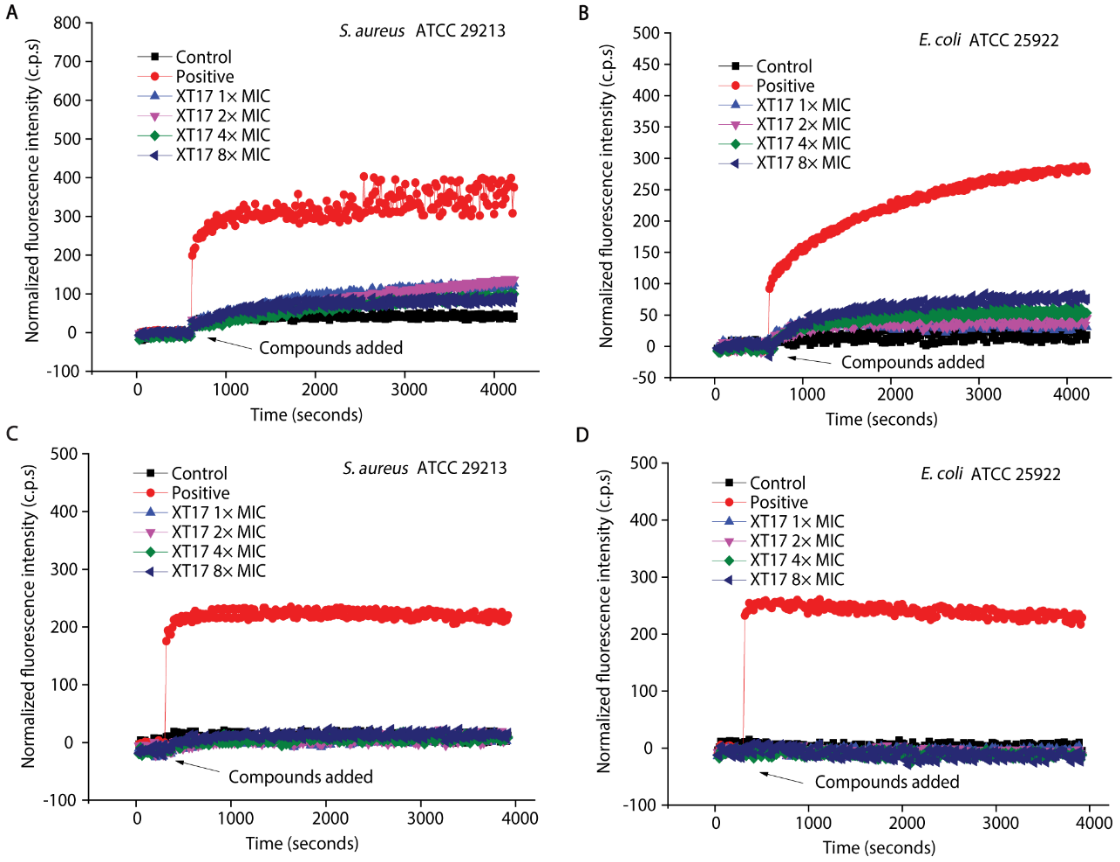

Fig. S2. Membrane binding and permeation ability of compound XT17. (**A** and **B**) Cytoplasmic membrane permeabilization of *S. aureus* ATCC 29213 (A) and *E. coli* ATCC 25299 (B) treated with compound **XT17** at different concentrations (1×, 2×, 4× and 8× MIC) by SYTOX Green uptake assay; 1% Triton X-100 served as a positive control. (**C** and **D**) Membrane depolarization of *S. aureus* ATCC 29213 (C) and *E. coli* ATCC 25299 (D) treated with compound **XT17** at different concentrations (1×, 2×, 4× and 8× MIC) via the $DiSC_3(5)$ dye assay; 1% Triton X-100 served as a positive control.

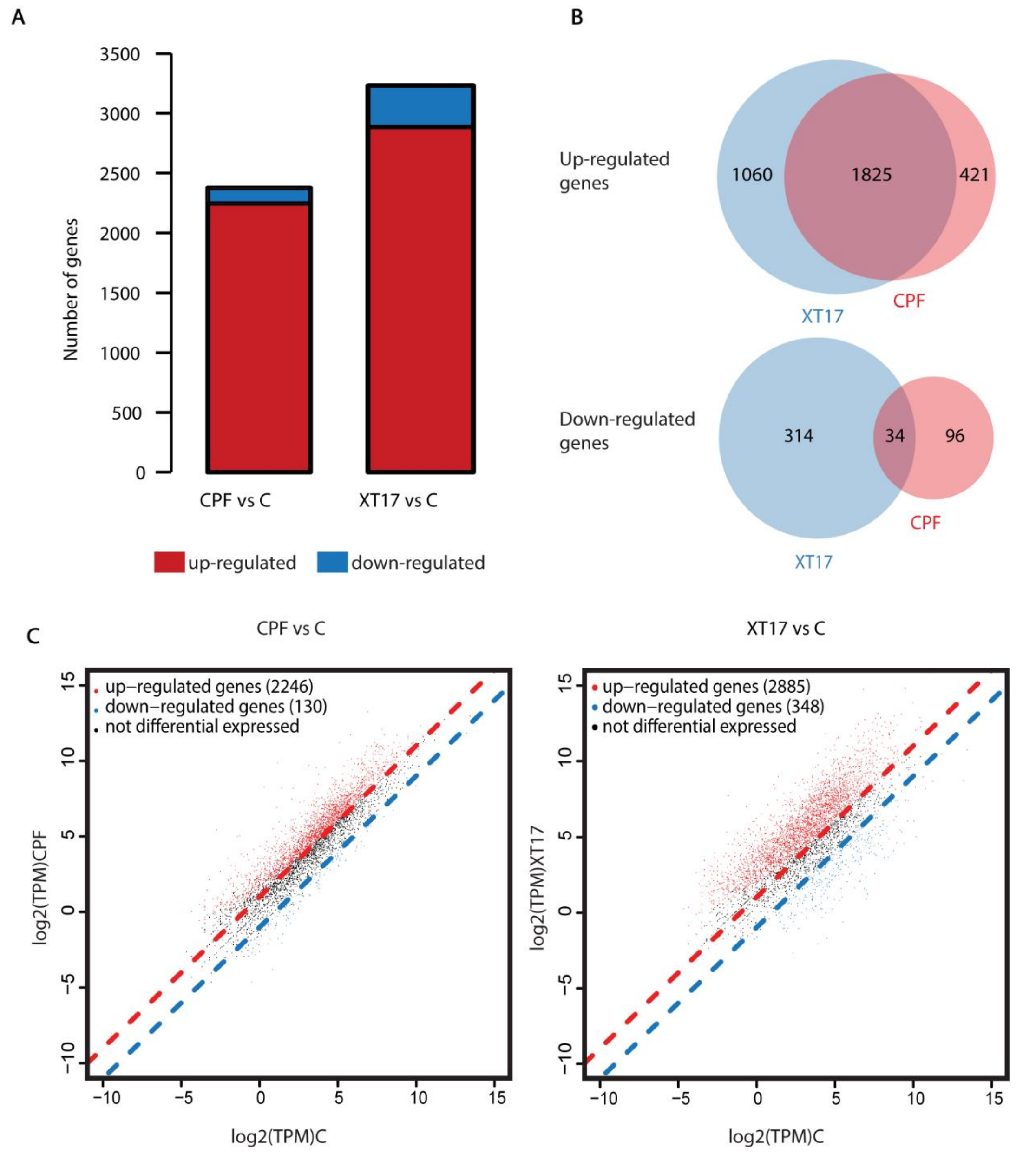

Fig. S3. Differentially expressed genes (DEG) analysis of RNA-seq datasets. (**A**) Bar plot representing the number of up- and down-regulated genes in both CPF- and compound **XT17**-treated samples compared to the control. (**B**) Venn diagram displaying the overlap of up- and down-regulated genes between CPF- and compound **XT17**-treated groups. (**C**) Scatter plot encoding the DEGs in all samples. Scale represents $\log_2$ TPM (Transcript per million).

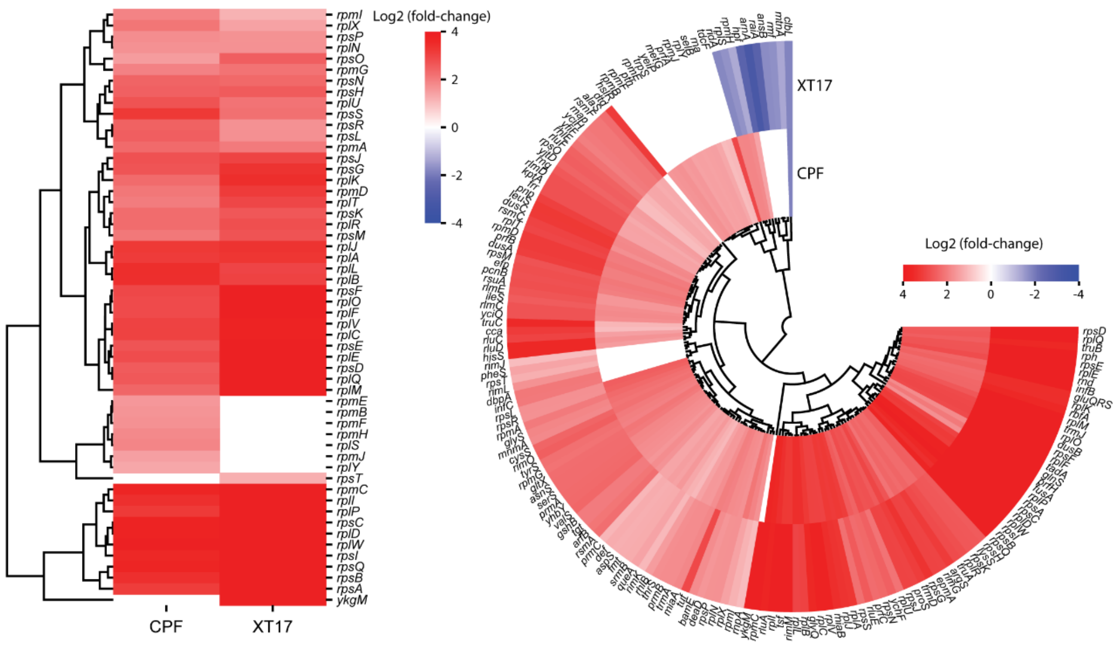

Fig. S4. Transcriptome response to ribosome using KEGG enrichment analysis (left panel), and response to translation, ribosomal structure, and biogenesis using COG analysis (right panel). We identified significant functionally enriched gene expression response of *E. coli* ATCC 25922 treated with CPF and compound **XT17**, respectively. Heatmap displayed the significant DEGs ($\log_2$ fold-change) with the expression level color patterned of red for up-regulated genes and green for down-regulated genes.

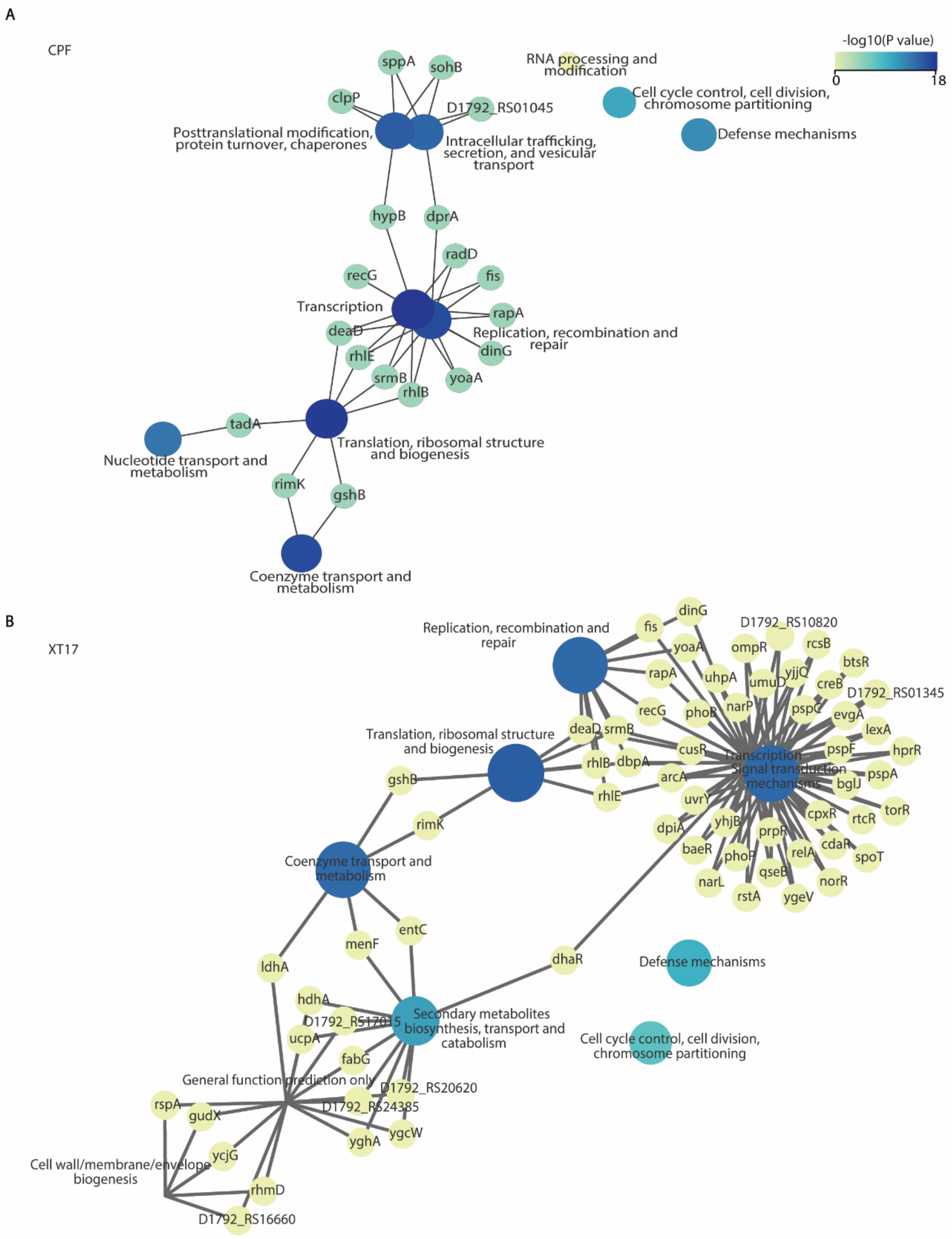

Fig. S5. Interaction network analysis based on COG. (**A** and **B**) The network nodes represent the proteins encoded by the DEGs of *E. coli* ATCC 25922 upon CPF- (A) and compound **XT17**-treated (B). The associations are meant to be specific and meaningful with associated proteins jointly contributing to a shared function.

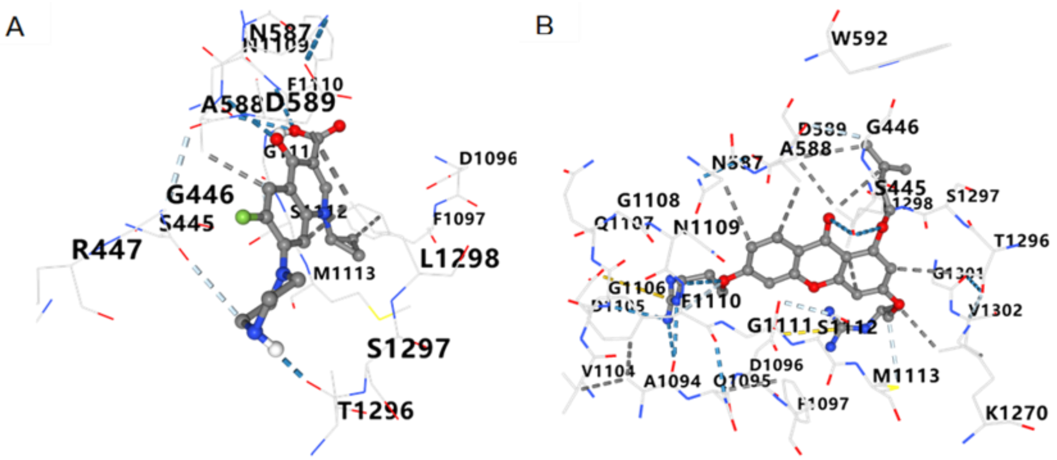

Fig. S6. 3D-binding interactions diagram for the complex protein-ligand. Docking interaction of CPF (**A**) and compound **XT17** (**B**) with DNA gyrase from *S. aureus* (PDB code: 2XCT).

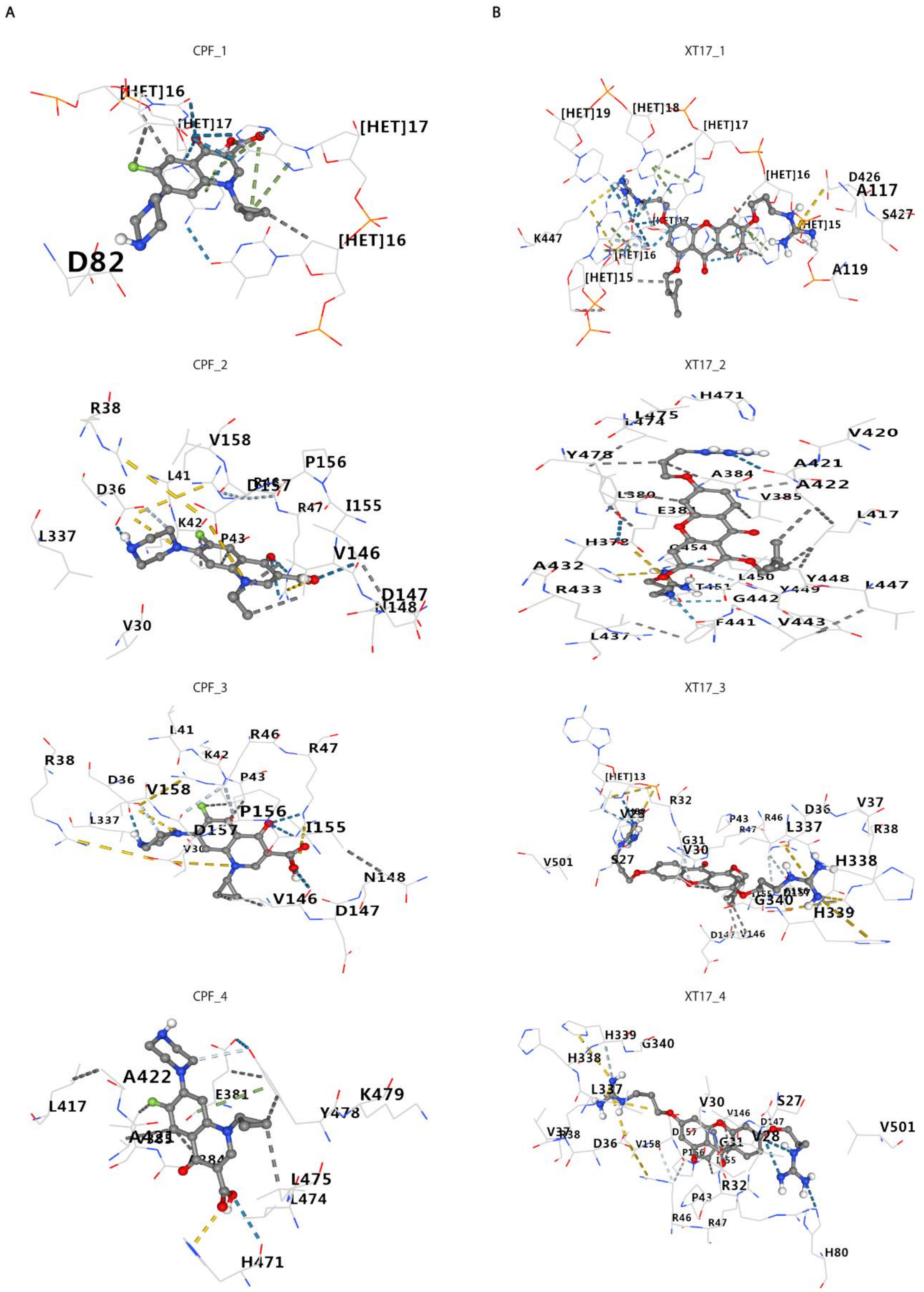

Fig. S7. The binding site of interacting residues with ligands after docking. (**A**) The 3D intermolecular contact between CPF and DNA gyrase from *E. coli* (PDB code: 6RKV). (**B**) The 3D intermolecular contact between compound **XT17** and 6RKV. Chemical structures were drawn by ChemDraw Pro 16.0 Suite (PerkinElmer, USA).

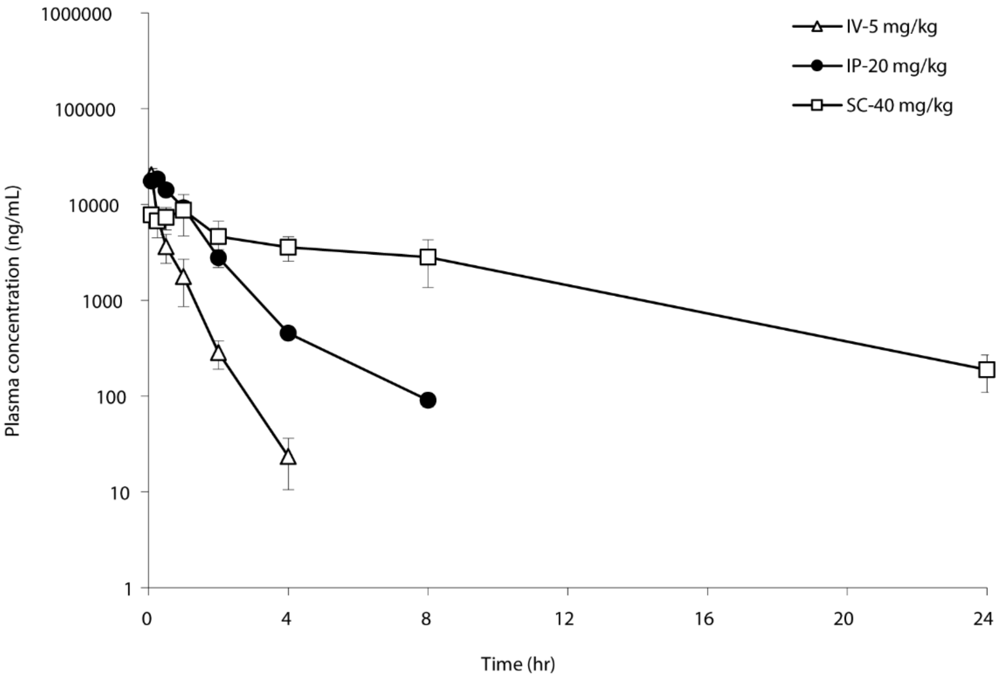

Fig. S8. Mean plasma concentration-time profiles of XT17 after single IV, IP and SC administrations in CD-1 mice (n = 3).

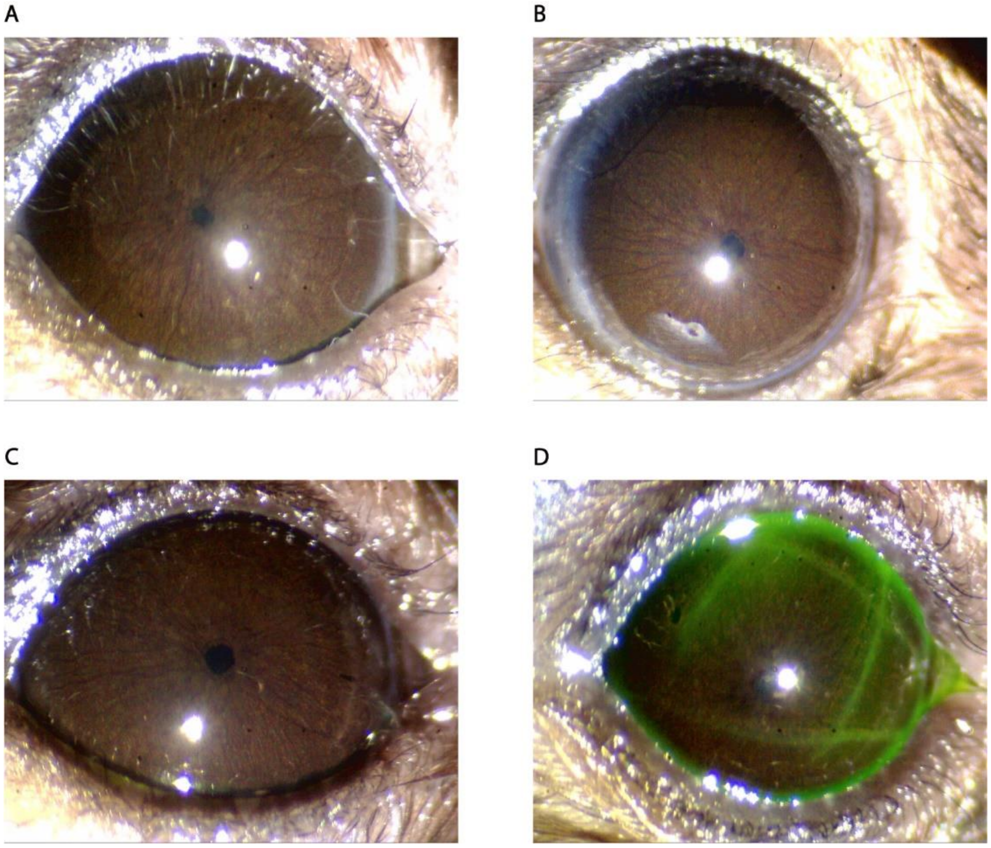

Fig. S9. In vivo toxicity of compound XT17 toward murine cornea by topical administration. (**A**) The cornea of the mouse was treated with a 5% glucose solution. (**B**) The cornea of the mouse was treated with 0.5% compound **XT17**. (**C**) The cornea of the mouse was treated with 1.5% compound **XT17**. (**D**) The cornea of the mouse was scratched with a sterile needle. Representative slit-lamp photographs for each group are shown (n = 3).

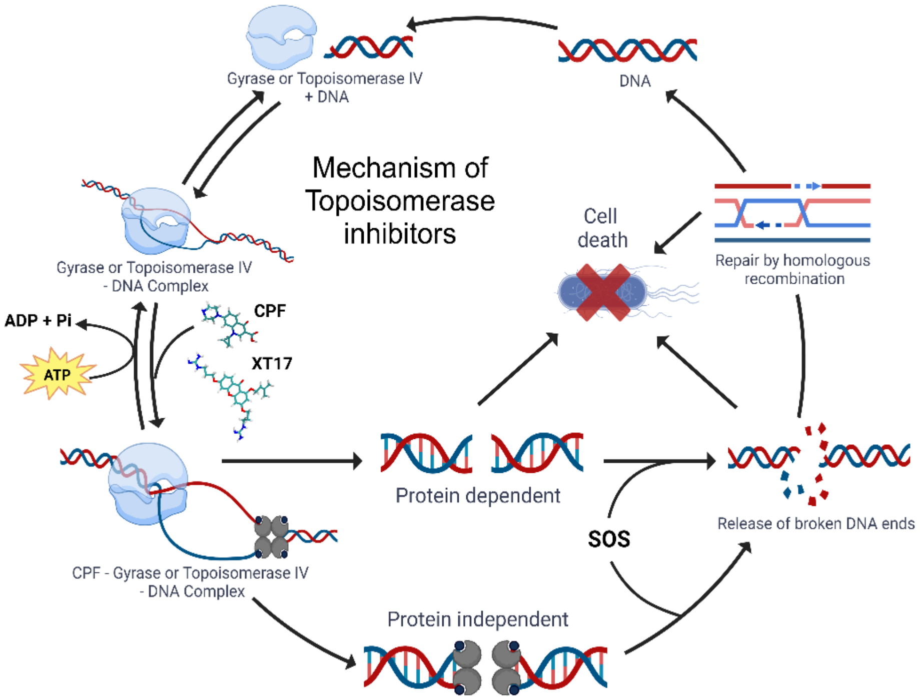

Fig. S10. Diagrammatic representation of topoisomerase inhibitors mechanism. Compound **XT17** target DNA gyrase and topoisomerase IV binding to specific domains and conformations as CPF in order to block DNA strand passage catalysis and stabilize DNA-enzyme complexes. These antibacterial agents have the ability to block the DNA replication apparatus and generate double breaks in DNA that underlie their bactericidal activity.

3. HPLC validation of xanthone derivatives.

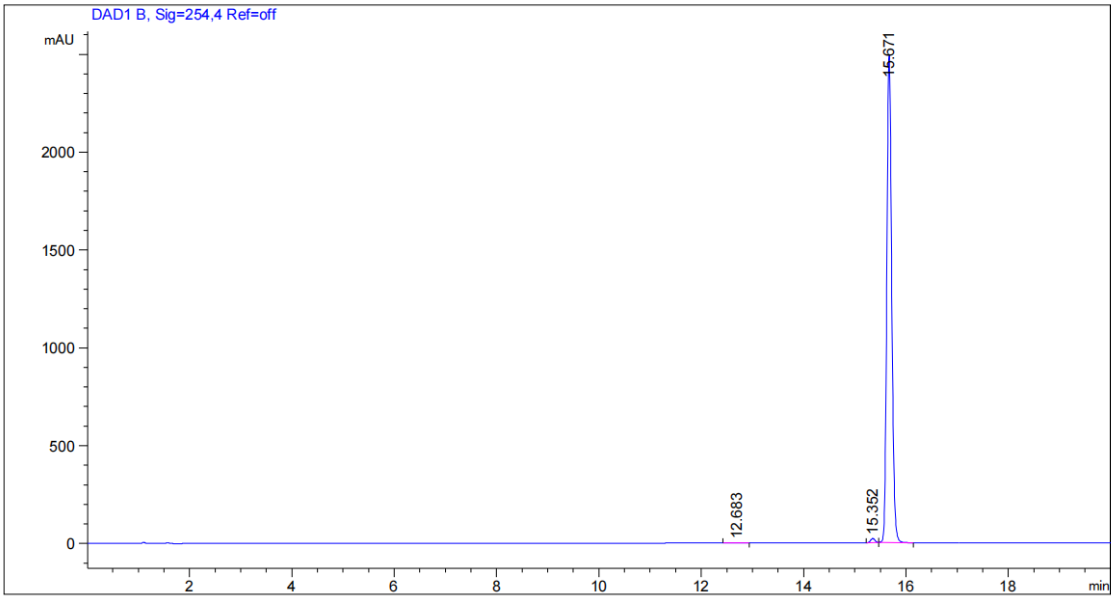

Peak #	RT [min]	Type	Width [min]	Area [mAU*s]	Height [mAU]	Area %
1	12.683	BB	0.1080	14.42894	2.00424	0.0867
2	15.352	BB	0.0876	119.44010	21.23061	0.7178
3	15.671	BB	0.1034	1.65068e4	2488.97363	99.1955

Fig. S11. HPLC validation of compound **XT01**.

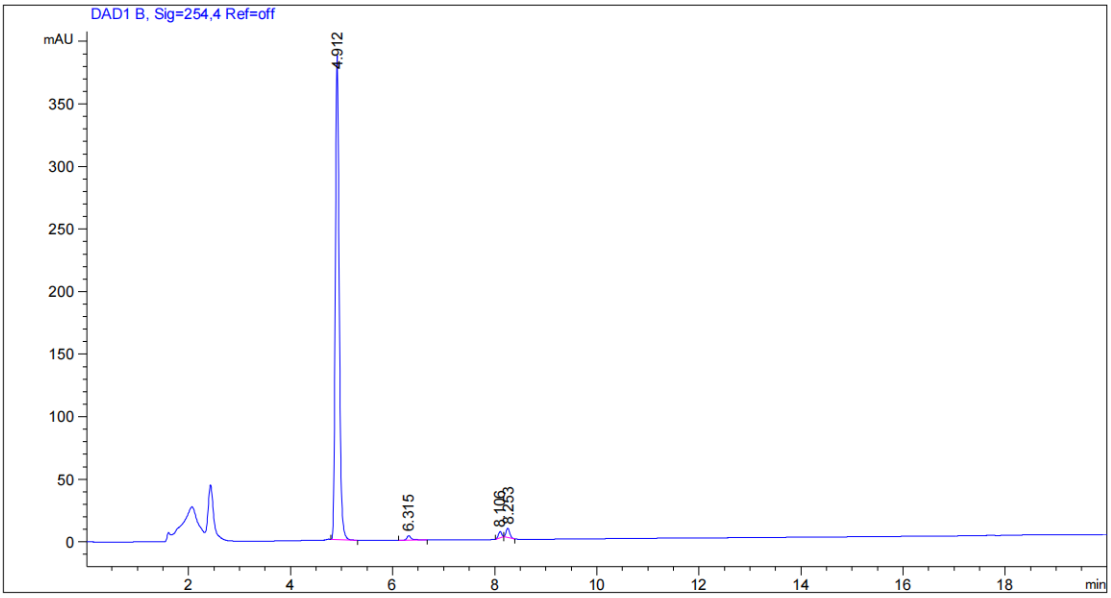

Peak #	RT [min]	Type	Width [min]	Area [mAU*s]	Height [mAU]	Area %
1	4.912	BB	0.0792	2030.55774	386.90891	96.1310
2	6.315	BB	0.0969	23.62749	3.58360	1.1186
3	8.106	BB	0.0699	22.13774	4.98058	1.0480
4	8.253	BB	0.0767	35.95926	7.39306	1.7024

Fig. S12. HPLC validation of compound **XT07**.

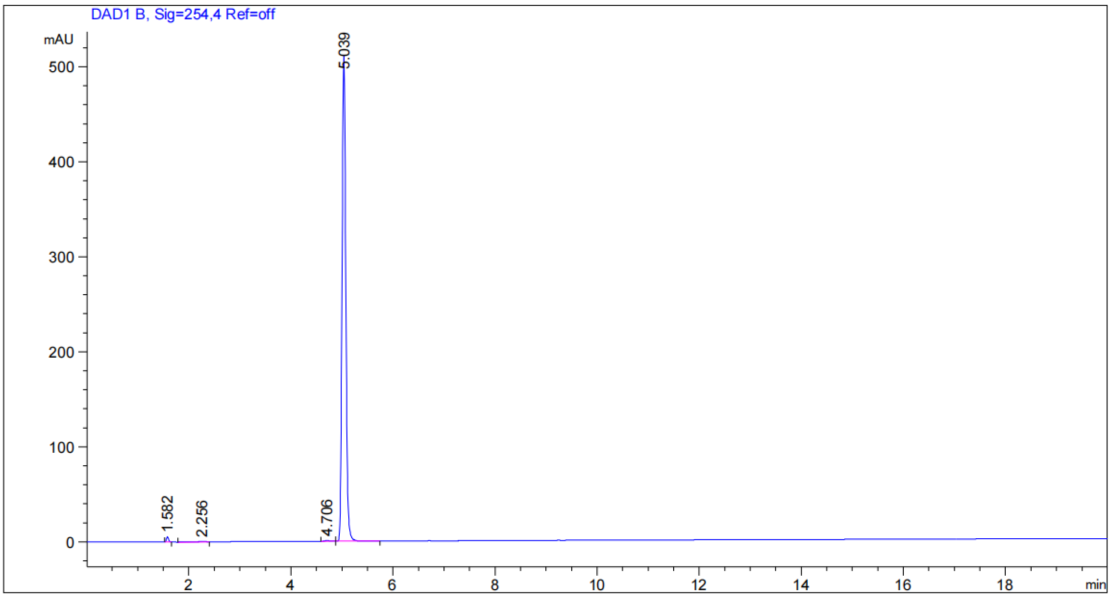

Peak #	RT [min]	Type	Width [min]	Area [mAU*s]	Height [mAU]	Area %
1	1.582	BB	0.0442	14.48051	5.24689	0.5488
2	2.256	BB	0.2404	9.52293	5.23141e-1	0.3609
3	4.706	BB	0.1001	5.52659	8.47455e-1	0.2094
4	5.039	BB	0.0795	2609.16284	511.71884	98.8809

Fig. S13. HPLC validation of compound **XT08**.

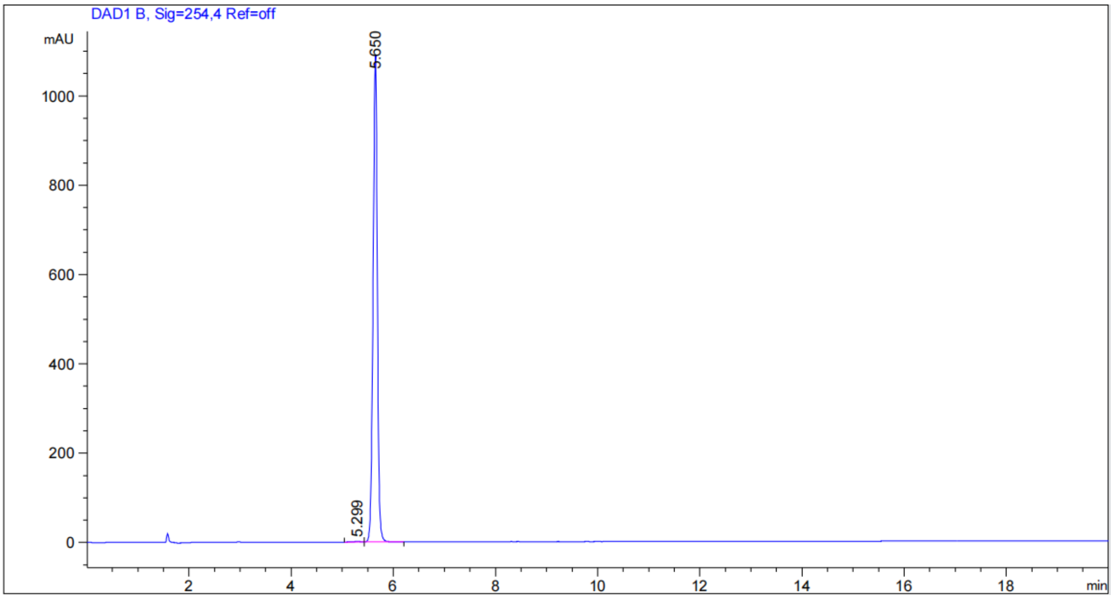

Peak #	RT [min]	Type	Width [min]	Area [mAU*s]	Height [mAU]	Area %
1	5.299	BB	0.1155	13.58097	1.77105	0.2129
2	5.650	BB	0.0921	6364.06592	1090.09949	99.7871

Fig. S14. HPLC validation of compound **XT09**.

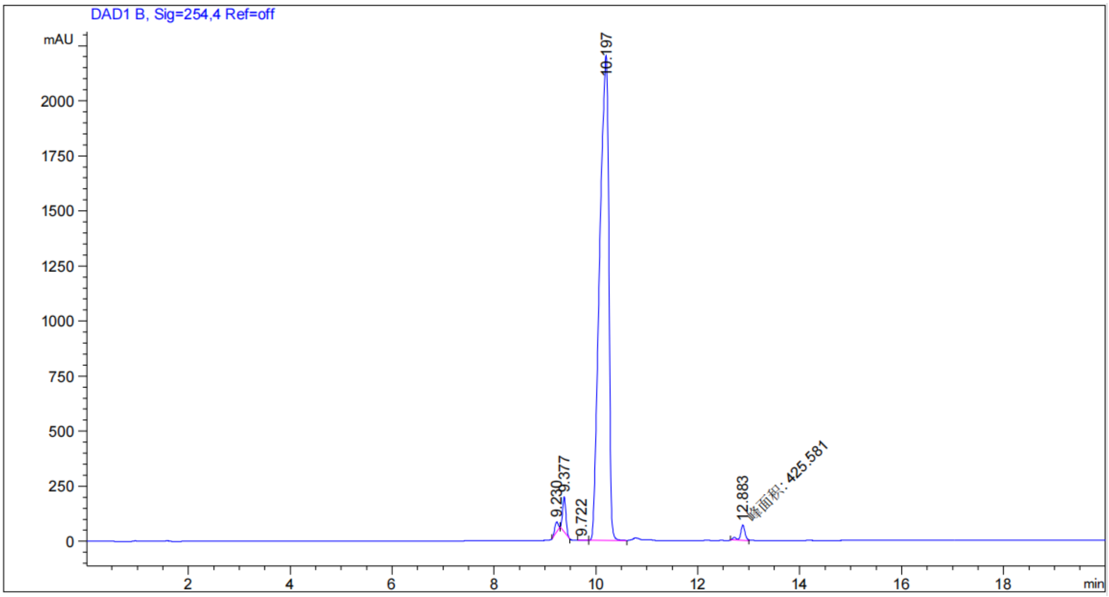

Peak #	RT [min]	Type	Width [min]	Area [mAU*s]	Height [mAU]	Area %
1	9.230	BB	0.0771	212.02026	44.85825	0.7385
2	9.377	BB	0.0718	735.71741	159.68297	2.5625
3	9.722	BB	0.0997	6.86449	1.03070	0.0239
4	10.197	BB	0.1729	2.73305e4	2201.26831	95.1928
5	12.883	MM	0.1030	425.58142	68.88184	1.4823

Fig. S15. HPLC validation of compound **XT10**.

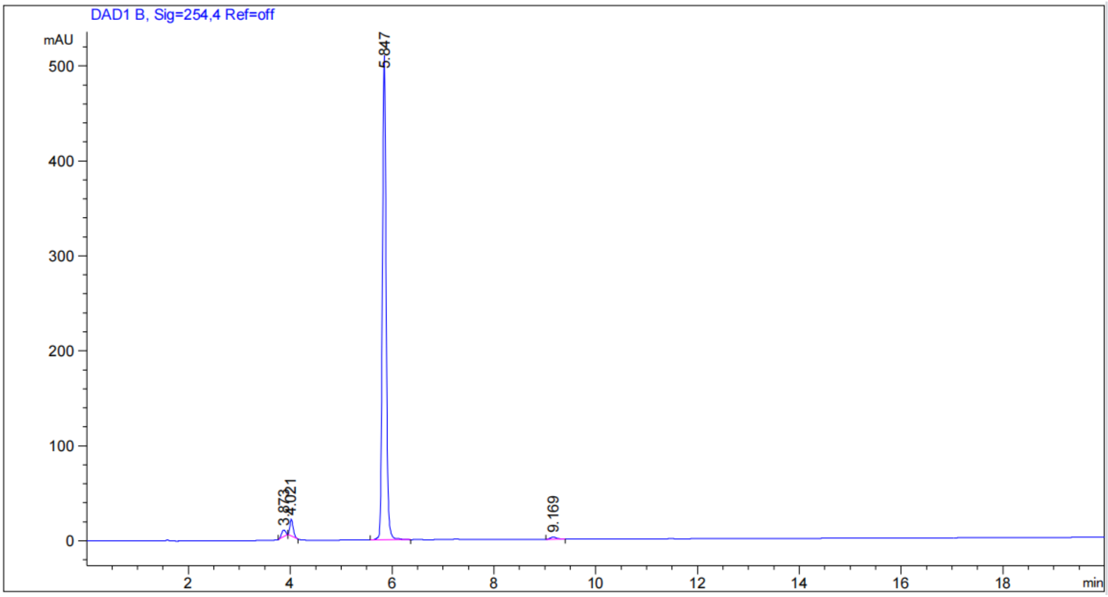

Peak #	RT [min]	Type	Width [min]	Area [mAU*s]	Height [mAU]	Area %
1	3.873	BB	0.0931	38.02700	6.81055	1.3920
2	4.021	BB	0.0711	79.57308	17.48491	2.9128
3	5.847	BB	0.0774	2595.52124	509.66757	95.0097
4	9.169	BB	0.1185	18.72607	2.36253	0.6855

Fig. S16. HPLC validation of compound **XT11**.

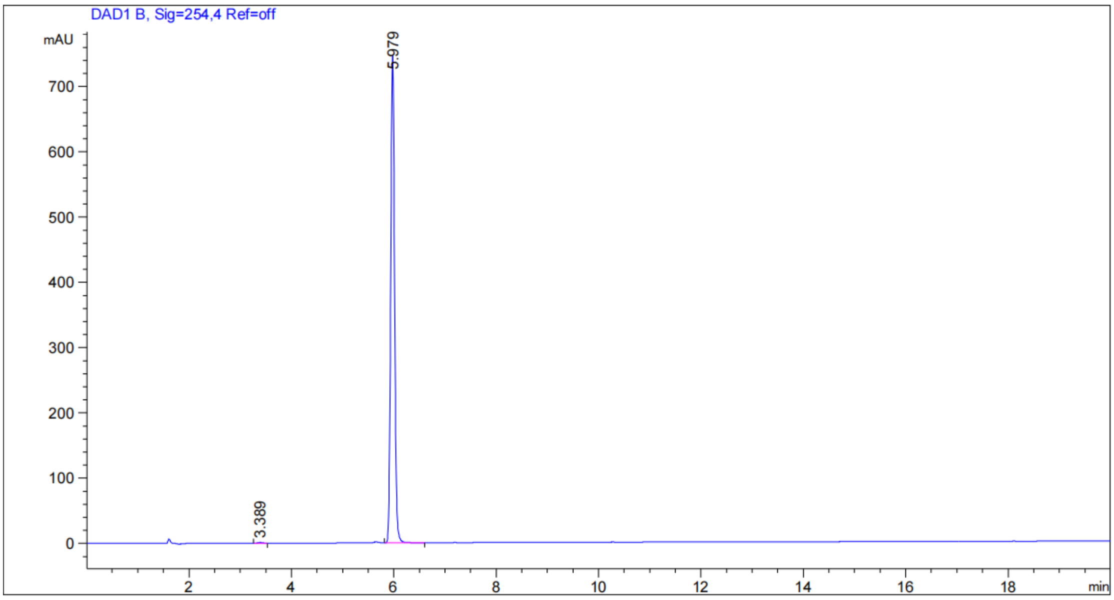

Peak #	RT [min]	Type	Width [min]	Area [mAU*s]	Height [mAU]	Area %
1	3.389	BB	0.0906	6.31090	1.07299	0.1624
2	5.979	BB	0.0806	3879.37988	747.22784	99.8376

Fig. S17. HPLC validation of compound **XT12**.

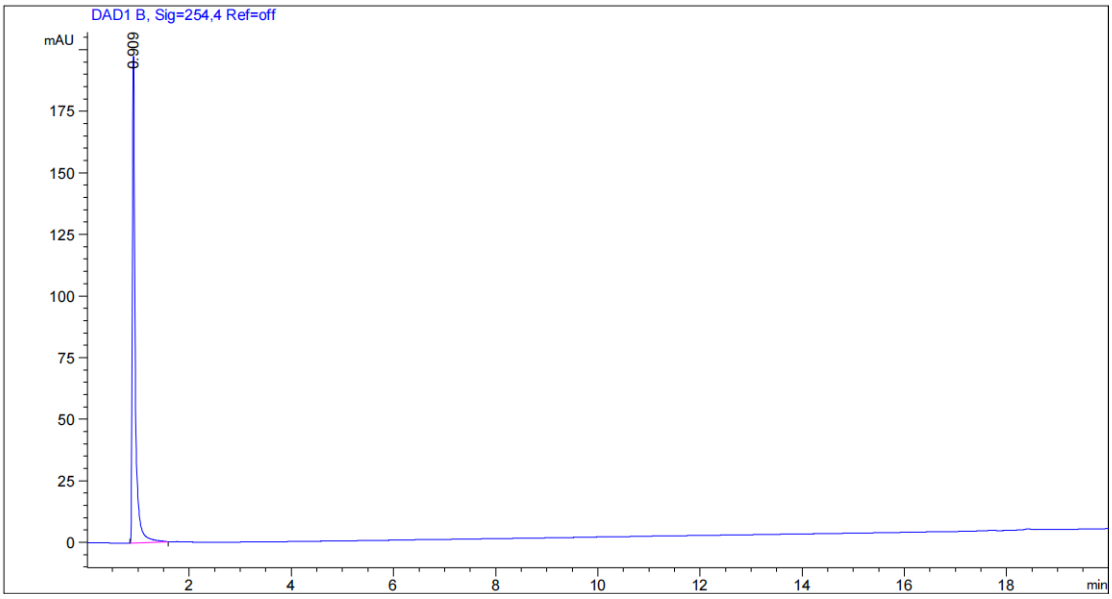

Peak #	RT [min]	Type	Width [min]	Area [mAU*s]	Height [mAU]	Area %
1	0.909	BB	0.0585	789.33539	198.10080	100.0000

Fig. S18. HPLC validation of compound **XT13**.

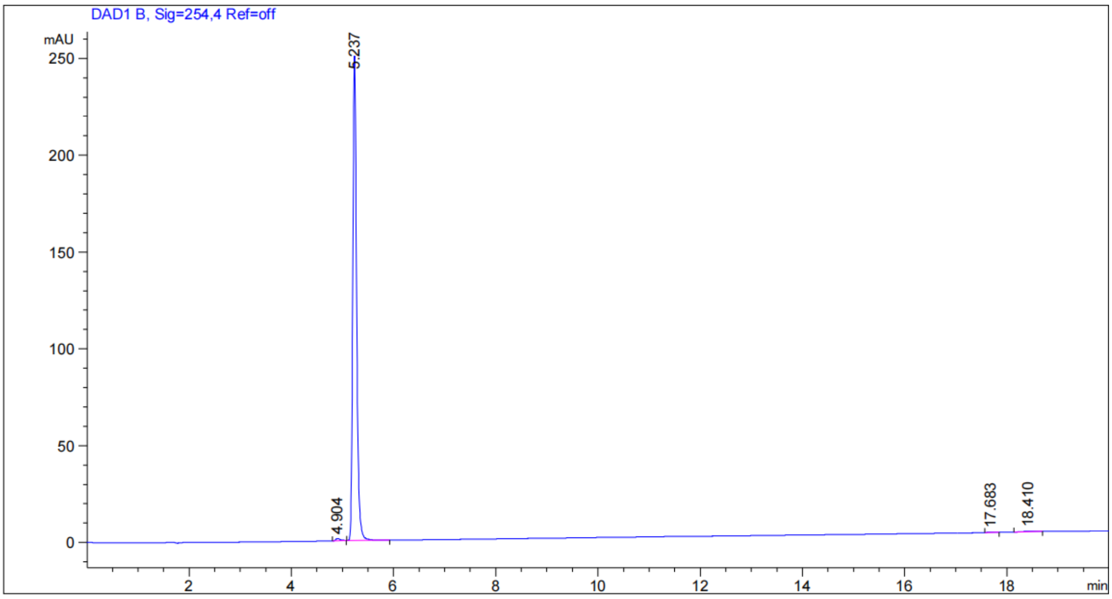

Peak #	RT [min]	Type	Width [min]	Area [mAU*s]	Height [mAU]	Area %
1	4.904	BB	0.0936	7.09755	1.15678	0.5345
2	5.237	BB	0.0811	1314.07471	250.73767	98.9588
3	17.683	BB	0.1042	1.61454	2.34835e-1	0.1216
4	18.410	BB	0.2076	5.11463	3.60297e-1	0.3852

Fig. S19. HPLC validation of compound **XT16**.

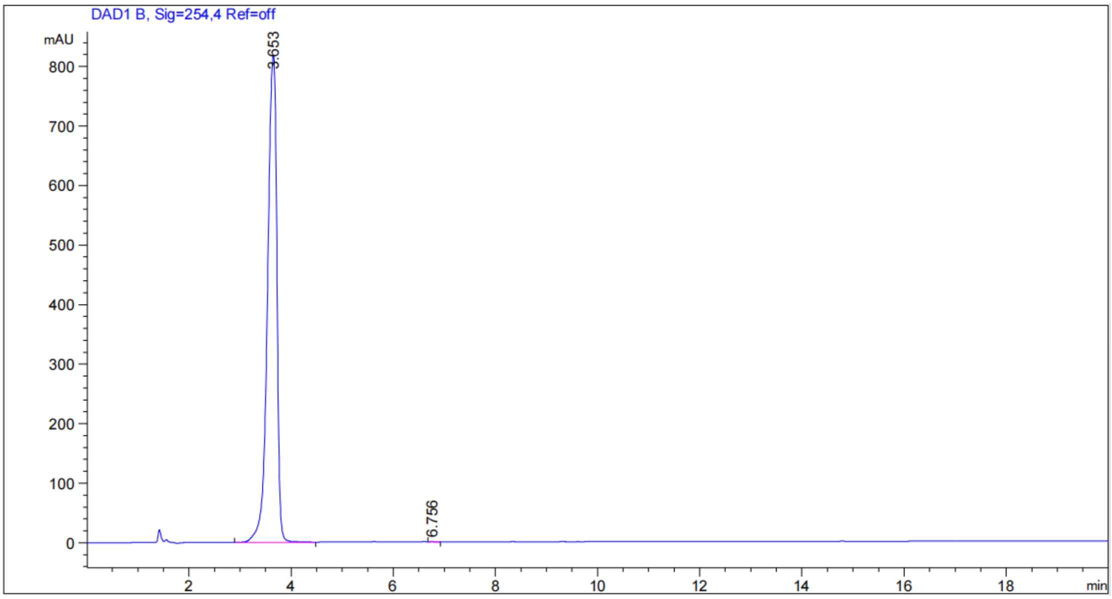

Peak #	RT [min]	Type	Width [min]	Area [mAU*s]	Height [mAU]	Area %
1	3.653	BB	0.2016	1.03325e4	816.94916	99.9769
2	6.756	BB	0.0919	2.38873	3.98599e-1	0.0231

Fig. S20. HPLC validation of compound **XT17**.

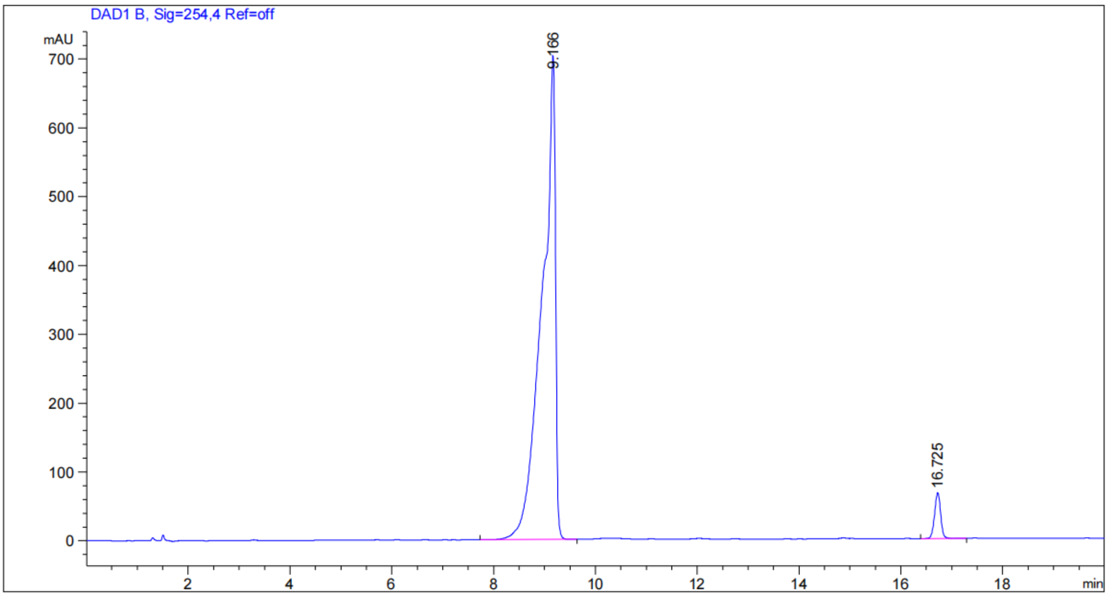

Peak #	RT [min]	Type	Width [min]	Area [mAU*s]	Height [mAU]	Area %
1	9.166	BB	0.2406	1.28269e4	703.78528	95.8711
2	16.725	BB	0.1268	552.42029	66.54612	4.1289

Fig. S21. HPLC validation of compound **XT18**.

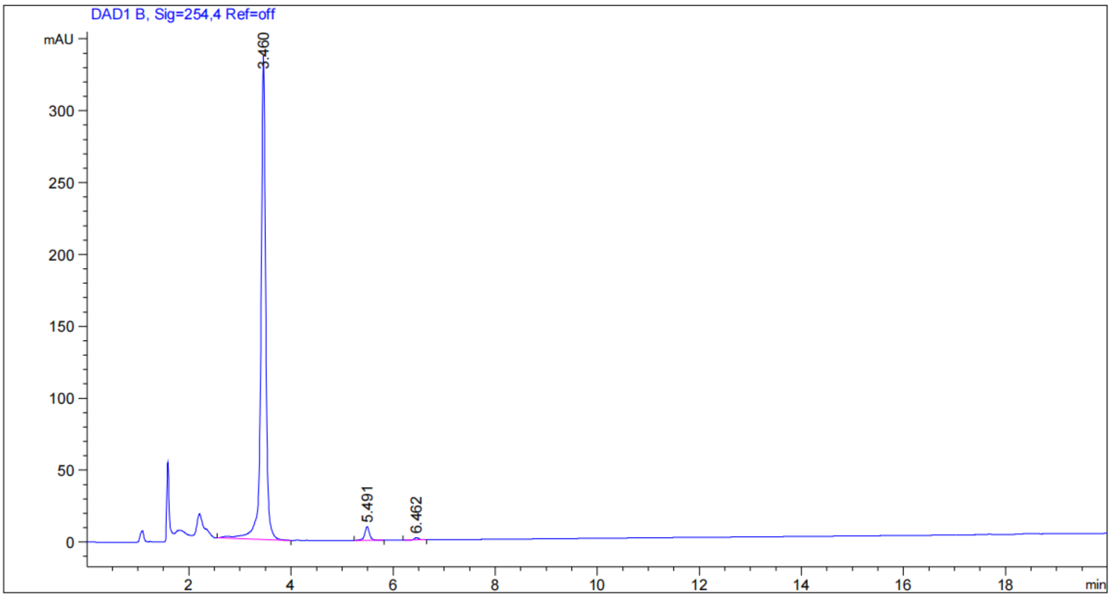

Peak #	RT [min]	Type	Width [min]	Area [mAU*s]	Height [mAU]	Area %
1	3.460	BB	0.0989	2218.88867	336.46219	96.8626
2	5.491	BB	0.0962	62.21436	9.52487	2.7159
3	6.462	BB	0.0927	9.65616	1.59435	0.4215

Fig. S22. HPLC validation of compound **XT19**.

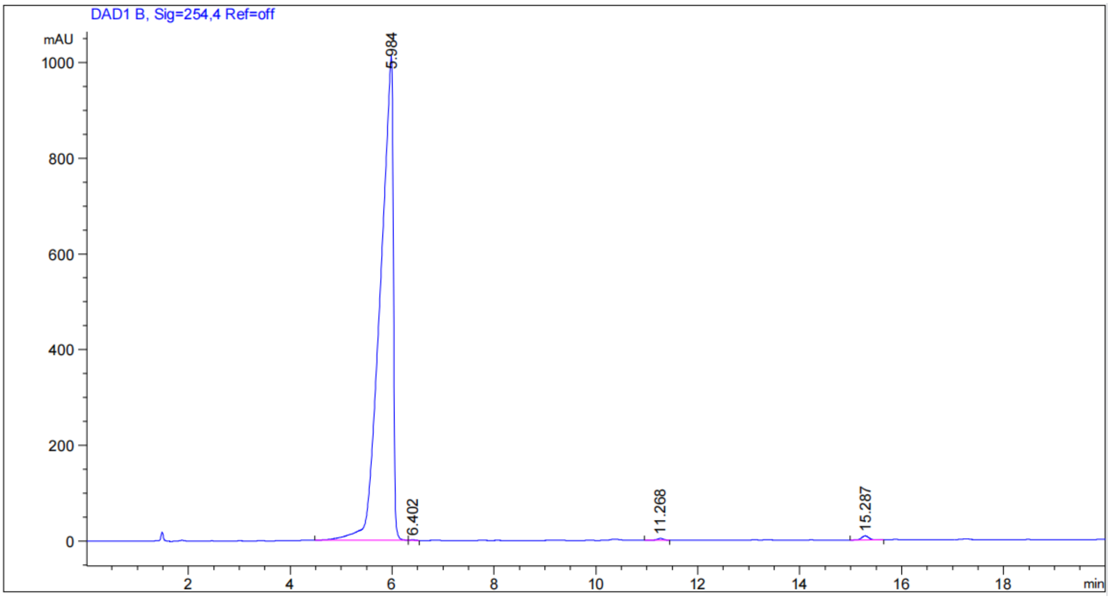

Peak #	RT [min]	Type	Width [min]	Area [mAU*s]	Height [mAU]	Area %
1	5.984	BB	0.2378	1.76824e4	1012.10156	99.3130
2	6.402	BB	0.0858	5.86157	1.07173	0.0329
3	11.268	BB	0.1323	32.22039	3.74655	0.1810
4	15.287	BB	0.1509	84.24142	8.70860	0.4731

Fig. S23. HPLC validation of compound **XT31**.

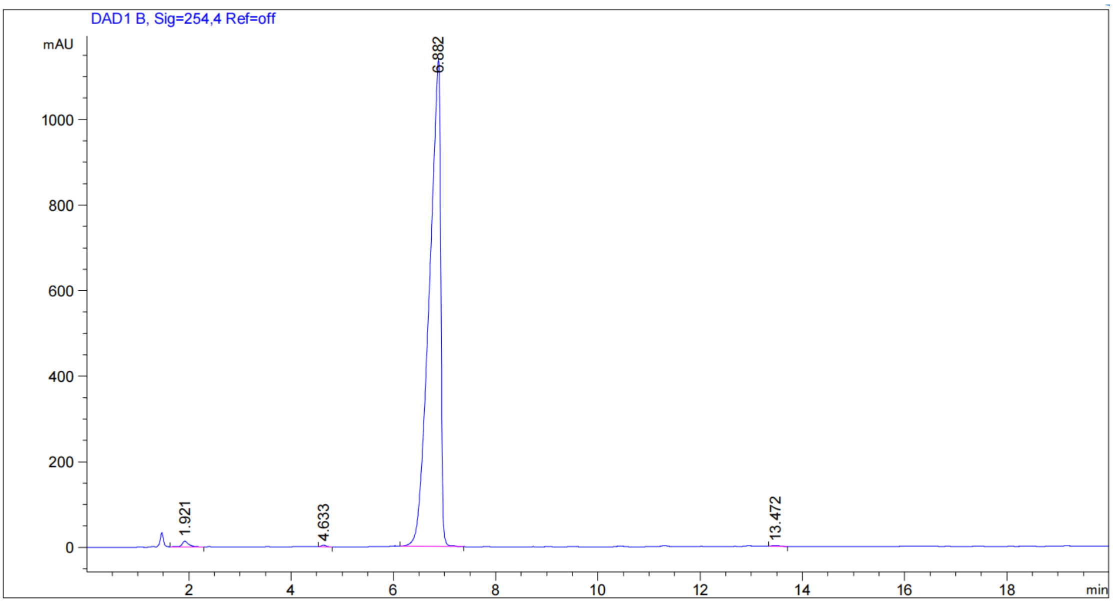

Peak #	RT [min]	Type	Width [min]	Area [mAU*s]	Height [mAU]	Area %
1	1.921	BB	0.1335	122.79779	13.32541	0.6963
2	4.633	BB	0.0988	23.49077	3.76435	0.1332
3	6.882	BB	0.2213	1.74737e4	1136.76074	99.0762
4	13.472	BB	0.1571	16.63669	1.60241	0.0943

Fig. S24. HPLC validation of compound **XT32**.

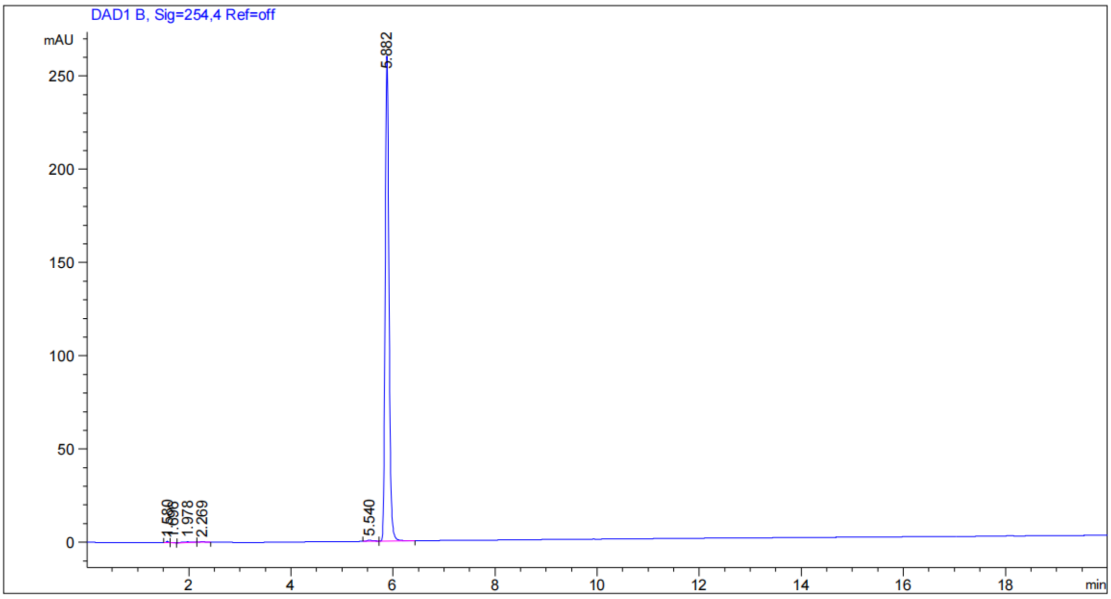

Peak #	RT [min]	Type	Width [min]	Area [mAU*s]	Height [mAU]	Area %
1	1.580	BB	0.0437	1.69224	6.23715e-1	0.1262
2	1.696	BB	0.0850	9.46811e-1	1.93738e-1	0.0706
3	1.978	BB	0.2405	4.91448	3.44331e-1	0.3665
4	2.269	BB	0.1017	1.69878	2.61893e-1	0.1267
5	5.540	BB	0.1013	3.89788	5.73220e-1	0.2907
6	5.882	BB	0.0796	1327.70410	260.08102	99.0193

Fig. S25. HPLC validation of compound **XT33**.

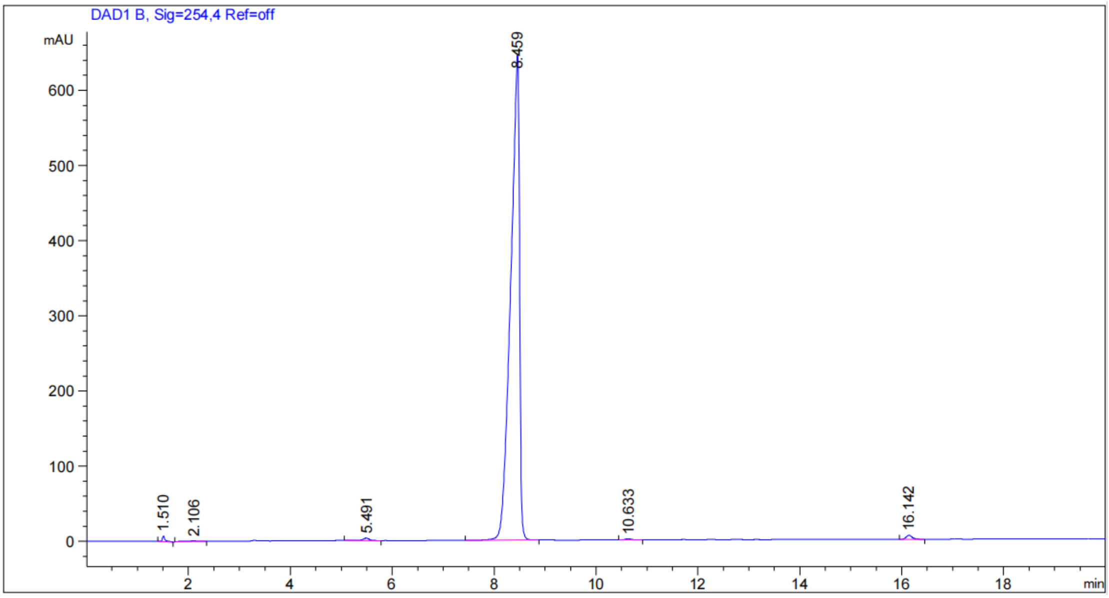

Peak #	RT [min]	Type	Width [min]	Area [mAU*s]	Height [mAU]	Area %
1	1.510	BB	0.0566	28.19057	7.38033	0.3578
2	2.106	BB	0.2239	21.29132	1.21843	0.2702
3	5.491	BB	0.1341	28.77729	3.16473	0.3653
4	8.459	BB	0.1682	7744.85107	644.61835	98.3022
5	10.633	BB	0.1323	11.73341	1.36489	0.1489
6	16.142	BB	0.1223	43.77029	5.41464	0.5556

Fig. S26. HPLC validation of compound **XT34**.

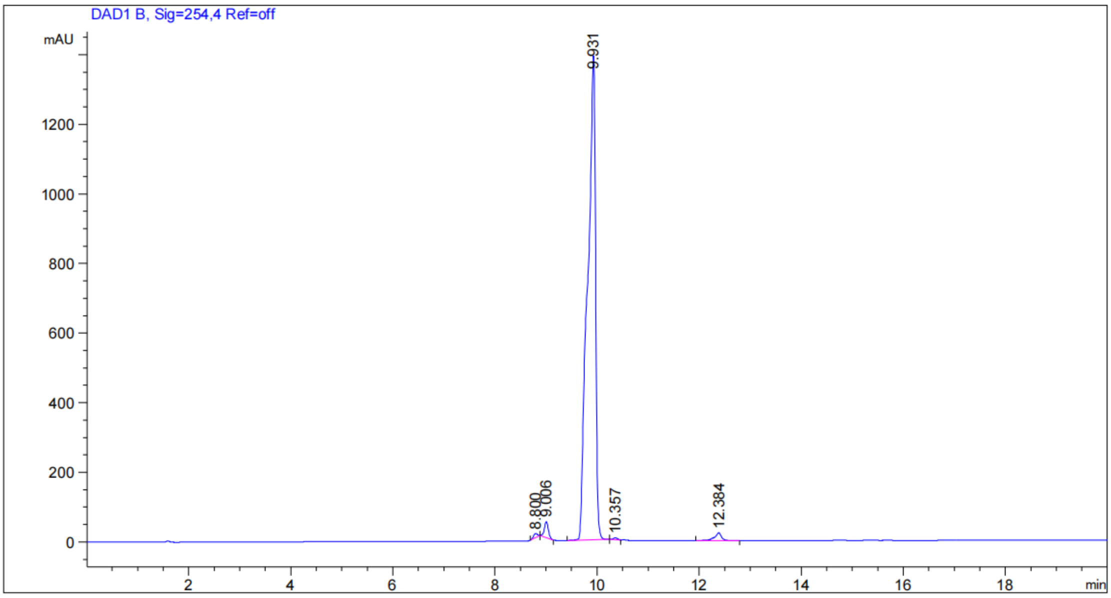

Peak #	RT [min]	Type	Width [min]	Area [mAU*s]	Height [mAU]	Area %
1	8.800	BB	0.0846	61.06458	11.37806	0.3927
2	9.006	BB	0.0833	249.74725	46.04846	1.6060
3	9.931	BB	0.1498	1.49993e4	1390.83252	96.4503
4	10.357	BB	0.0870	27.84045	4.99605	0.1790
5	12.384	BB	0.1326	213.37524	22.51359	1.3721

Fig. S27. HPLC validation of compound **XT35**.

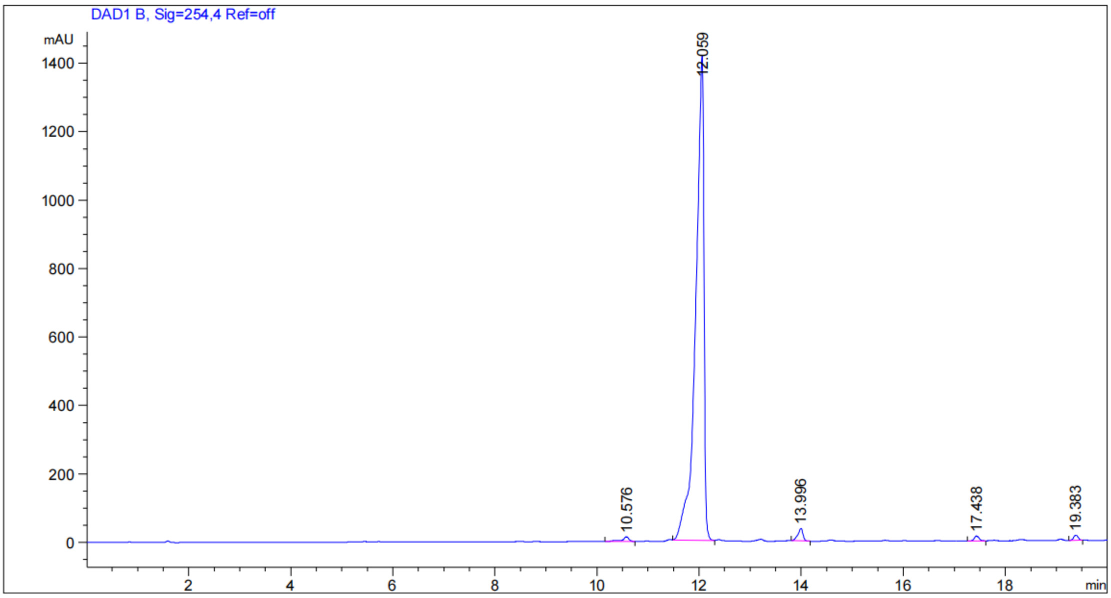

Peak #	RT [min]	Type	Width [min]	Area [mAU*s]	Height [mAU]	Area %
1	10.576	BB	0.1306	126.24835	13.81326	0.7735
2	12.059	BB	0.1539	1.57608e4	1415.06128	96.5691
3	13.996	BB	0.0974	252.10611	37.04534	1.5447
4	17.438	BB	0.0981	88.98345	14.01800	0.5452
5	19.383	BB	0.0941	92.60609	15.41493	0.5674

Fig. S28. HPLC validation of compound **XT37**.

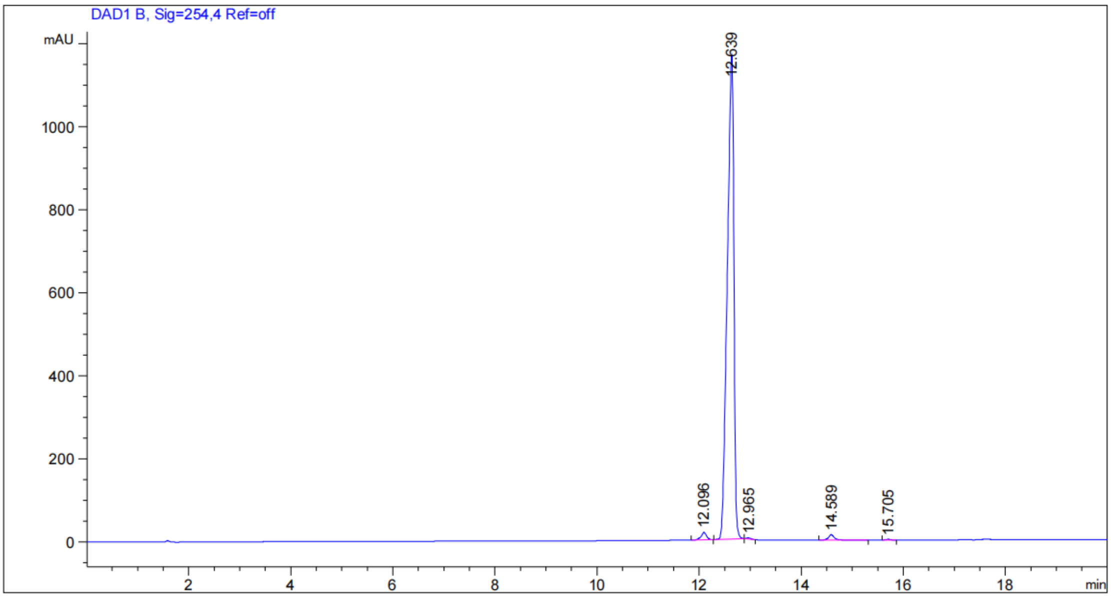

Peak #	RT [min]	Type	Width [min]	Area [mAU*s]	Height [mAU]	Area %
1	12.096	BB	0.1089	134.58775	18.06543	1.2597
2	12.639	BB	0.1305	1.04232e4	1164.20679	97.5579
3	12.965	BB	0.0881	15.11829	2.66846	0.1415
4	14.589	BB	0.1163	101.70264	12.85666	0.9519
5	15.705	BB	0.0998	9.50452	1.50195	0.0890

Fig. S29. HPLC validation of compound **XT38**.

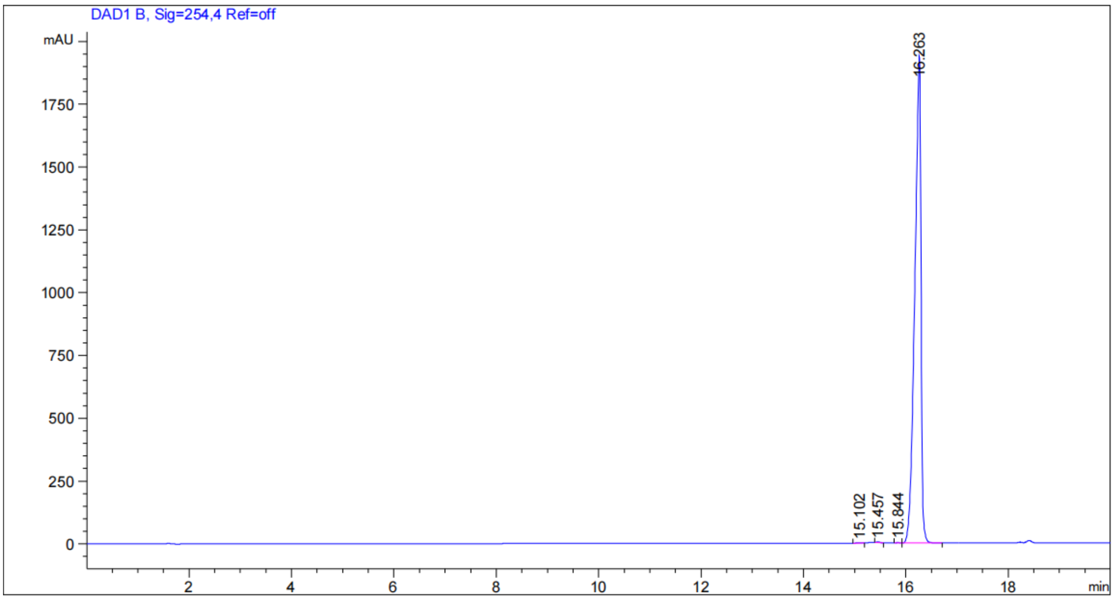

Peak #	RT [min]	Type	Width [min]	Area [mAU*s]	Height [mAU]	Area %
1	15.102	BB	0.0841	7.17633	1.34894	0.0440
2	15.457	BB	0.0721	11.45303	2.47188	0.0702
3	15.844	BB	0.0717	6.81913	1.53774	0.0418
4	16.263	BB	0.1199	1.62822e4	1940.54260	99.8439

Fig. S30. HPLC validation of compound **XT40**.

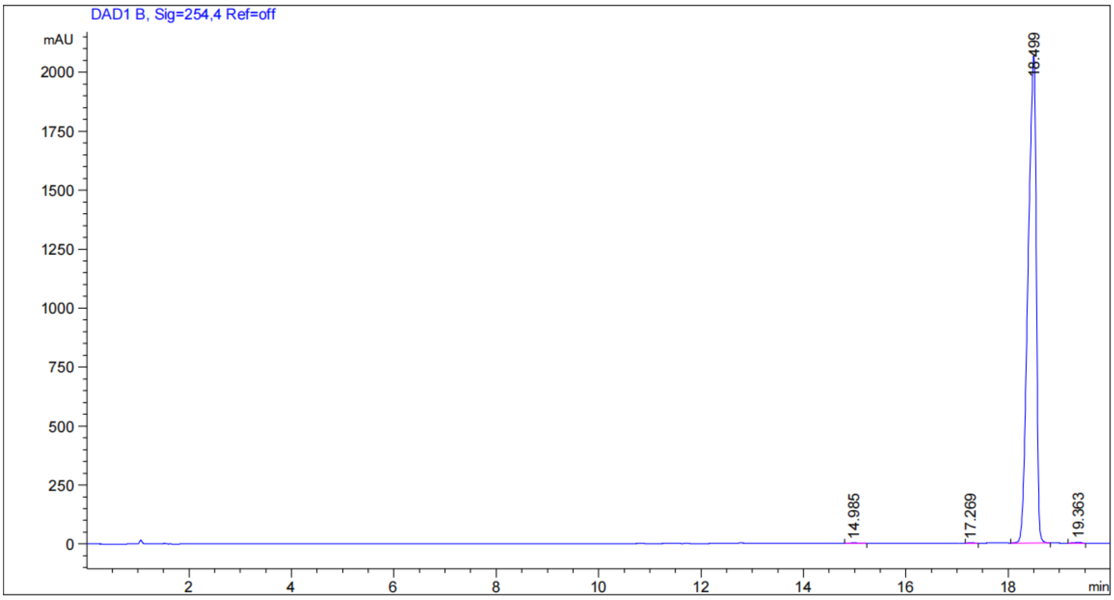

Peak #	RT [min]	Type	Width [min]	Area [mAU*s]	Height [mAU]	Area %
1	14.985	BB	0.1148	14.66707	1.97243	0.0642
2	17.269	BB	0.0963	11.94712	1.98355	0.0523
3	18.499	BB	0.1827	2.27907e4	2066.65967	99.7712
4	19.363	BB	0.1138	25.64459	3.48657	0.1123

Fig. S31. HPLC validation of compound **XT41**.

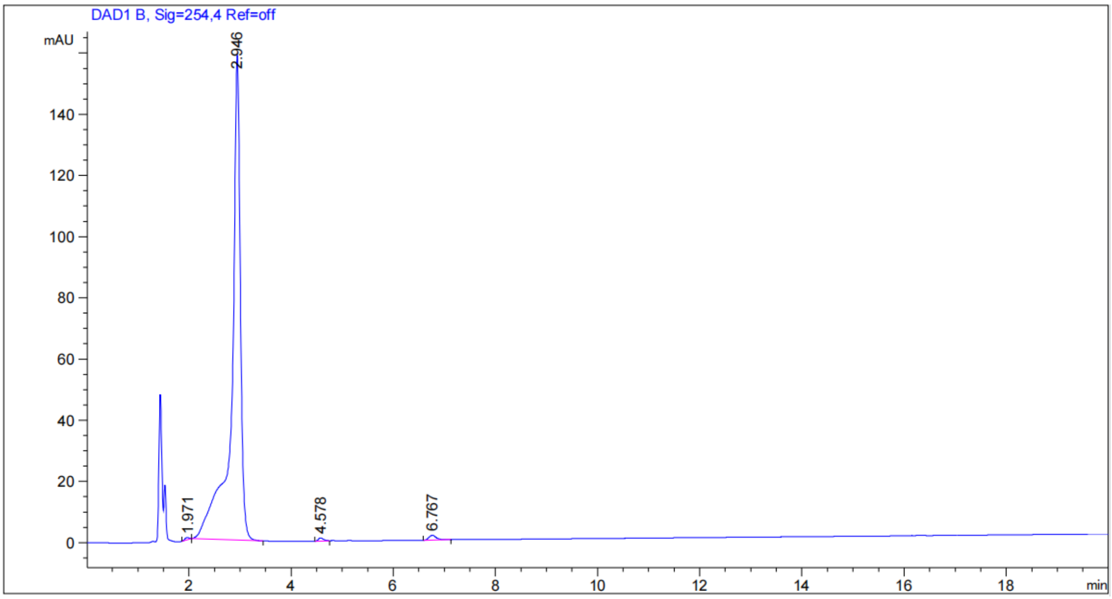

Peak #	RT [min]	Type	Width [min]	Area [mAU*s]	Height [mAU]	Area %
1	1.971	BB	0.0965	4.16262	6.88888e-1	0.2161
2	2.946	BB	0.1680	1899.64307	158.30838	98.6136
3	4.578	BB	0.1090	6.92650	9.97239e-1	0.3596
4	6.767	BB	0.1577	15.61793	1.54761	0.8108

Fig. S32. HPLC validation of compound **XT42**.

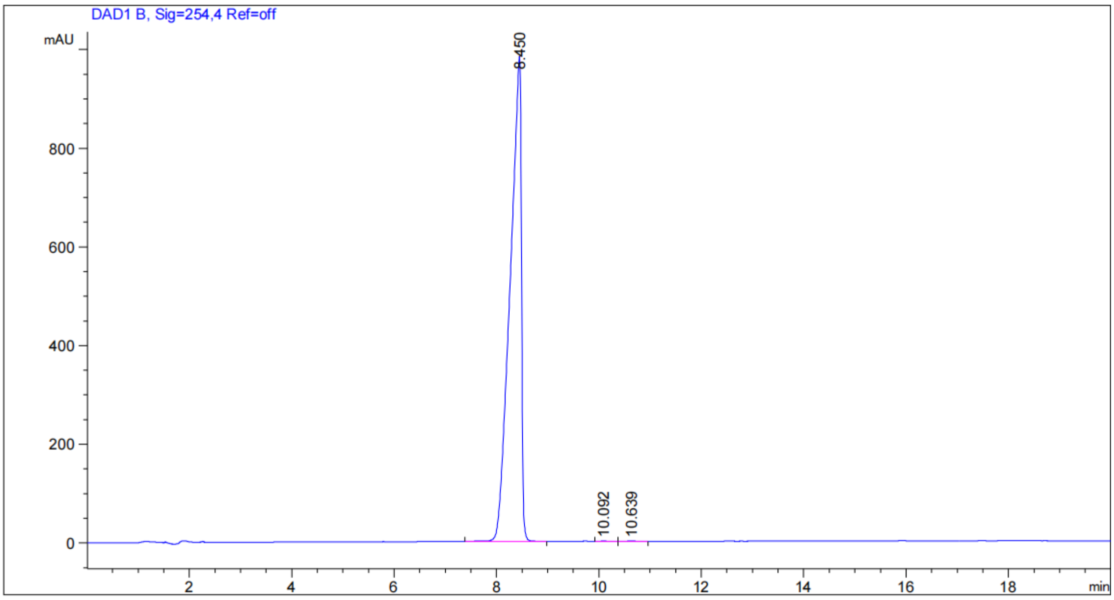

Peak #	RT [min]	Type	Width [min]	Area [mAU*s]	Height [mAU]	Area %
1	8.450	BB	0.2086	1.49180e4	985.38104	99.8617
2	10.092	BB	0.1218	8.28813	1.03088	0.0555
3	10.639	BB	0.1305	12.37154	1.40765	0.0828

Fig. S33. HPLC validation of compound **XT43**.

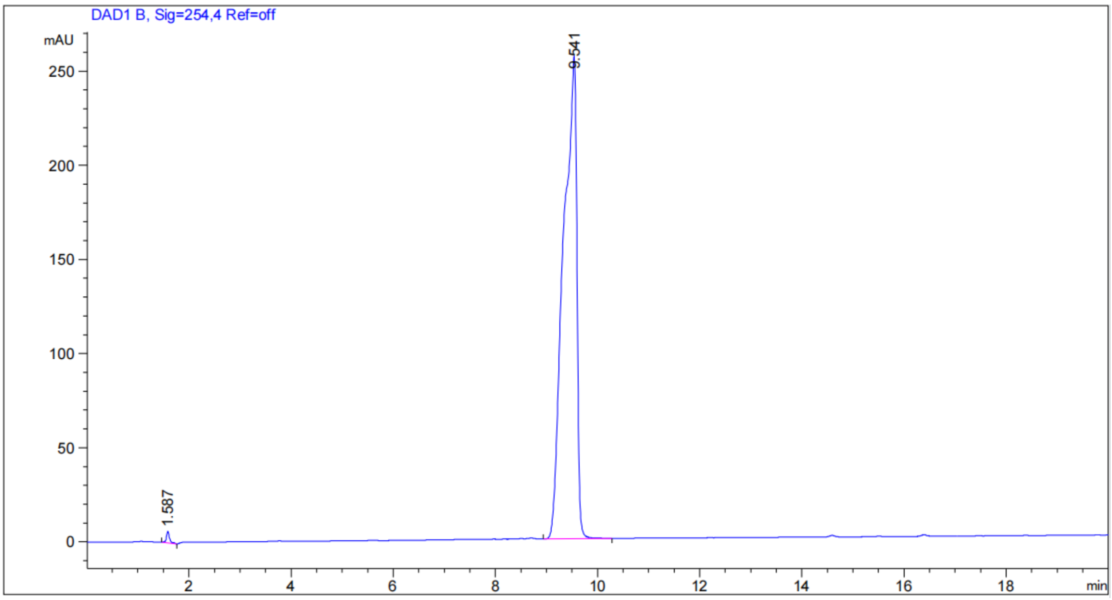

Peak #	RT [min]	Type	Width [min]	Area [mAU*s]	Height [mAU]	Area %
1	1.587	BB	0.0598	24.94770	6.09378	0.5351
2	9.541	BB	0.2392	4637.73438	256.24396	99.4649

Fig. S34. HPLC validation of compound **XT44**.

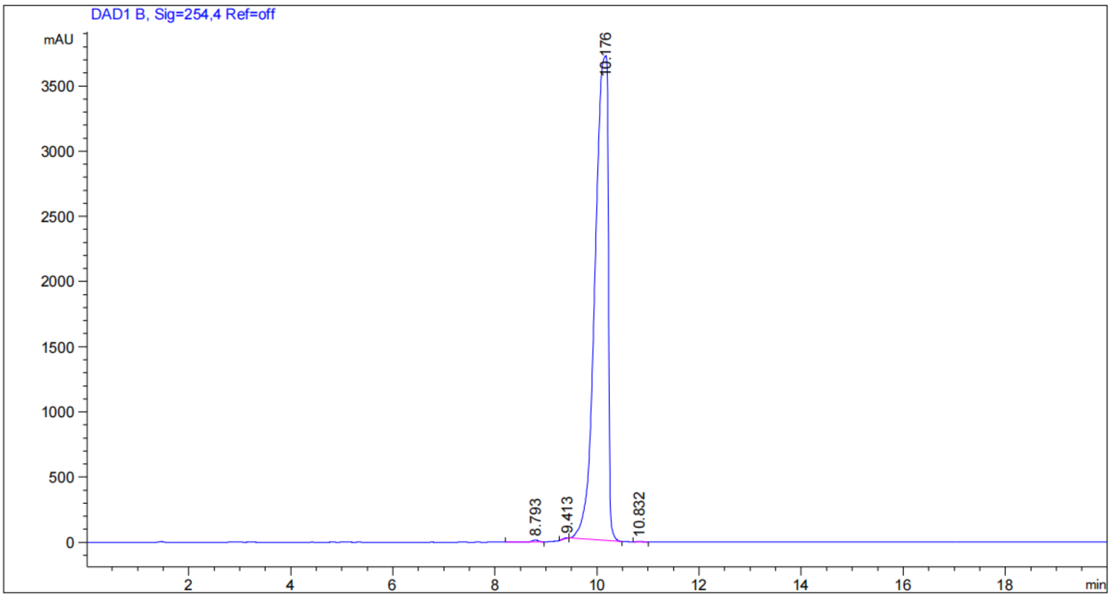

Peak #	RT [min]	Type	Width [min]	Area [mAU*s]	Height [mAU]	Area %
1	8.793	BB	0.1280	127.41756	15.16695	0.1909
2	9.413	BB	0.1335	43.96895	5.15444	0.0659
3	10.176	BB	0.2868	6.65377e4	3717.02734	99.6940
4	10.832	BB	0.1030	32.83716	4.97351	0.0492

Fig. S35. HPLC validation of compound **XT45**.

Supplementary tables

Table S1. Summary of the transcriptome sequence data.

**Sample group**	**CONTROL**	**CPF**	**XT17**
Total reads count	19491176	16116018	18294882
Total clean reads	18541210	15290344	17373620
GC content (%)	52.20	52.22	53.05
Total mapped	18457187 (99.55%)	15117078 (98.87%)	17251607 (99.30%)
Multiple mapped	9944216 (53.63%)	2985711 (19.53%)	10659248 (61.35%)
Uniquely mapped	8512971 (45.91%)	12131367 (79.34%)	6592359 (37.94%)

Table S2. RNA-seq profiles of the most significant gene alterations in *E. coli* ATCC 25922 treated shared with both ciprofloxacin and **XT17**. Gene loci are listed according to NCBI Sterne RefSeq assembly GCF_017357505.1 with the log_2 fold-change.

**Locus (D1792)**	**Gene ID**	**Protein ID**	**Log_2FC_ CPF**	**Log_2FC_ XT17**
RS09390	*recA*	recombinase RecA	6.2751	5.7854
RS18035	*priB*	primosomal replication protein N	3.5288	5.8981
RS05045	*ruvB*	Holliday junction branch migration DNA helicase RuvB	3.4475	4.1938
RS15260	*dnaN*	DNA polymerase III subunit beta	3.0865	2.4370
RS05050	*ruvA*	Holliday junction branch migration protein RuvA	2.8598	3.9191
RS16510	*priA*	primosomal protein N'	2.8529	3.3285
RS17280	*ssb1*	single-stranded DNA-binding protein SSB1	2.6468	3.2652
RS11750	*parE*	DNA topoisomerase IV subunit B	2.4557	2.3205
RS19090	*holD*	DNA polymerase III subunit psi	2.4551	2.6144
RS15250	*gyrB*	DNA topoisomerase (ATP-hydrolyzing) subunit B	2.4048	2.8529
RS07415	*gyrA*	DNA topoisomerase (ATP-hydrolyzing) subunit A	2.2544	2.7733
RS19035	*dnaT*	primosomal protein DnaT	2.2444	1.8873
RS15255	*recF*	DNA replication/repair protein RecF	2.2046	1.6106
RS00675	*holB*	DNA polymerase III subunit delta'	1.9890	3.5242
RS10385	*recJ*	single-stranded-DNA-specific exonuclease RecJ	1.9882	4.2947
RS08890	*recO*	DNA repair protein RecO	1.9736	2.8669
RS21845	*recR*	recombination mediator RecR	1.8874	2.2758
RS05065	*ruvC*	crossover junction endodeoxyribonuclease RuvC	1.8052	2.7878
RS20210	*dnaE*	DNA polymerase III subunit alpha	1.7739	2.4033
RS21835	*dnaX*	DNA polymerase III subunit gamma/tau	1.7268	1.3272
RS21820	*priC*	primosomal replication protein N''	1.7101	2.6334
RS11670	*parC*	DNA topoisomerase IV subunit A	1.5215	2.5193
RS20375	*dnaQ*	DNA polymerase III subunit epsilon	1.3670	2.6769
RS10110	*recB*	exodeoxyribonuclease V subunit beta	1.3516	2.0328
RS14890	*recG*	ATP-dependent DNA helicase RecG	1.1384	1.6673
RS18340	*holC*	DNA polymerase III subunit chi	1.1039	1.8432
RS10120	*recC*	exodeoxyribonuclease V subunit gamma	1.0093	2.4556
RS18760	*cspE*	transcription antiterminator/RNA stability regulator CspE	-1.4229	-4.5456
RS05880	*clbN*	colibactin non-ribosomal peptide synthetase ClbN	-2.3525	-2.7305

Table S3. KEGG pathways enrichment analysis of up- and down-regulated DEGs of the CPF- and compound **XT17**-treatment.

**KEGG pathway**	**Description**	*** *P* value _CPF**	*** *P* value _XT17**
**Up-regulated genes**			
ko03010	Ribosome	3.35E-11	0.00013
ko00970	Aminoacyl-tRNA biosynthesis	0.00019	0.01129
ko03440	Homologous recombination	0.00038	0.00022
ko03430	Mismatch repair	0.00431	0.03094
ko00540	Lipopolysaccharide biosynthesis	0.00569	0.02056
ko00900	Terpenoid backbone biosynthesis	0.01772	0.01438
ko03030	DNA replication	0.03915	0.01427
ko04112	Cell cycle - Caulobacter	0.04713	0.03376
ko00195	Photosynthesis	0.00581	-
ko00130	Ubiquinone and other terpenoid-quinone biosynthesis	0.01076	-
ko00030	Pentose phosphate pathway	0.02461	-
ko03060	Protein export	0.02577	-
ko02030	Bacterial chemotaxis	-	0.00429
ko00280	Valine, leucine and isoleucine degradation	-	0.00683
ko00071	Fatty acid degradation	-	0.01438
ko01053	Biosynthesis of siderophore group nonribosomal peptides	-	0.02210
ko00860	Porphyrin and chlorophyll metabolism	-	0.02227
ko02020	Two-component system	-	0.03219
ko03410	Base excision repair	-	0.04481
ko01210	2-Oxocarboxylic acid metabolism	-	0.04921
**Down-regulated genes**			
ko00920	Sulfur metabolism	0.011075	-
ko00643	Styrene degradation	0.015504	-
ko00300	Lysine biosynthesis	0.018717	-
ko00190	Oxidative phosphorylation	-	2.64E-08
ko02060	Phosphotransferase system (PTS)	-	0.007262
ko00630	Glyoxylate and dicarboxylate metabolism	-	0.010978
ko00910	Nitrogen metabolism	-	0.015284
ko00220	Arginine biosynthesis	-	0.023212

**P* values indicate adjusted *P*-values with the Benjamini-Hochberg procedure.

Table S4. Binding affinity and residual interactions between CPF or **XT17** and DNA gyrase from *E. coli* (PDB code: 6RKV).

**Ligand**	**Binding energy (Kcal/mol)**	**Residual interactions**
		**Hydrogen bond (H), Weak hydrogen bond (WH), Hydrophobic (HP), Cation-Pi (C), Pi-Pi Stacking (P), Ionic interaction (I), Bond (B)**
CPF_1	−7.9	**Chain A:** ASP82 (B)
CPF_2	−7.9	**Chain C:** VAL30(B) ASP36(WH/I) ARG38(I) LEU41(B) LYS42(B) PRO43(HP) ARG46(I) ARG47(H/I) VAL146(H) ASP147(B) ASN148(HP) ILE155(HP) PRO156(H) ASP157(I) VAL158(B) LEU337(B)
CPF_3	−7.9	**Chain A:** VAL30(B) ASP36(H/I) ARG38(B) LEU41(B) LYS42(B) PRO43(HP) ARG46(I) ARG47(H/I) VAL146(HP) ASP147(B) ASN148(HP) ILE155(HP) PRO156(WH) ASP157(B) VAL158(B) LEU337(B)
CPF_4	−6.8	**Chain A:** GLU381(H) ALA384(HP) VAL385(HP) LEU417(HP) ALA421(HP) ALA422(B) HIS471(H/I) LEU474(B) LEU475(HP) TYR478(WH/P) LYS479(B)
XT17_1	−8.3	**Chain B:** ASP426(I) SER427(B)<br>**Chain C:** ALA117(B) ALA119(B)<br>**Chain D:** LYS447(B)
XT17_2	−8.1	**Chain A:** HIS378(I) LEU380(HP) GLU381(H) ALA384(HP) VAL385(HP) LEU417(B) VAL420(B) ALA421(H/HP) ALA422(B) ALA432(B) ARG433(B) LEU437(B) PHE441(B) GLY442(B) VAL443(B) LEU447(B) TYR448(HP) TYR449(B) LEU450(B) THR451(B) GLN454(H) HIS471(B) LEU474(HP) LEU475(B) TYR478(HP)
XT17_3	−7.4	**Chain C:** SER27(H) VAL28(B) VAL30(B) GLY31(B) ARG32(B) ASP36(B) VAL37(B) ARG38(I) PRO43(B) ARG46(WH) ARG47(B) HIS80(H) VAL146(HP) ASP147(B) ILE155(HP) PRO156(WH) ASP157(I) VAL158(B) LEU337(B) HIS338(B) HIS339(B) GLY340(B)<br>**Chain D:** VAL501(B)
XT17_4	−7.4	**Chain A:** SER27(H) VAL28(B) VAL30(HP) GLY31(WH) ARG32(H/I) ASP36(I) VAL37(B) ARG38(B) PRO43(B) ARG46(I) ARG47(B) HIS80(B) VAL146(B) ASP147(B) ILE155(B) PRO156(WH) ASP157(WH) LEU337(B) HIS338(B) HIS339(B) GLY340(B)<br>**Chain B:** VAL501(B)

Table S5. Binding affinity and residual interactions between CPF or **XT17** and DNA gyrase from *S. aureus* (PDB code: 2XCT).

**Ligand**	**Binding energy (Kcal/mol)**	**Residual interactions**
		**Hydrogen bond (H), Weak hydrogen bond (WH), Hydrophobic (HP), Cation-Pi (C), Pi-Pi Stacking (P), Ionic interaction (I), Bond (B)**
CPF	−8.5	**Chain B: S**ER442 SER445(WH) GLY446(WH) ARG447(B) ASP448 SER449 ARG450 ASN587(H) ALA588(HP/H) ASP589(H) TRP592<br>**Chain D:** ALA1094 GLN1095 ASP1096(B) PHE1097(HP) GLY1106 GLN1107 GLY1108(B) ASN1109(H) PHE1110(H) GLY1111(WH) SER1112(WH) MET1113(B) ASP1114 LYS1270 ASP1294 GLU1295(B) THR1296(H) SER1297(B) LEU1298(HP) GLY1301 VAL1302
XT17	−8.3	**Chain B:** SER442 LYS444 SER445(H) GLY446(WH) ARG447 ASP448 SER449 MET586 ASN587(HP) ALA588(WH) ASP589(HP) GLN590 TRP592(B) GLU593 ASN597<br>**Chain D:** TYR1064 ALA1094(H) GLN1095(HP) ASP1096(I) PHE1097(HP) PRO1102 VAL1104(HP) ASP1105(I) GLY1106(B) GLN1107(B) GLY1108 ASN1109(B) PHE1110(H) GLY1111(B) SER1112(B) MET1113(B) ASP1114 ASP1116 PHE1218 PRO1219 THR1220 ALA1221 GLY1222 THR1262 GLU1263 ILE1264 LYS1270(HP) ALA1271 ASP1294 GLU1295 THR1296(WH) SER1297(B) LEU1298(HP) ARG1299 THR1300 GLY1301(H) VAL1302(HP) ARG1485

Table S6. Pharmacokinetic (PK) analysis of **XT17** via intravenous (IV) administration in mice.

**Individual and mean plasma concentration-time data of XT17 after an IV dose at 5 mg/kg in male CD-1 mice**								
Dose (mg/kg)	Dose route	Sampling time (hr)	Concentration (ng/mL)			**Mean (ng/mL)**	SD	CV (%)
			Individual					
5	IV	0.083	16530	21320	22970	**20273**	3345	16.5
		0.25	7057	8243	6777	**7359**	778	10.6
		0.5	4038	4582	2259	**3626**	1215	33.5
LLOQ=10 ng/mL		1	1497	2783	1022	**1767**	911	51.6
		2	265	381	201	**282**	91.5	32.4
		4	20.8	37.2	11.9	**23.3**	12.8	55.1
		8	BQL	BQL	BQL	**BQL**	NA	NA
		24	BQL	BQL	BQL	**BQL**	NA	NA

PK parameters	Unit	Estimated Value
Rsq_adjusted		0.989
CL	L/hr/kg	0.581
V_{ss}	L/kg	0.244
No_points_lambda_z		4
$T_{1/2}$	hr	0.478
AUC_{last}	hr*ng/mL	8593
AUC_{INF}	hr*ng/mL	8609
MRT_{last}	hr	0.412
MRT_{INF}	hr	0.420
CL	mL/min/kg	9.68

XT17 was prepared in 0.5% DMSO + 99.5% D5W to yield a clear solution at 1 mg/mL for IV dosing.

PK parameters were estimated by non-compartmental model using WinNonlin 8.2.

NA = not available.

If the adjusted Rsq (linear regression coefficient of the concentration value on the terminal phase) is less than 0.9, $T_{1/2}$ might not be accurately estimated.

The PK data highlighted in red represent the one calculated the $T_{1/2}$.

BQL = below quantifiable limit.

Table S7. PK analysis of **XT17** via intraperitoneal (IP) administration in mice.

**Individual and mean plasma concentration-time data of XT17 after an IP dose at 20 mg/kg in male CD-1 mice**									
Dose (mg/kg)	Dose route	Sampling time (hr)	Concentration (ng/mL)			**Mean (ng/mL)**	SD	CV(%)
			Individual					
20	IP	0.083	17430	17970	17030	**17477**	472	2.70
		0.25	20580	17330	17190	**18367**	1918	10.4
		0.5	14090	14040	20.8*	**14065**	NA	NA
LLOQ=10 ng/mL		1	7334	10050	10190	**9191**	1610	17.5
		2	2101	3003	3204	**2769**	587	21.2
		4	479	425	BQL*	**452**	NA	NA
		8	74.9	98.3	97.0	**90.1**	13.1	14.6
		24	BQL	BQL	BQL	**BQL**	NA	NA
PK parameters		Unit	Estimated Value					
Rsq_adjusted			0.932					
T_{max}		hr	0.250					
C_{max}		ng/mL	18367					
No_points_lambda_z			5					
$T_{1/2}$		hr	1.02					
AUC_{last}		hr*ng/mL	23873					
AUC_{INF}		hr*ng/mL	24006					
MRT_{last}		hr	1.08					
MRT_{INF}		hr	1.13					
F		%	69.7					

F = ($AUC_{INF\text{-}IP}$/mean $AUC_{INF\text{-}IV}$)/($Dose_{IP}$/$Dose_{IV}$)*100%, AUC_{last} was alternatively used for F calculation when AUC_{INF} was not available or beyond 120% of AUC_{last}.

XT17 was prepared in 0.5% DMSO+ 99.5% D5W to yield a clear solution at 2 mg/mL for IP dosing.

PK parameters were estimated by non-compartmental model using WinNonlin 8.2.

NA = not available

If the adjusted Rsq (linear regression coefficient of the concentration value on the terminal phase) is less than 0.9, $T_{1/2}$ might not be accurately estimated.

The PK data highlighted in red represent the one calculated the $T_{1/2}$.

*Abnormal data, not involved in parameter calculation.

BQL = below quantifiable limit.

Table S8. PK analysis of **XT17** via subcutaneous (SC) administration in mice.

**Individual and mean plasma concentration-time data of XT17 after an SC dose at 40 mg/kg in male CD-1 mice**								
Dose (mg/kg)	Dose route	Sampling time (hr)	Concentration (ng/mL) Individual			**Mean (ng/mL)**	SD	CV(%)
40	SC	0.083	7397	7327	8459	**7728**	634	8.21
		0.25	9237	5453	5392	**6694**	2203	32.9
		0.5	6143	6320	9549	**7337**	1917	26.1
LLOQ=10 ng/mL		1	7081	13220	5730	**8677**	3992	46.0
		2	6460	4999	2407	**4622**	2053	44.4
		4	3071	2890	4731	**3564**	1015	28.5
		8	1896	4481	2052	**2810**	1450	51.6
		24	103	260	201	**188**	79.1	42.0

PK parameters	Unit	Estimated Value
Rsq_adjusted		0.978
T_{max}	hr	1.00
C_{max}	ng/mL	8677
No_points_lambda_z		4
$T_{1/2}$	hr	4.68
AUC_{last}	hr*ng/mL	58848
AUC_{INF}	hr*ng/mL	60120
MRT_{last}	hr	5.54
MRT_{INF}	hr	6.07
F	%	87.3

F = ($AUC_{INF\text{-}SC}$/mean $AUC_{INF\text{-}IV}$)/($Dose_{SC}$/$Dose_{IV}$)*100%, AUC_{last} was alternatively used for F calculation when AUC_{INF} was not available or beyond 120% of AUC_{last}.

XT17 was prepared in 0.5% DMSO+ 99.5% D5W to yield a clear solution at 4 mg/mL for SC dosing.

PK parameters were estimated by non-compartmental model using WinNonlin 8.2.

If the adjusted Rsq (linear regression coefficient of the concentration value on the terminal phase) is less than 0.9, $T_{1/2}$ might not be accurately estimated.

The PK data highlighted in red represent the one calculated the $T_{1/2}$.

Table S9. Primers used in real-time quantitative RT-PCR experiment.

**Primers**	**Base pair (bp)**	**Template**	**Sequence (5'– 3')**
GAPDH-F	175	3270589	ACCCGCTTTAGCATCGAACA
GAPDH-R		3270763	AGTTGACCTGACCGTTCGTC
clbN-F	157	3019187	CAAGCGAGCATACGAACAGC
clbN-R		3019343	TGGAAAGCAGTCGCGGTATT
cspE-F	142	4591192	TACGTTTGCAGCAGAAGGGC
cspE-R		4591333	CATTACTCCGGAAGACGGCAG
gyrA-F	147	166917	GGATGTGTTCCATCAGCCCT
gyrA-R		166936	CTAACCTGCTGGTGAACGGT
gyrB-F	135	852626	GGTTACCGGCGAGACTGAAA
gyrB-R		852760	CCGGAGTTGAGGAACGACAA
galE-F	109	4476724	GCAAAGCCCTTACGGCAAAA
galE-R		4476832	AACTGGGTTGAAGTAGCGCA
ompC-F	116	140091	GTATGCTACAGACGGACGCA
ompC-R		140206	CCCAGACCTACAACGCAACT
parC-F	94	1589947	TGCAGGAGCCGAAAATGCTA
parC-R		1590040	GGTGGAATATCGGTCGCCAT
parE-F	114	1572691	CCGATACCACTCGCCCTAAC
parE-R		1572804	ACGACTGGTCGGCATGTAAG
